# Ring-Enlargement of in Situ Generated Cyclopropanones with Carbonyls and Imines: Synthesis of γ‑Butyrolactones and -lactams

**DOI:** 10.1021/acs.orglett.6c00839

**Published:** 2026-04-01

**Authors:** Marvin Lange, Arthur Semmelmaier, Richard Herzog, Heinrich F. von Köller, Daniel B. Werz

**Affiliations:** Institute of Organic Chemistry, 9174 Albert-Ludwigs-Universität Freiburg, Albertstraße 21, 79104 Freiburg, Germany

## Abstract

We report a ring-enlargement of in situ generated cyclopropanones with aldehydes, ketones, and aldimines to access γ-butyrolactones and γ-lactams. Using bench-stable 1-sulfonylcyclopropanols as cyclopropanone precursors, the reaction proceeds under mild conditions and affords the products in yields of up to 84% across a range of carbonyl and imine coupling partners. Mechanistic experiments and DFT studies provide insight into the reaction pathway.

## Graphical abstract

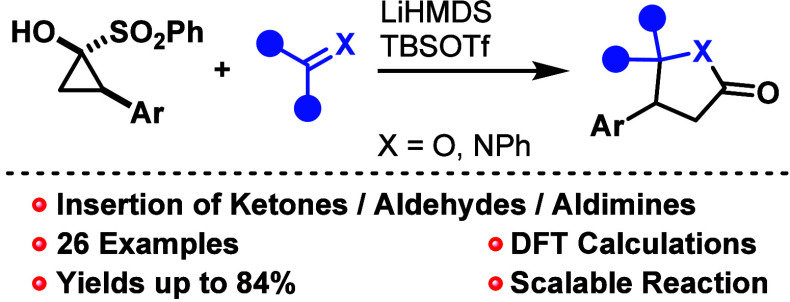

Rearrangement and cycloaddition reactions of small ring systems affording normal- or medium-sized rings have become a major strategy to access more complex structures.^1,2^ In the last 20 years especially donor–acceptor (D–A) cyclopropanes, originally invented by Wenkert and Reissig in the 1970/80s, have attracted the interest of synthetic organic chemists.^3^ They are easily available building blocks and have been widely exploited as masked 1,3-zwitterions in organic synthesis and methodology.^4,5^ Activated by Lewis acids numerous 1,2-, 1,3- and 1,4-dipoles have been easily inserted into the strained system.^6^ While nonpolarized cyclopropanes do not show such reactivity, other three-membered ring systems, such as cyclopropanones, which are even more strained, can be used in a similar manner.^7^

As the highly reactive cyclopropanones are difficult to handle and isolate, they are most conveniently formed in situ. Previously, cyclopropanone ketals have been utilized as precursors.^8^ However, this was associated with a number of drawbacks, in particular the relatively harsh conditions needed for the release of the highly reactive cyclopropanone. Furthermore, due to the formation of stable esters, cyclopropanone ketals are prone to generate homoenolates which can result in the occurrence of undesired reaction pathways. More recently, Chen and co-workers identified bench-stable 1-sulfonylcyclopropanols (SCPs) as suitable precursors for the in situ generation of cyclopropanones under mildly basic reaction conditions eliminating the aforementioned issues.^9^ Later on, Lindsay and co-workers developed a novel enantioselective approach to such cyclopropanone surrogates.^10^

For D–A cyclopropanes the insertion of aldehydes and imines was reported more than a decade ago.^11^ These reactions furnished highly substituted tetrahydrofurans and pyrrolidines. With the aldehyde or imine being the electron-withdrawing group rearrangements in highly stereoselective fashion were observed.^12^ In these reported reactions, the oxygen or nitrogen nucleophile invariably attacks the donor-substituted position of the three-membered ring. We envisioned an alternative mode wherein the carbonyl group would be installed in the new five-membered ring with reversed regiochemistry. To achieve this, we reasoned that cyclopropanones would serve as ideal substrates. Nucleophilic attack of a carbonyl compound at the electrophilic cyclopropanone carbonyl would generate an oxocarbenium cyclopropanolate intermediate in situ—a formal D–A cyclopropane equivalent—that could rearrange to the corresponding γ-butyrolactones or γ-butyrolactams.^13^ We anticipated that even the weakly nucleophilic oxygen of aldehydes or ketones would be sufficient to initiate this transformation, driven by relief of the substantial ring strain (49 kcal/mol) and rehybridization of the formally sp-hybridized carbonyl carbon (Scheme 1).^14^ Importantly, unlike previously reported cyclopropanone reactions that proceed via a 1,2-shift with retention of configuration,^15^ product formation in this case formally requires a 1,3-shift, which in a concerted scenario would have to be antarafacial. For steric reasons this appears highly unlikely, rendering a stepwise mechanism with loss of stereochemical information more plausible.

**Scheme 1:**
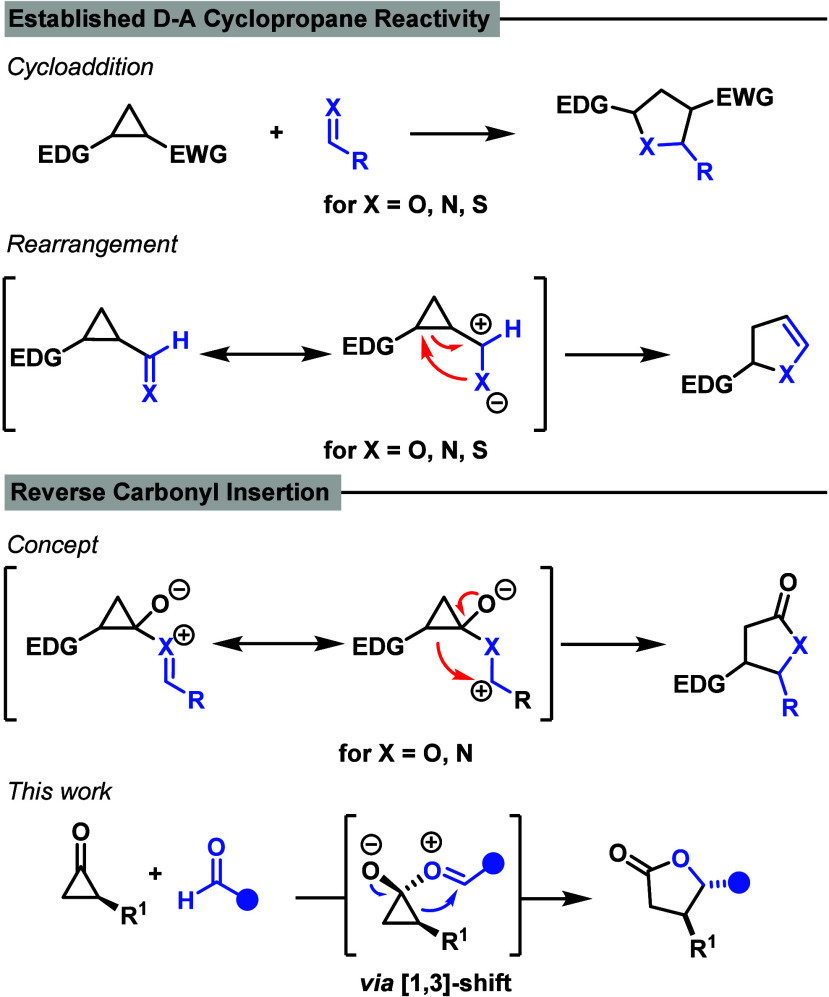
Previous Work and This Work

We initiated our study using SCP **1a** and benzaldehyde (8 equiv) as starting materials in THF with LiHMDS as the base to generate the corresponding cyclopropanone in situ. The reaction carried out from 0 °C to room temperature afforded lactone **2a** in 44% NMR yield. Addition of Sn­(OTf)_2_ (10 mol %) slightly increased the yield to 48%, whereas lowering the temperature to −20 °C had no effect. Notably, the addition of Lewis acids led to a cleaner conversion and a significant reduction in side products, as concluded from a comparison of GC/MS analyses of the crude reaction mixtures. Performing the reaction in DMF improved the yield to 58%, and a 1:1 mixture of THF and DMF provided **2a** in 61% yield. When SnCl_4_ was employed as Lewis acid, the product was obtained in 63% yield. Dilution of the reaction mixture to 0.02 M further enhanced the yield to 71%. Conducting the reaction at −78 °C afforded **2a** in 73% yield, which was further improved to 80% upon use of TBSOTf as Lewis acid. The diastereomeric ratio remained largely unaffected under all conditions, consistently ranging from 1.3:1 to 1.6:1 (Table 1).

**Table 1:**
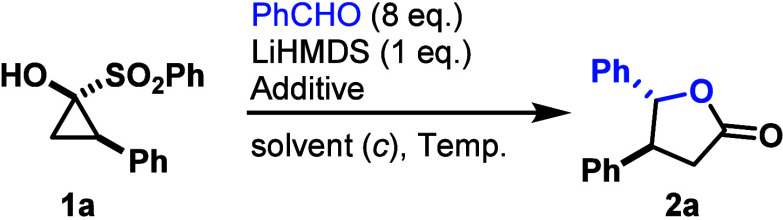
Optimization Table of the Reaction Conditions^a^ ^,^ ^b^

Entry	Solvent (*c*/M)^c^	*T* [°C]	Additive (mol %)	Yield (*dr*) [%]^d^
1	THF (0.1)	0 to r.t.	–	44 (1.4:1)
2	THF (0.1)	0 to r.t.	Sn(OTf)_2_ (10)	48 (1.6:1)
3	THF (0.1)	–20	Sn(OTf)_2_ (10)	48 (1.4:1)
4	DMF (0.1 M)	–20	Sn(OTf)_2_ (10)	58 (1.3:1)
5	THF/DMF (1:1, 0.1)	–20	Sn(OTf)_2_ (10)	61 (1.3:1)
6	THF/DMF (1:1, 0.1)	–20	SnCl_4_ (15)	63 (1.3:1)
7	THF/DMF (1:1, 0.02)	–20	SnCl_4_ (15)	71 (1.3:1)
8	THF/DMF (1:1, 0.02)	–78	SnCl_4_ (15)	73 (1.3:1)
9	THF/DMF (1:1, 0.02)	–78	TBSOTf (15)	80 (1.3:1)
10^e^	THF/DMF (1:1, 0.02)	–78 to r.t.	TBSOTf (15)	77 (1:1)

a See Supporting Information for detailed information.

b Reactions were carried out on a 0.06 mmol scale with respect to the SCP **1a**.

c Concentration with respect to SCP **1a**.

d Yields refer to ^1^H NMR yield against a 1,3,5-trimethoxybenzene standard. Diastereomeric ratios (given in parentheses) were determined by ^1^H NMR with the *anti*-diastereomer being the major stereoisomer formed.

e Reaction was performed on a 0.2 mmol scale with respect to **1a**, the yield refers to isolated and purified products.

With these optimized conditions in hand, next, the substrate scope was investigated. *para*-Substitution of the benzaldehyde with a methoxy group gave **2b** with a yield of 84%, whereas fluorine in the same position slightly reduced the yield of the corresponding product **2c** to 74%. Using furfural, the oxazol-substituted γ-butyrolactone **2d** was accessible in 72% yield. When sterically demanding pivaldehyde was used, **2e** was obtained in 59%. Notably, most likely due to the impact of the bulky *tert*-butyl group, the diastereomeric ratio increased to 5:1 in favor of the *anti*-diastereomer. The styryl-substituted γ-butyrolactone **2f** was obtained in 52% yield without double bond isomerization or the formation of the corresponding seven-membered analog resulting from the (4+3)-cycloaddition of cinnamaldehyde. Methyl, methoxy and fluorine *para*-substitution of the aryl moiety of the SCP gave the corresponding products **2g**, **2h** and **2i** in moderate to good yields of 66%, 49% and 70%, respectively (Scheme 2, top). Unfortunately, aldehydes bearing electron-withdrawing groups (e.g., nitro or ester substituents) in the *para* position did not yield the desired products.

**Scheme 2:**
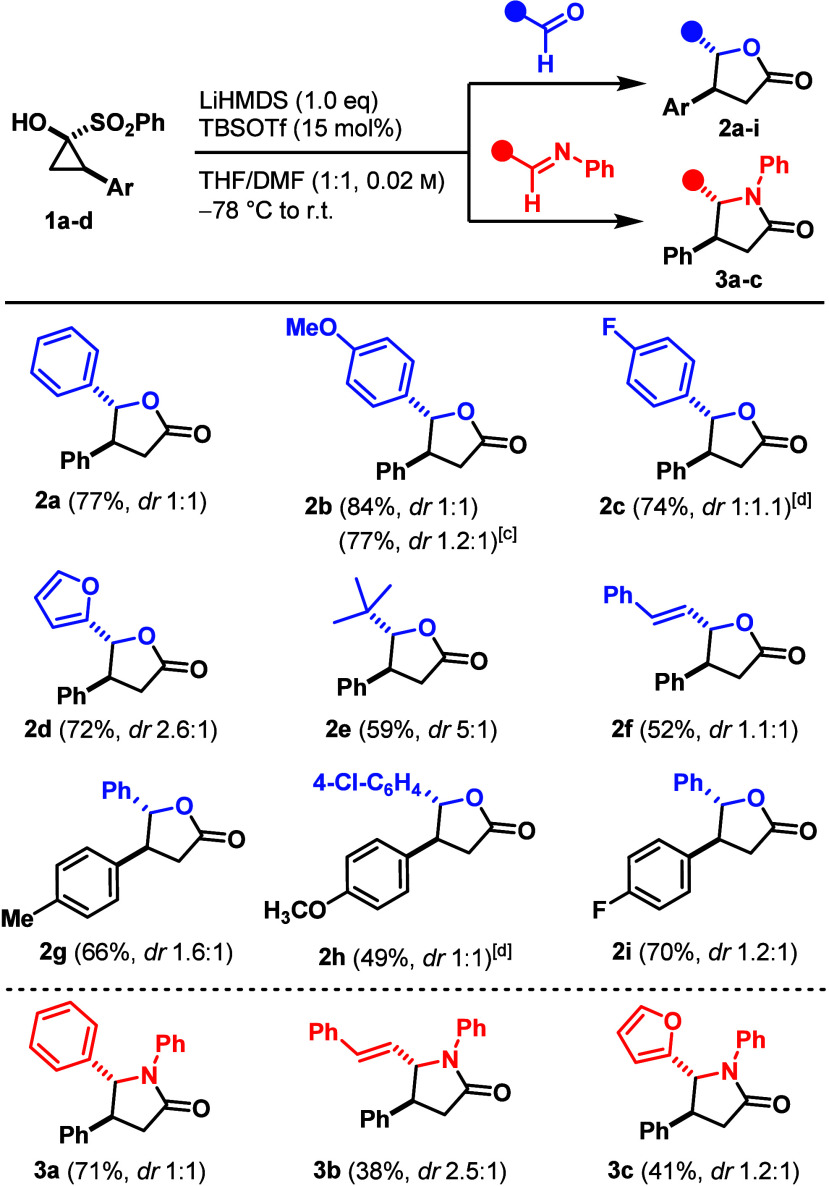
Aldehyde and Aldimine Substrate Scope^a^ ^,^ ^b^

a Amount of aldehyde/imine: 8.0 equiv; *dr* was determined by ^1^H NMR of the crude reaction mixture.
b Isolated yields on a 0.2 mmol scale if not stated otherwise.
c On a 1.0 mmol scale with respect to SCP.
d On a 0.15 mmol scale with respect to SCP.

After demonstrating that a diverse array of aldehydes and SCPs are amenable to transformation under these conditions, we next evaluated our methodology with aldimines. *N*-Benzylideneaniline was efficiently converted to lactam **3a** in 71% yield with a 1:1 diastereomeric ratio. In contrast, lactams **3b** and **3c** were only obtained in moderate yields of 38% (*dr* 2.5:1) and 41% (*dr* 1.2:1), respectively, from the corresponding aldimines (Scheme 2, bottom).

Next, we investigated the insertion of symmetric ketones into SCPs. Benzophenone reacted with **1a** under our standard conditions to afford γ-butyrolactone **4a** in 65% yield. *para*-Substituted SCPs **1b**–**d** bearing methyl, methoxy, or fluoro groups reacted with benzophenone to afford the products **4b–d** in 48%, 45%, and 55% yield, respectively. The dialkyl-substituted lactone **4e** was generated in moderate yield from 3-pentanone. 4,4’-Dimethyl benzophenone furnished lactone **4f** in 43% yield, while the trifluoromethyl analogue gave **4g** in 63% yield. Halogenated benzophenones yielded products **4h–4j** in 70%, 65% and 66%, respectively. These data indicate that electron-deficient ketones promote the transformation. Challenging spirocyclic lactone **4k** from 9-fluorenone was obtained, albeit in low yield, demonstrating the broad applicability of this protocol. Extension to heteroaryl ketones provided γ-butyrolactones **4l** (2-furyl, 52%), **4m** (2-thienyl, 57%), and **4n** (2-pyridyl, 40%) (Scheme 3).

**Scheme 3:**
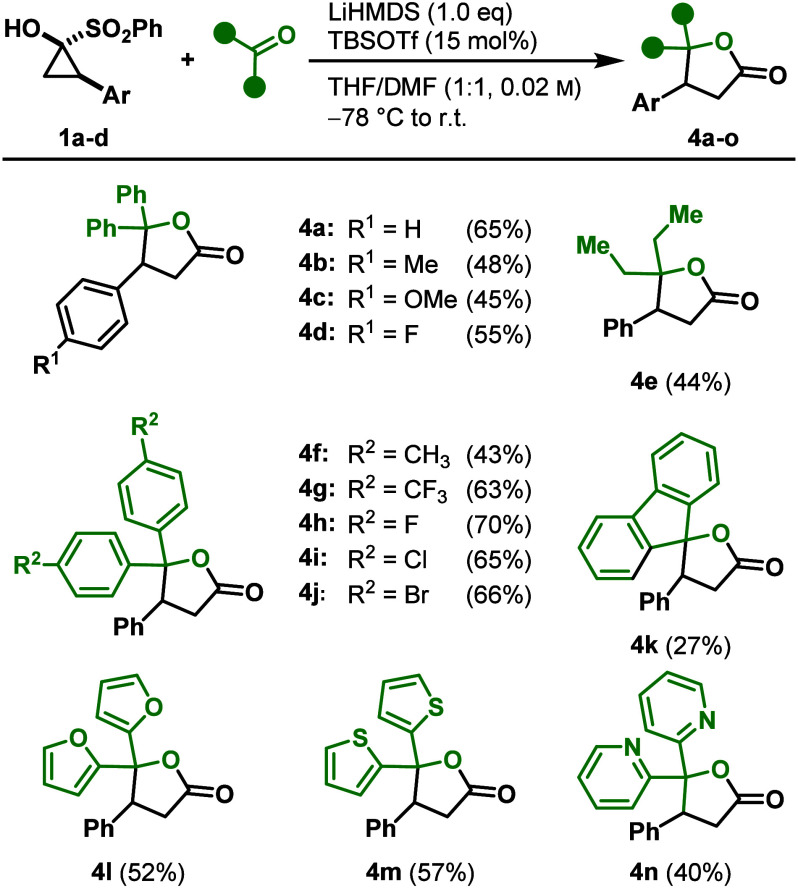
Ketone Substrate Scope^a^

a Isolated yields on a 0.2 mmol scale; amount of ketone: 8.0 equiv.

Despite the broad substrate scope, several limitations were identified. For example, 4,4′-dimethoxybenzophenone showed insufficient conversion to the desired lactone. Instead, the acylated SCP **5** (Scheme 4) cleanly formed as the sole product of the reaction, indicating the nucleophilic attack of an SCP moiety on the cyclopropanone, followed by ring opening. In addition, aliphatic 2-substituted SCPs proved entirely unreactive toward benzophenone or benzaldehyde under the standard conditions (see the Supporting Information, section *Ketone Substrate Scope*). These results suggest the involvement of an intermediate bearing a negative charge at the former 2-position of the cyclopropanone, which subsequently acts as a nucleophile toward the carbonyl carbon. With this carbonyl carbon being too electron-rich (e.g., if R^2^ = OMe) this attack becomes unfavored and therefore does not lead to product formation. Consistent with this hypothesis, the reaction of enantioenriched SCP **(** ***R*** **)-1a** with benzaldehyde gave a racemic product, as confirmed by chiral HPLC analysis. This indicates a racemization during the reaction and, hence, supports a mechanism via a ring-opened intermediate.

**Scheme 4:**
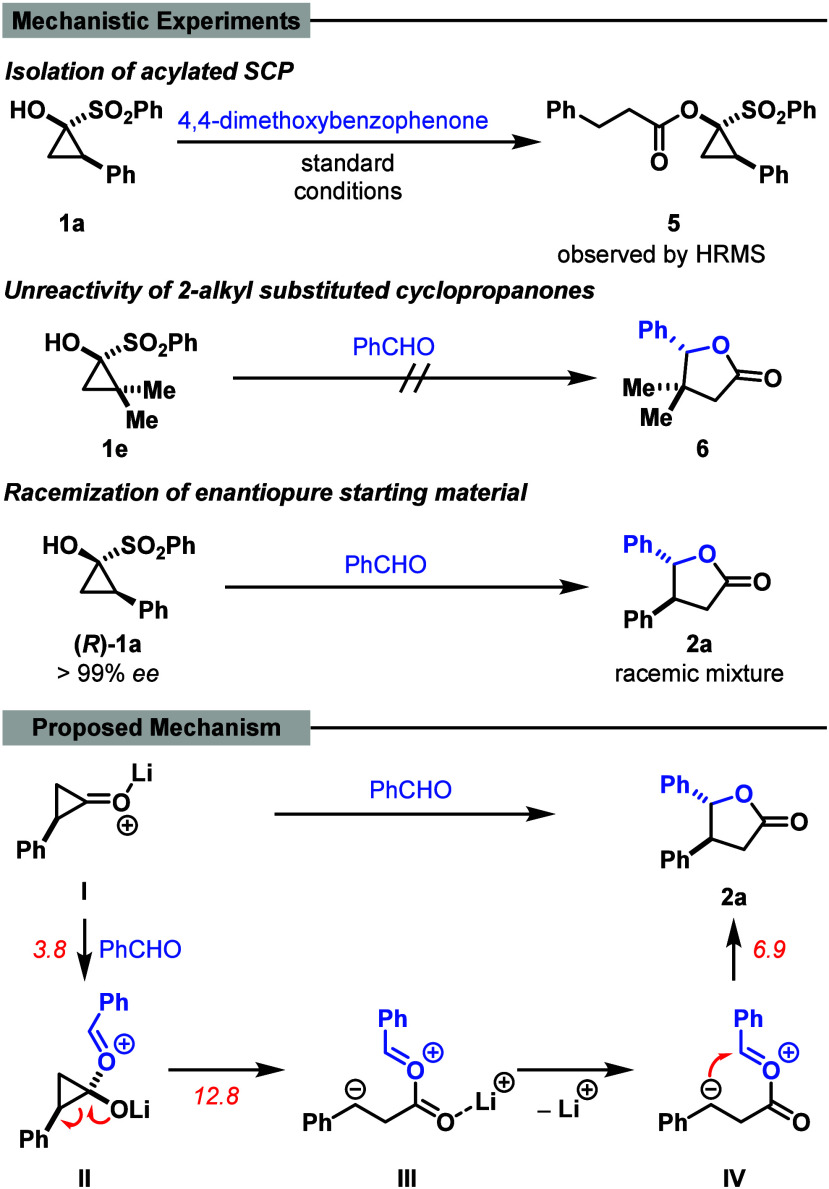
Mechanistical Investigation and Proposed Reaction Mechanism (Activation Barriers (in red) Are Given in kcal/mol; PW6B95/def2-TZVP-D4//r^2^scan-3c, CPCM­(THF))

Mechanistic insights were obtained from DFT studies aimed at elucidating the reaction pathway.^16,17^ Initial calculations were carried out in the absence of a Lewis acid. A viable pathway was identified in which nucleophilic attack of the carbonyl oxygen of benzaldehyde on the cyclopropanone is followed by ring opening to a linear zwitterionic intermediate that then cyclizes to the five-membered lactone. However, with a computed barrier of 20.3 kcal/mol for the rate-determining benzaldehyde attack, the transformation is predicted to be very slow at −78 °C. These data indicate that activation of the cyclopropanone is required to facilitate nucleophilic attack at the carbonyl center. Coordination of a TBS cation to the carbonyl oxygen affords a virtually barrierless aldehyde addition, but the resulting intermediate is calculated to be too stable to undergo further reaction.

In contrast, lithium coordination to the cyclopropanone permitted a viable pathway: the lithium-activated cyclopropanone **I** was attacked by the aldehyde, forming intermediate **II** with an activation barrier of 3.8 kcal/mol. Cleavage of the three-membered ring afforded zwitterionic intermediate **III**, identified as the rate-determining step with a calculated activation barrier of 12.8 kcal/mol. Dissociation of the lithium ion subsequently enabled direct cyclization of intermediate **IV** to the five-membered ring (Δ*G* ^*‡*^ = 6.9 kcal/mol), yielding **2a-** *trans*, or facilitated bond rotation of intermediate **IV** (Δ*G* ^*‡*^ = 6.5 kcal/mol), followed by ring-closure (Δ*G* ^*‡*^ = 5.2 kcal/mol) to form **2a-** *cis*. This mechanistic scenario accounts for the observed racemization of enantioenriched starting materials and the formation of a 1:1 mixture of *cis* and *trans* products (Scheme 4). Without prior dissociation of lithium before the ring-closing step, activation barriers for this process were calculated to be 17.3–20.0 kcal/mol, which is too high for this pathway to be considered likely under the reaction conditions. Notably, consideration of explicit solvent coordination to lithium was required for accurate modeling (further details are provided in the Supporting Information, in the section *DFT Studies*). Nevertheless, the addition of TBSOTf was found crucial to minimizing the formation of side products. Consistent with our computational results and the obersvation of the acylated SCP **5**, we conclude that the TBS cation stabilizes reactive intermediates that would otherwise undergo side reactions.

In conclusion, an efficient method has been developed for the insertion of carbonyl compounds (aldehydes, aldimines, and ketones) into cyclopropanones generated in situ from bench-stable SCPs. A broad range of functional groups on the carbonyl coupling partner is tolerated. Both electron-donating and electron-withdrawing substituents on the aryl ring of the SCP are compatible, whereas replacement of the aryl group with an alkyl substituent results in no reaction under the standard conditions. The lack of reactivity in the alkyl case, together with the formation of a racemic product from an enantiopure SCP, is consistent with the involvement of a ring-opened intermediate bearing a negative charge at the former 2-position of the cyclopropanone. DFT studies support this mechanistic proposal and highlight both the crucial role of lithium-ion activation and TBS cation stabilization to controlling the cyclopropanone reactivity.

## Supplementary Material



## Data Availability

The data underlying this study are available in the published article and its Supporting Information.

## Notes

Detailed experimental procedures and analytical data for all new compounds (PDF)

## References

[1] e Biju, A. , Banerjee, P. , Eds. Donor Acceptor Cyclopropanes in Organic Synthesis; Wiley: Weinheim, 2024.

[2] Nguyen T. V. T., Bossonnet A., Wodrich M. D., Waser J. (2023). Photocatalyzed
2σ + 2σ and 2σ + 2π Cycloadditions for the
Synthesis of Bicyclo3.1.1heptanes and 5- or 6-Membered Carbocycles. J. Am. Chem. Soc..

[3] Reissig H.-U., Hirsch E. (1980). Donor-Acceptor Substituted
Cyclopropanes:
Synthesis and Ring Opening to 1,4-Dicarbonyl Compounds. Angew. Chem., Int. Ed. Engl..

[4] Pandey A. K., Ghosh A., Banerjee P. (2016). Reactivity of Donor-Acceptor
Cyclopropanes with Saturated and Unsaturated Heterocyclic Compounds. Isr. J. Chem..

[5] Sabbatani J., Maulide N. (2016). Temporary Generation of a Cyclopropyl
Oxocarbenium Ion Enables Highly Diastereoselective Donor-Acceptor
Cyclopropane Cycloaddition. Angew. Chem., Int.
Ed..

[6] Mlostoń G., Kowalczyk M., Augustin A. U., Jones P. G., Werz D. B. (2021). Lewis-Acid-Catalyzed
(3 + 2)-Cycloadditions of Donor-Acceptor Cyclopropanes with Thioketenes. Eur. J. Org. Chem..

[7] b Stereochemistry I; Topics in Current Chemistry Fortschritte der Chemischen Forschung 47; Springer: Berlin Heidelberg, Berlin, Heidelberg, 1974.

[8] Wasserman H. H., Clagett D. C. (1964). Cyclopropanone derivatives from alkoxyvinyl
esters. The preparation of 1-substituted cyclopropanols. Tetrahedron Lett..

[9] An Y., Liu J., Jiang H.-Y., Wang Y., Chen Z. (2008). A convenient new method to construct 1-alkynyl cyclopropanol and
its synthetic application to prepare trisubstituted dienones. Tetrahedron Lett..

[10] Poteat C. M., Jang Y., Jung M., Johnson J. D., Williams R. G., Lindsay V. N. G. (2020). Enantioselective
Synthesis of Cyclopropanone Equivalents and Application to the Formation
of Chiral β-Lactams. Angew. Chem., Int.
Ed..

[11] Carson C. A., Kerr M. A. (2005). Diastereoselective
synthesis of pyrrolidines via the Yb­(OTf)­3 catalyzed three-component
reaction of aldehydes, amines, and 1,1-cyclopropanediesters. J. Org. Chem..

[12] Schneider T. F., Kaschel J., Dittrich B., Werz D. B. (2009). anti-Oligoannelated
THF moieties: synthesis via push-pull-substituted
cyclopropanes. Org. Lett..

[13] Burke E. D., Lim N. K., Gleason J. L. (2003). Catalytic Enantioselective Homoaldol
Reactions Using Binol Titanium­(IV) Fluoride Catalysts. Synlett.

[14] Bach R. D., Dmitrenko O. (2002). The effect
of substitutents on the strain energies of small ring compounds. J. Org. Chem..

[15] Muir J. E., Sulc B. M., Tran D. T., Poteat C. M., MacMillan A. K., Lindsay V. N. G. (2025). Stereospecific
Synthesis of Cyclobutanones and Spirohexanones via Formal 3 + 1 Cycloaddition
of Cyclopropanones with Sulfur Ylides. Org.
Lett..

[16] f Neese, F. Software Update: The ORCA Program System—Version 6.0. WIREs Comput. Mol. Sci. 2025 15,10.1002/wcms.70019

[17] Walker M., Harvey A. J. A., Sen A., Dessent C. E. H. (2013). Performance of
M06, M06–2X, and M06-HF density functionals for conformationally
flexible anionic clusters: M06 functionals perform better than B3LYP
for a model system with dispersion and ionic hydrogen-bonding interactions. J. Phys. Chem. A.

