# Supplementary material for: Ring-Enlargement of in Situ Generated Cyclopropanones with Carbonyls and Imines: Synthesis of γ‑Butyrolactones and -lactams
Source: Org Lett. 2026 Apr 1;28(15):4783–8. doi: 10.1021/acs.orglett.6c00839 (PMC13097245; doi:10.1021/acs.orglett.6c00839)
Supplement: Supplementary file 1 [file ol6c00839_si_001.pdf]

Supporting Information

Ring-Enlargement of In Situ Generated Cyclopropanones with Carbonyls and Imines: Synthesis of γ-Butyrolactones and lactams

Marvin Lange, Arthur Semmelmaier, Richard Herzog,

Heinrich F. von Köller, and Daniel B. Werz*

Albert-Ludwigs-Universität Freiburg

Institute of Organic Chemistry

Alberstraße 21, 79104 Freiburg, Germany

(*corresponding author: daniel.werz@chemie.uni-freiburg.de)

Table of Contents

General Information S2
General Procedure S4
Aldehyde Substrate Scope S4
Lactam Substrate Scope S48
Ketone Substrate Scope S58
DFT Studies S95
Synthesis of Starting Materials S118
Optimization of Reaction Conditions S135
References S137

General Information

Synthesis and purification:

Air and/or moisture sensitive **reactions** were carried out in flame-dried (using a heat gun at 600 °C) Schlenk flasks under a positive pressure of argon equipped either with a rubber septum or sealed with a glass stopper (using PTFE joint sleeve) and a clamp. **Anhydrous solvents** were either taken from a solvent purification system (CH_2Cl_2, Et_2O, THF, MeCN, toluene) and stored in flame-dried flask over activated molecular sieves (4 Å) under an argon atmosphere or were purchased (Thermo Scientific) anhydrous over molecular sieves (MeOH, EtOH, acetone, DMF, $CHCl_3$, $(CH_2Cl)_2$, EtOAc, pyridine, 1,4-dioxane).

Reaction temperatures refer to the respective cooling bath/ heating bath temperature which was either achieved by using an immersion cooler (Julabo FT902), an appropriate cooling bath or a silicon oil bath:

- ambient temperature: 18 °C to 25 °C,
- 0 °C: ice/water mixture,
- −40 C: MeCN saturated with dry ice,
- −78 C: acetone saturated with dry ice.

If necessary, starting materials were **purified** prior to use by one of the following methods:

- if liquid: distilled in vacuo (50 mbar),
- if solid: recrystallized from *i*-PrOH.

Thin-layer chromatography was carried out on SIL G-25 UV254 glass plates from Macherery-Nagel. Detection was achieved by UV-fluorescence quenching at 254 nm and/or by visualization using one of the following staining reagent followed by gently heating using a heat gun:

- **Ceric ammonium molbydate (CAM) stain**: dissolve 0.5 g of ceric ammonium sulfate dihydrate ($Ce(NH_4)_4(SO_4)_4 \cdot 2H_2O$) and 12 g of ammonium molybdate tetrahydrate $(NH_4)_6Mo_7O_{24} \cdot 4H_2O$ in 235 mL H_2O and add 15 mL conc. H_2SO_4 dropwise with stirring.
- **$KMnO_4$ stain**: dissolve 5 g $KMnO_4$ and 13.5 g K_2CO_3 in 200 mL H_2O and add 5 mL aq. NaOH solution (1 M).

Flash column chromatography was carried out using Silica 60 (0.04–0.063 mm, Macherey-Nagel). Solvents used for workup and chromatography are of technical grade and were distilled prior to use.

Analytics:

NMR spectroscopy was carried out on a 500 MHz Bruker DRX 500 or 400 MHz Bruker Advance II 400 Instrument. ^{1}H- and ^{13}C-NMR shifts are given relative to the residual undeuterated solvent signal ($CDCl_3$: δ_H = 7.26 ppm (s), δ_C = 77.2 ppm; CD_2Cl_2: δ_H = 5.32 ppm (t), δ_C = 53.8 ppm; acetone-d_6: δ_H = 2.05 ppm (quin), δ_C = 29.8 ppm; DMSO-d_6: δ_H = 2.50 ppm (quin), δ_C = 39.5 pp). Signals are described as following: "s" = singlet, "d" = doublet, "t" = triplet, "q" = quartet, "quin" = quintet and combinations of the latter. Multiplets are labeled as "m". Broad signals are additionally labeled with "br". Signal of higher order that nevertheless appear as first order signals are labeled "app" (apparent) and coupling constants are given in quotation marks "*J*". Assignment of signals was assisted by 2D-NMR experiments (^{1}H,^{13}C-HSQC, ^{1}H,^{13}C-HMBC, ^{1}H,^{1}H-COSY, ^{1}H,^{1}H-NOESY).

High-resolution mass spectrometry was carried out either on a Thermo Exactive mass spectrometer (ESI and APCI measurements with Orbitrap analyzer) or an Agilent 7890B GC System coupled to a 6545 LC/Q-TOF mass spectrometer (APCI measurements with Q-TOF analyzer).

ATR-FTIR spectroscopy was carried out on a Spectrum Two Spectrometer from Perkin Elmer. Samples were measured neat on a diamond ATR crystal. Transmission-bands are given in cm^{-1}.

GC/FID or **GC/MS** measurements were carried out on an Agilent 7820A Gas Chromatograph coupled to an FID or 5977E MSD.

General Procedure

A flame-dried (2 x) Schlenk flask was charged with SCP (0.20 mmol, 1.00 eq.) and the respective carbonyl compound (*if solid, otherwise added after dissolution of the SCP*, 1.60 mmol, 8.00 eq.). The flask was evacuated and backfilled with argon (2 x) before anhydrous THF (5 mL) and anhydrous DMF (5 mL) were added, respectively. The homogenous solution was cooled to −78 °C. At this temperature, TBSOTf (7.9 mg, 0.03 mmol, 7 µL,0.15 eq.) was added followed by dropwise addition of LiHMDS (1 M in THF, 0.20 mmol, 0.20 mL, 1.00 eq.) within 1 min. The reaction mixture was allowed to slowly warm to room temperature and stirred overnight. The reaction was then stopped by adding aq. NaCl solution (10% *w*/*v*, 25 mL) and the aq. phase was extracted with M*t*BE (3 x 10 mL). The combined org. phases were dried by stirring over anhydrous $MgSO_4$, filtered through a plug of cotton wool, silica gel or celite was added and the solvent was removed under vacuum. Purification by flash column chromatography (SiO_2) afforded the respective insertion products.

Aldehyde Substrate Scope

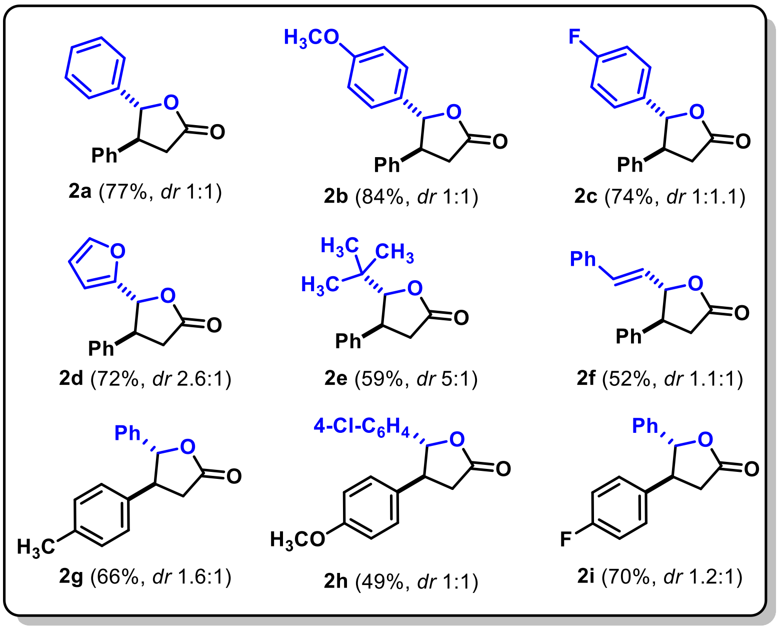

Lactone 2a

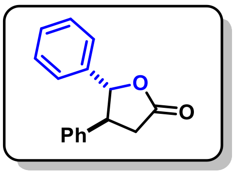

Prepared according to the **General Procedure** from SCP **1a** (54.9 mg, 0.20 mmol, 1.00 eq.) and benzaldehyde (170 mg, 1.60 mmol, 163 µL, 8.00 eq.). Purification by flash column chromatography (SiO_2, *n*-pentane/EtOAc 20:1 to 2:1) afforded the lactones ***anti*-2a** (18.6 mg, 0.08 mmol, 39%) and ***syn*-2a** (17.9 mg, 0.08 mmol, 38%) as colorless solids (overall yield: 77%, *dr* 1:1).

FTIR (ATR): $\tilde{\nu}$ [cm^{-1}] = 1778, 1455, 1269, 1195, 1142, 1030, 994, 879, 760, 698.

^{1}H-NMR (500 MHz, CD_2Cl_2): δ_H (ppm) = 7.38–7.29 (m, 6H), 7.23–7.17 (m, 4H), 5.44 (d, *J* = 8.5 Hz, 1H), 3.61 (dt, *J* = 10.7, 8.5 Hz, 1H), 3.07 (dd, *J* = 17.6, 8.5 Hz, 1H), 2.93 (dd, *J* = 17.6, 10.7 Hz, 1H).

^{13}C-NMR (126 MHz, CD_2Cl_2): δ_C (ppm) = 175.4 (qC), 138.0 (qC), 137.8 (qC), 129.2 (CH), 128.7 (CH), 128.0 (CH), 127.5 (CH), 125.7 (CH), 87.5 (CH), 50.6 (CH), 37.2 (CH_2).

HR-MS (ESI+, Orbitrap): calc. for $C_{16}H_{15}O_2$ [M+H]$^+$: 239.1067, found: 239.1070.

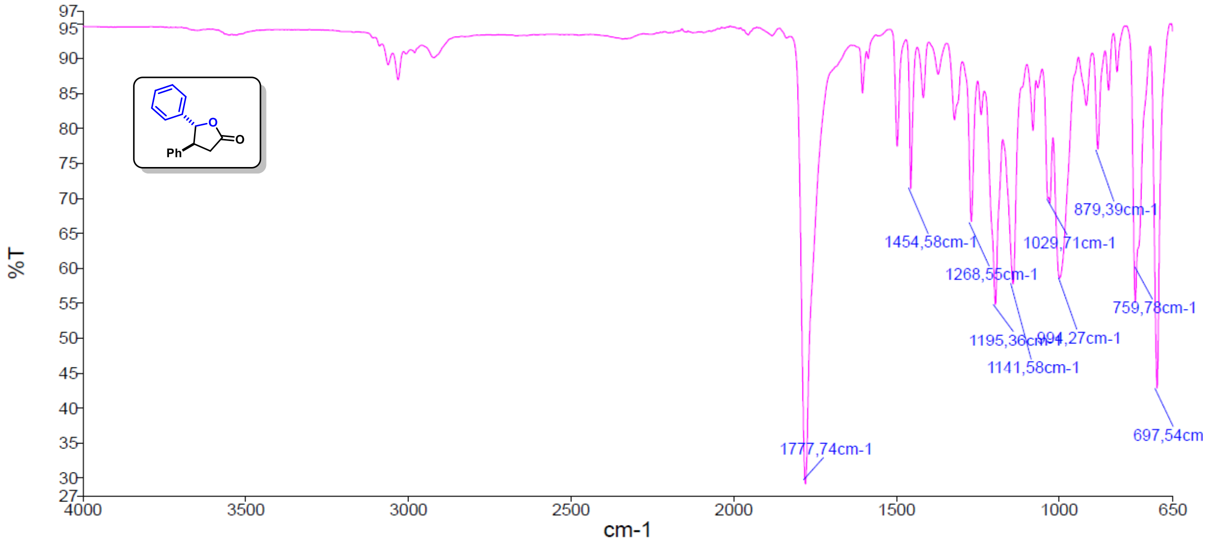

FT-IR Spectrum (ATR, thin film) of ***anti*-2a**.

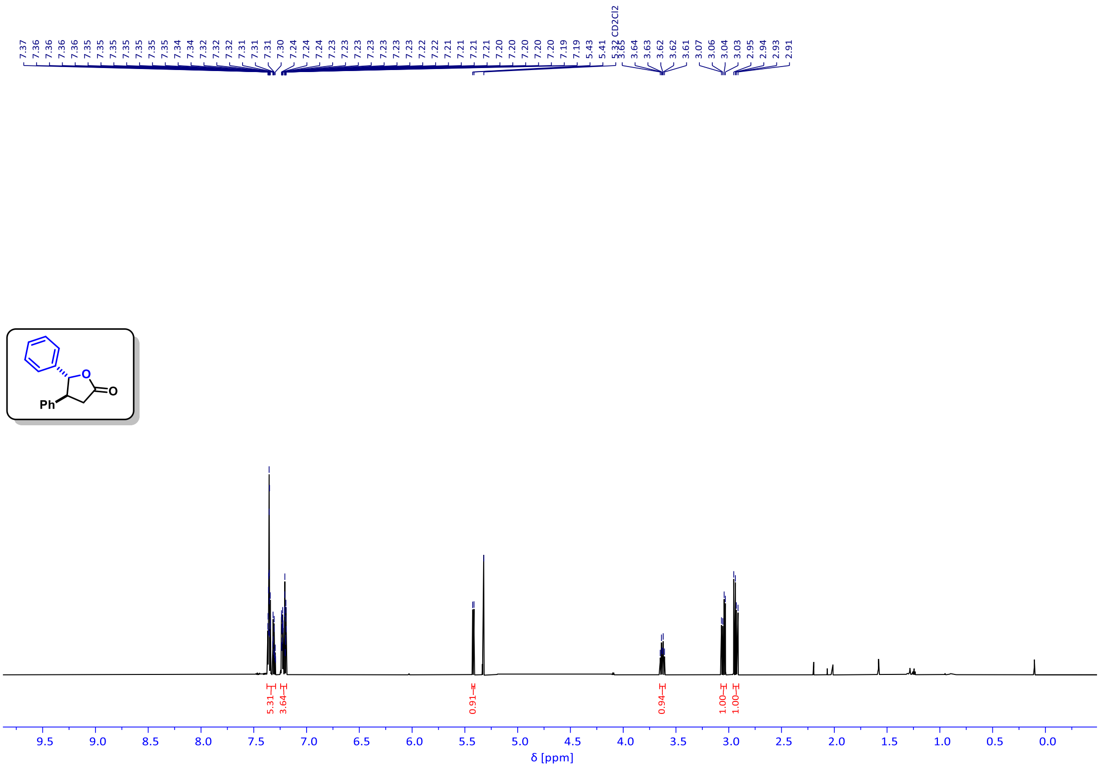

^{1}H-NMR spectrum (700 MHz, CD_2Cl_2) of ***anti*-2a**.

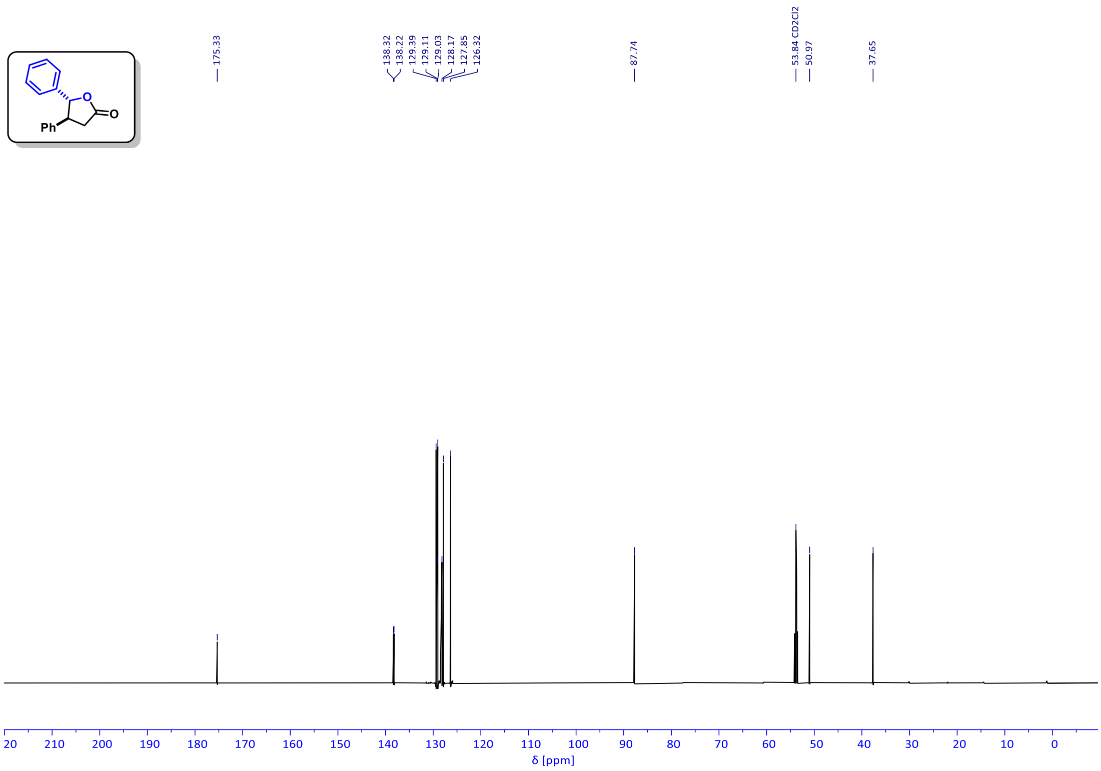

^{13}C-NMR spectrum (176 MHz, CD_2Cl_2) of ***anti*-2a**.

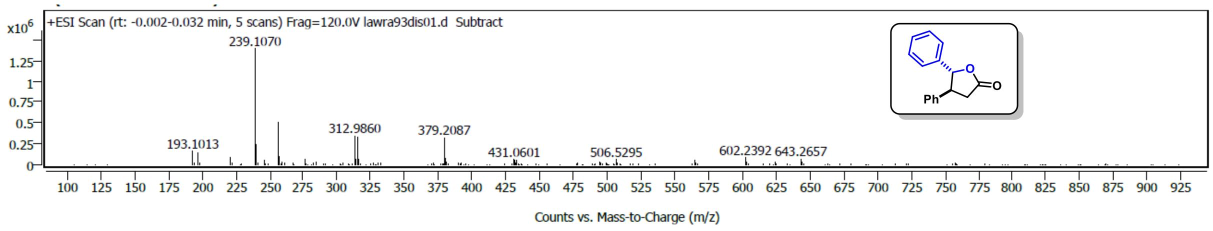

HR-MS Spectrum (ESI+) of ***anti*-2a**.

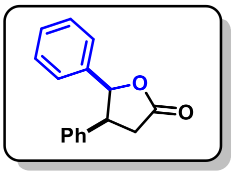

FTIR (ATR): $\tilde{\nu}$ [cm^{-1}] = 1778, 1454, 1173, 1143, 1029, 1013, 978, 757, 723, 699.

^{1}H-NMR (700 MHz, $CDCl_3$): δ_H (ppm) = 7.17–7.06 (m, 6H), 6.94–6.87 (m, 2H), 6.86–6.78 (m, 2H), 5.83 (d, J = 6.7 Hz, 1H), 4.06 (dt, J = 8.3, 6.7 Hz, 1H), 3.05 (dd, J = 17.5, 8.3 Hz, 1H), 2.95 (dd, J = 17.5, 6.7 Hz, 1H).

^{13}C-NMR (176 MHz, $CDCl_3$): δ_C (ppm) = 176.8, 136.8, 135.7, 128.4, 128.1, 128.1, 128.0, 127.5, 125.8, 84.8, 47.1, 35.1.

HR-MS (GC-APCI, +, Q-TOF): calc. for $C_{16}H_{15}O_2$ [M+H]$^+$: 239.1067, found: 239.1066.

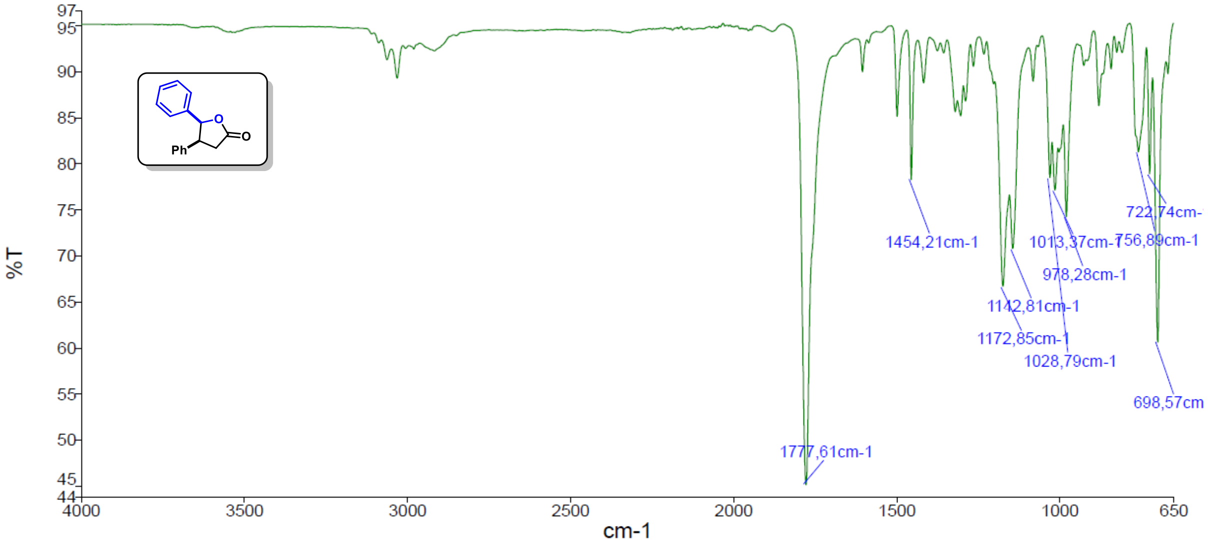

FT-IR Spectrum (ATR, thin film) of ***syn*-2a**.

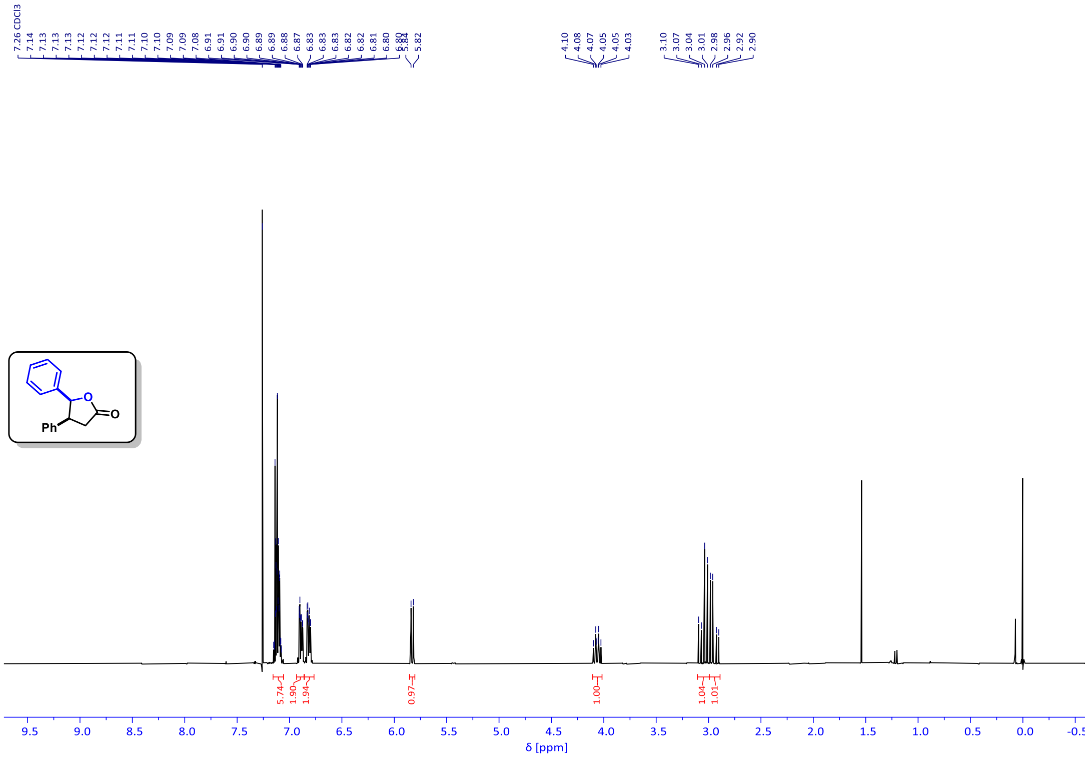

^{1}H-NMR spectrum (300 MHz, $CDCl_3$) of ***syn*-2a**.

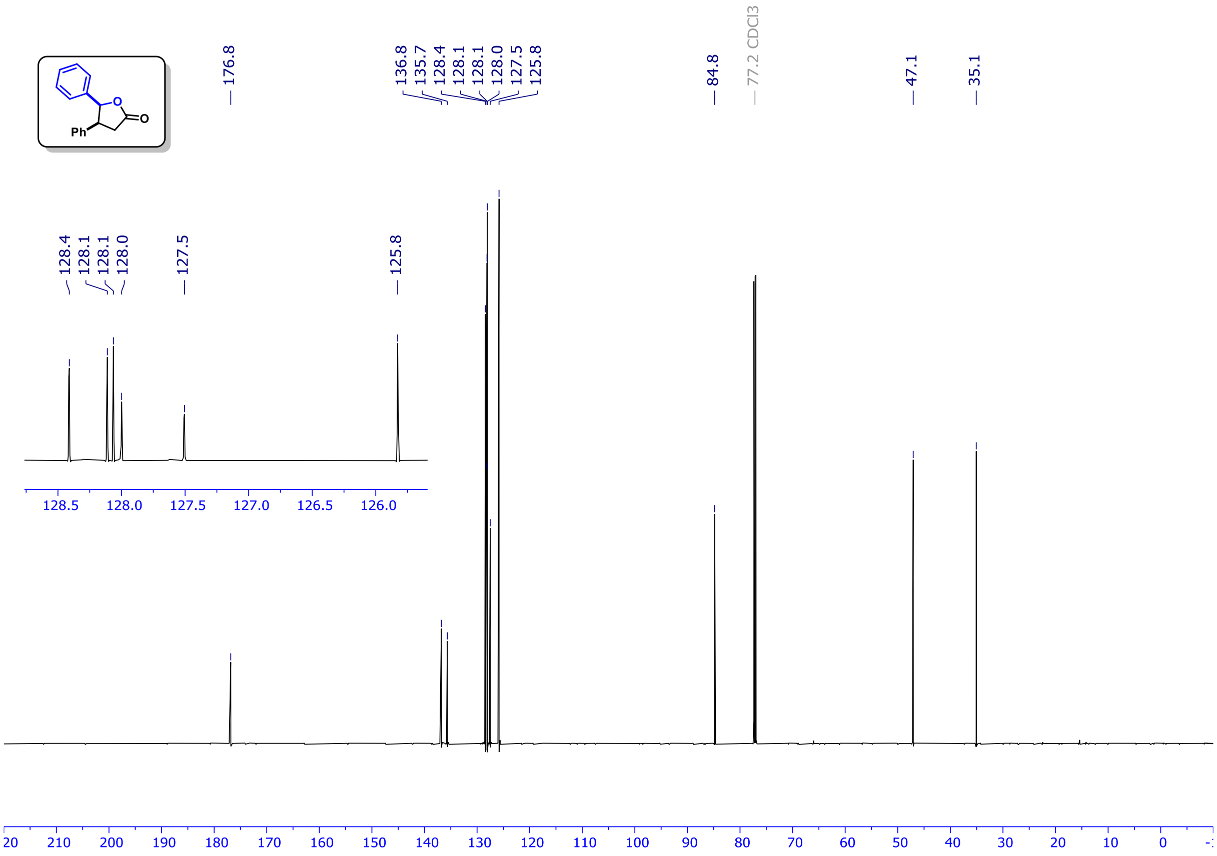

^{13}C-NMR spectrum (176 MHz, $CDCl_3$) of ***syn*-2a**.

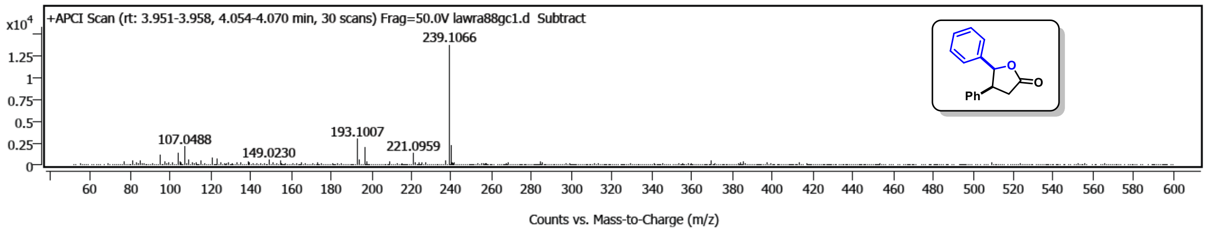

HR-MS Spectrum (APCI,+) of ***syn*-2a**.

Lactone 2b

Prepared according to the **General Procedure** from SCP **1a** (54.9 mg, 0.20 mmol, 1.00 eq.) and anisaldehyde (218 mg, 1.60 mmol, 195 µL, 8.00 eq.). Purification by flash column chromatography (SiO_2, *n*-pentane/EtOAc 8:1 to 6:1) afforded the lactones ***anti*-2b** (22.4 mg, 0.08 mmol, 42%) and ***syn*-2b** (22.6 mg, 0.08 mmol, 42%) as colorless solids (overall yield: 84%, *dr* 1:1).

On a 1 mmol scale

A flame-dried (2 x) Schlenk flask was charged with SCP **1a** (274 mg, 1.00 mmol, 1.00 eq.) and the flask was evacuated and backfilled with argon (2 x) before anhydrous THF (25 mL) and anhydrous DMF (25 mL) were added. Afterwards, freshly distilled (10 mbar) anisaldehyde (1.09 g, 8.00 mmol, 0.97 mL, 8.00 eq.) was added and the homogenous

solution was cooled to −78 °C. At this temperature, TBSOTf (39.5 mg, 0.15 mmol, 0.15 eq.) was added followed by dropwise addition of LiHMDS (1 M in THF, 1.00 mmol, 1.00 mL, 1.00 eq.) within 2 min. The reaction mixture was allowed to slowly warm to room temperature and stirred for 17.5 h. The reaction was then stopped by adding aq. NaCl solution (10% *w*/*v*, 150 mL) and the aq. phase was extracted with M*t*BE (3 x 50 mL). The combined org. phases were dried by stirring over anhydrous $MgSO_4$, filtered through a plug of cotton wool and the solvent was removed under vacuum. Purification by flash column chromatography (SiO_2, *n*-pentane/acetone 15:1 to 10:1) afforded the lactone **2b** (206 mg, 0.77 mmol, 77%) as a mixture of both diastereomers (*dr* 1.2:1) as a pale-yellow oil.

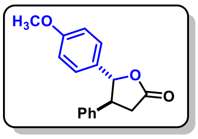

FTIR (ATR): $\tilde{\nu}$ [cm^{-1}] = 1776, 1515, 1248, 1195, 1176, 1143, 1030, 985, 815, 699.

^{1}H-NMR (700 MHz, $CDCl_3$): δ_H (ppm) = 7.36–7.31 (m, 2H), 7.31–7.27 (m, 1H), 7.18–7.10 (m, 4H), 6.87–6.85 (m, 2H), 5.37 (d, *J* = 8.6 Hz, 1H), 3.80 (s, 3H), 3.61 (dt, *J* = 11.0, 8.6 Hz, 1H), 3.06 (dd, *J* = 17.6, 8.6 Hz, 1H), 2.93 (dd, *J* = 17.6, 11.0 Hz, 1H).

^{13}C-NMR (176 MHz, $CDCl_3$): δ_C (ppm) = 175.4 (qC), 160.1 (qC), 138.0 (qC), 129.7 (qC), 129.2 (CH), 128.0 (CH), 127.5 (CH), 127.5 (CH), 114.2 (CH), 87.6 (CH), 55.4 (CH_3), 50.6 (CH), 37.3 (CH_2).

HR-MS (ESI+, Orbitrap): calc. for $C_{17}H_{17}O_3$ $[M+H]^+$: 269.1172, found: 269.1178.

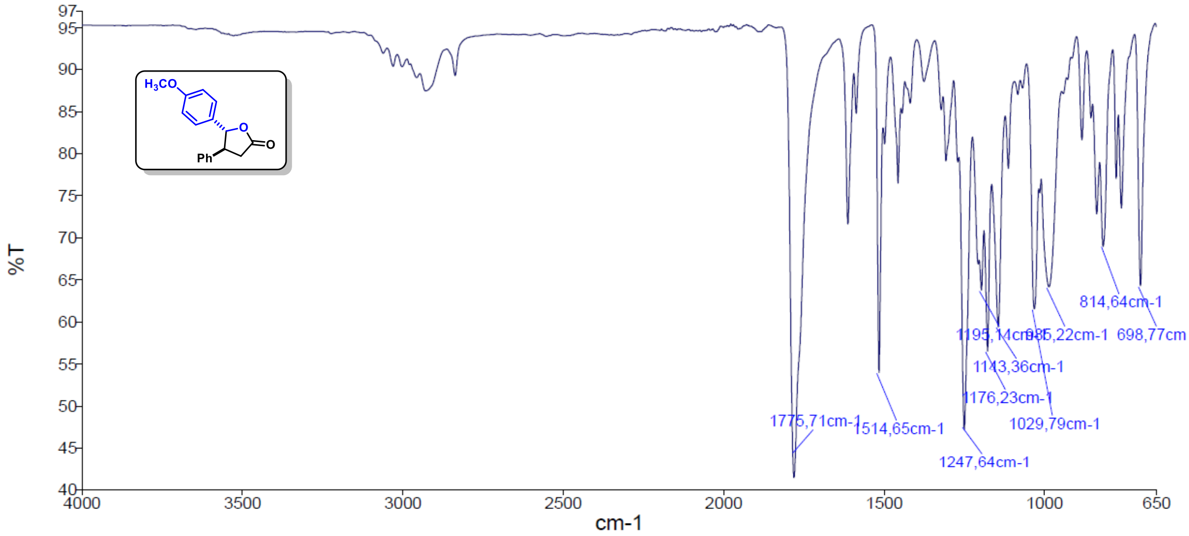

FT-IR Spectrum (ATR, thin film) of ***anti*-2b**.

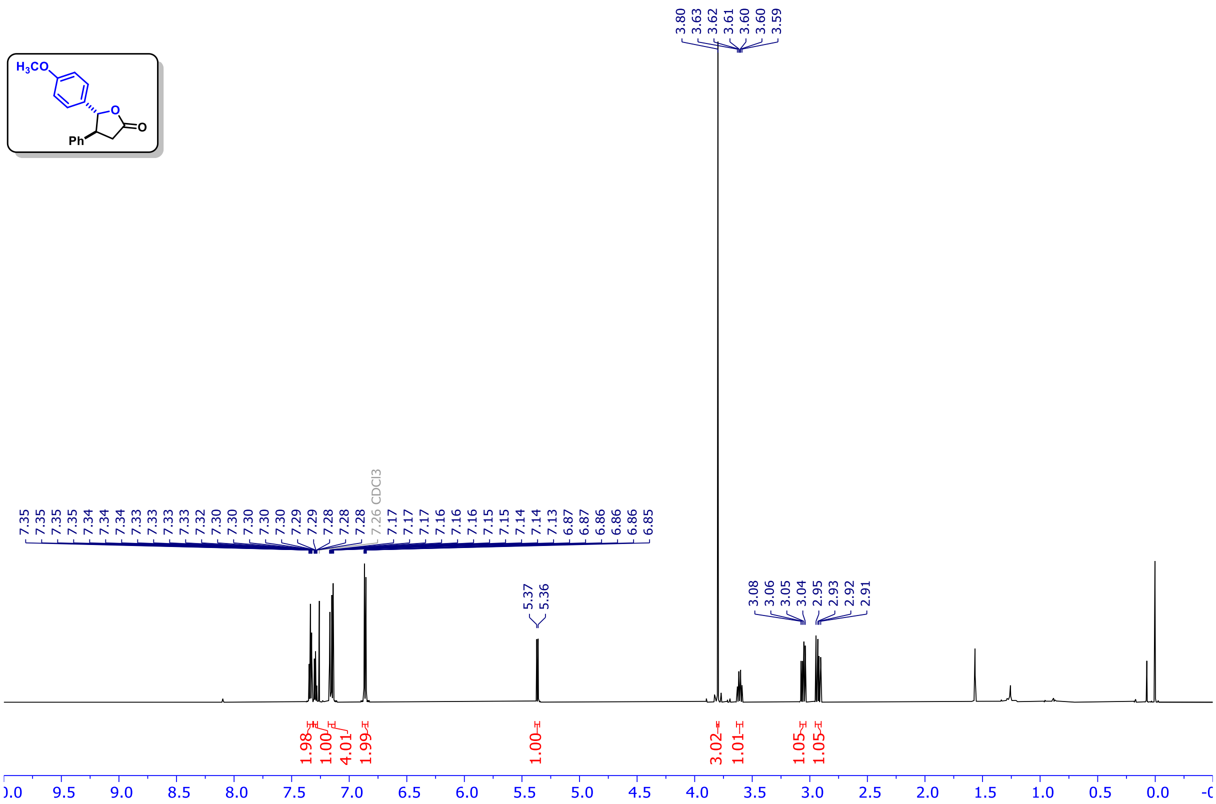

^{1}H-NMR spectrum (700 MHz, $CDCl_3$) of ***anti*-2b**.

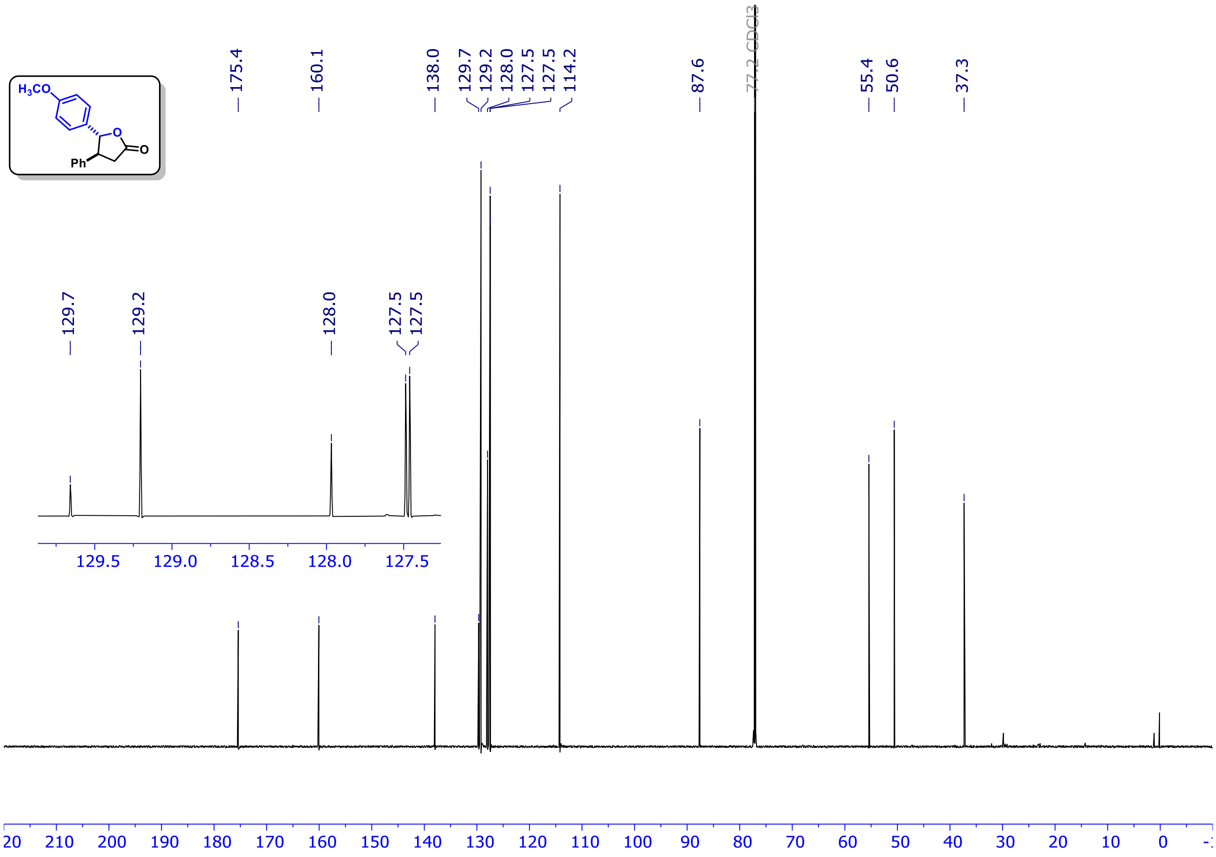

^{13}C-NMR spectrum (176 MHz, $CDCl_3$) of ***anti*-2b**.

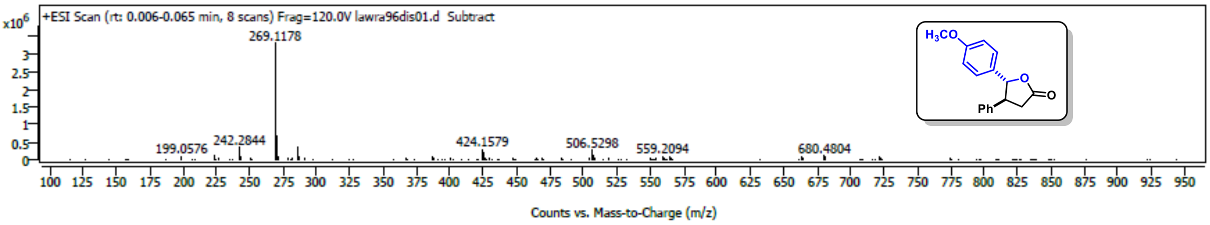

HR-MS Spectrum (ESI+) of ***anti*-2b**.

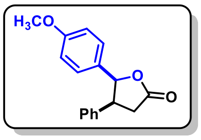

FTIR (ATR): $\tilde{\nu}$ [cm^{-1}] = 1775, 1612, 1514, 1302, 1249, 1171, 1143, 1029, 978, 700.

^{1}H-NMR (500 MHz, $CDCl_3$): δ_H (ppm) = 7.16–7.09 (m, 3H), 6.87–6.81 (m, 2H), 6.81–6.74 (m, 2H), 6.69–6.62 (m, 2H), 5.78 (d, J = 6.7 Hz, 1H), 4.02 (dt, J = 8.1, 6.7 Hz, 1H), 3.71 (s, 3H), 3.02 (dd, J = 17.4, 8.1 Hz, 1H), 2.94 (dd, J = 17.4, 6.7 Hz, 1H).

^{13}C-NMR (126 MHz, $CDCl_3$): δ_C (ppm) = 176.9 (qC), 159.3 (qC), 136.8 (qC), 128.4 (CH), 128.2 (CH), 127.6 (CH), 127.5 (CH), 127.2 (CH), 113.5 (CH), 84.8 (CH), 55.3 (CH_3), 47.1 (CH), 35.0 (CH_2).

HR-MS (ESI+, Orbitrap): calc. for $C_{17}H_{17}O_3$ $[M+H]^+$: 269.1172, found: 269.1178.

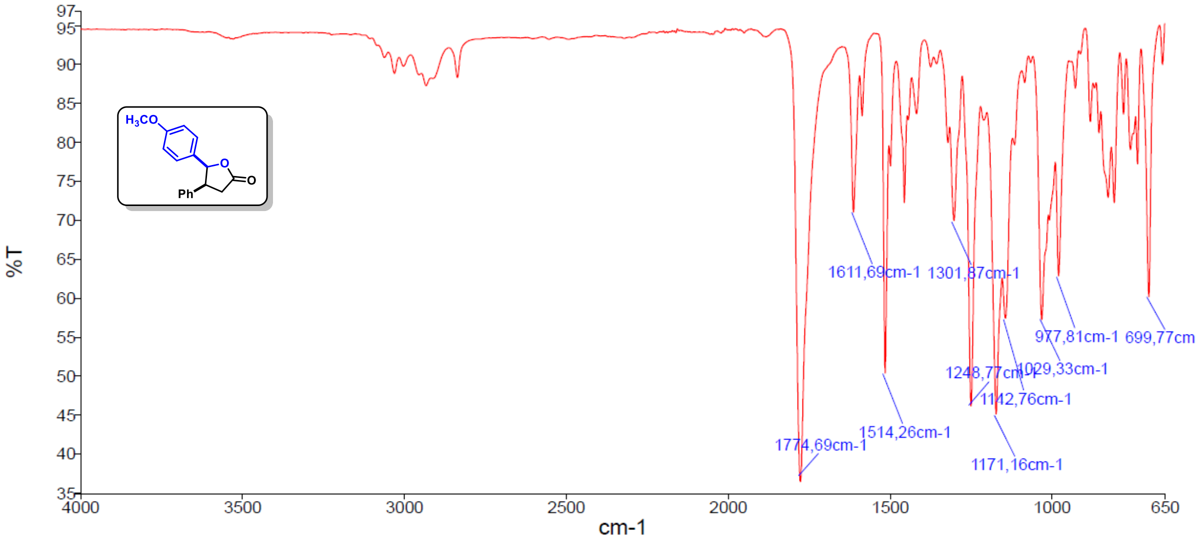

FT-IR Spectrum (ATR, thin film) of ***syn*-2b**.

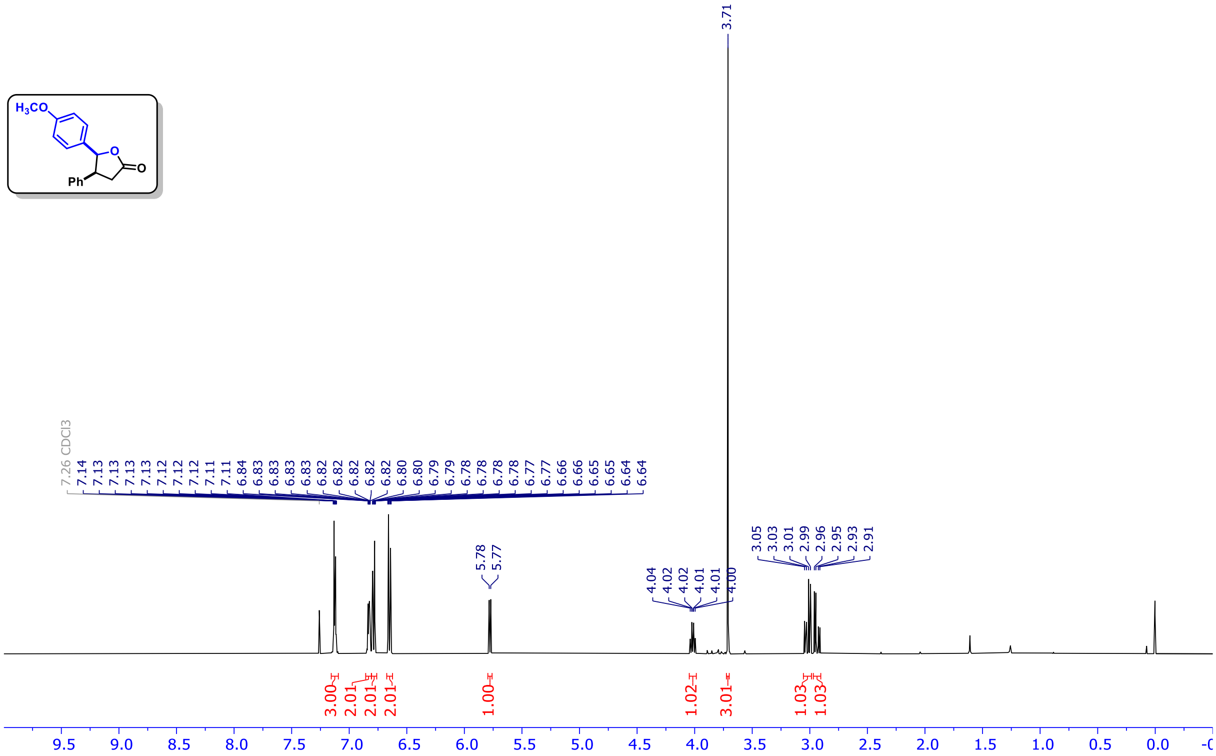

^{1}H-NMR spectrum (500 MHz, $CDCl_3$) of ***syn*-2b**.

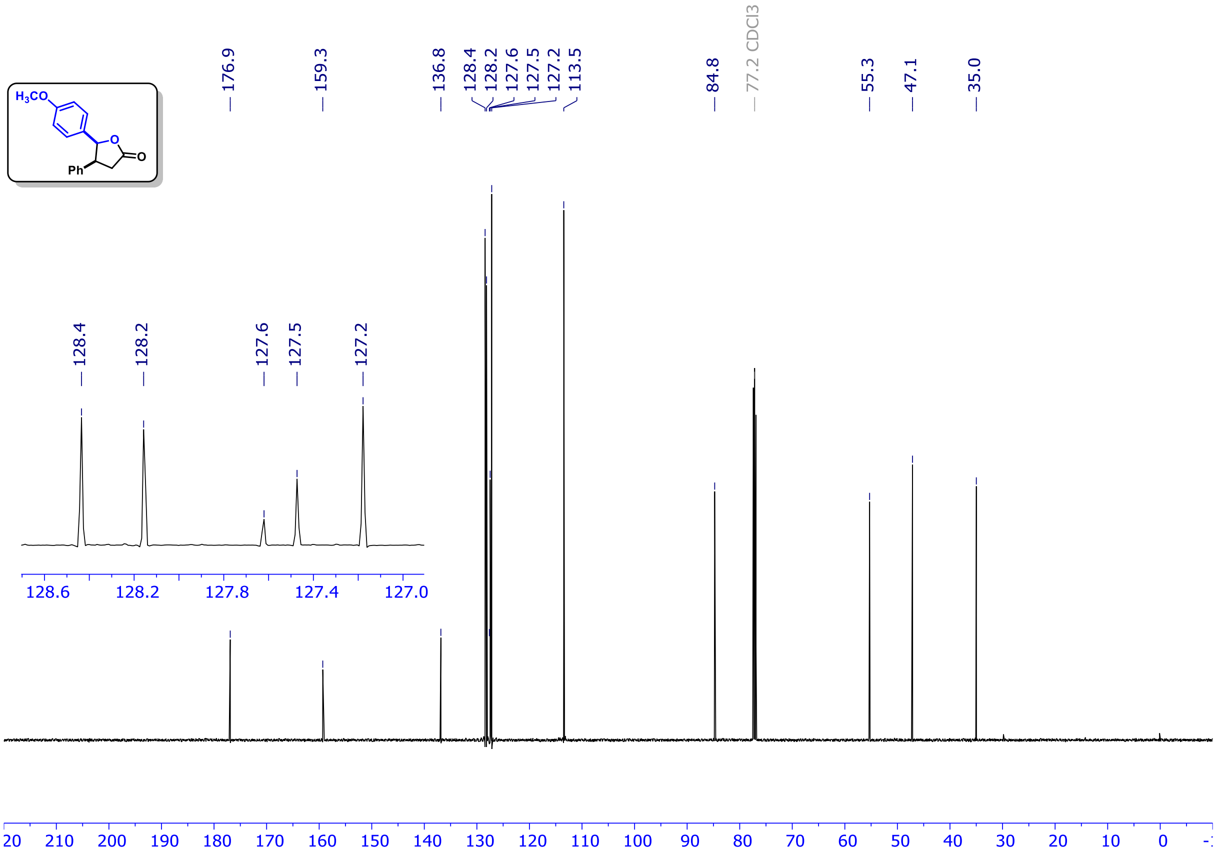

^{13}C-NMR spectrum (126 MHz, $CDCl_3$) of ***syn*-2b**.

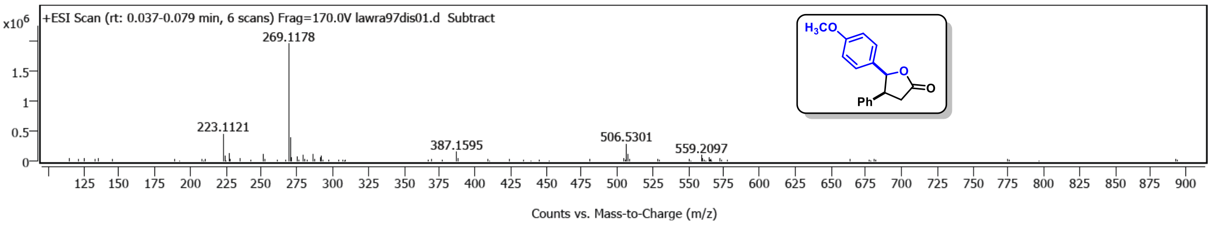

HR-MS Spectrum (ESI+) of ***syn*-2b**.

Lactone 2c

Prepared according to the **General Procedure** from SCP **1a** (41.1 mg, 0.15 mmol, 1.00 eq.), 4-fluorobenzaldehyde (149 mg, 1.20 mmol, 129 µL, 8.00 eq.), LiHMDS (1 M in THF, 0.15 mmol, 0.15 mL, 1.00 eq.) and TBSOTf (0.05 M in THF, 6.0 mg, 0.02 mmol, 0.45 mL, 0.15 eq.) in THF (3.3 mL) and DMF (3.75 mL). Purification by flash column chromatography (SiO_2, *n*-pentane/acetone 20:1) afforded the lactones ***anti*-2c** (13.5 mg, 0.05 mmol, 35%) and ***syn*-2c** (15.0 mg, 0.06 mmol, 39%) as colorless solids (overall yield: 74%, *dr* 1:1:1).

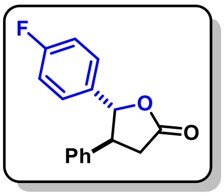

FTIR (ATR): $\tilde{\nu}$ [cm^{-1}] = 1786, 1606, 1513, 1230, 1148, 998, 819, 699.

^{1}H-NMR (700 MHz, $CDCl_3$): δ_H (ppm) = 7.38–7.34 (m, 2H), 7.33–7.30 (m, 1H), 7.20–7.14 (m, 4H), 7.05–7.00 (m, 2H), 5.39 (d, *J* = 8.7 Hz, 1H), 3.55 (dt, *J* = 11.2, 8.7 Hz, 1H), 3.06 (dd, *J* = 17.6, 8.7 Hz, 1H), 2.94 (dd, *J* = 17.6, 11.2 Hz, 1H).

^{13}C-NMR (176 MHz, $CDCl_3$): δ_C (ppm) = 175.1, 163.0 (d, *J* = 248.0 Hz), 137.6, 133.6 (d, *J* = 3.1 Hz), 129.3, 128.2, 127.7 (d, *J* = 8.4 Hz), 127.5, 115.8 (d, *J* = 22.1 Hz), 87.0, 51.0, 37.3.

^{19}F-NMR (659 MHz, $CDCl_3$): δ_F (ppm) = −112.9 (m).

HR-MS (GC-APCI, +, Q-TOF): calc. for $C_{16}H_{14}FO_2$ $[M+H]^+$: 257.0972, found: 257.0968.

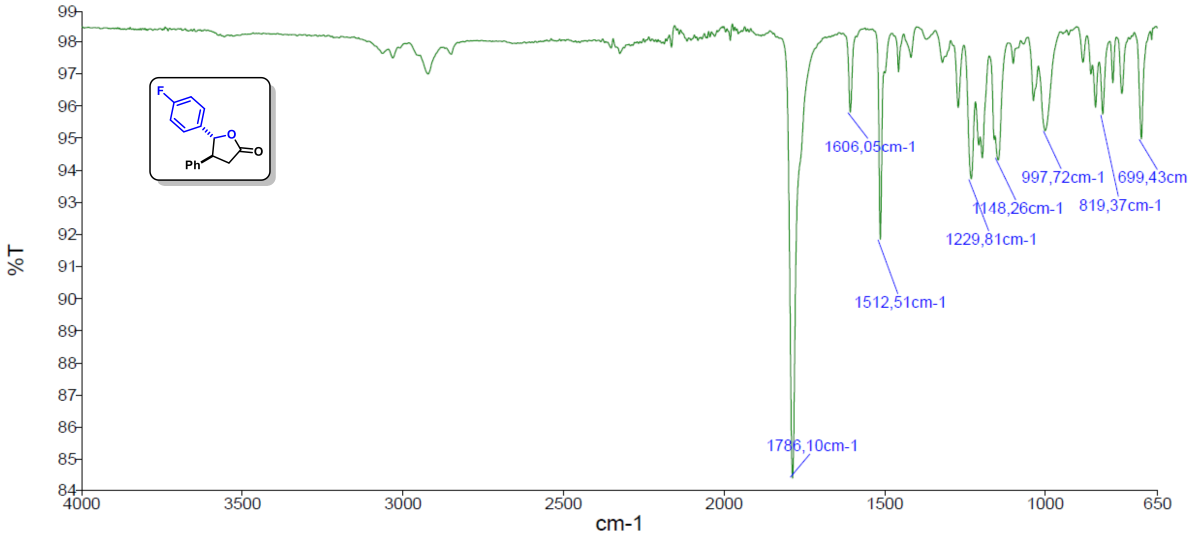

FT-IR Spectrum (ATR, thin film) of ***anti*-2c**.

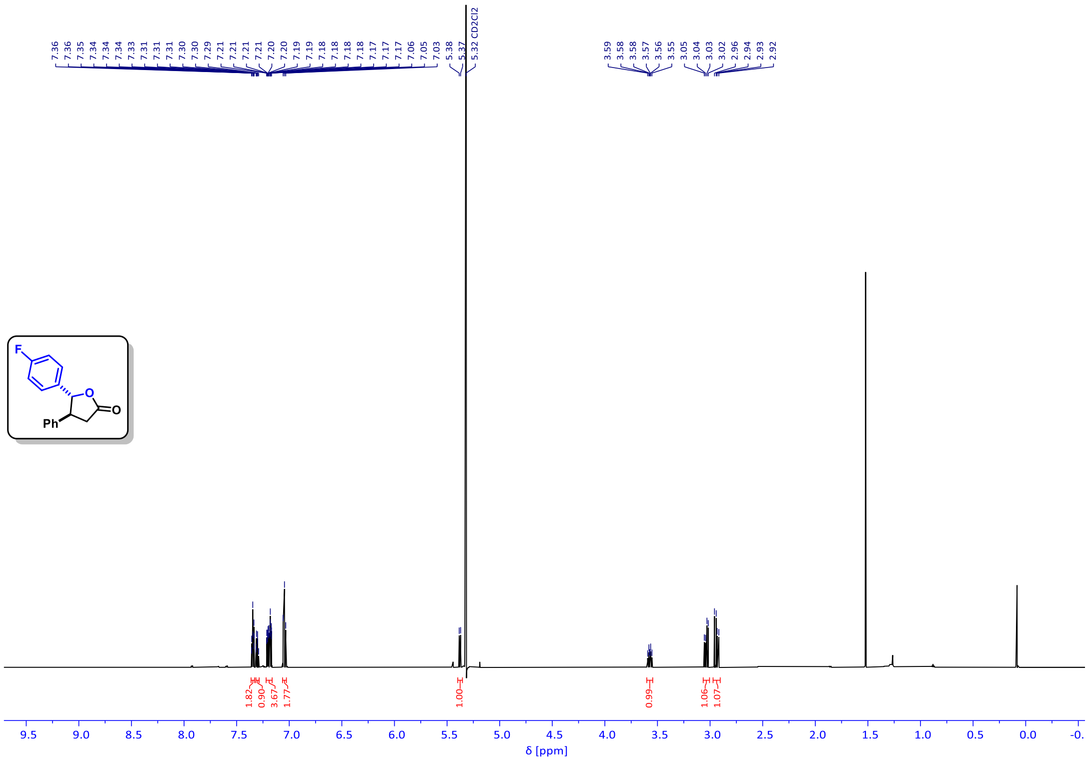

^{1}H-NMR spectrum (700 MHz, CD_2Cl_2) of ***anti*-2c**.

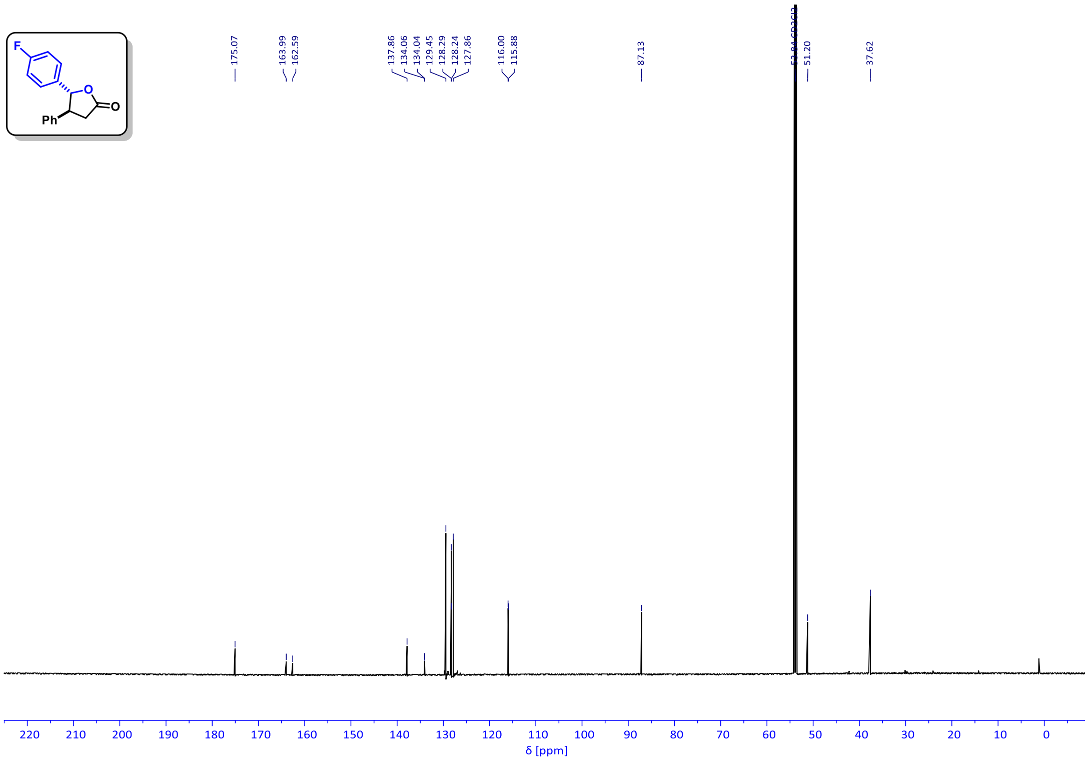

^{13}C-NMR spectrum (176 MHz, CD_2Cl_2) of ***anti*-2c**.

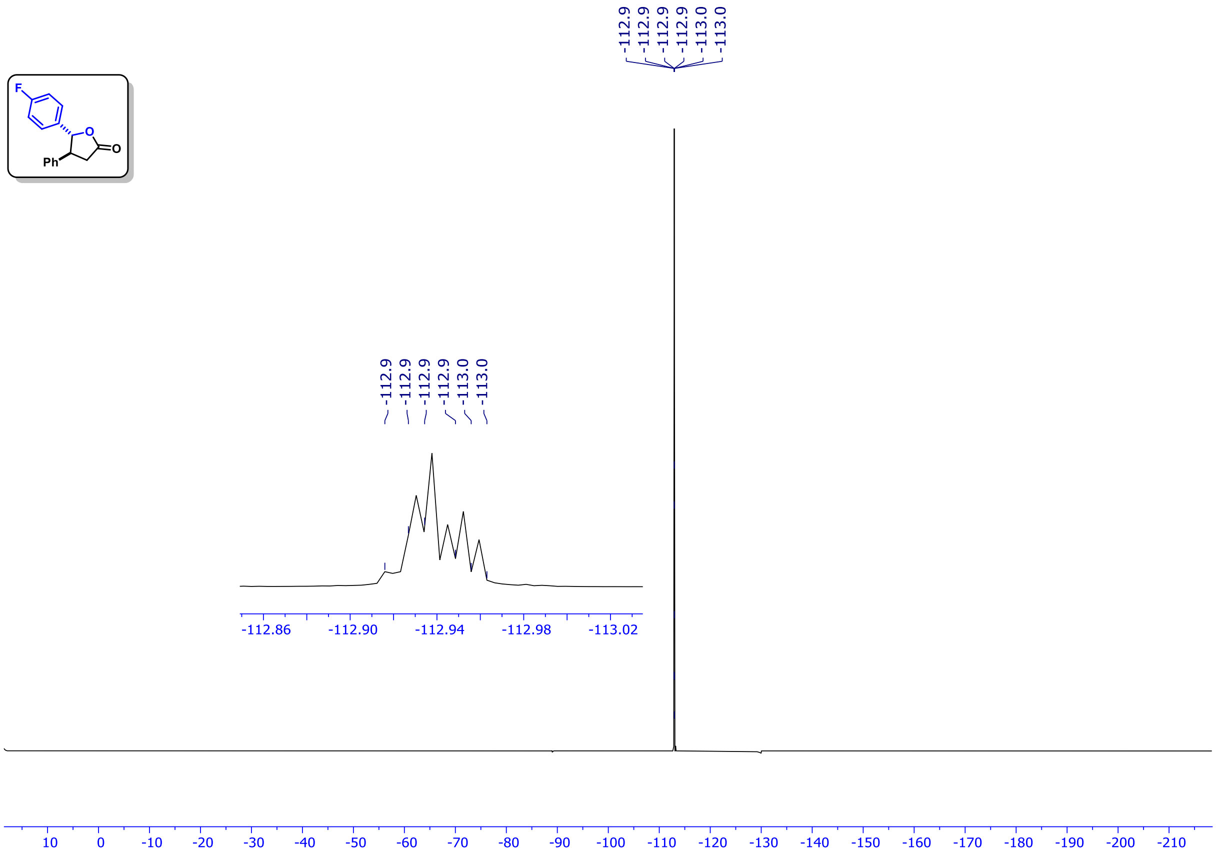

^{19}F-NMR spectrum (659 MHz, $CDCl_3$) of ***anti*-2c**.

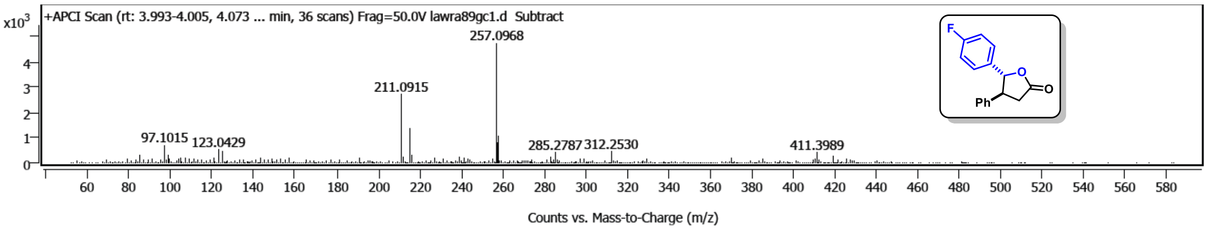

HR-MS Spectrum (APCI,+) of ***anti*-2c**.

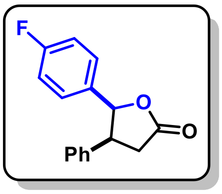

FTIR (ATR): $\tilde{\nu}$ [cm^{-1}] = 1780, 1511, 1225, 1174, 1157, 1142, 1021, 980, 833, 700.

^{1}H-NMR (700 MHz, $CDCl_3$): δ_H (ppm) = 7.19–7.09 (m, 3H), 6.89–6.83 (m, 2H), 6.84–6.79 (m, 4H), 5.80 (d, J = 6.4 Hz, 1H), 4.03 (dt, J = 8.3, 6.4 Hz, 1H), 3.06 (dd, J = 17.5, 8.3 Hz, 1H), 2.94 (dd, J = 17.5, 6.4 Hz, 1H).

^{13}C-NMR (176 MHz, $CDCl_3$): δ_C (ppm) = 176.6, 162.4 (d, J = 246. Hz), 136.7, 131.5 (d, J = 3.1 Hz), 128.6, 128.1, 127.7, 127.6 (d, J = 8.4 Hz), 115.1 (d, J = 22.1 Hz), 84.2, 47.0, 35.1.

HR-MS (APCI, +, Orbitrap): calc. for $C_{16}H_{14}FO_2$ [M+H]$^+$: 257.0972, found: 257.0974.

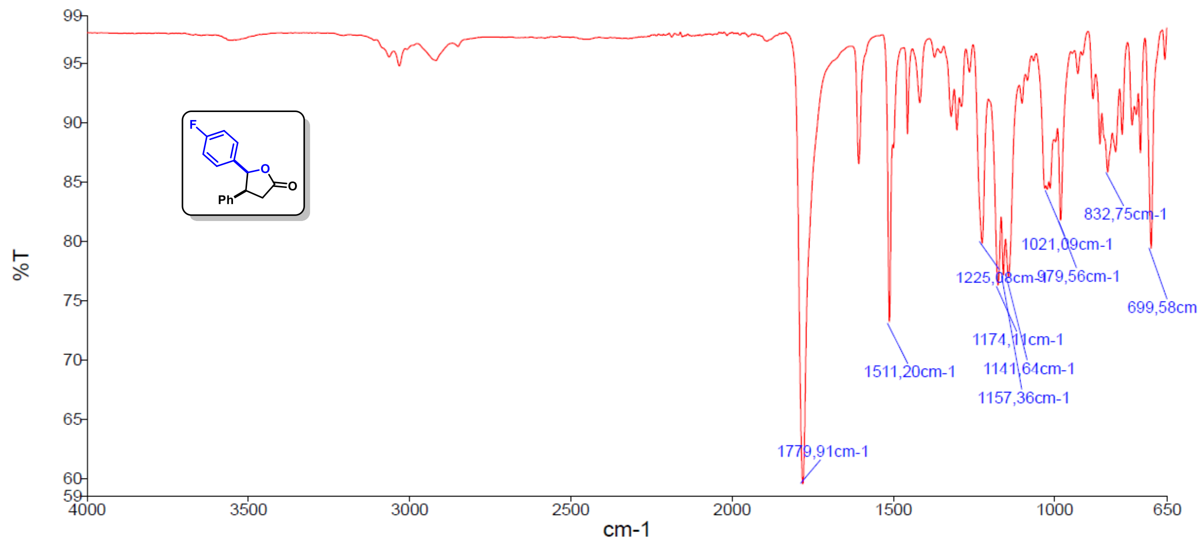

FT-IR Spectrum (ATR, thin film) of ***syn*-2c**.

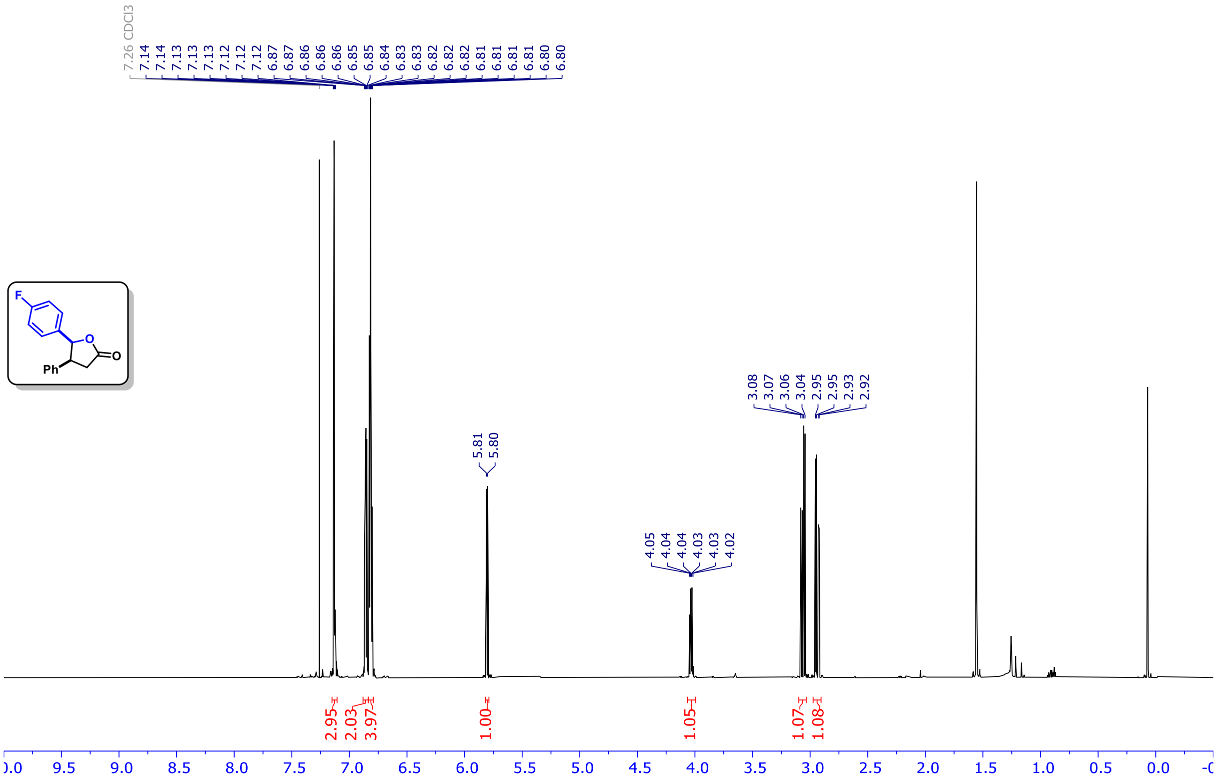

^{1}H-NMR spectrum (700 MHz, $CDCl_3$) of ***syn*-2c**.

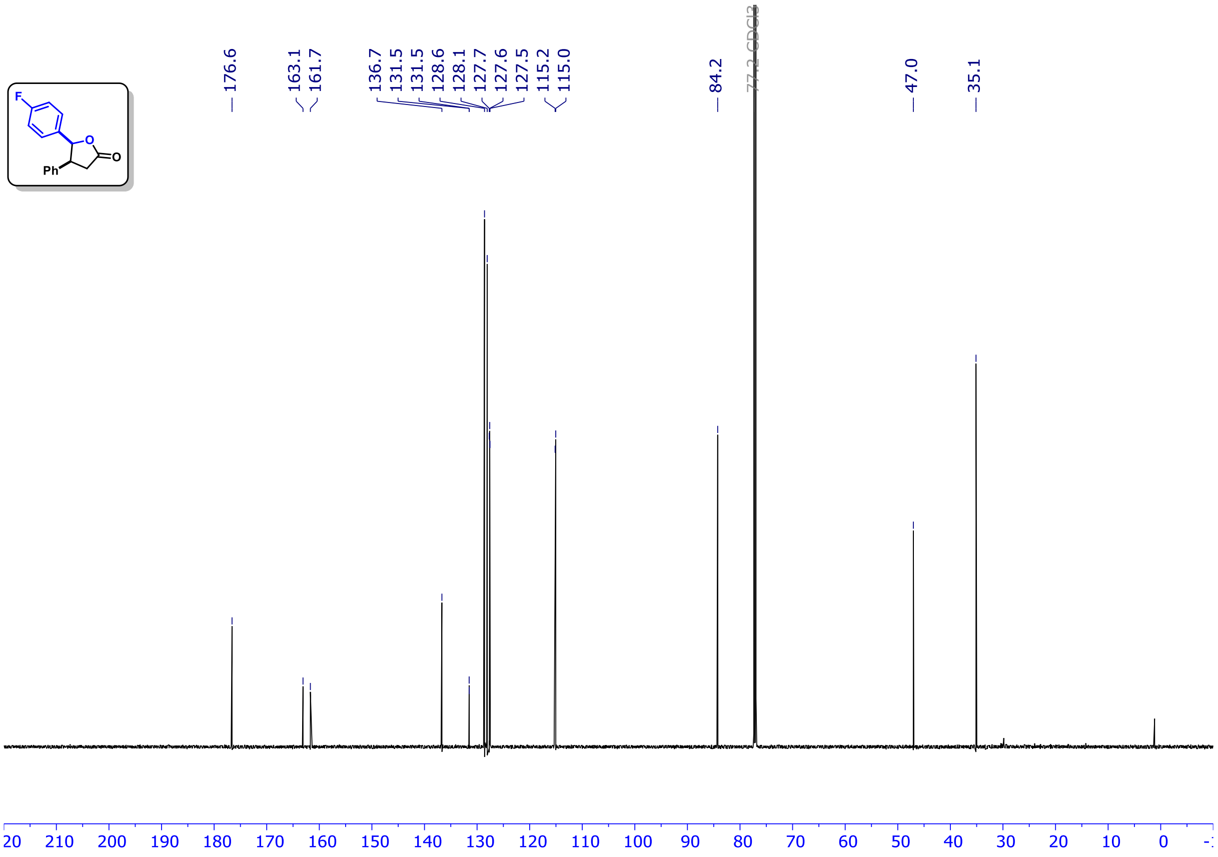

^{13}C-NMR spectrum (176 MHz, $CDCl_3$) of ***syn*-2c**.

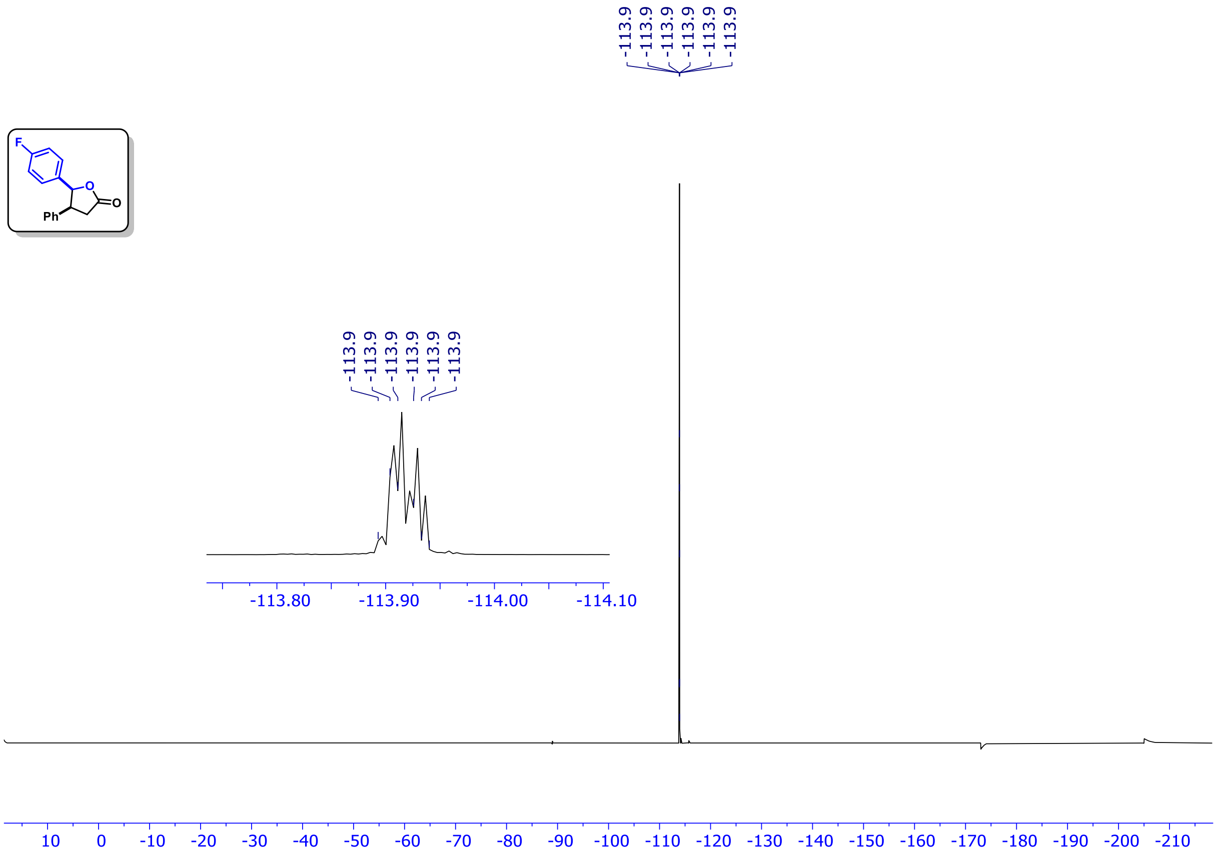

^{19}F-NMR spectrum (659 MHz, $CDCl_3$) of ***syn*-2c**.

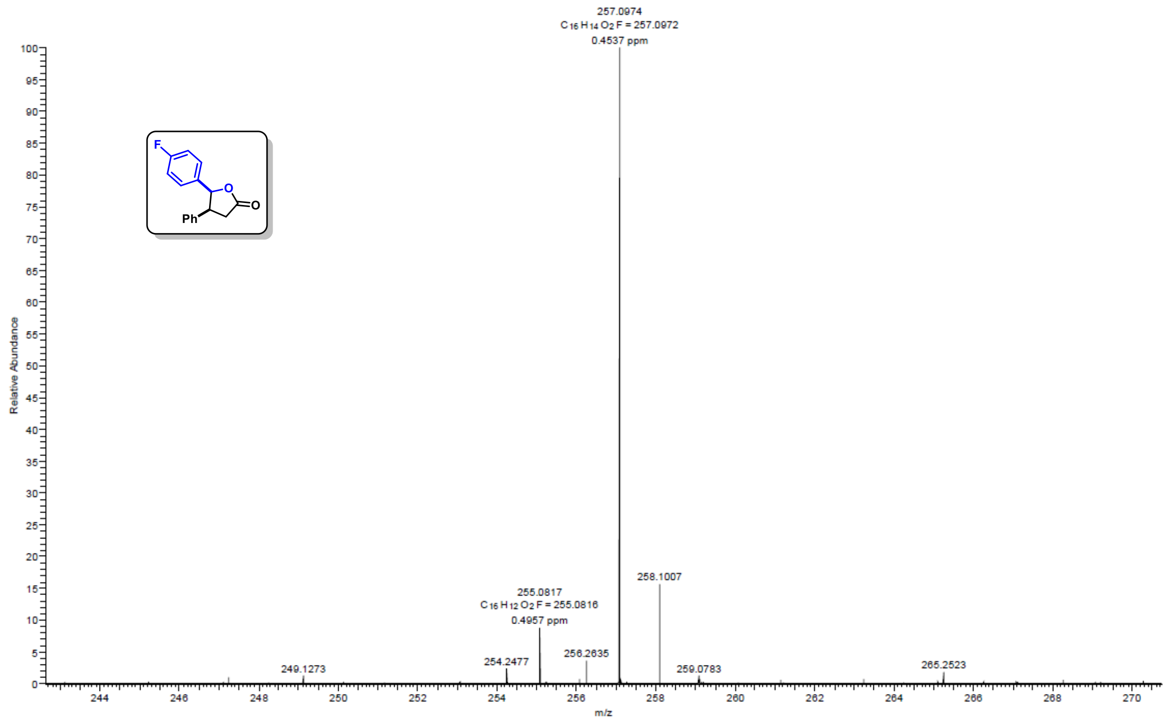

HR-MS Spectrum (APCI,+) of ***syn*-2c**.

Lactone 2d

Prepared according to the **General Procedure** from SCP **1a** (54.9 mg, 0.20 mmol, 1.00 eq.) and furfural (154 mg, 1.60 mmol, 133 µL, 8.00 eq.). Purification by flash column chromatography (SiO_2, *n*-pentane/EtOAc 12:1 to 8:1) afforded the lactones ***anti*-2d** (23.6 mg, 0.10 mmol, 52%) and ***syn*-2d** (9.1 mg, 0.04 mmol, 20%) as colorless solids (overall yield: 72%, *dr* 2.6:1).

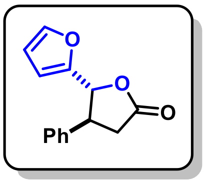

FTIR (ATR): $\tilde{\nu}$ [cm^{-1}] = 1779, 1499, 1272, 1192, 1145, 1014, 976, 936, 751, 699.

^{1}H-NMR (700 MHz, acetone-*d*$_6$): δ_H (ppm) = 7.63 (dd, *J* = 1.9, 0.8 Hz, 1H), 7.41–7.38 (m, 2H), 7.36–7.32 (m, 2H), 7.29–7.25 (m, 1H), 6.62 (dd, *J* = 3.4, 0.8 Hz, 1H), 6.44 (dd, *J* = 3.4, 1.9 Hz, 1H), 5.54 (d, *J* = 9.4 Hz, 1H), 4.15 (ddd, *J* = 11.2, 9.4, 8.6 Hz, 1H), 3.06 (dd, *J* = 17.2, 8.6 Hz, 1H), 3.00 (dd, *J* = 17.2, 11.2 Hz, 1H).

^{13}C-NMR (176 MHz, acetone-*d*$_6$): δ_C (ppm) = 174.9 (qC), 151.0 (qC), 144.9 (CH), 139.3 (qC), 129.7 (CH), 128.4 (CH), 128.4 (CH), 111.5 (CH), 111.5 (CH), 80.3 (CH), 46.5 (CH), 37.4 (CH_2).

HR-MS (APCI, +, Orbitrap): calc. for $C_{14}H_{13}O_3$ $[M+H]^+$: 229.0859, found: 229.0859.

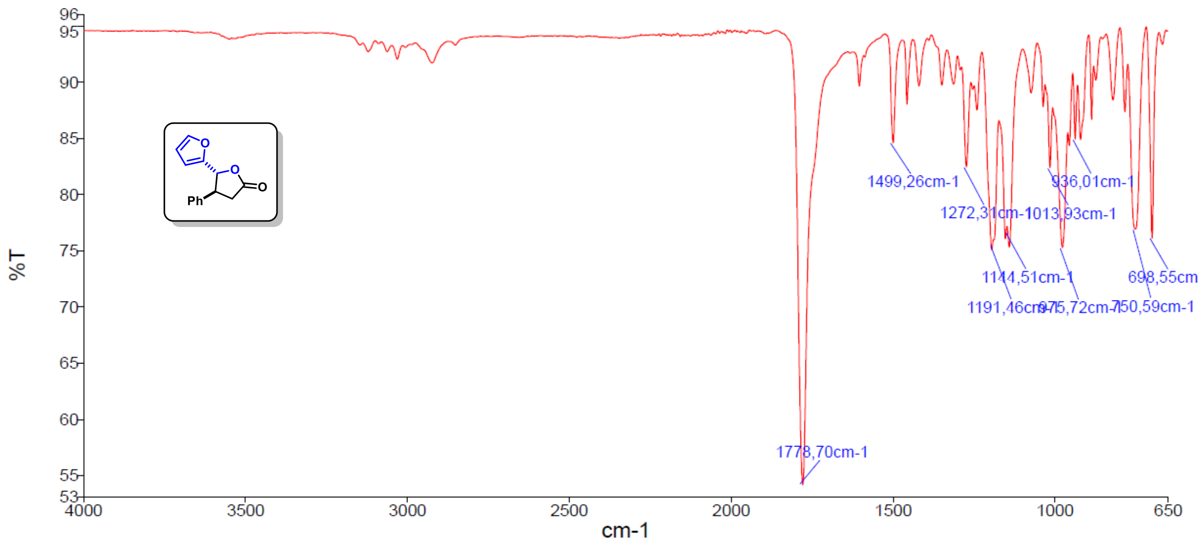

FT-IR Spectrum (ATR, thin film) of ***anti*-2d**.

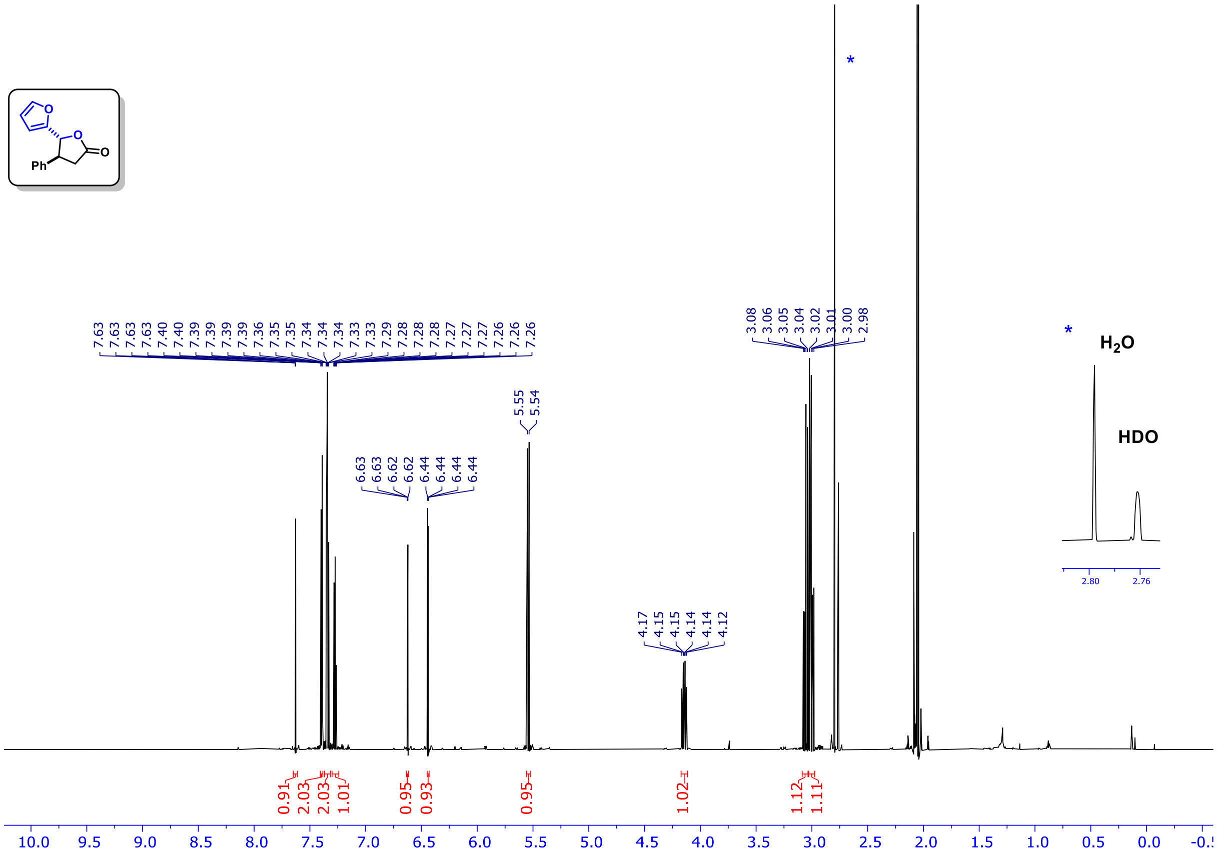

^{1}H-NMR spectrum (700 MHz, acetone-d_6) of ***anti*-2d**.

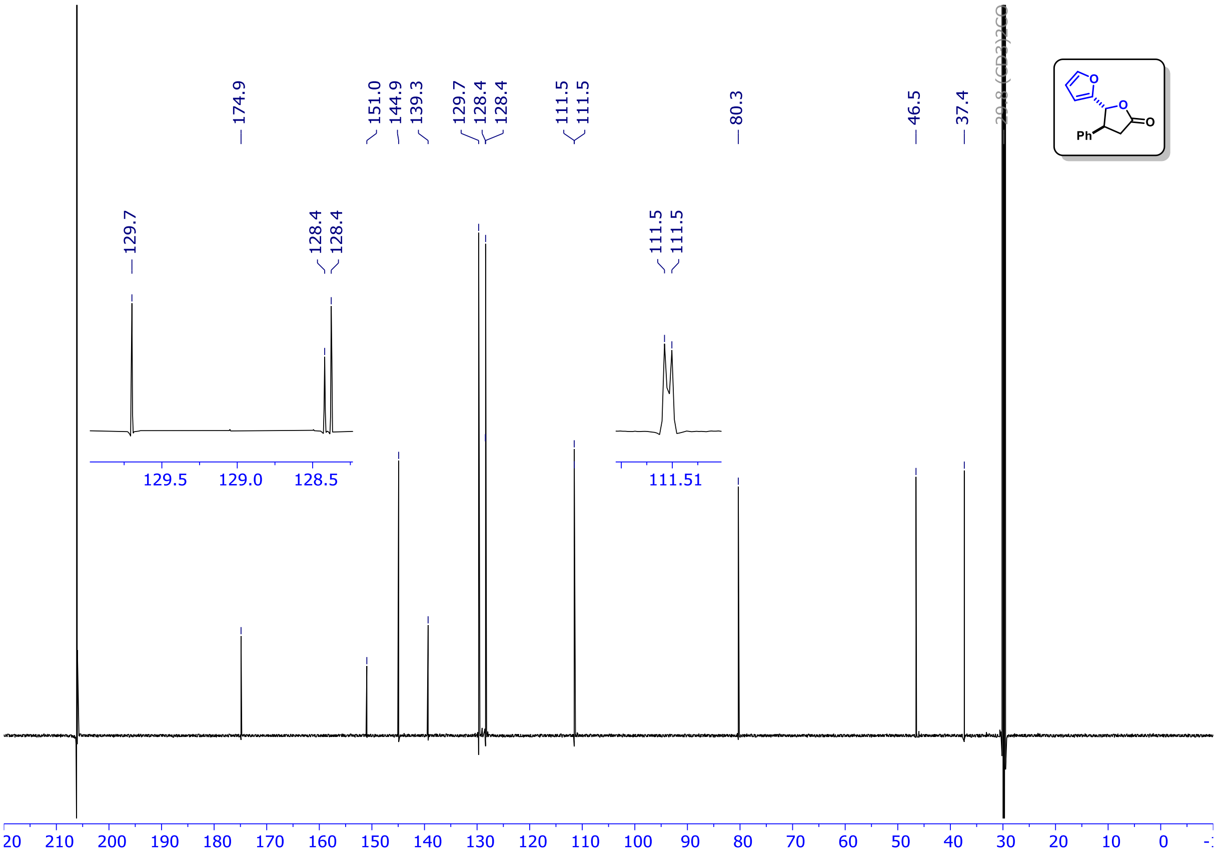

^{13}C-NMR spectrum (176 MHz, acetone-d_6) of ***anti*-2d**.

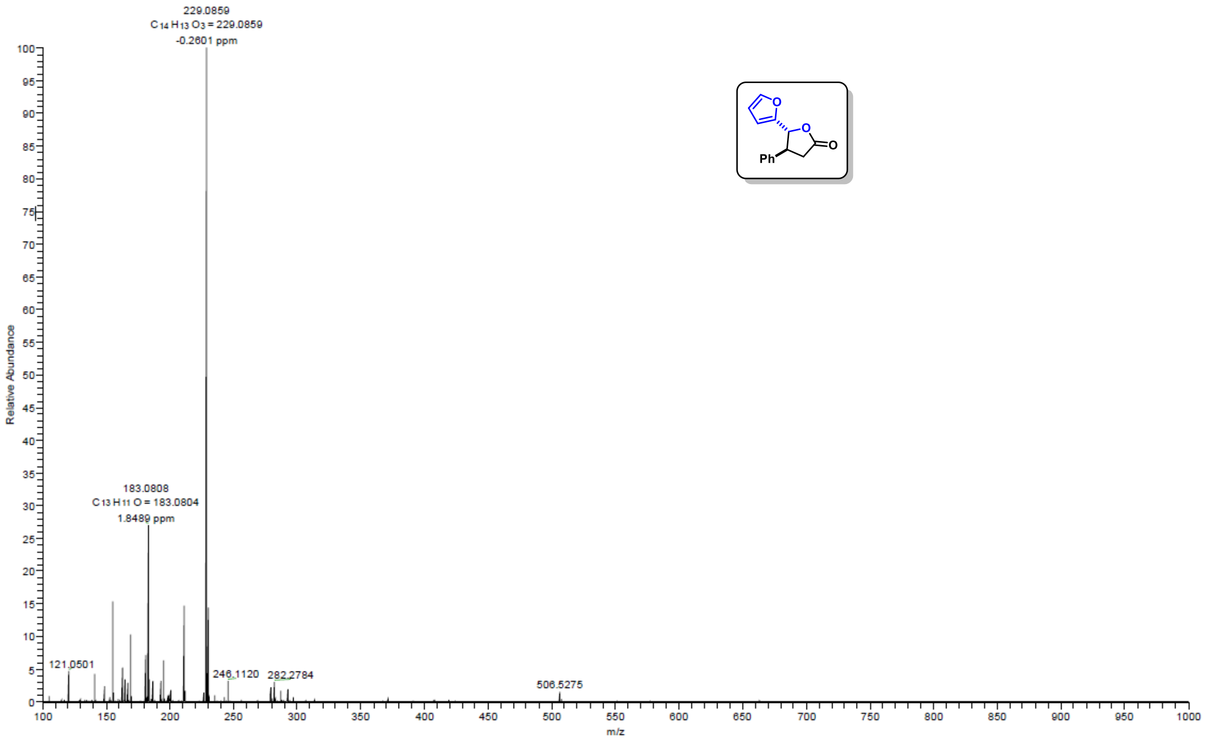

HR-MS Spectrum (APCI,+) of ***anti*-2d**.

FTIR (ATR): $\tilde{\nu}$ [cm^{-1}] = 1777, 1308, 1152, 993, 972, 912, 886, 786, 745, 698.

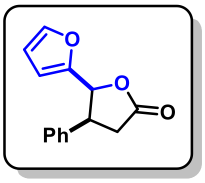

^{1}H-NMR (700 MHz, acetone-d_6): δ_H (ppm) = 7.39 (dd, J = 1.9, 0.9 Hz, 1H), 7.23–7.20 (m, 2H), 7.18–7.14 (m, 3H), 6.20 (dd, J = 3.3, 1.9 Hz, 1H), 6.14 (dd, J = 3.3, 0.9 Hz, 1H), 5.92 (d, J = 8.1 Hz, 1H), 4.31 (dt, J = 11.6, 8.1 Hz, 1H), 3.26 (dd, J = 17.0, 11.6 Hz, 1H), 2.92 (dd, J = 17.0, 8.1 Hz, 1H).

^{13}C-NMR (176 MHz, acetone-d_6): δ_C (ppm) = 176.2, 151.0, 143.9, 137.7, 129.0, 128.5, 128.0, 111.0, 110.8, 78.7, 46.0, 33.1.

HR-MS (APCI, +): calc. for $C_{14}H_{13}O_3$ [M+H]$^+$: 229.0859, found: 229.0856.

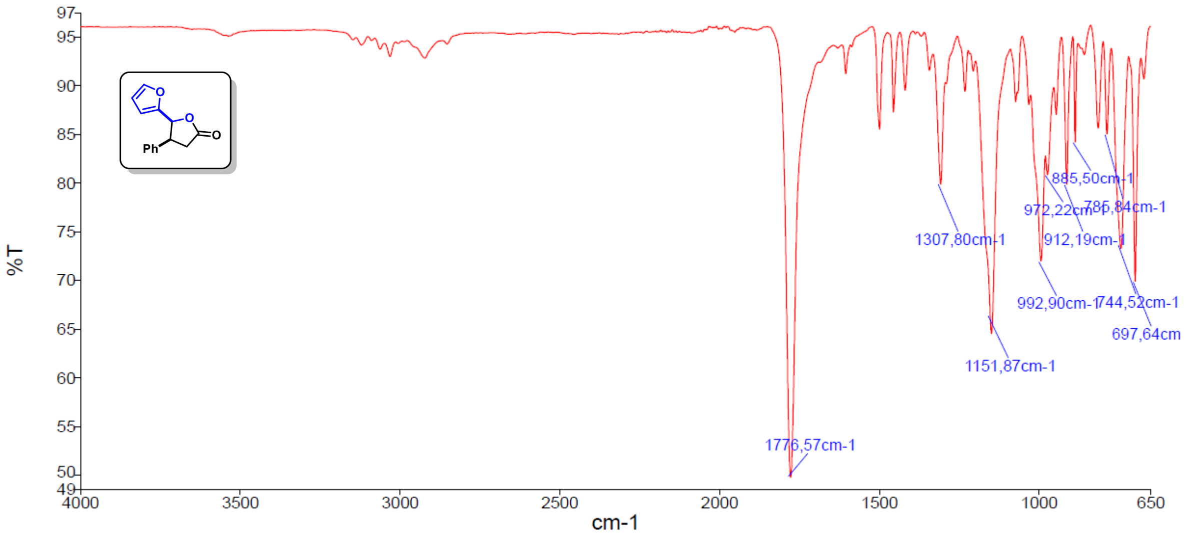

FT-IR Spectrum (ATR, thin film) of ***syn*-2d**.

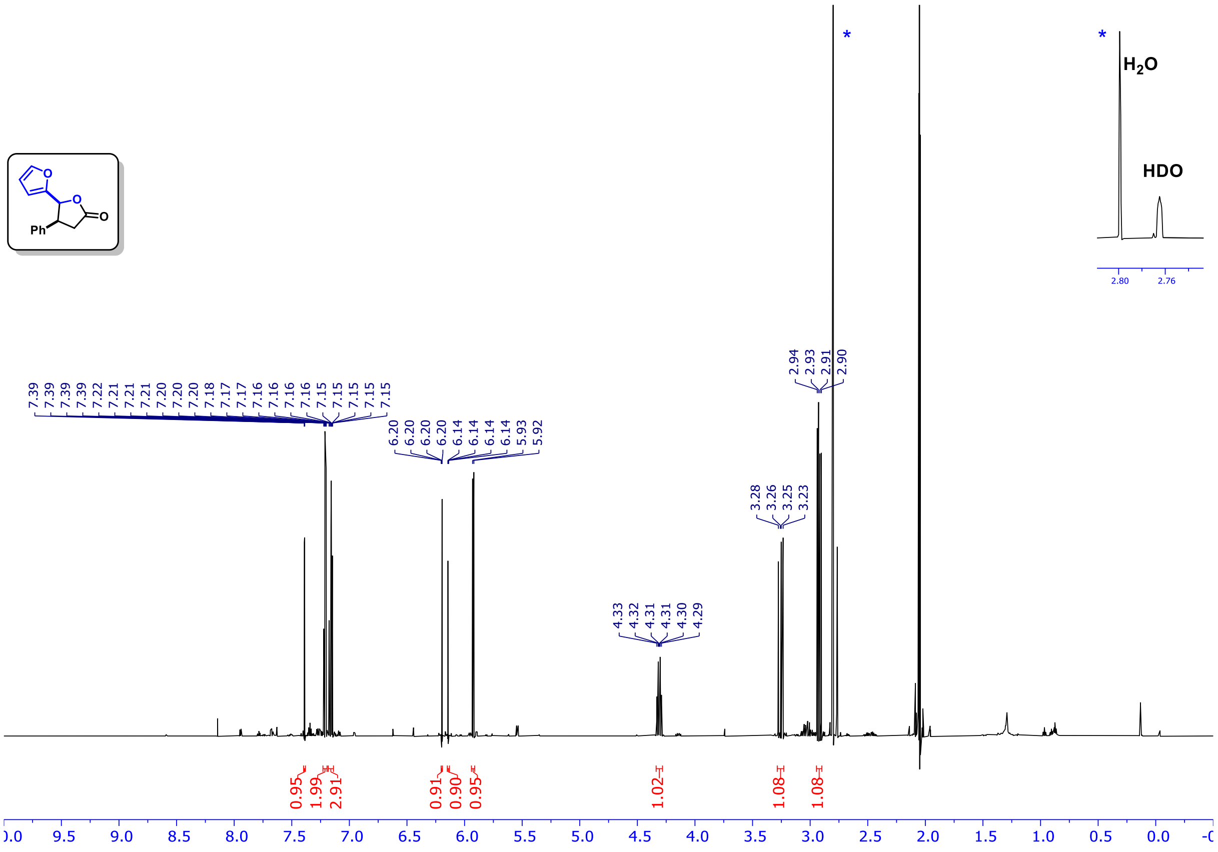

^{1}H-NMR spectrum (700 MHz, acetone-d_6) of ***syn*-2d**.

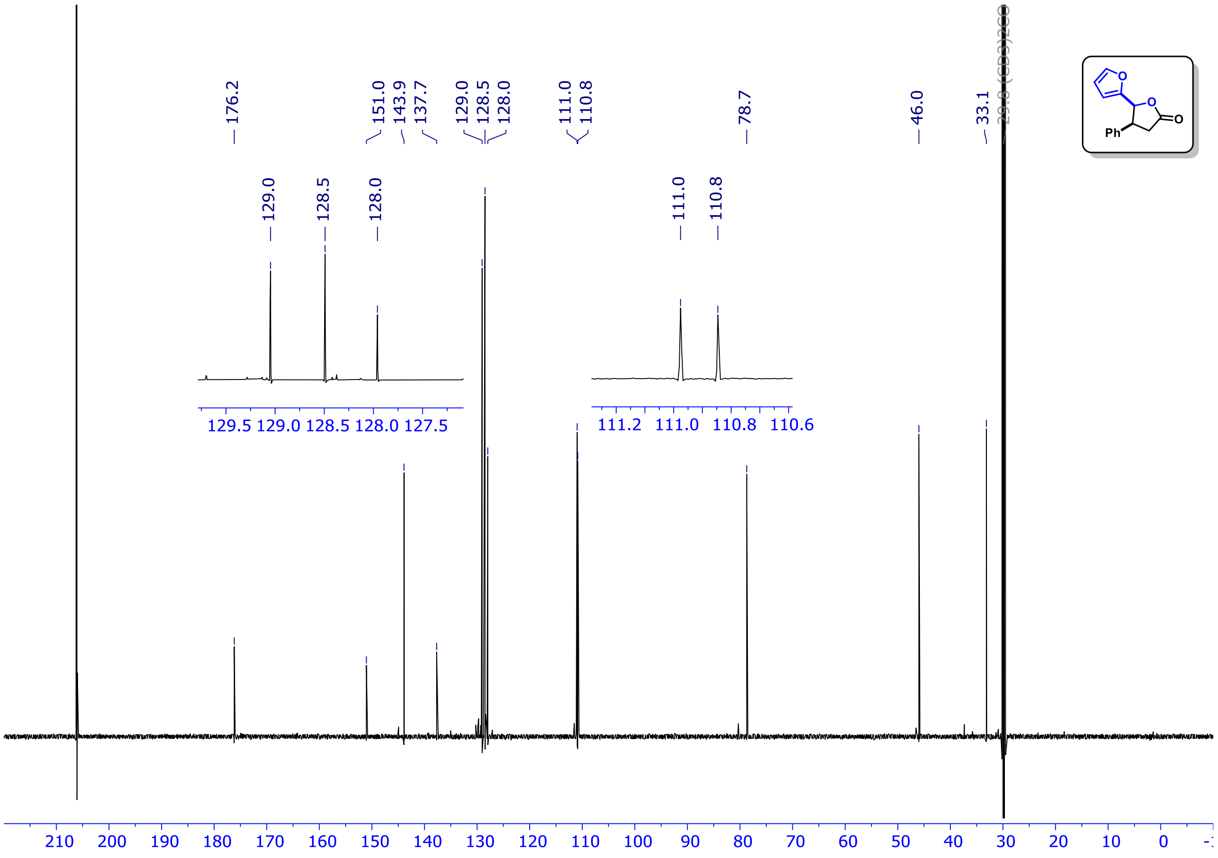

^{13}C-NMR spectrum (176 MHz, acetone-d_6) of ***syn*-2d**.

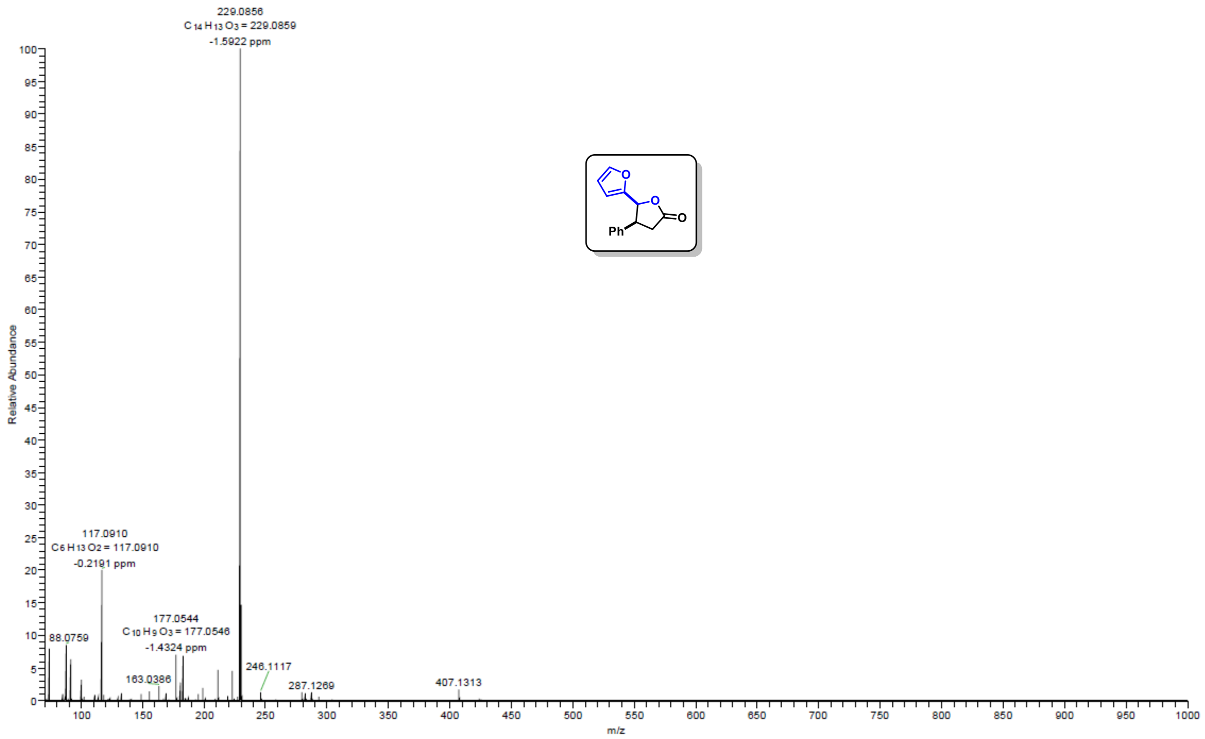

HR-MS Spectrum (APCI,+) of ***syn*-2d**.

Lactone 2e

Prepared according to the **General Procedure** from SCP **1a** (54.9 mg, 0.20 mmol, 1.00 eq.) and pivaldehyde (138 mg, 1.60 mmol, 175 µL, 8.00 eq.). Purification by flash column chromatography (SiO_2, *n*-pentane/EtOAc 15:1) afforded the lactones ***anti*-2e** (21.6 mg, 0.10 mmol, 49%) and ***syn*-2e** (4.3 mg, 0.02 mmol, 10%) as colorless solids (overall yield: 59%, *dr* 5:1).

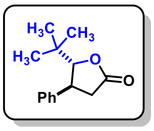

FTIR (ATR): $\tilde{\nu}$ [cm^{-1}] = 1767, 1368, 1192, 1174, 1149, 1044, 999, 758, 748, 699.

^{1}H-NMR (700 MHz, acetone-d_6): δ_H (ppm) = 7.43–7.40 (m, 2H), 7.38–7.34 (m, 2H), 7.29–7.25 (m, 1H), 4.37 (d, J = 7.1 Hz, 1H), 3.66 (ddd, J = 9.7, 8.4, 7.1 Hz, 1H), 2.97 (dd, J = 18.0, 9.7 Hz, 1H), 2.60 (dd, J = 18.0, 8.4 Hz, 1H), 0.92 (s, 9H).

^{13}C-NMR (126 MHz, acetone-d_6): δ_C (ppm) = 175.6, 144.3, 129.8, 128.2, 127.8, 94.2, 42.9, 39.5, 35.6, 25.8.

HR-MS (GC-APCI, +, Q-TOF): calc. for $C_{14}H_{19}O_2$ $[M+H]^+$: 219.1380, found: 219.1384.

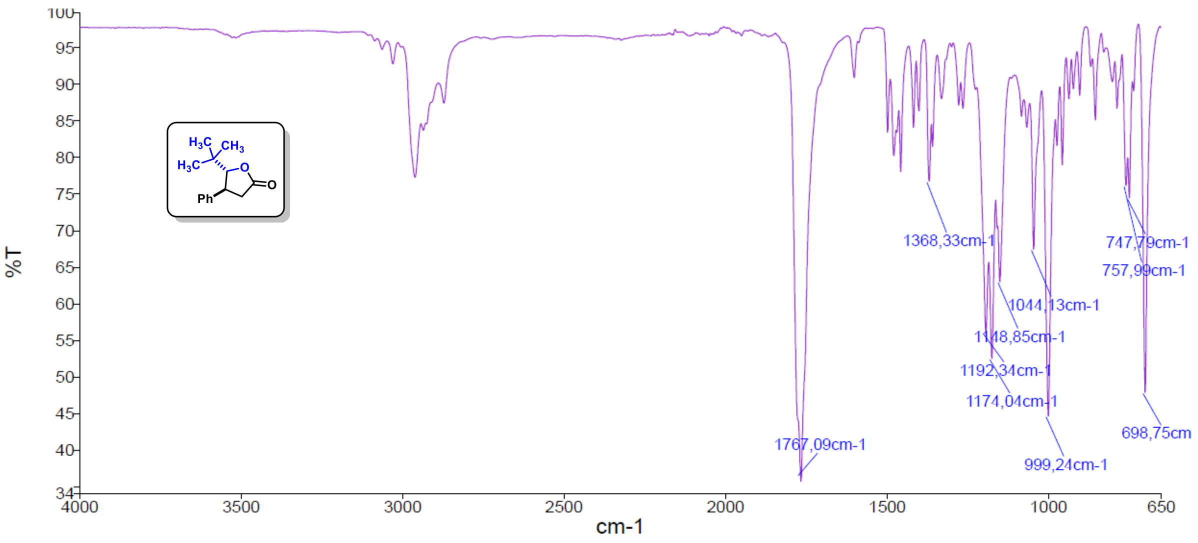

FT-IR Spectrum (ATR, thin film) of ***anti*-2e**.

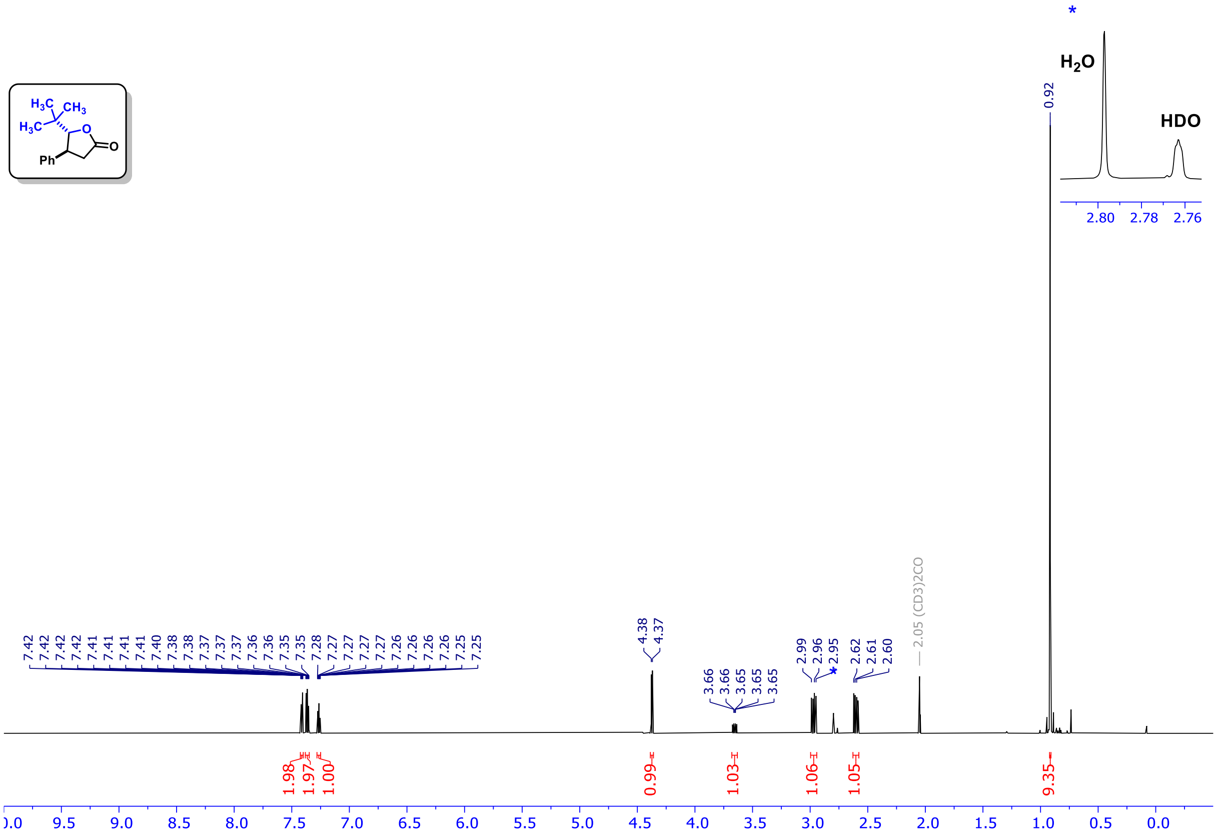

^{1}H-NMR spectrum (700 MHz, acetone-d_6) of ***anti*-2e**.

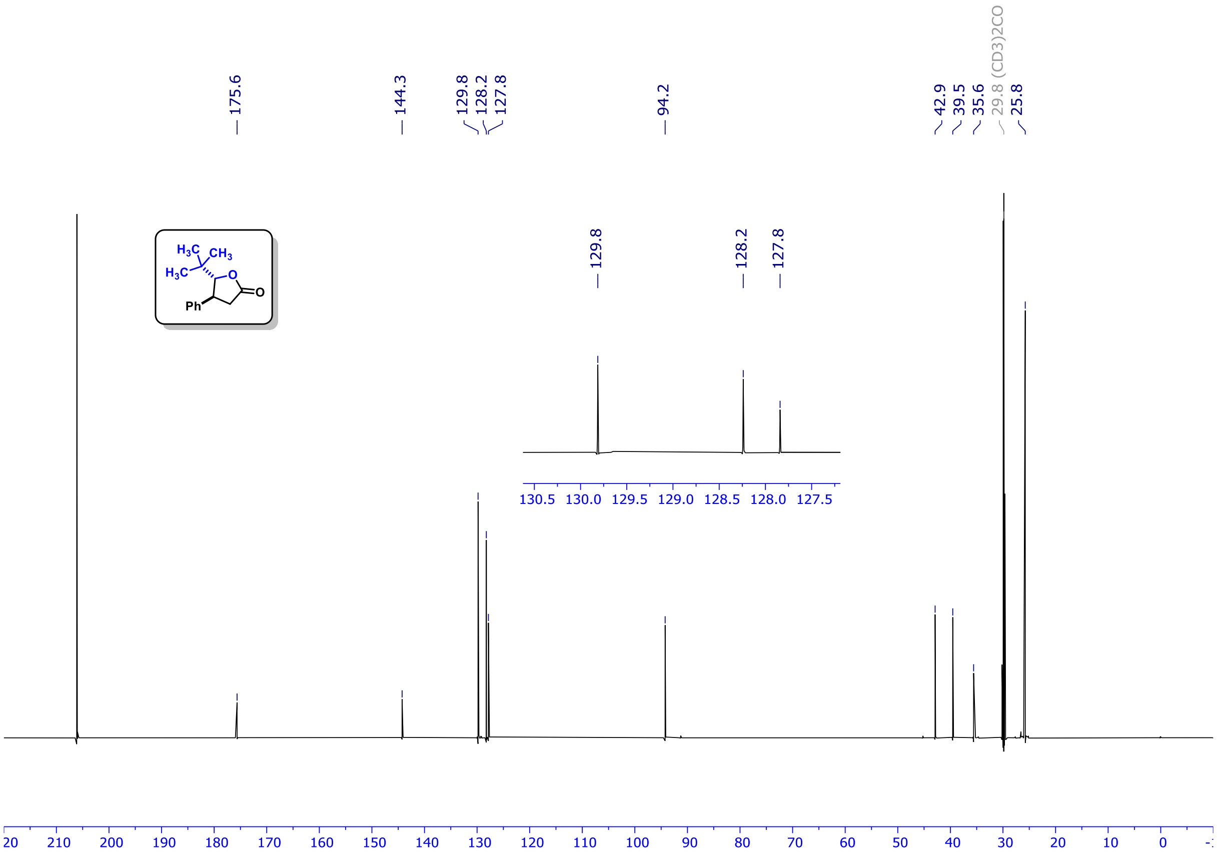

^{13}C-NMR spectrum (176 MHz, acetone-d_6) of ***anti*-2e**.

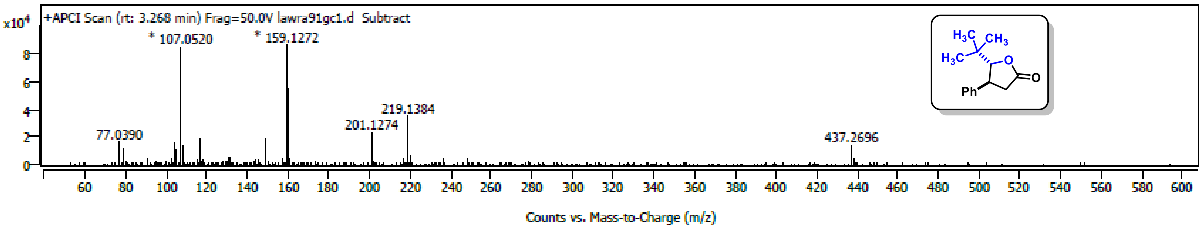

HR-MS Spectrum (APCI,+) of ***anti*-2e**.

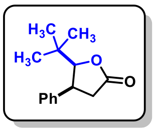

FTIR (ATR): $\tilde{\nu}$ [cm^{-1}] = 2951, 1769, 1455, 1366, 1177, 1144, 1004, 983, 961, 701.

^{1}H-NMR (700 MHz, acetone-d_6): δ_H (ppm) = 7.35–7.32 (m, 2H), 7.31–7.27 (m, 1H), 7.28–7.23 (m, 2H), 4.46 (d, J = 5.4 Hz, 1H), 3.84 (ddd, J = 8.0, 5.4, 2.1 Hz, 1H), 3.17 (dd, J = 17.1, 8.0 Hz, 1H), 2.53 (dd, J = 17.1, 2.1 Hz, 1H), 0.73 (s, 9H).

^{13}C-NMR (126 MHz, acetone-d_6): δ_C (ppm) = 177.5 (qC), 141.2 (qC), 129.3 (CH), 128.2 (CH), 91.3 (CH), 45.2 (CH), 39.6 (CH_2), 34.7 (qC), 26.6 (CH_3). *Due to overlap, not all aromatic signals are resolved (cp. HSQC spectrum).*

HR-MS (GC-APCI, +, Q-TOF): calc. for $C_{14}H_{19}O_2$ [M+H]$^+$: 219.1380, found: 219.1381.

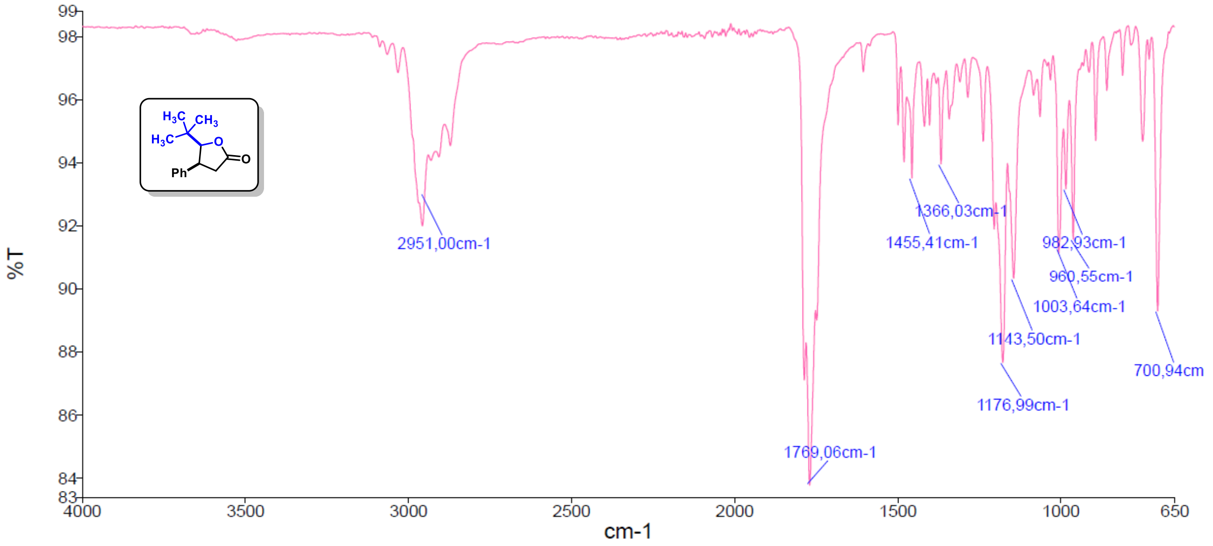

FT-IR Spectrum (ATR, thin film) of ***syn*-2e**.

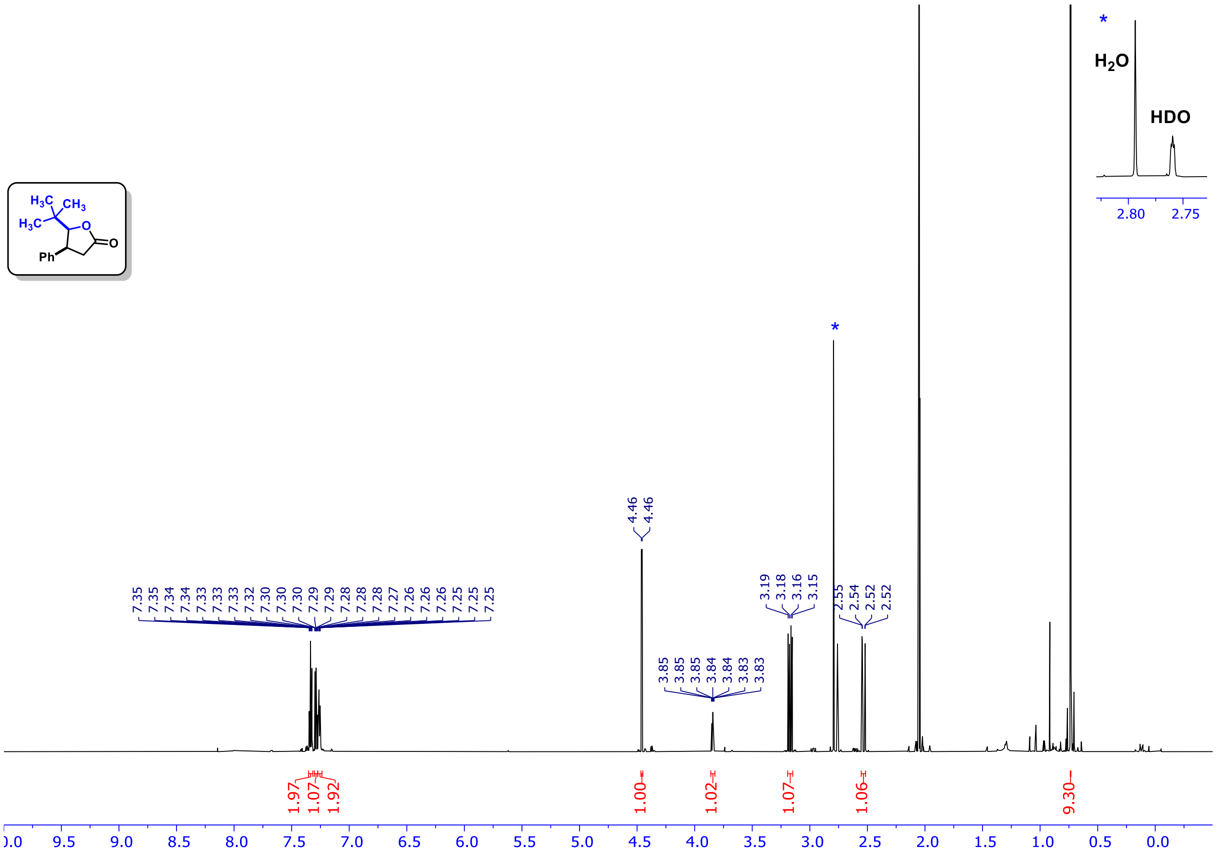

^{1}H-NMR spectrum (700 MHz, acetone-d_6) of ***syn*-2e**.

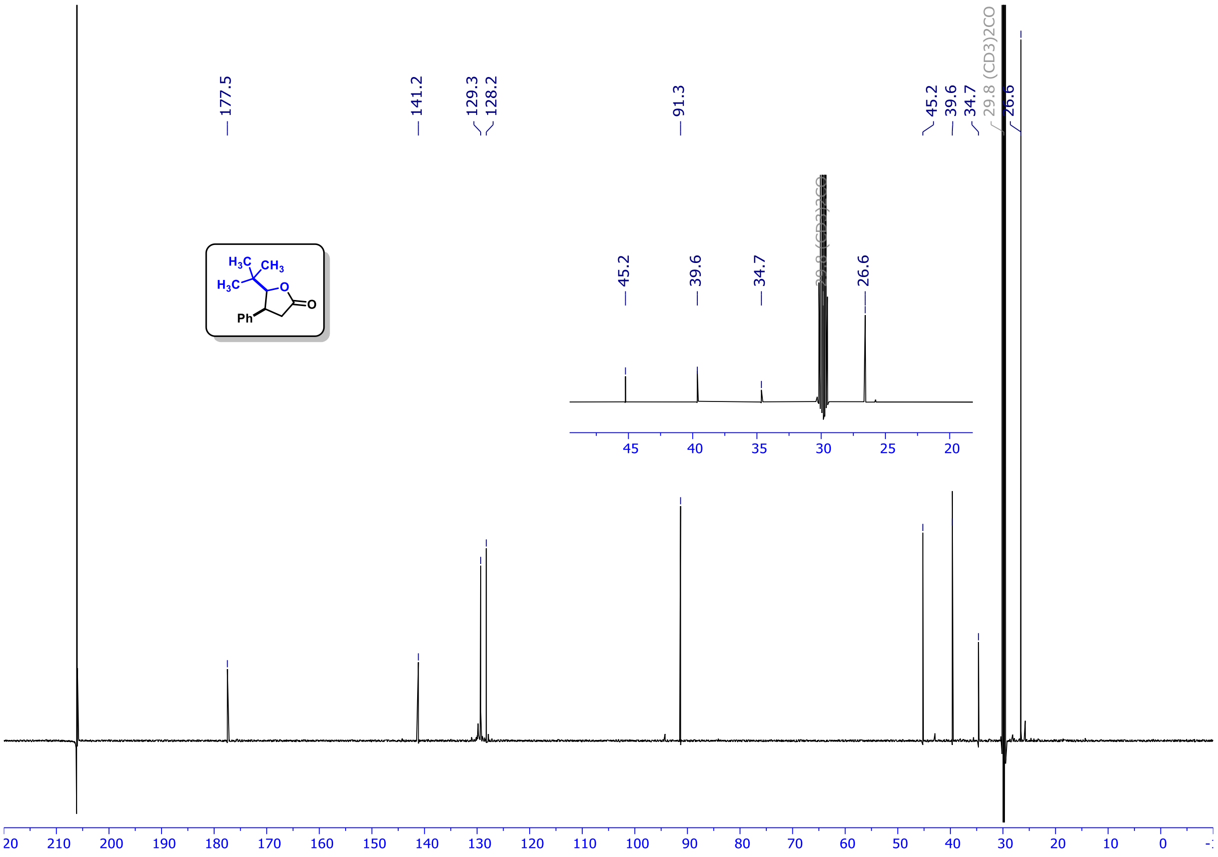

^{13}C-NMR spectrum (176 MHz, acetone-d_6) of ***syn*-2e**.

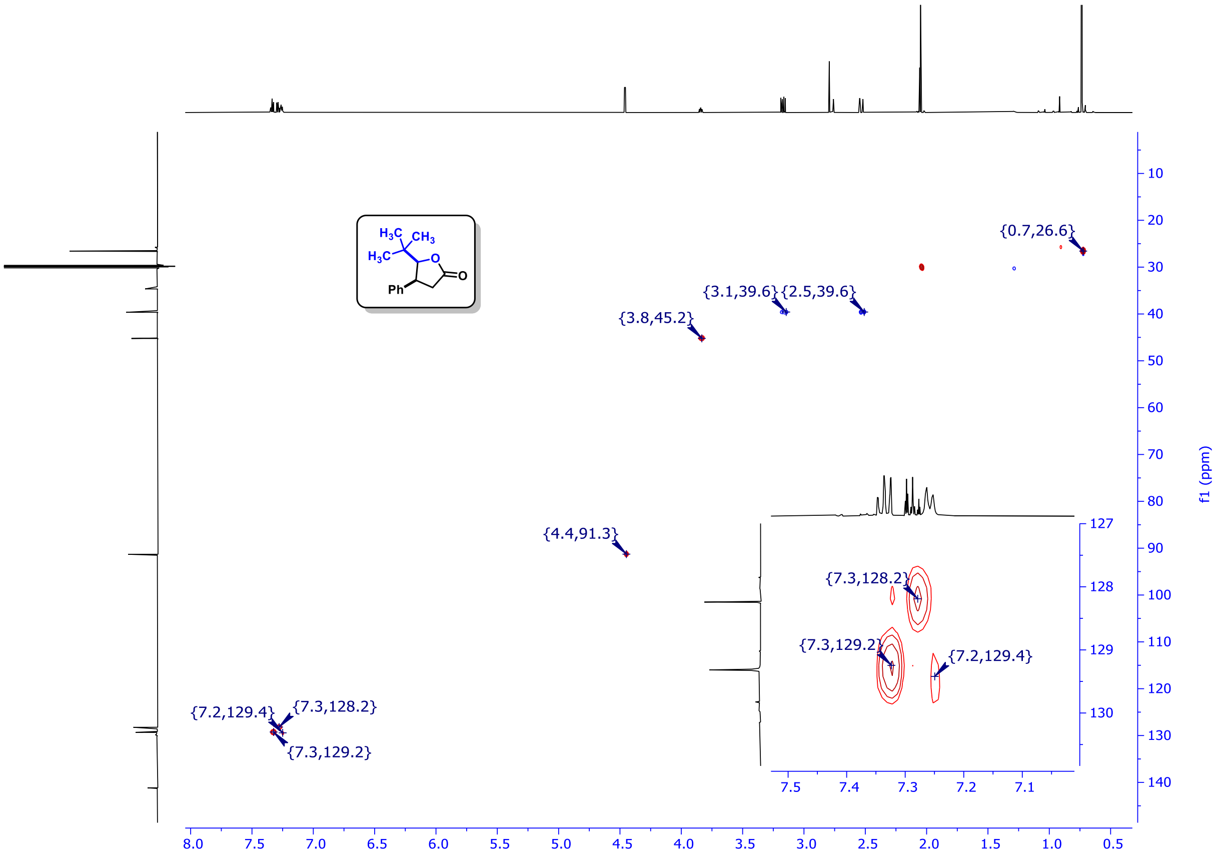

Ed^{1}H,^{13}C-HSQC-spectrum (acetone-d_6) of ***syn*-2e**.

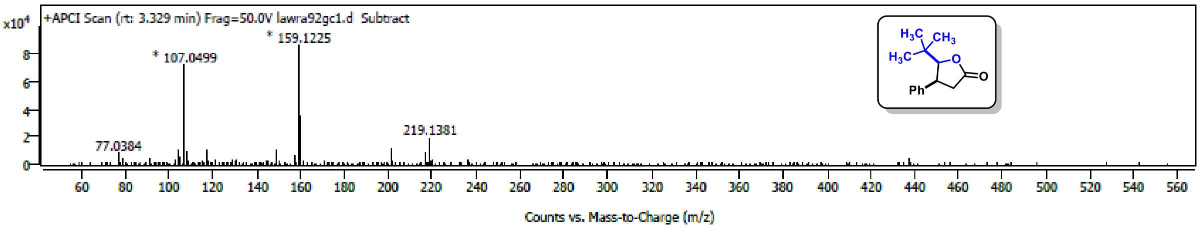

HR-MS Spectrum (APCI,+) of ***syn*-2e**.

Lactone 2f

Prepared according to the **General Procedure** from SCP **1a** (54.9 mg, 0.20 mmol, 1.00 eq.) and *trans*-cinnamaldehyde (211 mg, 1.60 mmol, 201 µL, 8.00 eq.). Purification by flash column chromatography (SiO_2, *n*-pentane/EtOAc 8:1) afforded the lactones ***anti*-2f** (13.3 mg, 0.05 mmol, 25%) and ***syn*-2f** (14.4 mg, 0.05 mmol, 27%) as colorless solids (overall yield: 52%, *dr* 1.1:1).

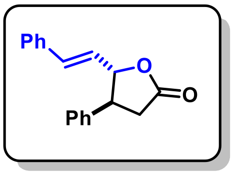

FTIR (ATR): $\tilde{\nu}$ [cm^{-1}] = 1777, 1496, 1452, 1253, 1228, 1199, 1151, 967, 750, 696.

^{1}H-NMR (700 MHz, acetone-d_6): δ_H (ppm) = 7.49–7.43 (m, 4H), 7.39–7.35 (m, 2H), 7.35–7.31 (m, 2H), 7.31–7.25 (m, 2H), 6.62 (dd, J = 15.9, 1.0 Hz, 1H), 6.42 (dd, J = 15.9, 7.5 Hz, 1H), 5.09 (ddd, J = 8.7, 7.5, 1.0 Hz, 1H), 3.69 (dt, J = 10.9, 8.7 Hz, 1H), 2.99–2.91 (m, 2H).

^{13}C-NMR (176 MHz, acetone-d_6): δ_C (ppm) = 175.4, 139.6, 136.9, 134.7, 129.6, 129.5, 129.1, 128.6, 128.3, 127.6, 126.7, 87.3, 49.0, 37.2.

HR-MS (APCI, +, Orbitrap): calc. for $C_{18}H_{17}O_2$ $[M+H]^+$: 265.1223, found: 265.1223.

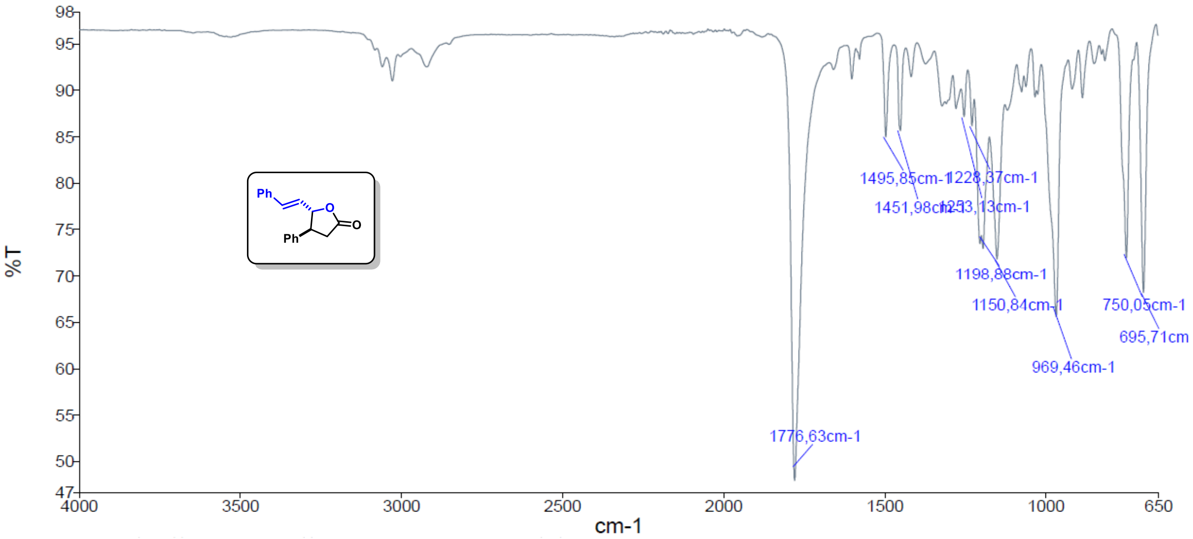

FT-IR Spectrum (ATR, thin film) of ***anti*-2f**.

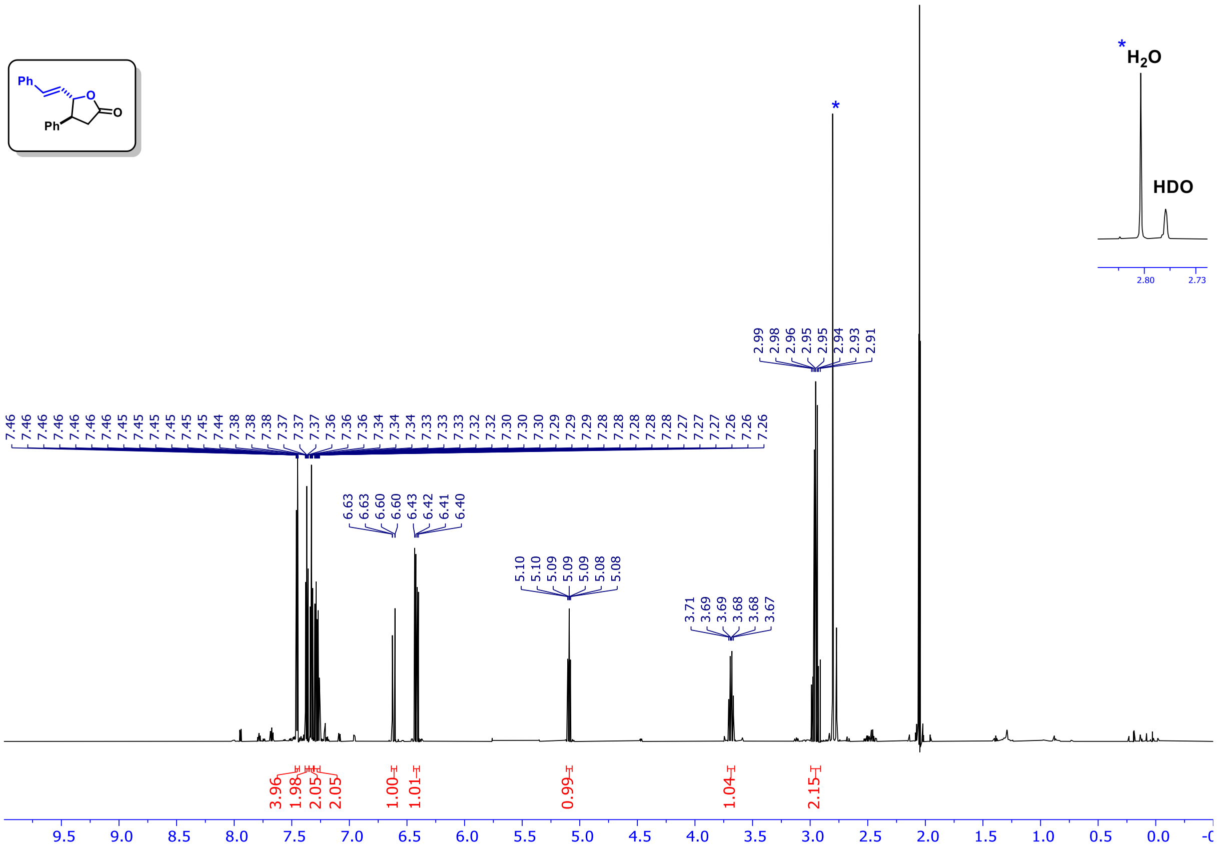

^{1}H-NMR spectrum (700 MHz, acetone-d_6) of ***anti*-2f**.

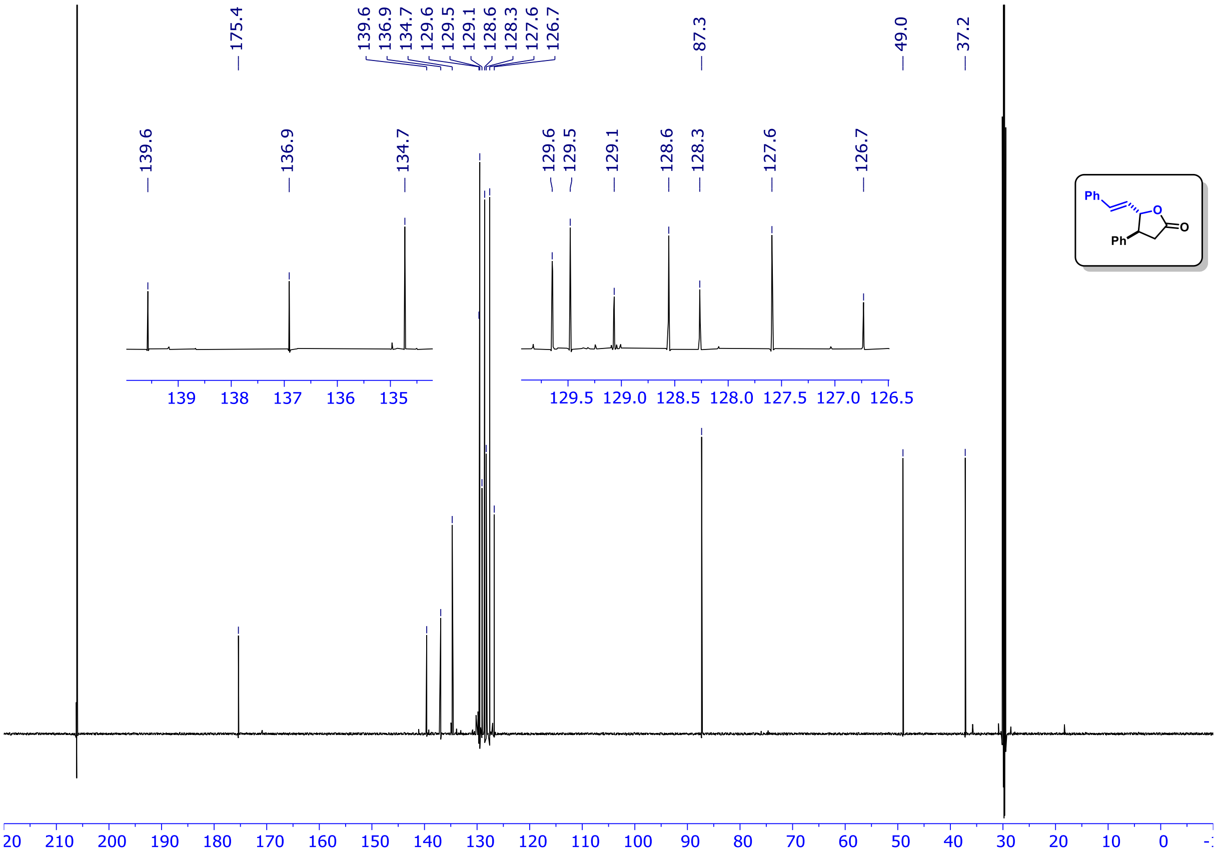

^{13}C-NMR spectrum (176 MHz, acetone-d_6) of ***anti*-2f**.

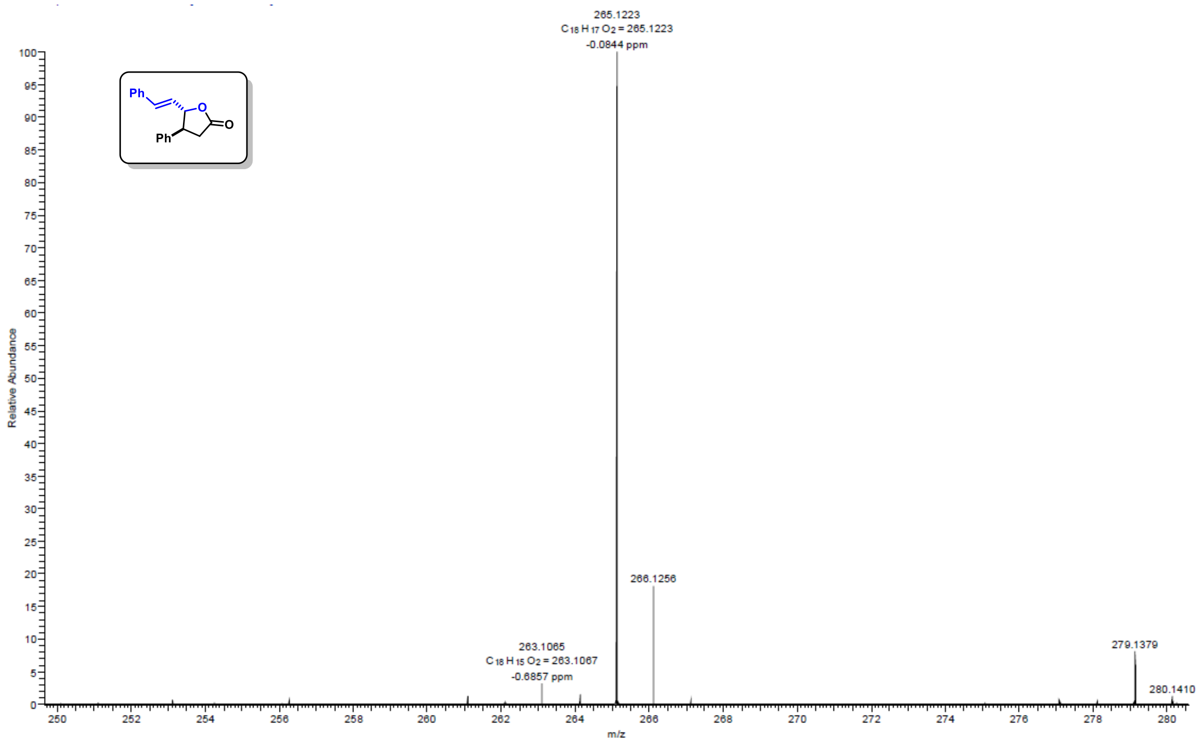

HR-MS Spectrum (APCI,+) of ***anti*-2f**.

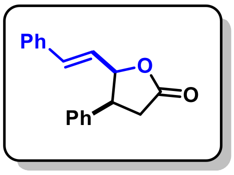

FTIR (ATR): $\tilde{\nu}$ [cm^{-1}] = 1772, 1496, 1452, 1313, 1174, 1148, 1027, 964, 749, 696.

^{1}H-NMR (700 MHz, acetone-d_6): δ_H (ppm) = 7.36–7.32 (m, 2H), 7.30–7.27 (m, 2H), 7.27–7.24 (m, 3H), 7.24–7.19 (m, 3H), 6.61 (dd, J = 15.9, 1.2 Hz, 1H), 5.88 (dd, J = 15.9, 7.7 Hz, 1H), 5.48 (td, J = 7.7, 1.2 Hz, 1H), 4.11 (q, J = 7.7 Hz, 1H), 3.02 (dd, J = 17.2, 7.7 Hz, 1H), 2.95 (dd, J = 17.2, 7.7 Hz, 1H).

^{13}C-NMR (176 MHz, acetone-d_6): δ_C (ppm) = 176.6, 138.9, 137.1, 134.1, 129.4, 129.4, 129.0, 128.9, 128.1, 127.4, 125.8, 84.3, 46.2, 34.5.

HR-MS (APCI, +, Orbitrap): calc. for $C_{18}H_{17}O_2$ $[M+H]^+$: 265.1223, found: 265.1228.

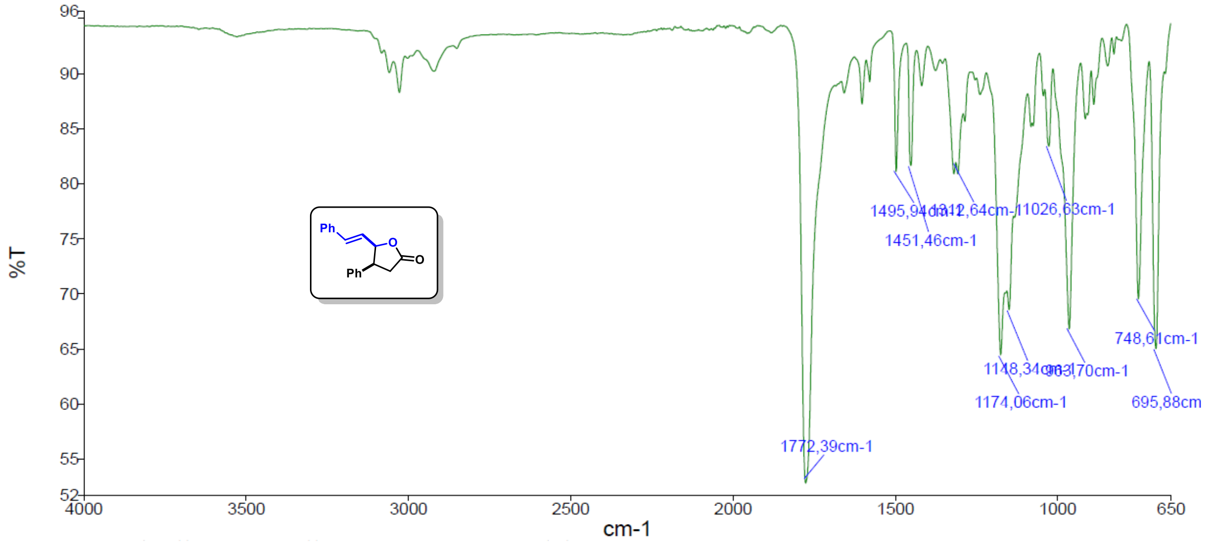

FT-IR Spectrum (ATR, thin film) of ***syn*-2f**.

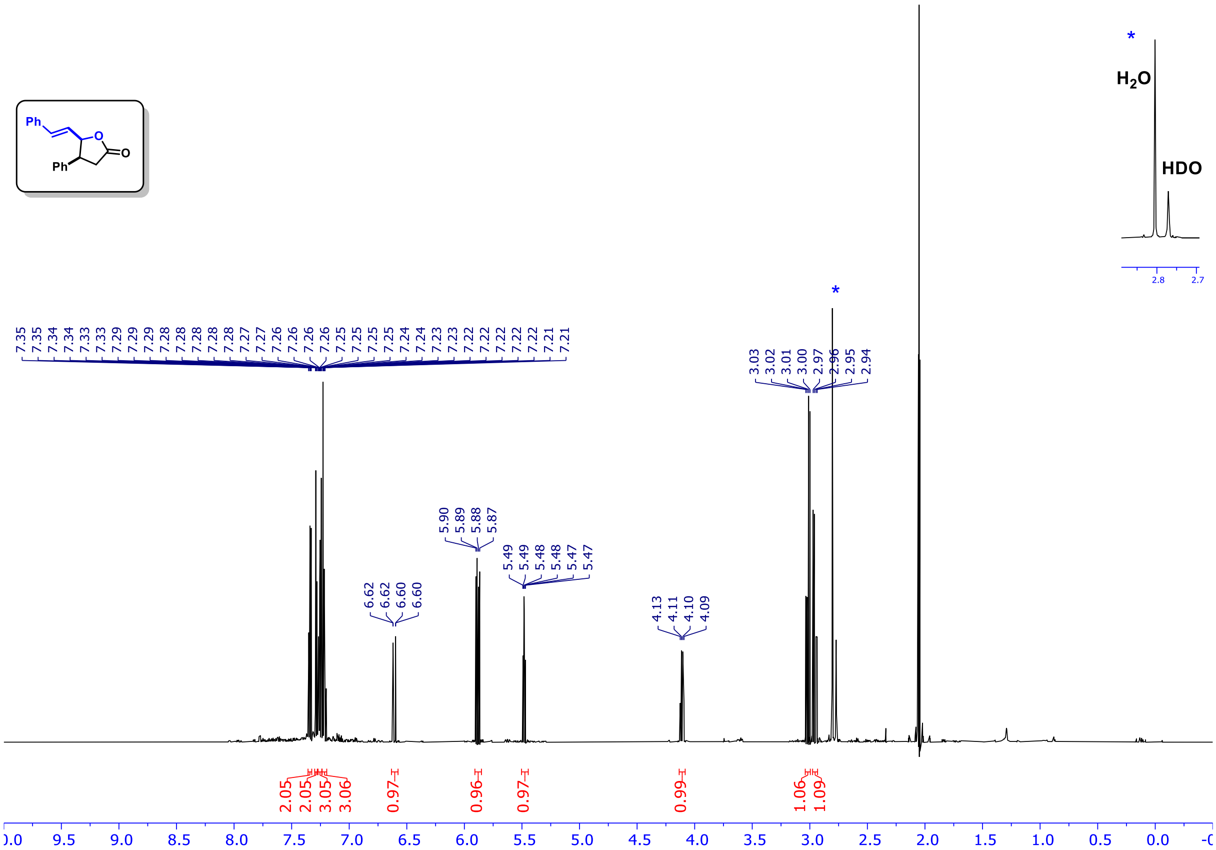

^{1}H-NMR spectrum (700 MHz, acetone-d_6) of ***syn*-2f**.

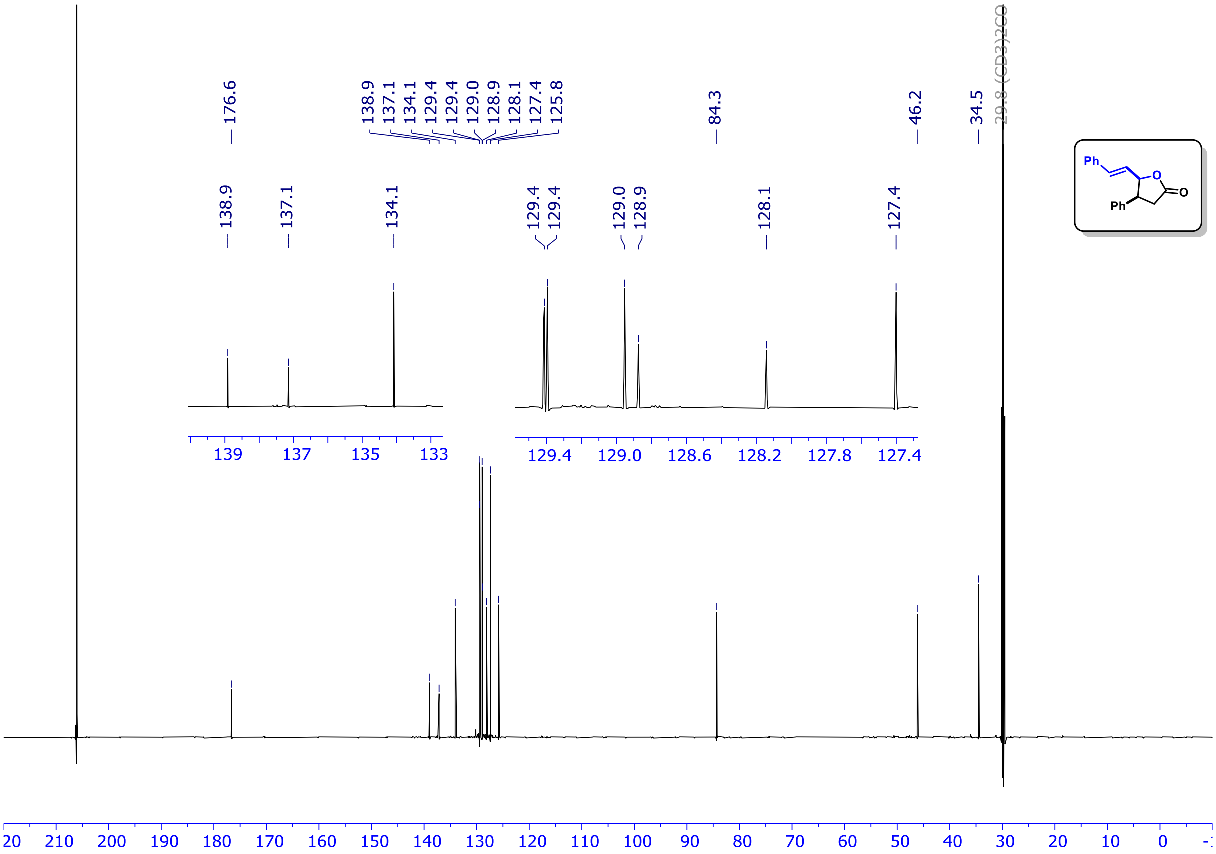

^{13}C-NMR spectrum (176 MHz, acetone-d_6) of ***syn*-2f**.

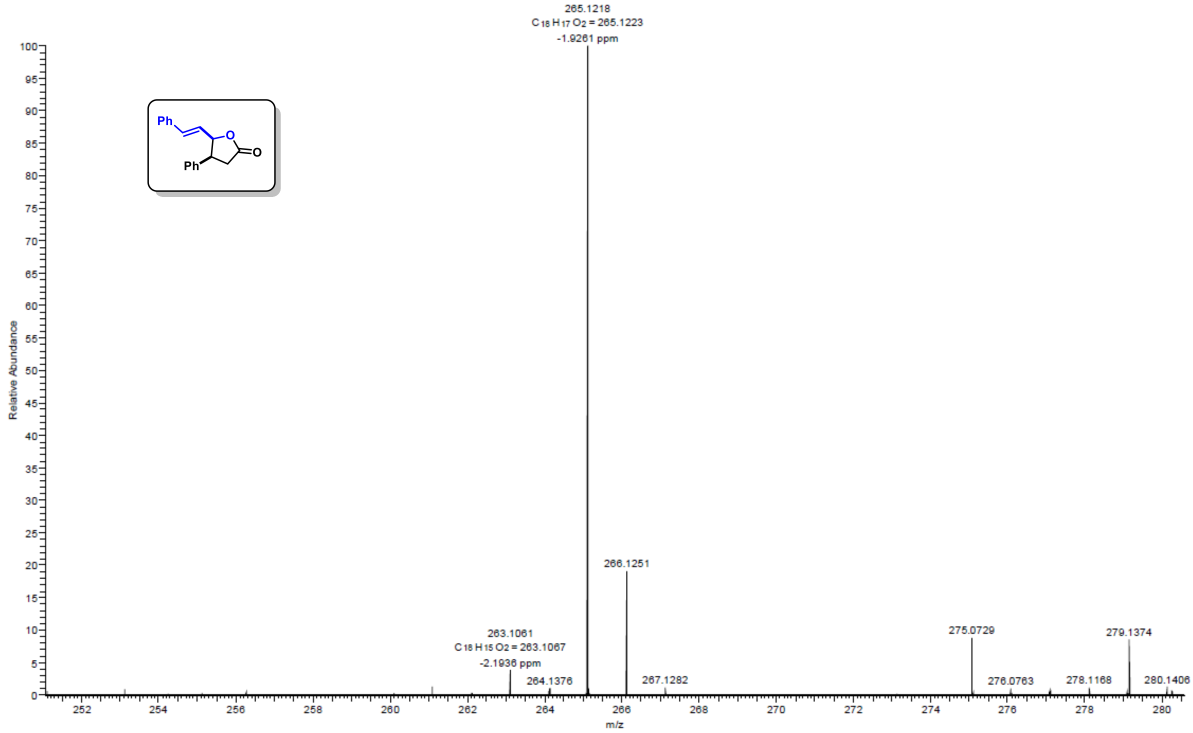

HR-MS Spectrum (APCI,+) of ***syn*-2f**.

Lactone 2g

Prepared according to the **General Procedure** from SCP **1b** (57.7 mg, 0.20 mmol, 1.00 eq.) and benzaldehyde (170 mg, 1.60 mmol, 163 µL, 8.00 eq.). Purification by flash column chromatography (SiO_2, *n*-pentane/EtOAc 20:1 to 4:1) afforded the lactones ***anti*-2g** (20.5 mg, 0.08 mmol, 41%) and ***syn*-2g** (12.8 mg, 0.05 mmol, 25%) as colorless solids (overall yield: 66%, *dr* 1.6:1).

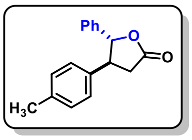

FTIR (ATR): $\tilde{\nu}$ [cm^{-1}] = 1780, 1516, 1268, 1192, 1144, 1032, 997, 812, 757, 699.

^{1}H-NMR (500 MHz, $CDCl_3$): δ_H (ppm) = 7.37–7.31 (m, 3H), 7.23–7.18 (m, 2H), 7.18–7.14 (m, 2H), 7.09–7.04 (m, 2H), 5.41 (d, J = 8.5 Hz, 1H), 3.56 (dt, J = 10.7, 8.5 Hz, 1H), 3.04 (dd, J = 17.6, 8.5 Hz, 1H), 2.90 (dd, J = 17.6, 10.7 Hz, 1H), 2.35 (s, 3H).

^{13}C-NMR (126 MHz, $CDCl_3$): δ_C (ppm) = 175.5 (qC), 138.0 (qC), 137.8 (qC), 135.0 (qC), 129.9 (CH), 128.7 (CH), 127.4 (CH), 125.8 (CH), 87.6 (CH), 50.4 (CH), 37.4 (CH_2), 21.2 (CH_3).

HR-MS (GC-APCI, +, Q-TOF): calc. for $C_{17}H_{17}O_2$ $[M+H]^+$: 253.1223, found: 253.1221.

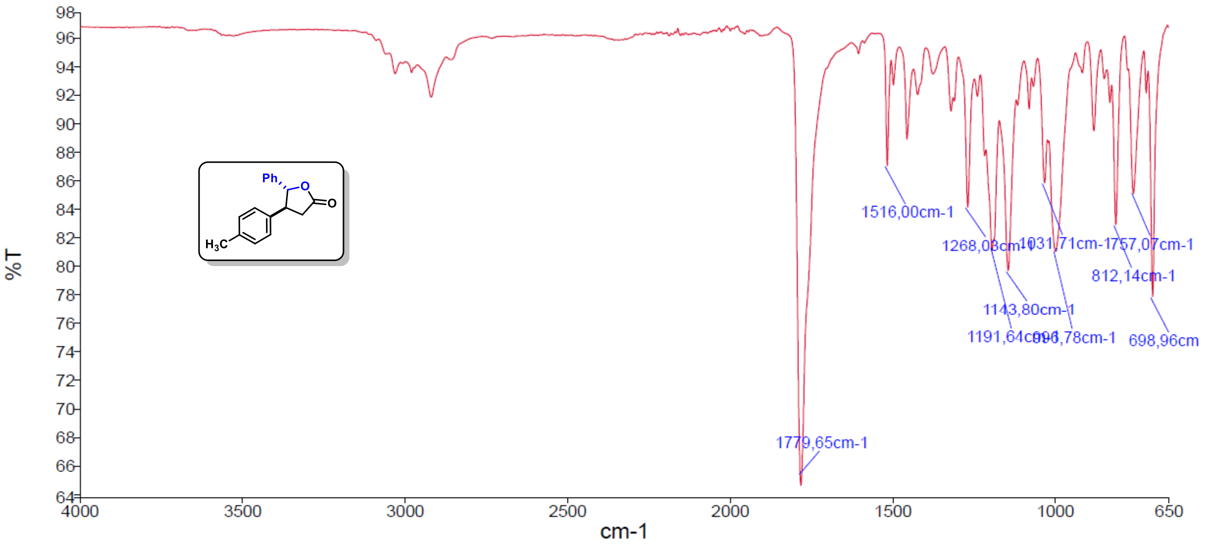

FT-IR Spectrum (ATR, thin film) of ***anti*-2g**.

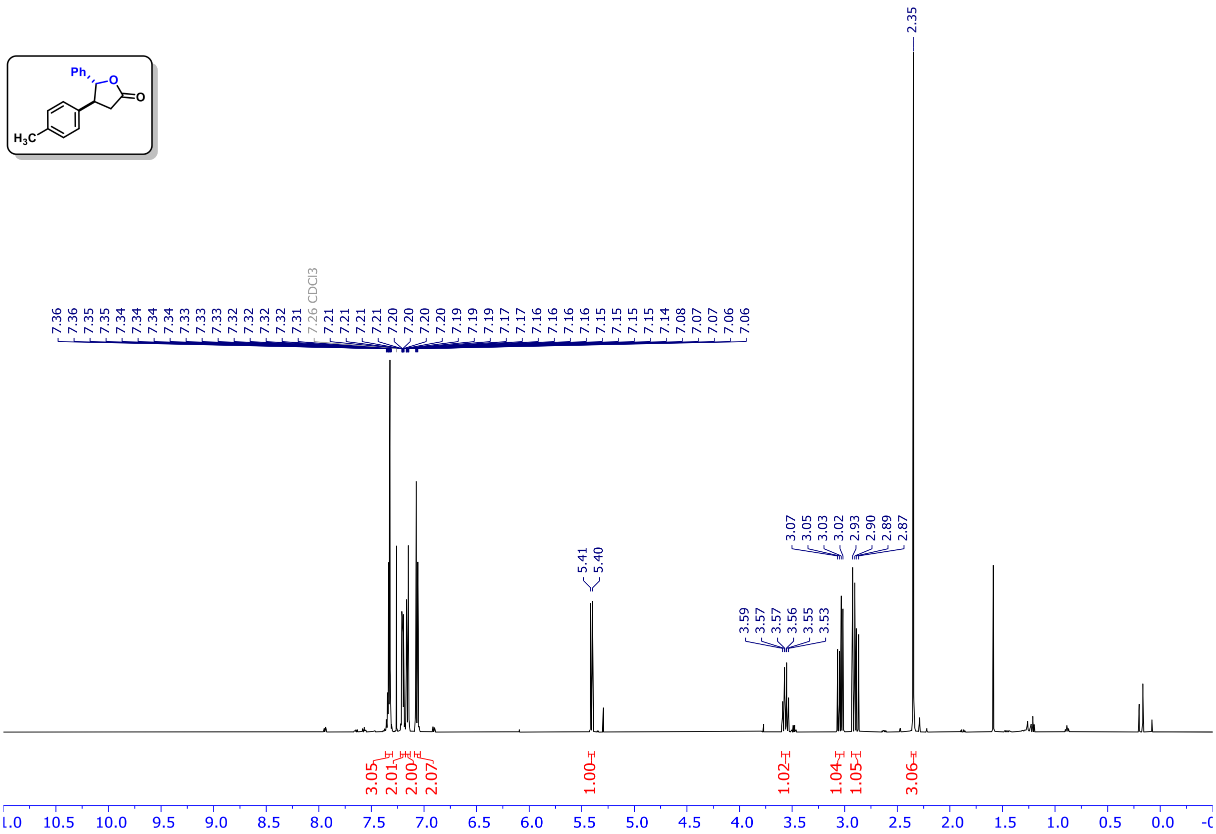

^{1}H-NMR spectrum (500 MHz, $CDCl_3$) of ***anti*-2g**.

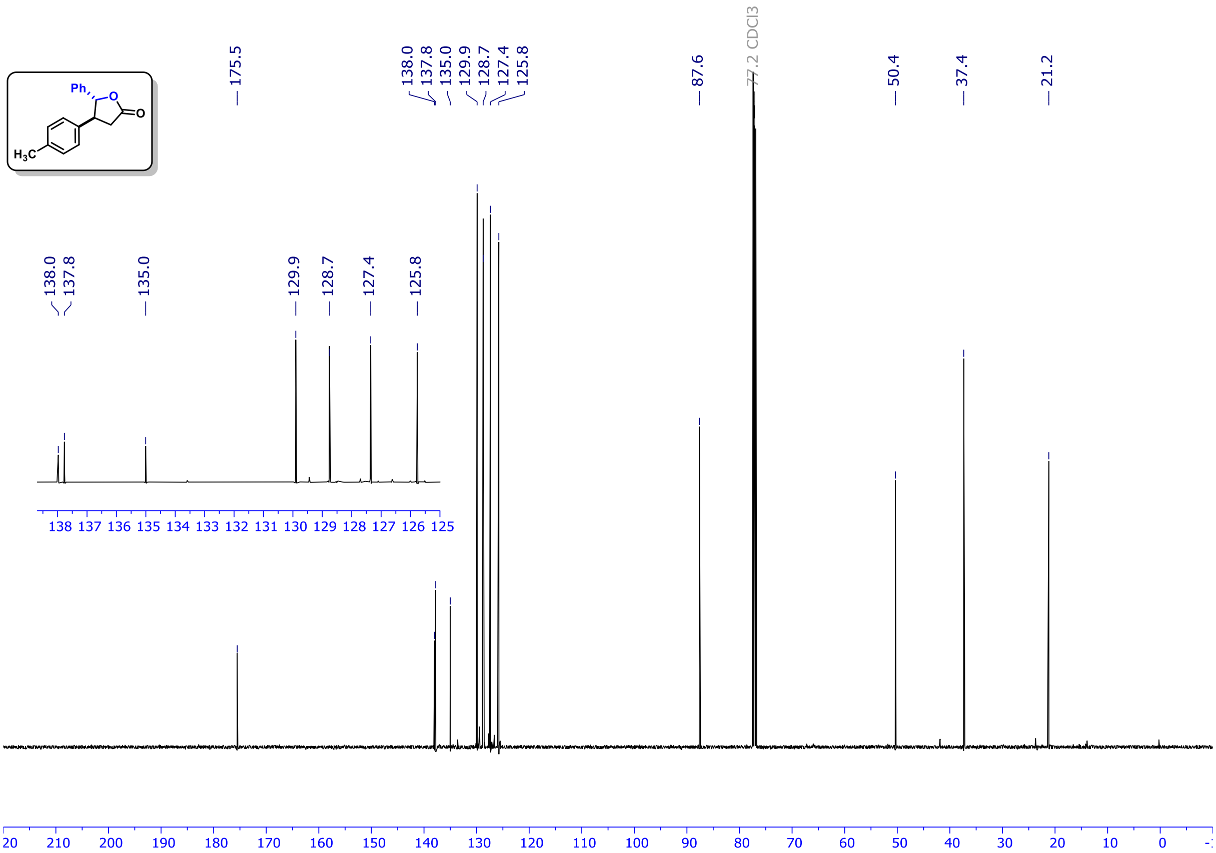

^{13}C-NMR spectrum (126 MHz, $CDCl_3$) of ***anti*-2g**.

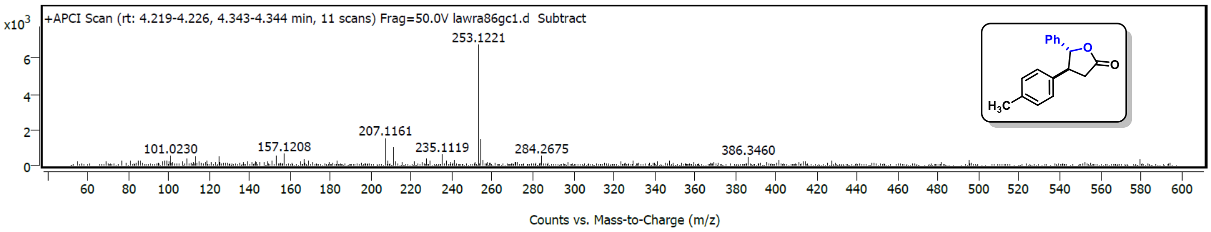

HR-MS Spectrum (APCI,+) of ***anti*-2g**.

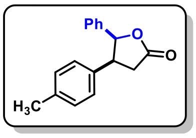

FTIR (ATR): $\tilde{\nu}$ [cm^{-1}] = 1776, 1517, 1170, 1141, 1031, 1012, 979, 822, 747, 698.

^{1}H-NMR (700 MHz, $CDCl_3$): δ_H (ppm) = 7.17–7.11 (m, 3H), 6.95–6.87 (m, 4H), 6.73–6.65 (m, 2H), 5.80 (d, J = 6.8 Hz, 1H), 4.03 (dt, J = 8.2, 6.8 Hz, 1H), 3.01 (dd, J = 17.4, 8.2 Hz, 1H), 2.91 (dd, J = 17.4, 6.8 Hz, 1H), 2.22 (s, 3H).

^{13}C-NMR (176 MHz, $CDCl_3$): δ_C (ppm) = 177.0, 137.2, 135.8, 133.6, 129.1, 128.1, 128.0, 127.9, 125.9, 84.9, 46.7, 35.2, 21.1.

HR-MS (GC-APCI, +, Q-TOF): calc. for $C_{17}H_{17}O_2$ [M+H]$^+$: 253.1223, found: 253.1223.

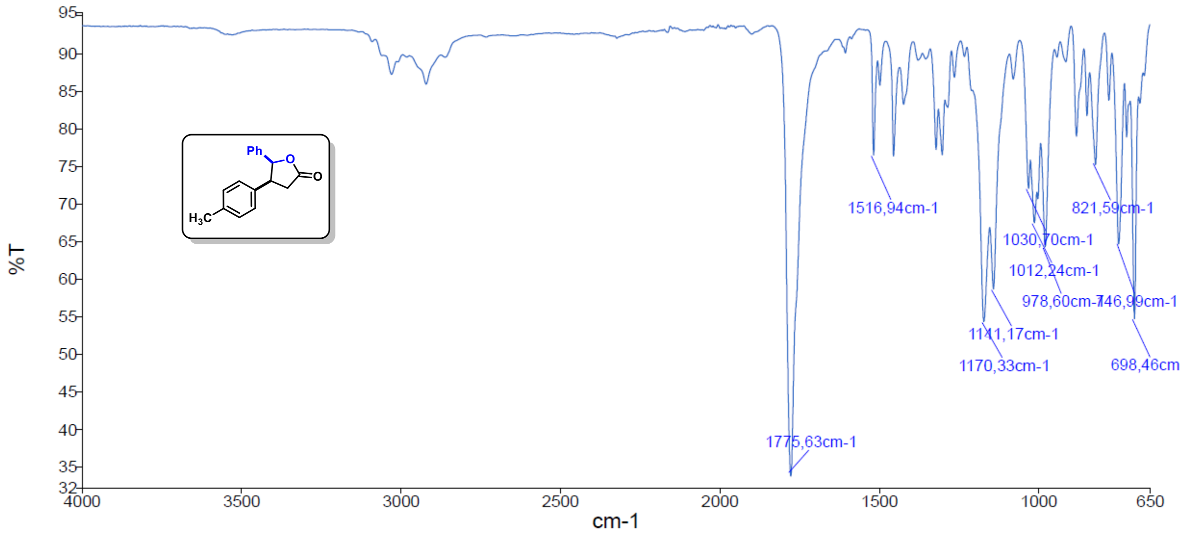

FT-IR Spectrum (ATR, thin film) of ***syn*-2g**.

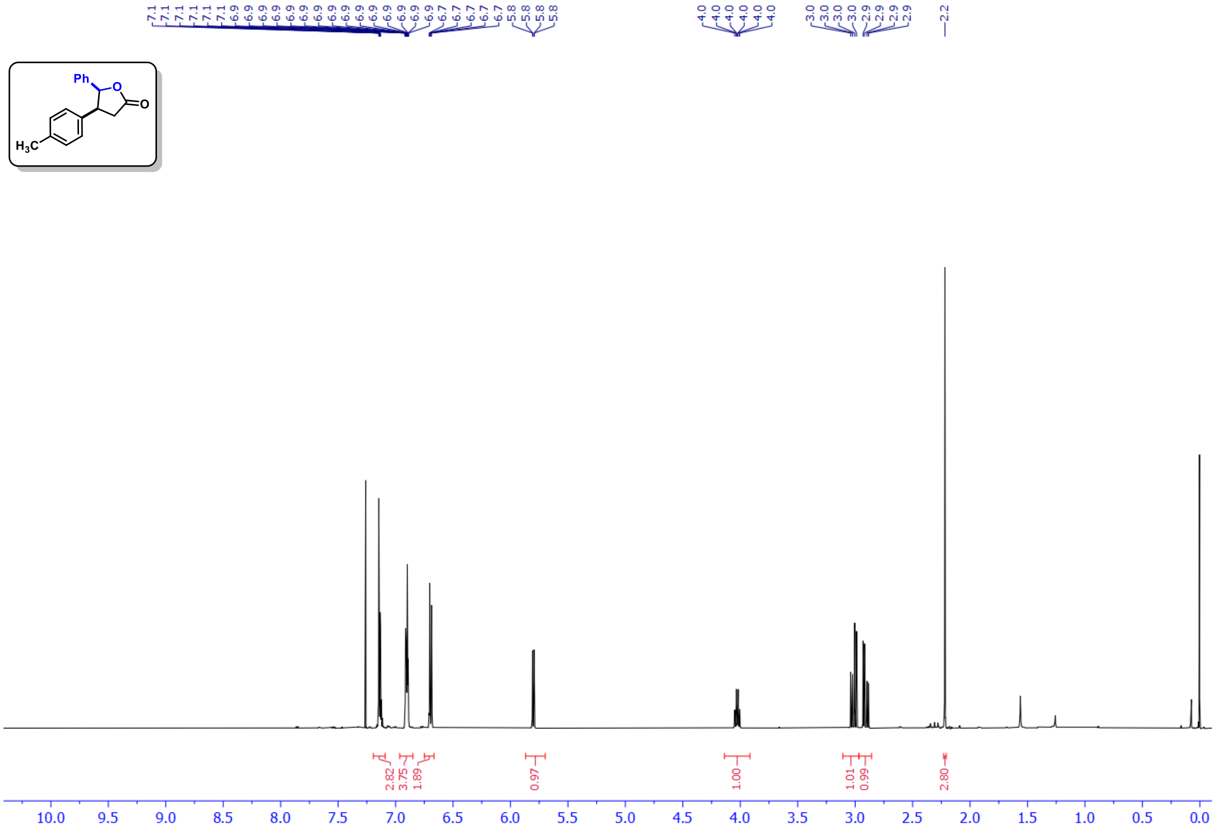

^{1}H-NMR spectrum (500 MHz, $CDCl_3$) of ***syn*-2g**.

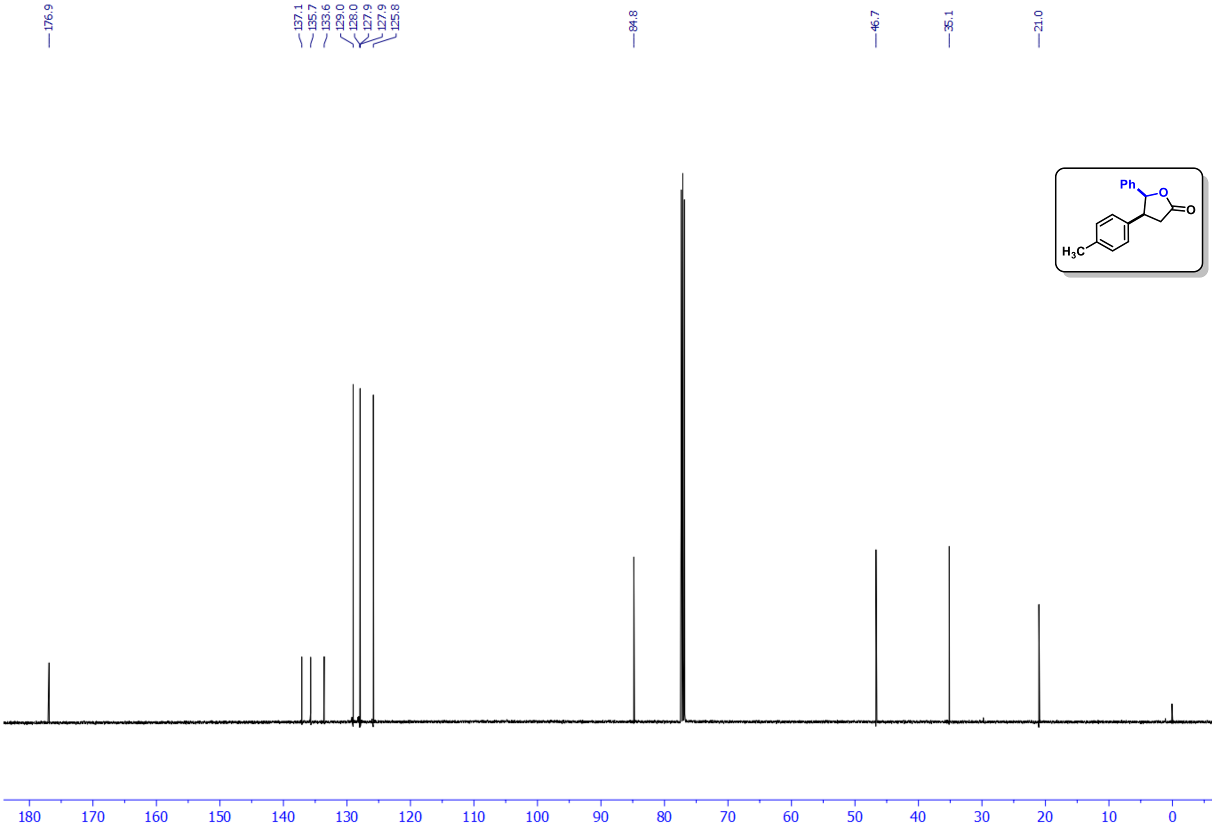

^{13}C-NMR spectrum (126 MHz, $CDCl_3$) of ***syn*-2g**.

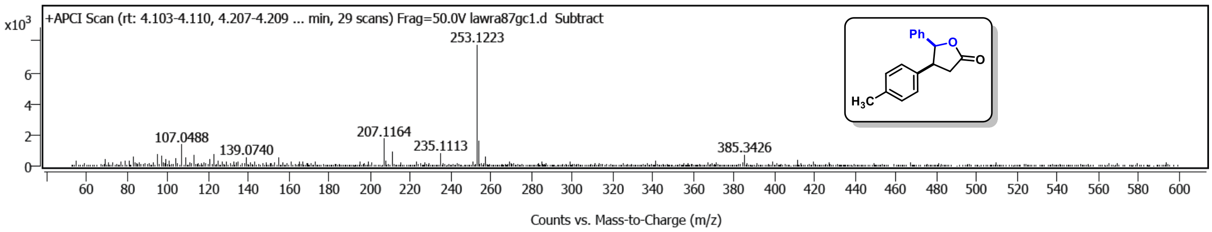

HR-MS Spectrum (APCI,+) of ***syn*-2g**.

Lactone 2h

Prepared according to the **General Procedure** from SCP **1c** (45.7 mg, 0.15 mmol, 1.00 eq.) and 4-chlorobenzaldehyde (169 mg, 1.20 mmol, 8.00 eq.), LiHMDS (1 M in THF, 0.15 mmol, 0.15 mL, 1.00 eq.) and TBSOTf (0.04 M in THF, 5.9 mg, 0.02 mmol, 0.60 mL, 0.15 eq.) in THF (3.15 mL) and DMF (3.75 mL). Purification by flash column chromatography (SiO_2, *n*-pentane/EtOAc 20:1 to 10:1 to 5:1) afforded the lactones ***anti*-2h** (10.9 mg, 0.04 mmol, 24%) and ***syn*-2h** (11.4 mg, 0.04 mmol, 25%) as colorless solids (overall yield: 49%, *dr* 1:1).

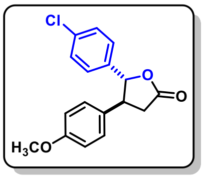

FTIR (ATR): $\tilde{\nu}$ [cm^{-1}] = 1784, 1515, 1494, 1253, 1184, 1146, 1090, 1034, 1003, 826.

^{1}H-NMR (400 MHz, $CDCl_3$): δ_H (ppm) = 7.32–7.28 (m, 2H), 7.14–7.05 (m, 4H), 6.90–6.86 (m, 2H), 5.32 (d, *J* = 8.7 Hz, 1H), 3.81 (s, 3H), 3.47 (dt, *J* = 11.3, 8.7 Hz, 1H), 3.02 (dd, *J* = 17.5, 8.7 Hz, 1H), 2.89 (dd, *J* = 17.5, 11.3 Hz, 1H).

^{13}C-NMR (101 MHz, $CDCl_3$): δ_C (ppm) = 175.1, 159.5, 136.4, 134.7, 129.2, 129.0, 128.6, 127.1, 114.7, 87.0, 55.5, 50.4, 37.4.

HR-MS (APCI, +, Orbitrap): calc. for $C_{17}H_{16}O_3Cl$ $[M+H]^+$: 303.0782, found: 303.0779.

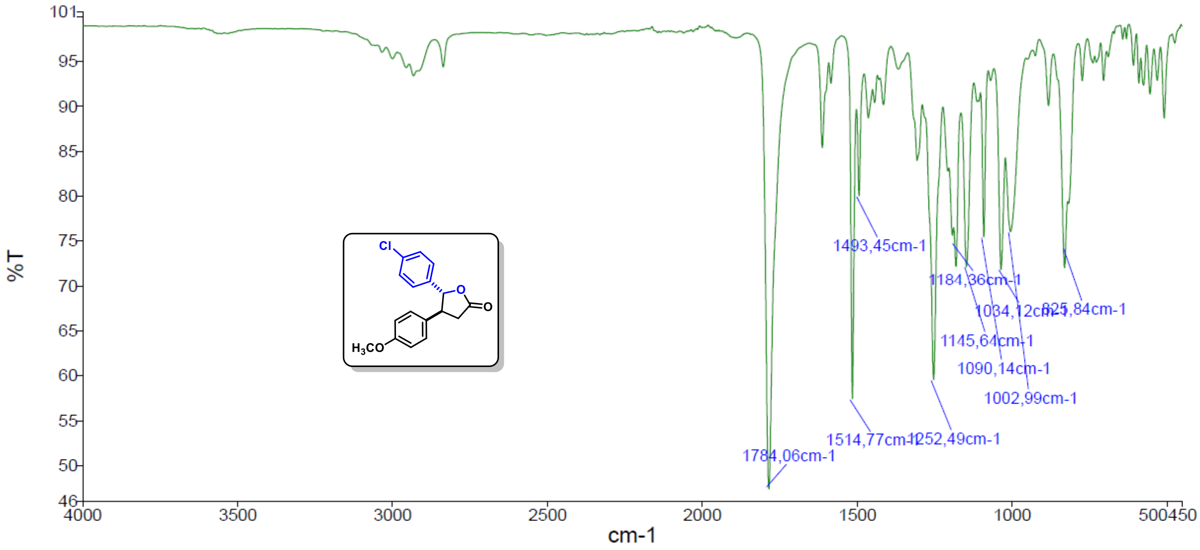

FT-IR Spectrum (ATR, thin film) of ***anti*-2h**.

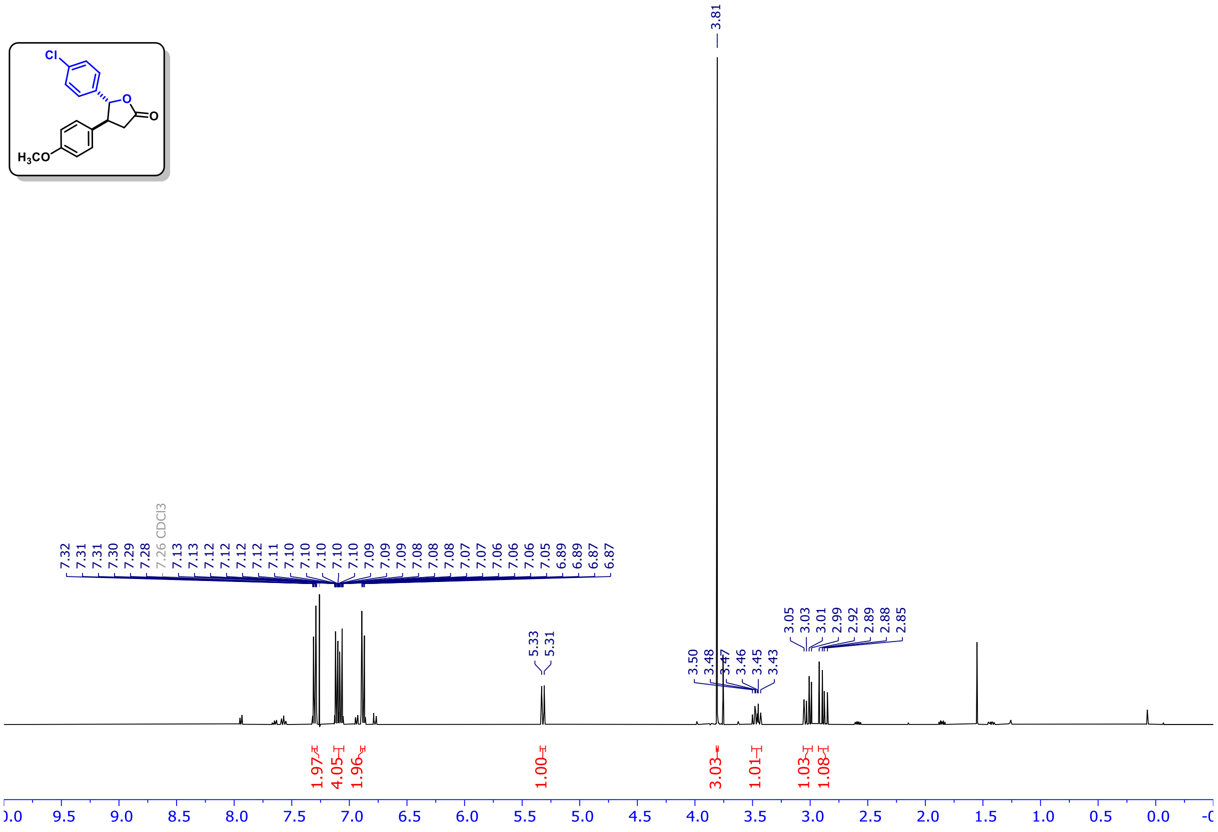

^{1}H-NMR spectrum (400 MHz, $CDCl_3$) of ***anti*-2h**.

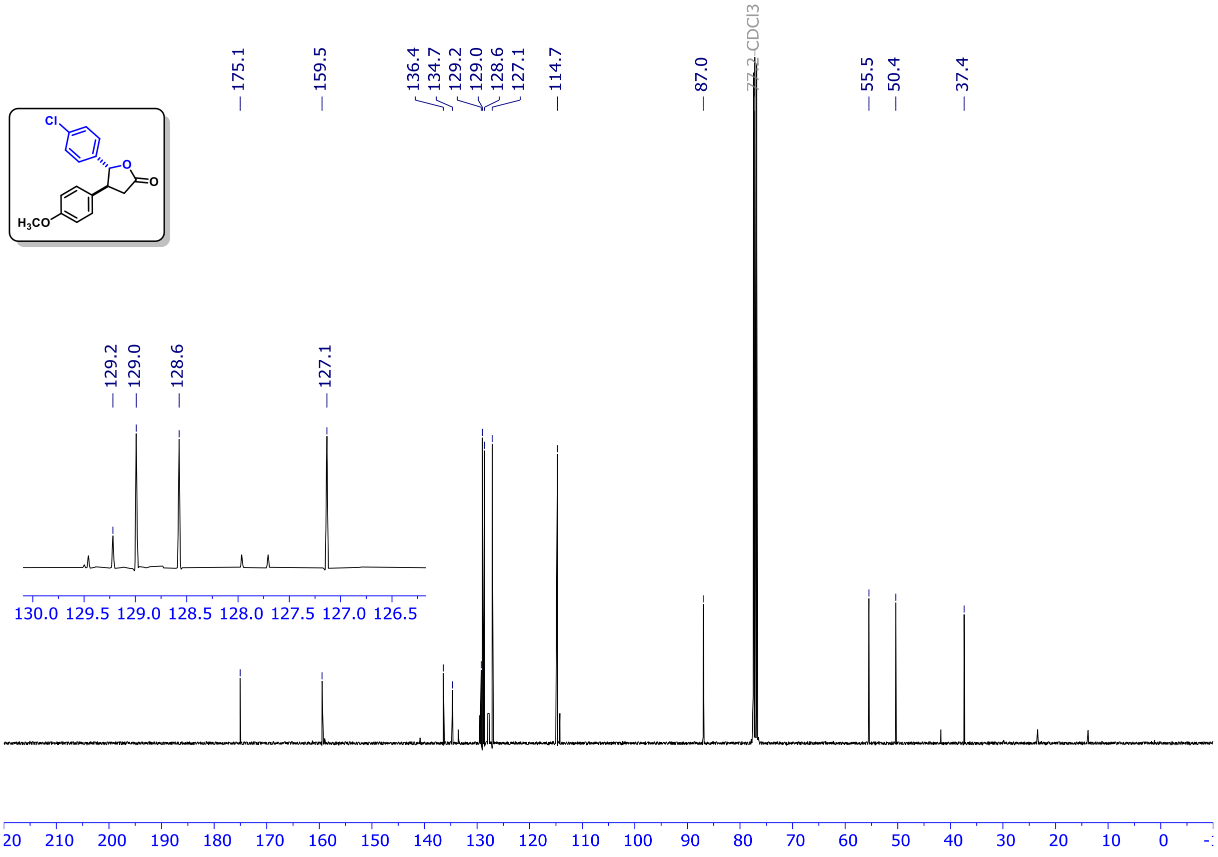

^{13}C-NMR spectrum (101 MHz, $CDCl_3$) of ***anti*-2h**.

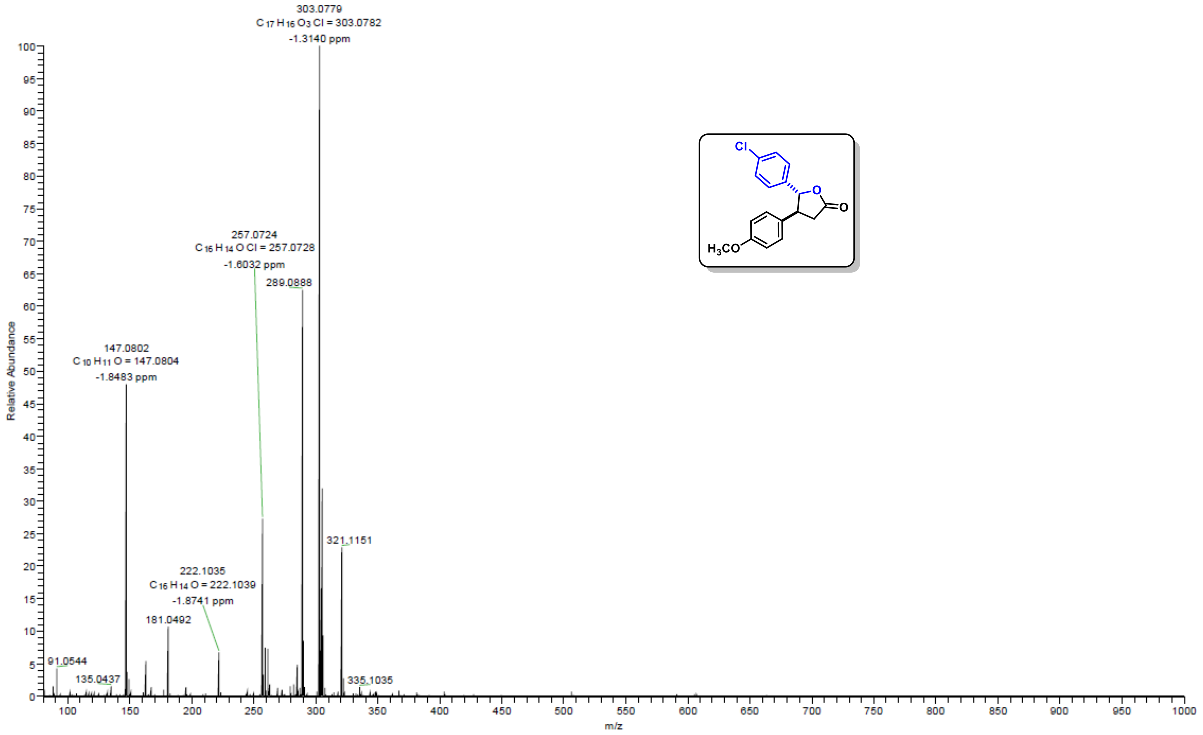

HR-MS Spectrum (APCI,+) of ***anti*-2h**.

FTIR (ATR): $\tilde{\nu}$ [cm^{-1}] = 1783, 1515, 1494, 1252, 1176, 1091, 1033, 1012, 982, 784.

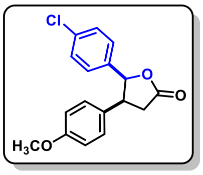

^{1}H-NMR (400 MHz, $CDCl_3$): δ_H (ppm) = 7.15–7.09 (m, 2H), 6.87–6.81 (m, 2H), 6.75–6.71 (m, 2H), 6.69–6.63 (m, 2H), 5.75 (d, J = 6.5 Hz, 1H), 3.99 (dt, J = 8.2, 6.5 Hz, 1H), 3.73 (s, 3H), 3.03 (dd, J = 17.4, 8.2 Hz, 1H), 2.86 (dd, J = 17.4, 6.5 Hz, 1H).

^{13}C-NMR (101 MHz, $CDCl_3$): δ_C (ppm) = 176.6, 159.1, 134.4, 133.9, 129.1, 128.5, 128.3, 127.3, 114.0, 84.3, 55.4, 46.3, 35.4.

HR-MS (APCI, +, Orbitrap): calc. for $C_{17}H_{16}O_3Cl$ $[M+H]^+$: 303.0782, found: 303.0779.

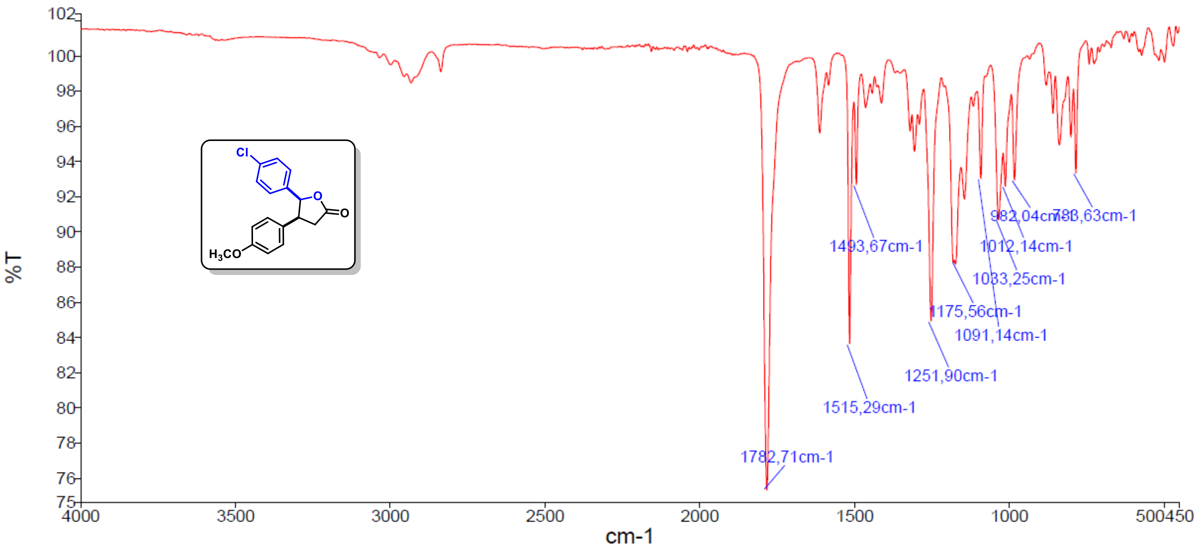

FT-IR Spectrum (ATR, thin film) of ***syn*-2h**.

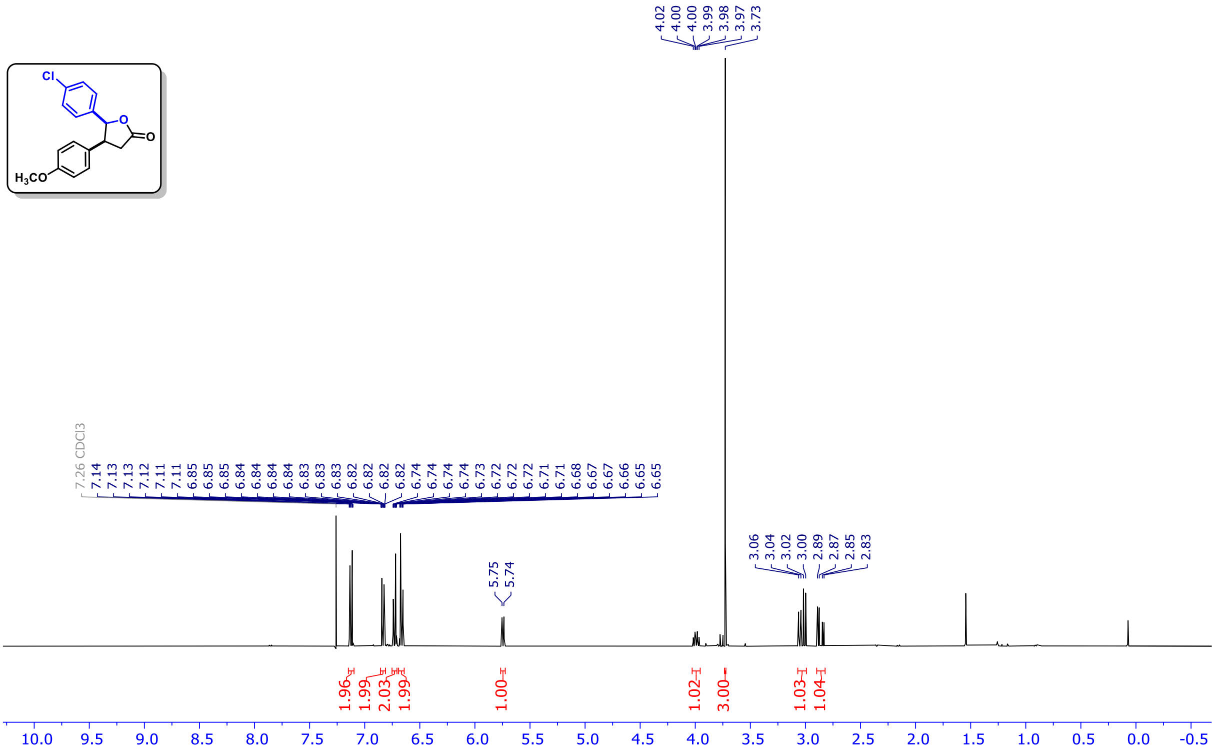

^{1}H-NMR spectrum (400 MHz, CDCl$_3$) of ***syn*-2h**.

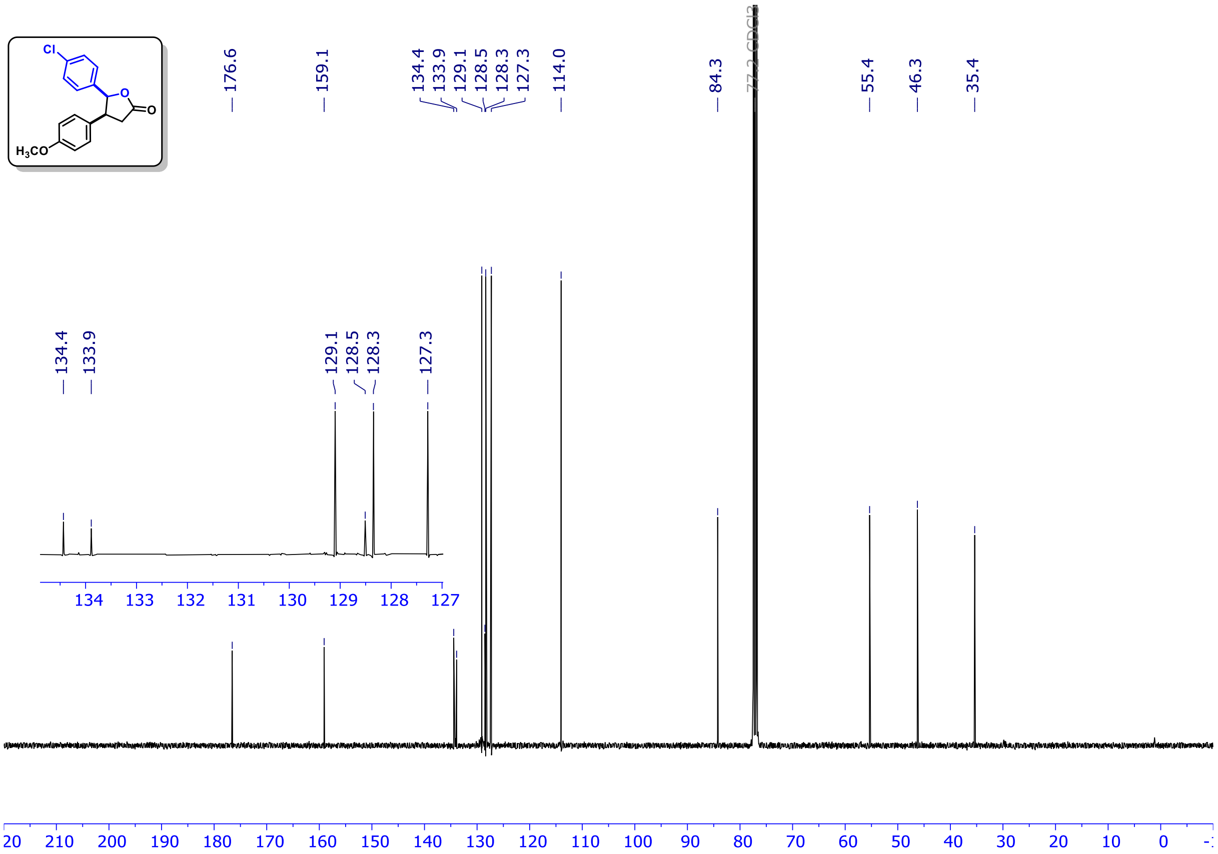

^{13}C-NMR spectrum (101 MHz, $CDCl_3$) of ***syn*-2h**.

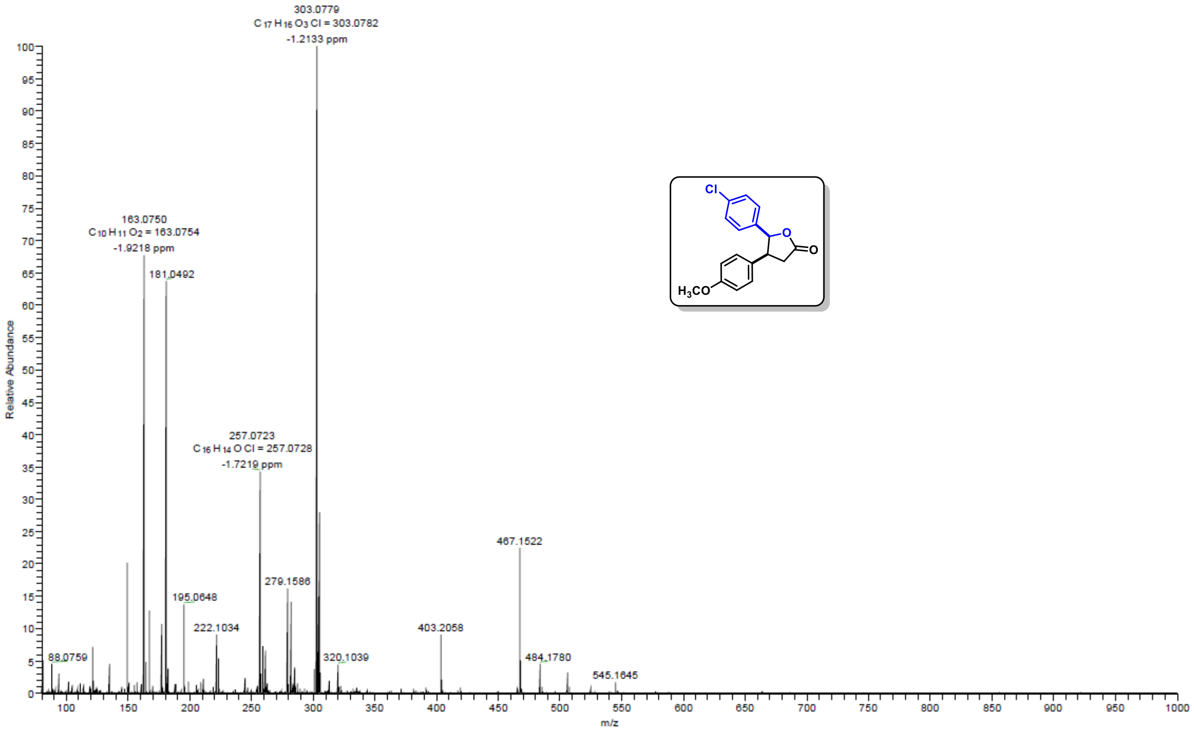

HR-MS Spectrum (APCI,+) of ***syn*-2h**.

Lactone 2i

Prepared according to the **General Procedure** from SCP **1d** (58.5 mg, 0.20 mmol, 1.00 eq.) and benzaldehyde (170 mg, 1.60 mmol, 163 µL, 8.00 eq.). Purification by flash column chromatography (SiO_2, *n*-pentane/EtOAc 7:1 to 2:1) afforded the lactones ***anti*-2i** (19.4 mg, 0.08 mmol, 38%) and ***syn*-2i** (16.6 mg, 0.06 mmol, 32%) as colorless oils (overall yield: 70%, *dr* 1.2:1).

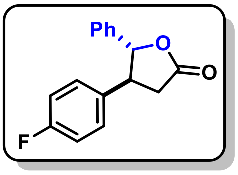

FTIR (ATR): $\tilde{\nu}$ [cm^{-1}] = 1781, 1512, 1225, 1192, 1162, 1145, 996, 832, 759, 699.

^{1}H-NMR (500 MHz, $CDCl_3$): δ_H (ppm) = 7.37–7.31 (m, 3H), 7.21–7.17 (m, 2H), 7.16–7.11 (m, 2H), 7.07–7.01 (m, 2H), 5.36 (d, J = 8.5 Hz, 1H), 3.58 (dt, J = 10.9, 8.5 Hz, 1H), 3.06 (dd, J = 17.6, 8.5 Hz, 1H), 2.88 (dd, J = 17.6, 10.9 Hz, 1H).

^{13}C-NMR (126 MHz, $CDCl_3$): δ_C (ppm) = 175.1, 162.4 (d, J = 247 Hz), 137.6, 133.7, 129.1 (d, J = 7.6 Hz), 128.9, 128.9, 125.8, 116.2 (d, J = 20.7 Hz), 87.6, 50.1, 37.2.

^{19}F-NMR (471 MHz, $CDCl_3$): δ_F (ppm) = −114.7 (m).

HR-MS (GC-APCI, +, Q-TOF): calc. for $C_{16}H_{14}FO_2$ [M+H]$^+$: 257.0972, found: 257.0972.

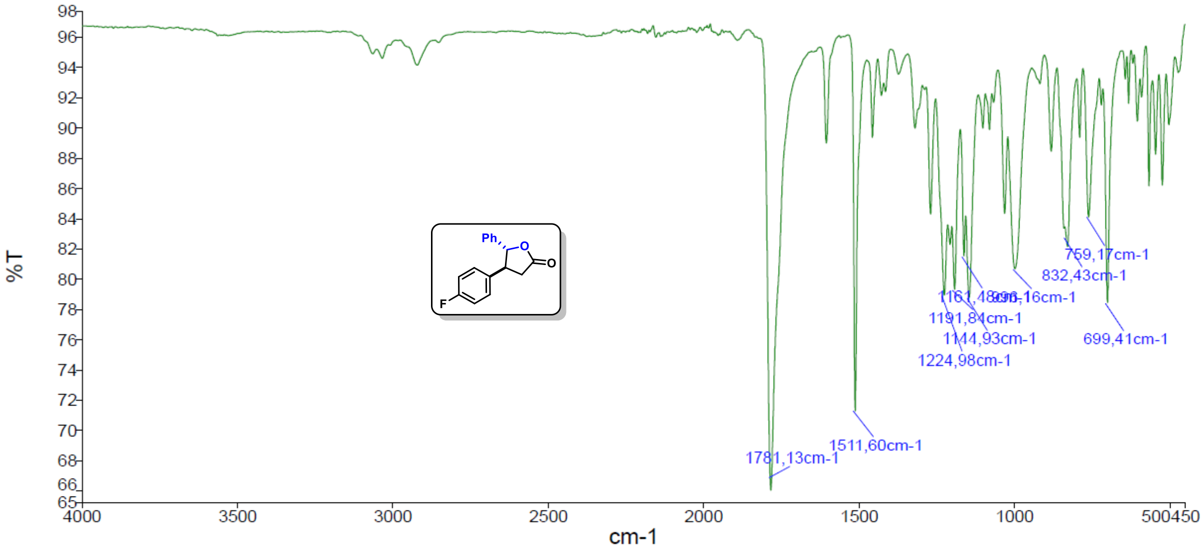

FT-IR Spectrum (ATR, thin film) of ***anti*-2i**.

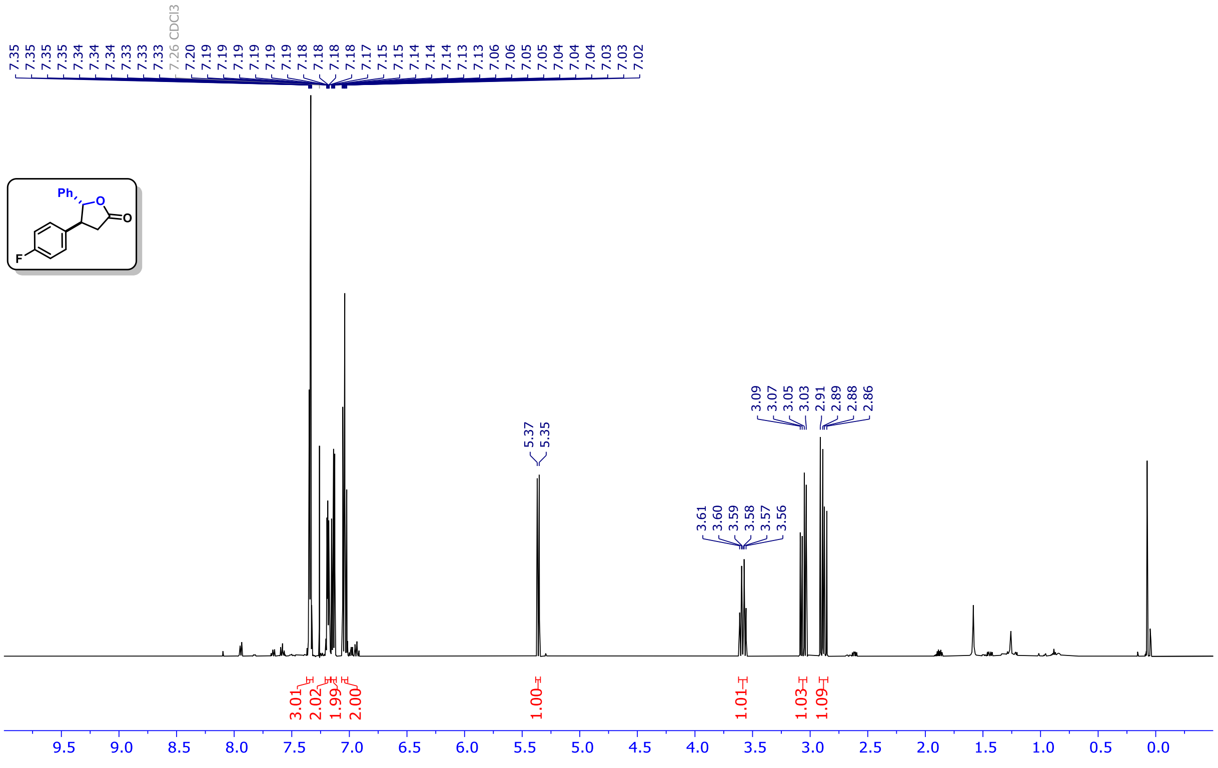

^{1}H-NMR spectrum (500 MHz, $CDCl_3$) of ***anti*-2i**.

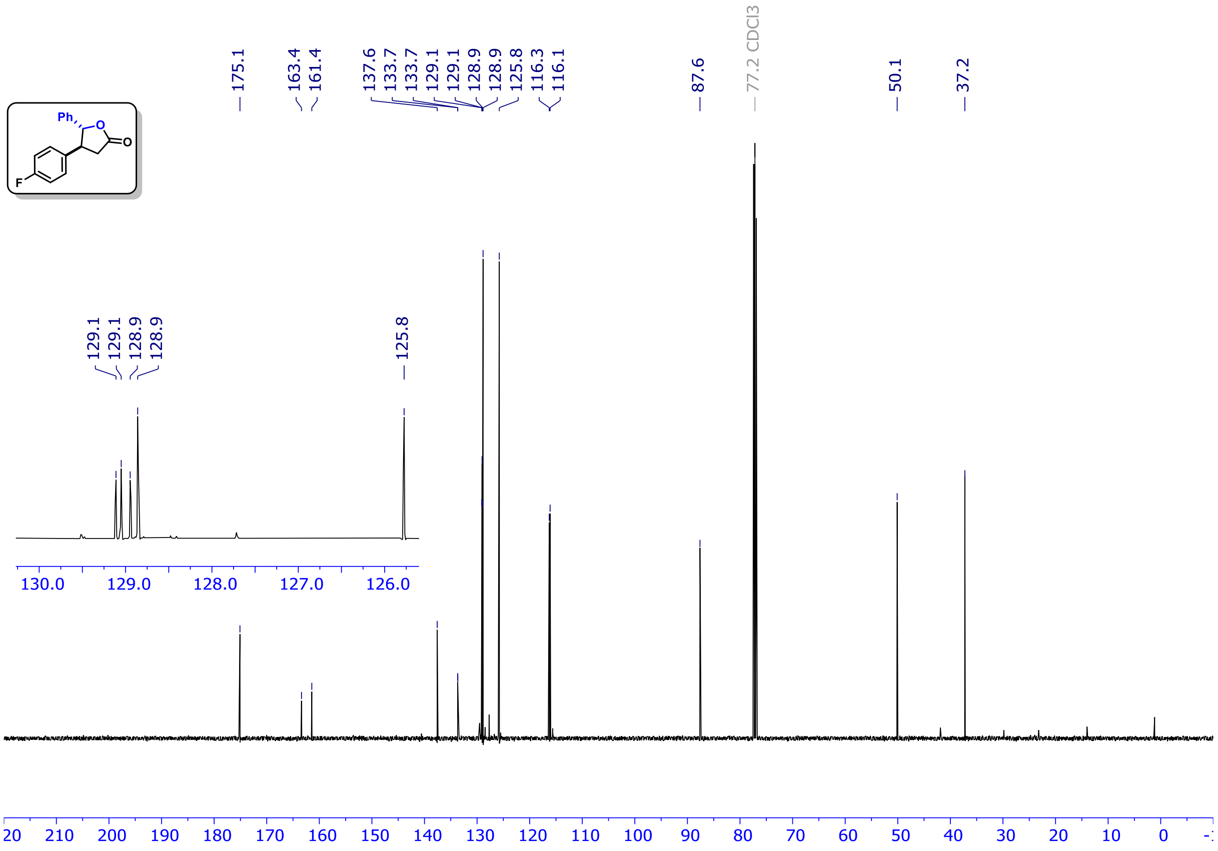

^{13}C-NMR spectrum (126 MHz, $CDCl_3$) of ***anti*-2i**.

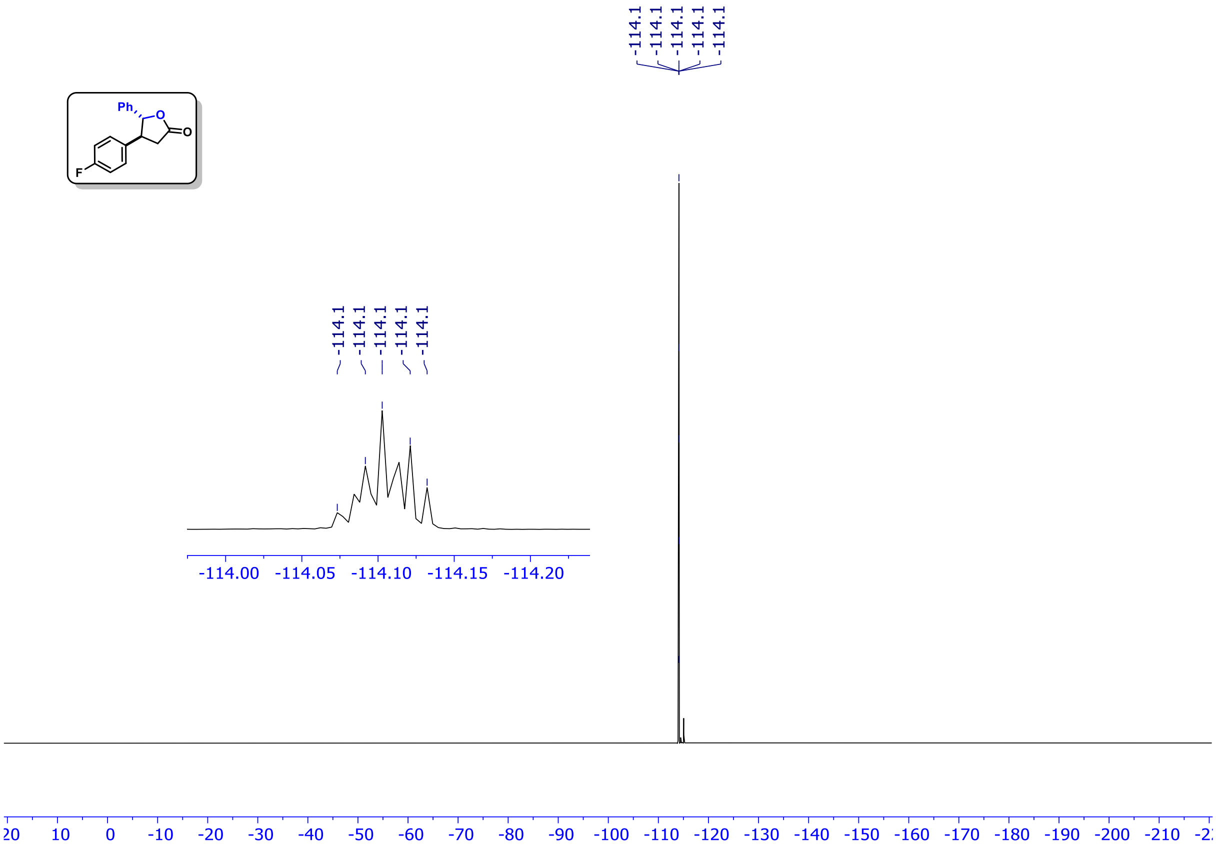

^{19}F-NMR spectrum (471 MHz, $CDCl_3$) of ***anti*-2i**.

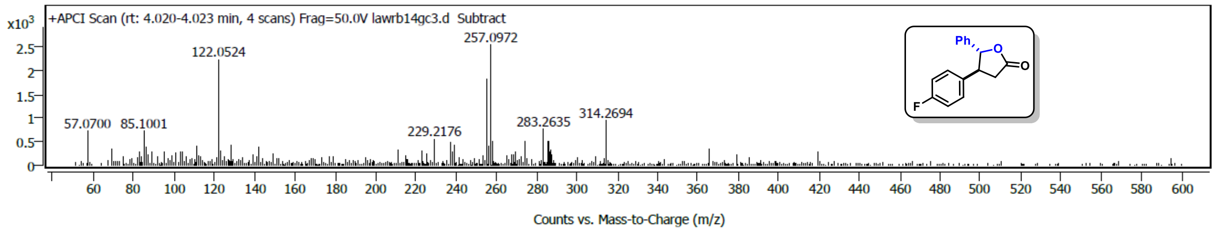

HR-MS Spectrum (APCI,+) of ***anti*-2i**.

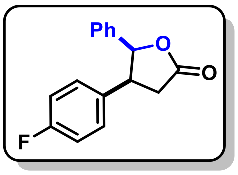

FTIR (ATR): $\tilde{\nu}$ [cm^{-1}] = 1780, 1512, 1228, 1167, 1142, 1014, 982, 838, 750, 700.

^{1}**H-NMR** (500 MHz, $CDCl_3$): δ_H (ppm) = 7.18–7.11 (m, 3H), 6.91–6.86 (m, 2H), 6.83–6.74 (m, 4H), 5.81 (d, J = 6.5 Hz, 1H), 4.04 (dt, J = 8.3, 6.5 Hz, 1H), 3.05 (dd, J = 17.5, 8.3 Hz, 1H), 2.89 (dd, J = 17.5, 6.5 Hz, 1H).

^{13}**C-NMR** (126 MHz, $CDCl_3$): δ_C (ppm) = 176.6, 162.1 (d, J = 246 Hz), 135.5, 132.6 (d, J = 3.2 Hz), 129.6, 129.6, 128.2 (d, J = 8.7 Hz), 125.7, 115.3 (d, J = 20.7 Hz), 84.7, 46.4, 35.3.

^{19}**F-NMR** (471 MHz, $CDCl_3$): δ_F (ppm) = −114.7 (m).

HR-MS (GC-APCI, +, Q-TOF): calc. for $C_{16}H_{14}FO_2$ [M+H]$^+$: 257.0972, found: 257.0972,

calc. for $C_{16}H_{17}FNO_2$ [M+NH$_4$]$^+$: 274.1238, found: 274.1238.

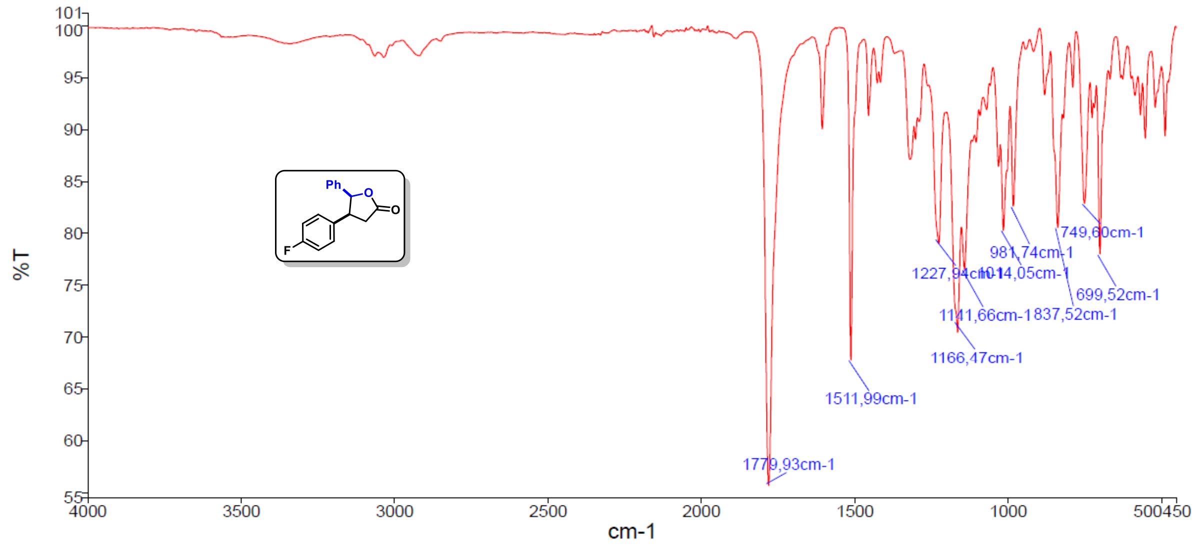

FT-IR Spectrum (ATR, thin film) of ***syn*-2i**.

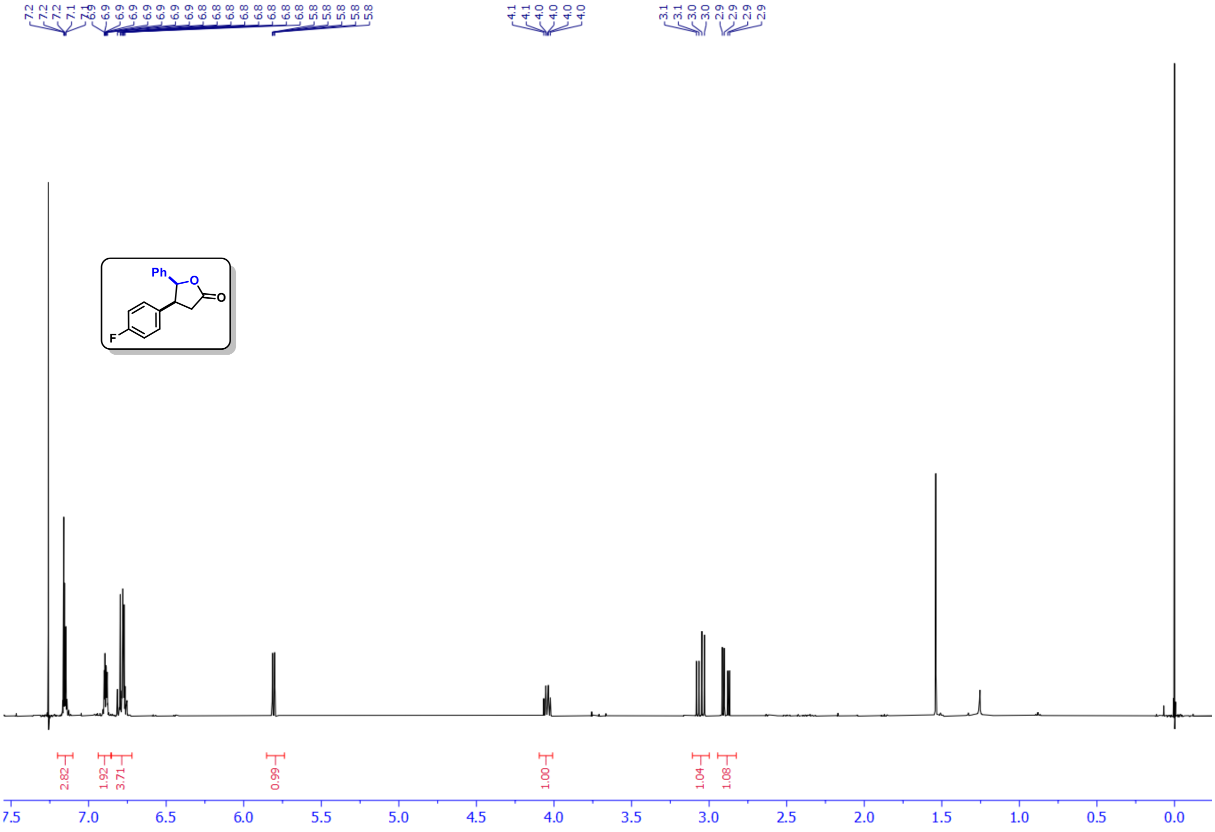

^{1}H-NMR spectrum (500 MHz, $CDCl_3$) of ***syn*-2i**.

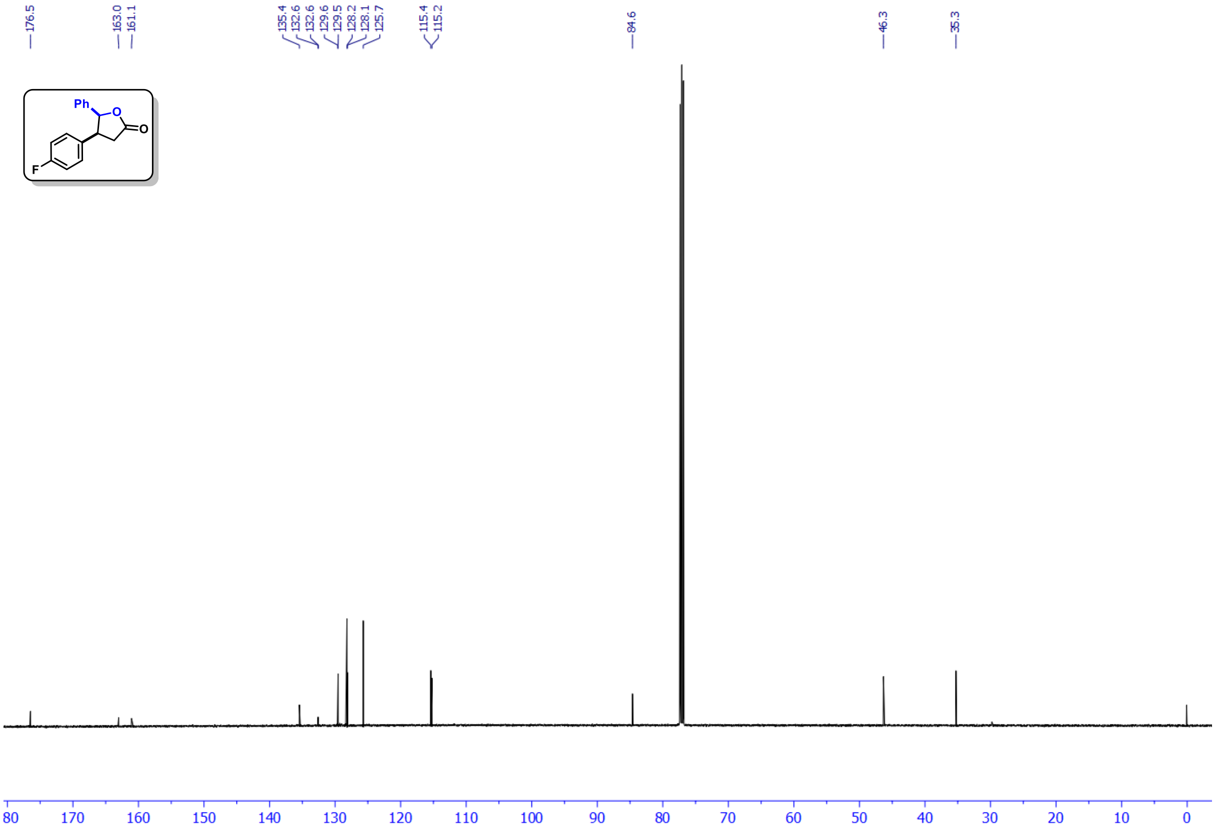

^{13}C-NMR spectrum (126 MHz, $CDCl_3$) of ***syn*-2i**.

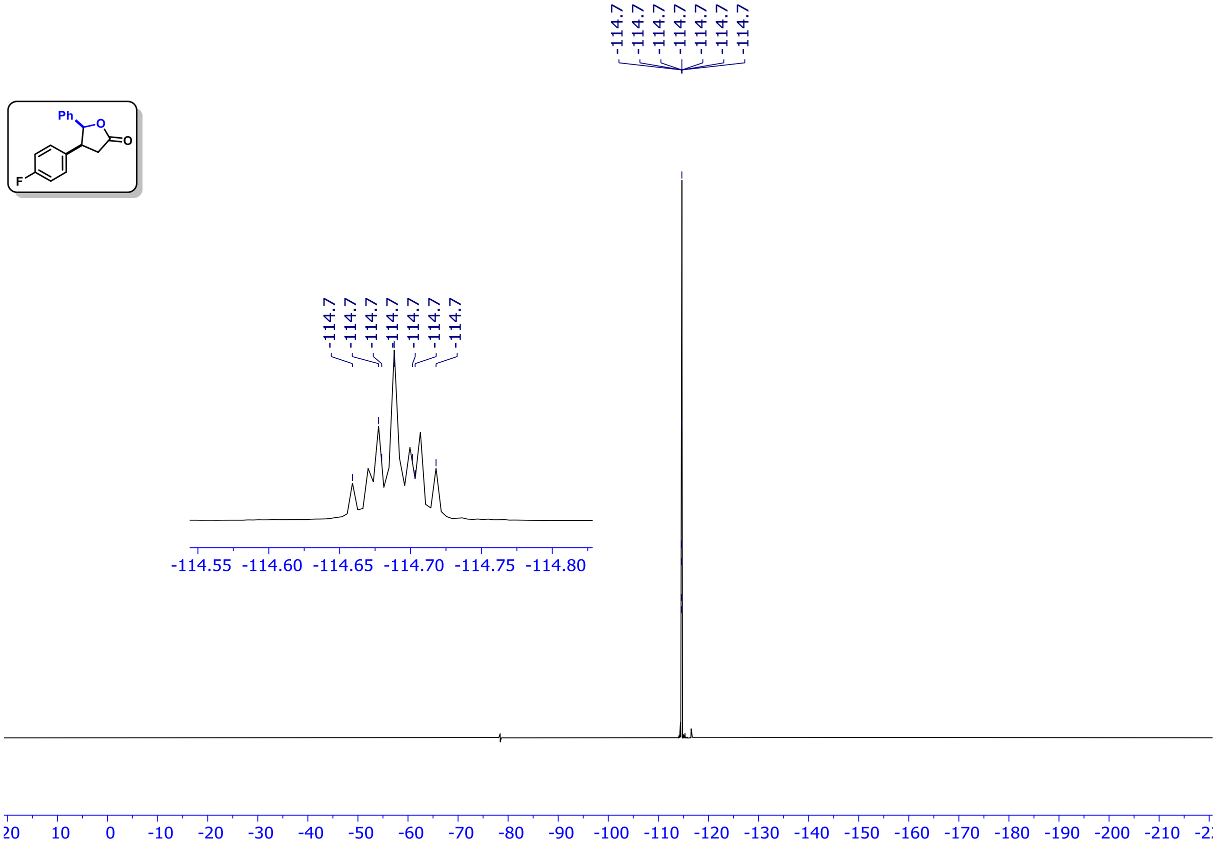

^{19}F-NMR spectrum (471 MHz, $CDCl_3$) of ***syn*-2i**.

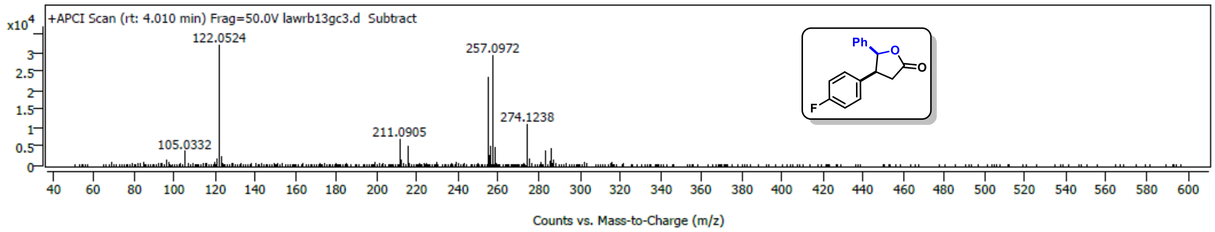

HR-MS Spectrum (APCI,+) of ***syn*-2i**.

Lactam Substrate Scope

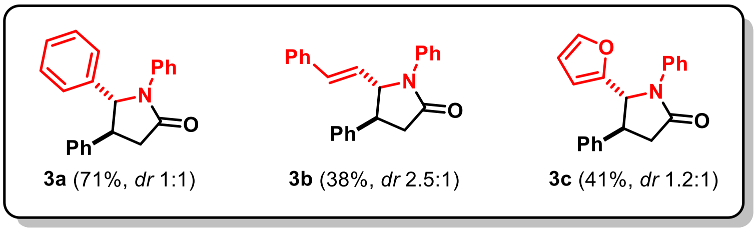

Lactam 3a

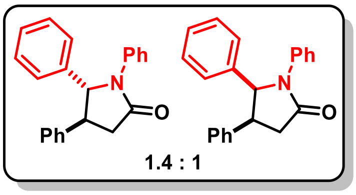

Prepared according to the **General Procedure** from SCP **1a** (54.9 mg, 0.20 mmol, 1.00 eq.) and *N*-diphenyl-methanimine (217 mg, 1.60 mmol, 8.00 eq.). Purification by flash column chromatography (SiO_2, *n*-pentane/EtOAc 8:1 to 2:1) afforded lactam **3a** (44.4 mg, 0.14 mmol, 71%, *anti*/*syn* 1.4:1) as a mixture of two inseparable diastereomers as a pale yellow solid.

FTIR (ATR): $\tilde{\nu}$ [cm^{-1}] = 1694, 1496, 1455, 1379, 1353, 1216, 758, 744, 697, 692.

^{1}H-NMR (700 MHz, acetone-d_6): δ_H (ppm) = 7.74–6.80 (m, 15H), 5.68 (d, J = 8.0 Hz, 1H), 5.41 (d, J = 6.5 Hz, 1H), 4.27 (dt, J = 12.5, 7.9 Hz, 1H), 3.50 (td, J = 8.6, 6.5 Hz, 1H), 3.17 (dd, J = 16.5, 12.5 Hz, 1H), 3.06 (dd, J = 16.9, 8.6 Hz, 1H), 2.80 (dd, J = 16.9, 8.6 Hz, 1H), 2.72 (dd, J = 16.5, 7.9 Hz, 1H). *Signals of the anti-isomer are underlined.*

^{13}C-NMR (176 MHz, acetone-d_6): δ_C (ppm) = 174.1, 173.5, 142.5, 141.3, 140.2, 139.5, 138.4, 138.1, 129.6, 129.5, 129.3, 129.1, 128.9, 128.7, 128.5, 128.3, 128.2, 128.1, 128.0, 127.6, 127.6, 125.3, 124.9, 123.6, 121.9, 71.3, 68.6, 49.4, 44.9, 39.9, 36.2. *Due to overlap, not all aromatic signals are resolved.*

HR-MS (GC-APCI, +, Q-TOF): calc. for $C_{22}H_{19}NO$ $[M+H]^+$: 313.1467, found: 313.1464.

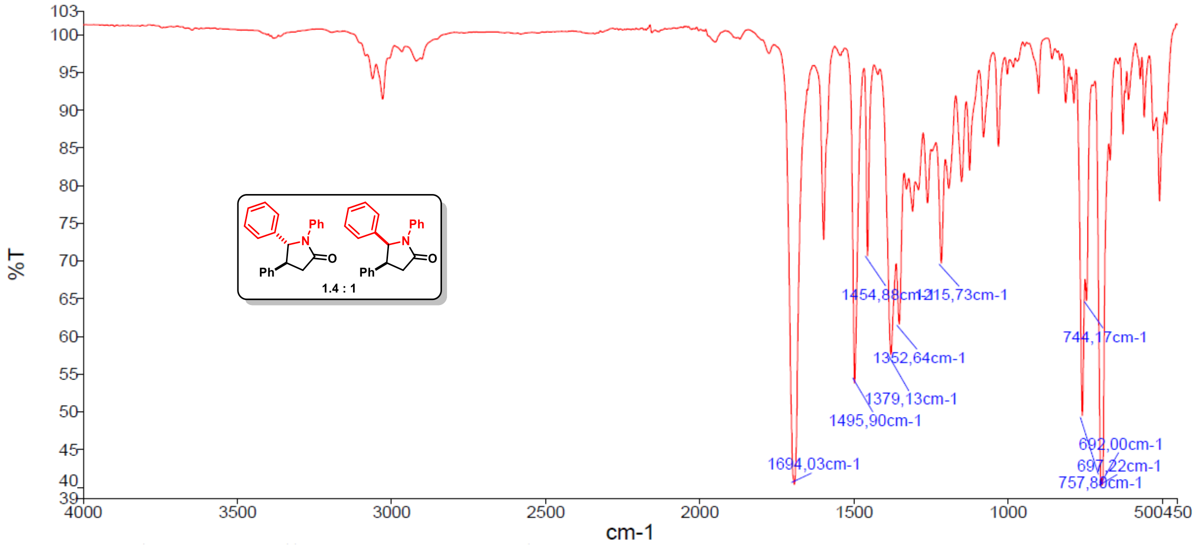

FT-IR Spectrum (ATR, thin film) of **3a** (mixture of *anti*- and *syn*-isomer).

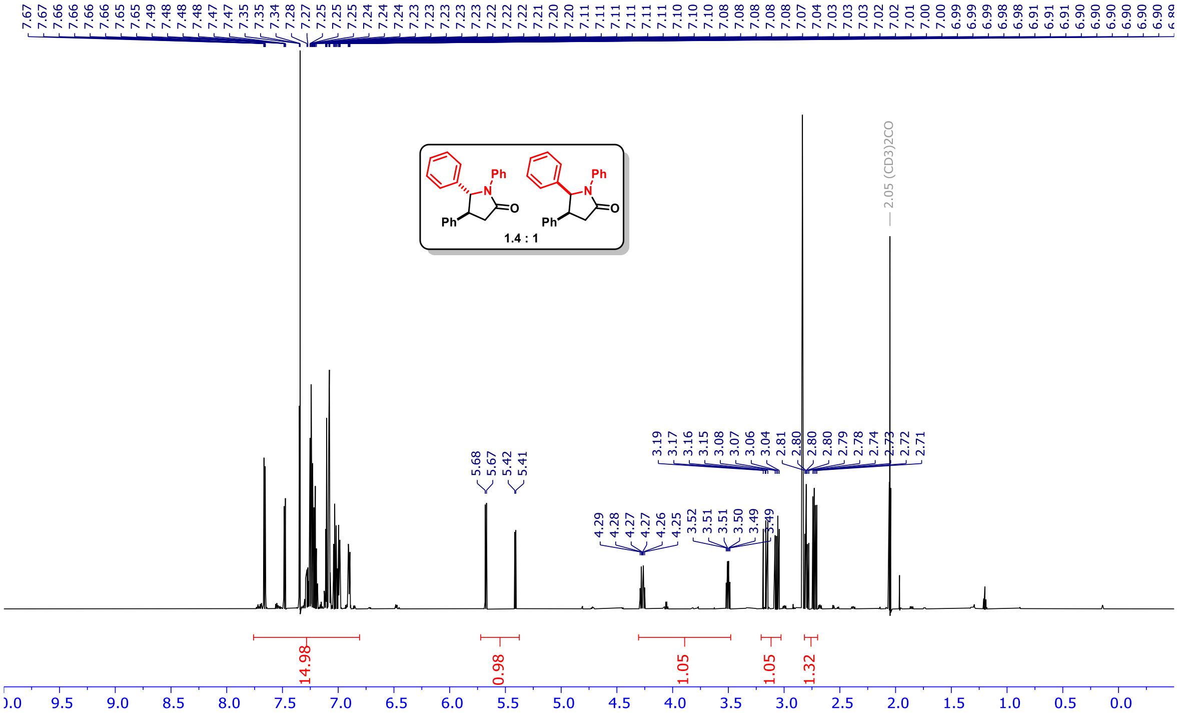

^{1}H-NMR spectrum (700 MHz, acetone-d_6) of **3a** (mixture of *anti*- and *syn*-isomer). Signals of identical protons of both diastereomers are integrated together.

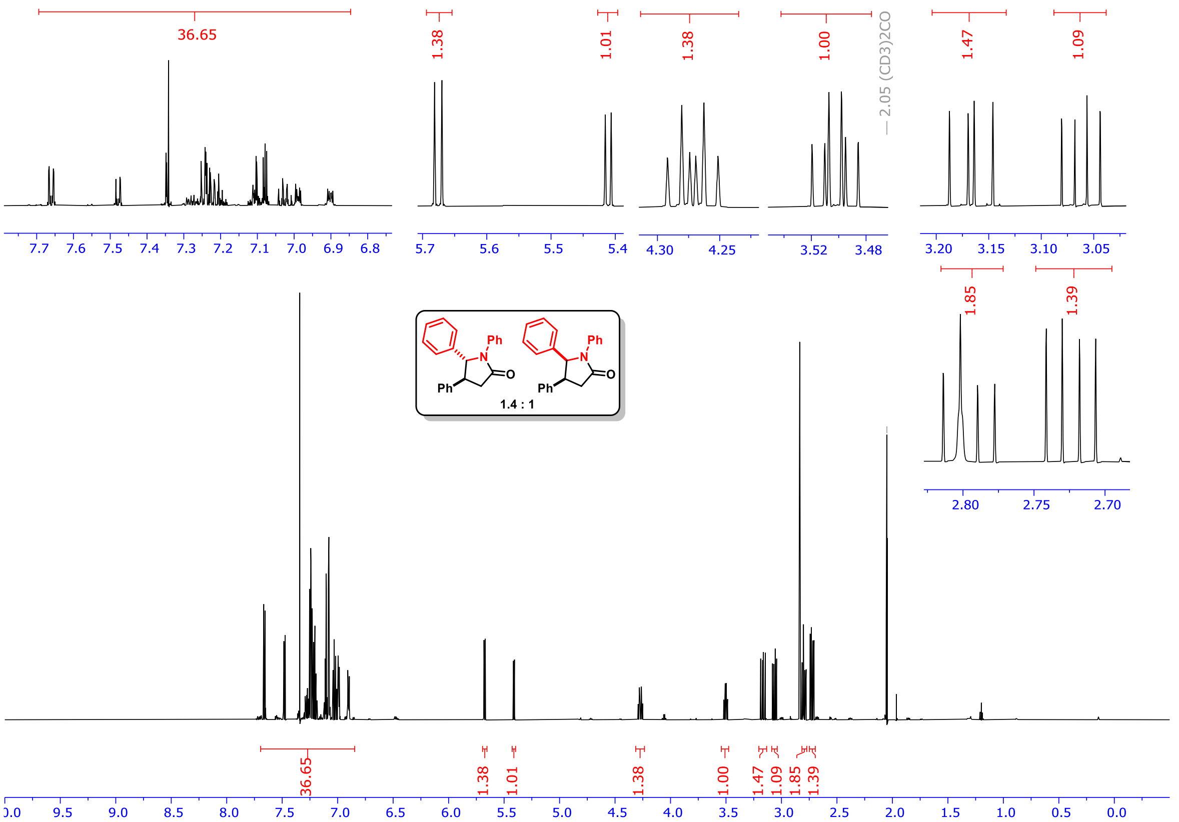

^{1}H-NMR spectrum (700 MHz, acetone-d_6) of **3a**. Signals of both diastereomers are integrated separately.

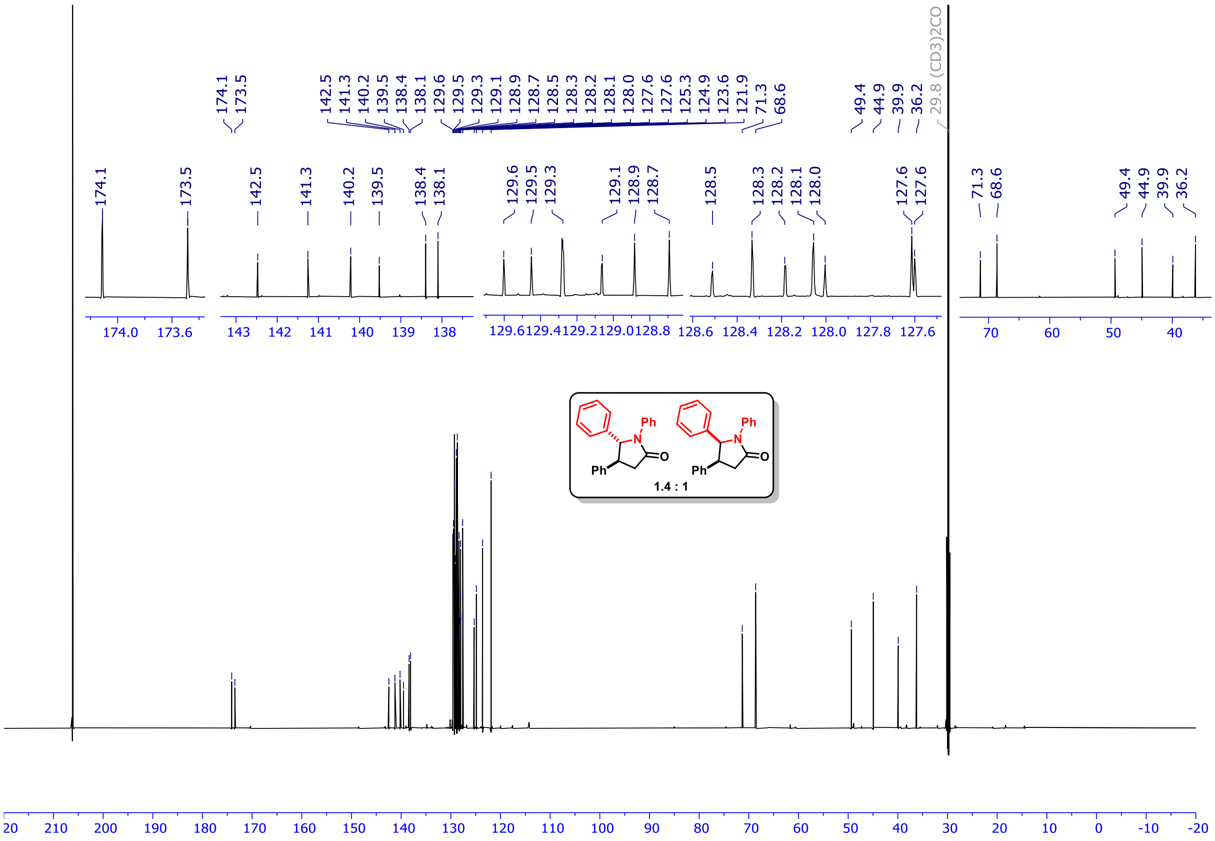

^{13}C-NMR spectrum (176 MHz, acetone-d_6) of **3a** (mixture of *anti-* and *syn*-isomer).

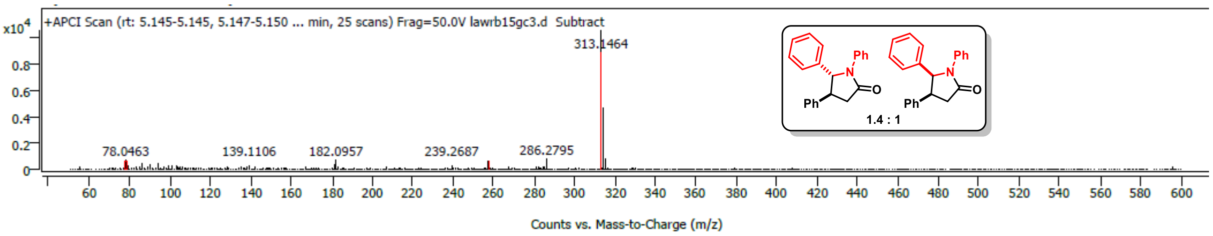

HR-MS Spectrum (APCI,+) of **3a** (mixture of *anti-* and *syn*-isomer).

Lactam 3b

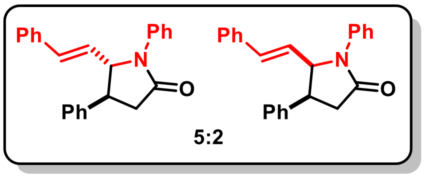

Prepared according to the **General Procedure** from SCP **1a** (54.9 mg, 0.20 mmol, 1.00 eq.) and *trans-N*-diphenylprop-2-en-1-imine (332 mg, 1.60 mmol, 8.00 eq.). Purification by flash column chromatography (SiO_2, *n*-pentane/acetone 10:1) afforded lactam **3b** (26.0 mg, 0.08 mmol, 38%, *anti*/*syn* 5:2) as a mixture of two inseparable diastereomers as a yellow oil.

FTIR (ATR): $\tilde{\nu}$ [cm^{-1}] = 1697, 1597, 1495, 1376, 1269, 1209, 969, 756, 742, 694.

^{1}H-NMR (700 MHz, CD_2Cl_2): δ_H (ppm) = 7.68–7.00 (m, 15H), 6.36* (m, 1H), 6.13 (dd, *J* = 15.9, 8.0 Hz, 1H), <u>5.68</u> (dd, *J* = 16.0, 7.2 Hz, 1H), <u>5.07</u> (dd, *J* = 7.2, 1.2 Hz, 1H), 4.77 (ddd, *J* = 8.2, 6.1, 0.9 Hz, 1H), <u>4.01</u> (dt, *J* = 10.7, 7.7 Hz, 1H), 3.45 (td, *J* = 8.4, 6.1 Hz, 1H),

3.17–2.98* (m, 1H), <u>2.83</u> (dd, J = 16.5, 7.8 Hz, 1H), 2.79 (dd, J = 17.2, 8.0 Hz, 1H). *Signals of the anti-isomer are underlined.* (*) *Signals of anti- and syn-isomer overlap.*

^{13}C-NMR (176 MHz, CD_2Cl_2): δ_C (ppm) = 173.6, 173.2, 141.6, 139.1, 138.5, 138.2, 136.6, 136.3, 133.7, 133.3, 129.3, 129.1, 129.0, 129.0, 128.9, 128.9, 128.7, 128.5, 128.3, 128.2, 127.8, 127.7, 127.7, 126.8, 126.8, 126.2, 125.8, 125.3, 124.0, 122.6, 70.3, 66.5, 45.8, 44.1, 39.3, 36.5. *Due to overlap, not all aromatic signals are resolved.*

HR-MS (ESI+, Orbitrap): calc. for $C_{24}H_{22}ON$ [M+H]$^+$: 340.1696, found: 340.1693.

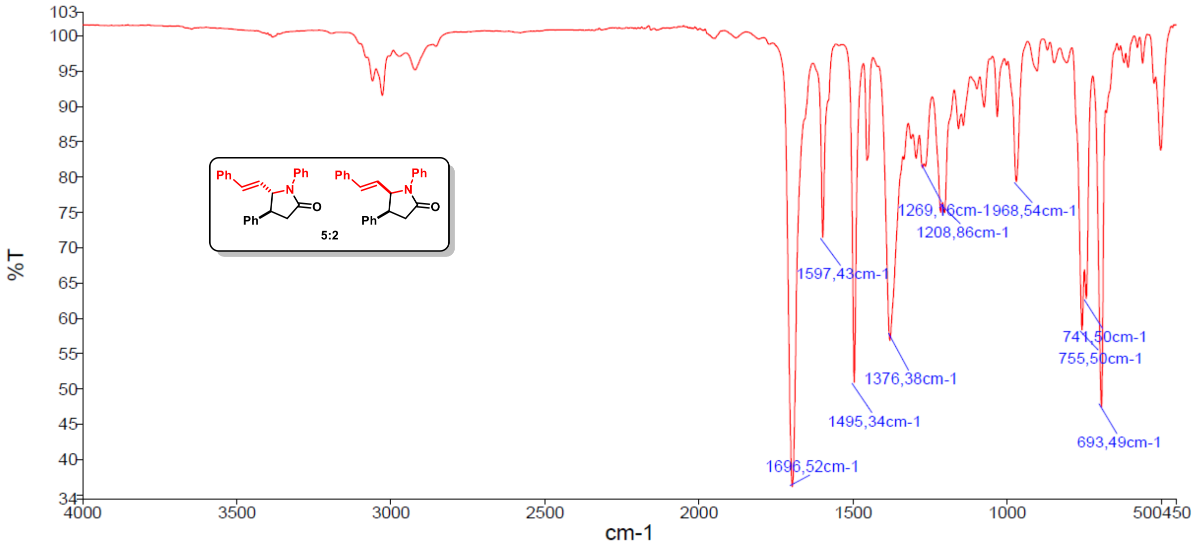

FT-IR Spectrum (ATR, thin film) of **3b** (mixture of *anti*- and *syn*-isomer).

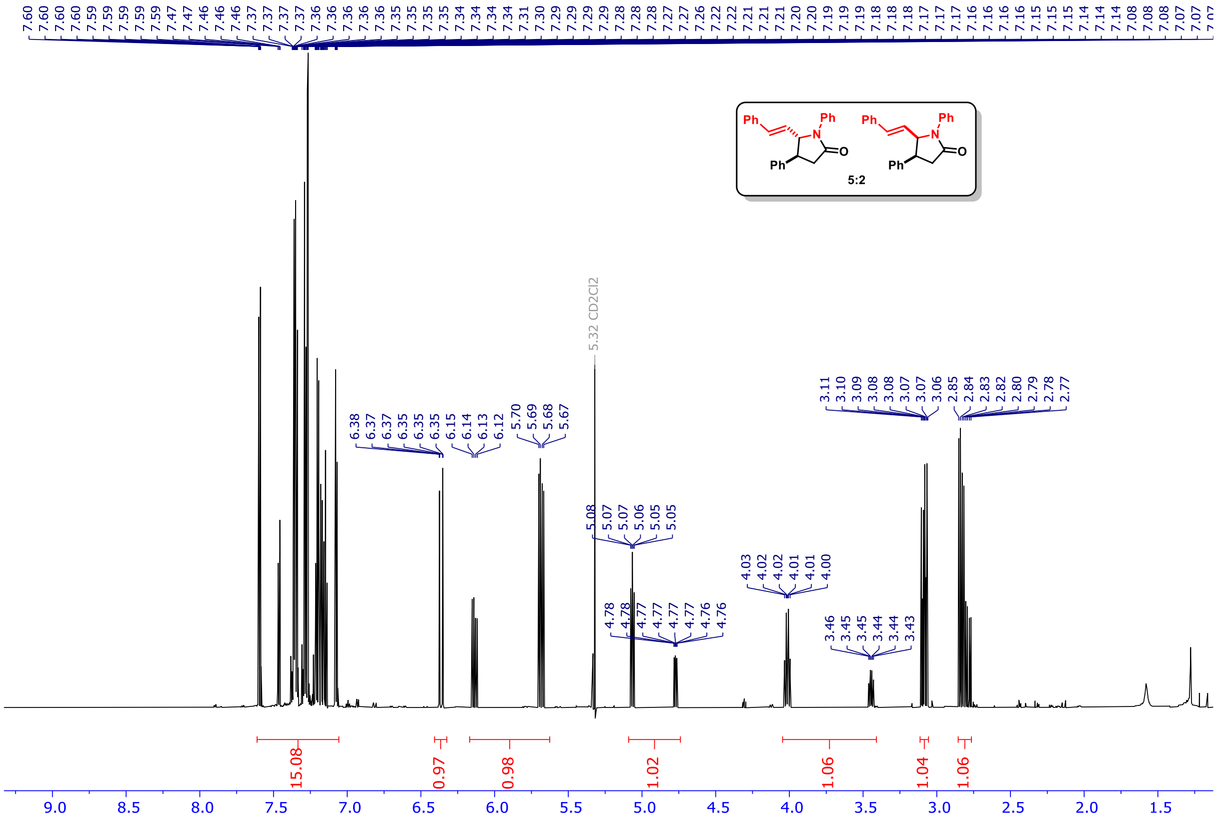

^{1}H-NMR spectrum (700 MHz, CD_2Cl_2) of **3b** (mixture of *anti*- and *syn*-isomer). Signals of identical protons of both diastereomers are integrated together.

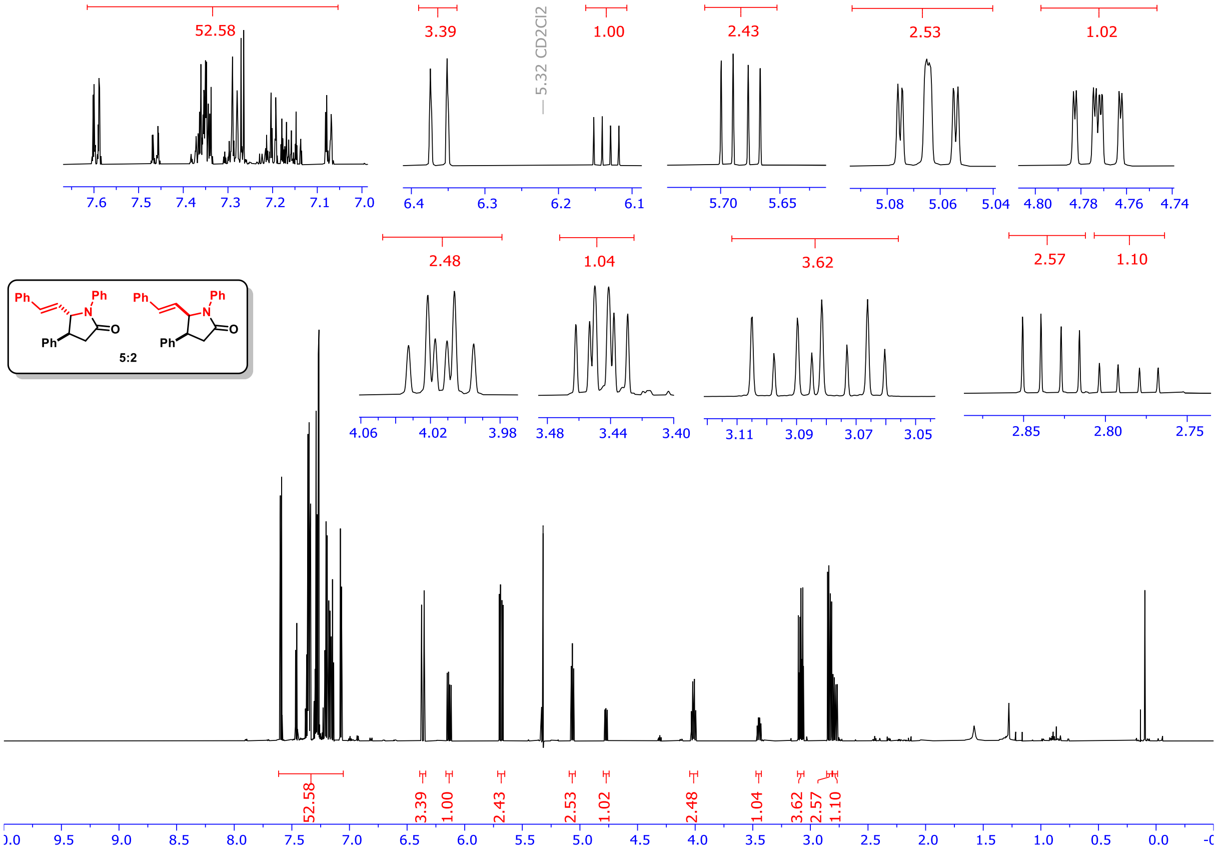

^{1}H-NMR spectrum (700 MHz, CD_2Cl_2) of **3b**. Signals of both diastereomers are integrated separately.

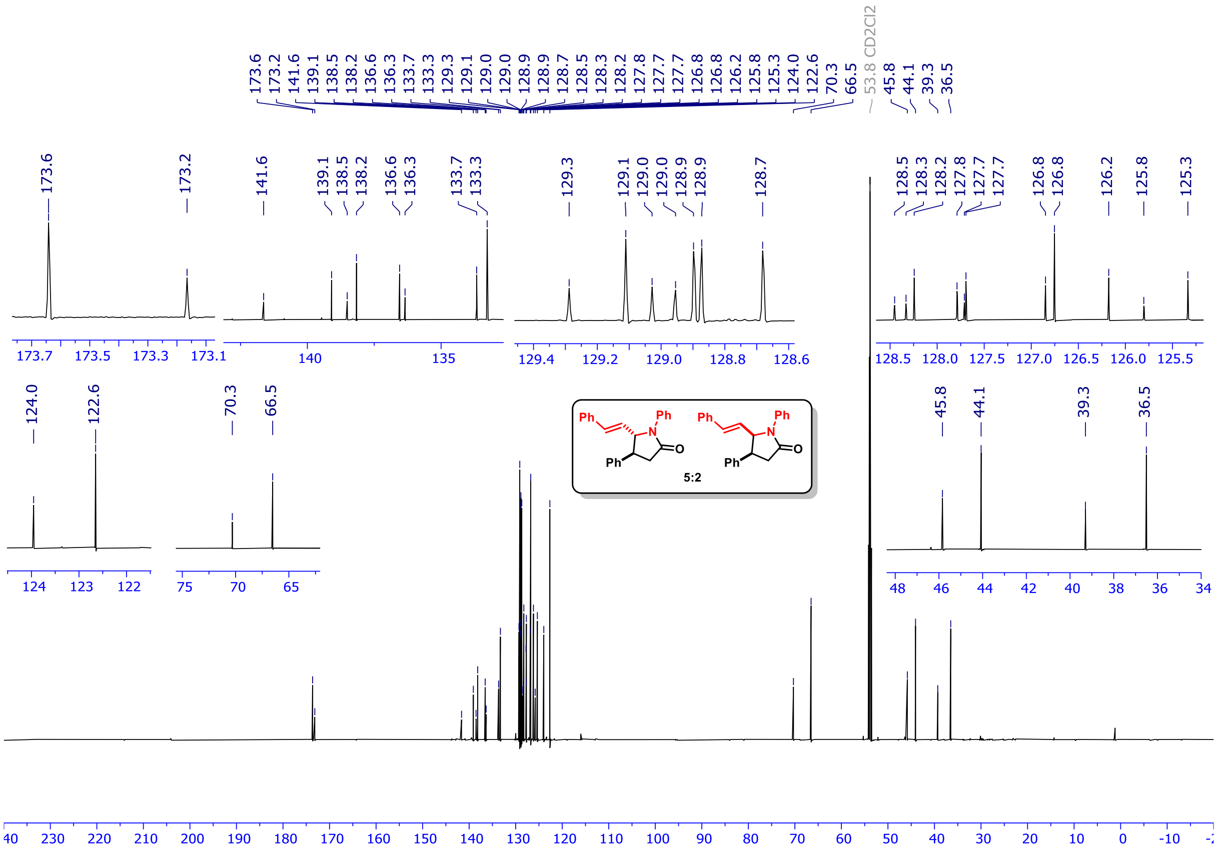

^{13}C-NMR spectrum (176 MHz, CD_2Cl_2) of **3b**.

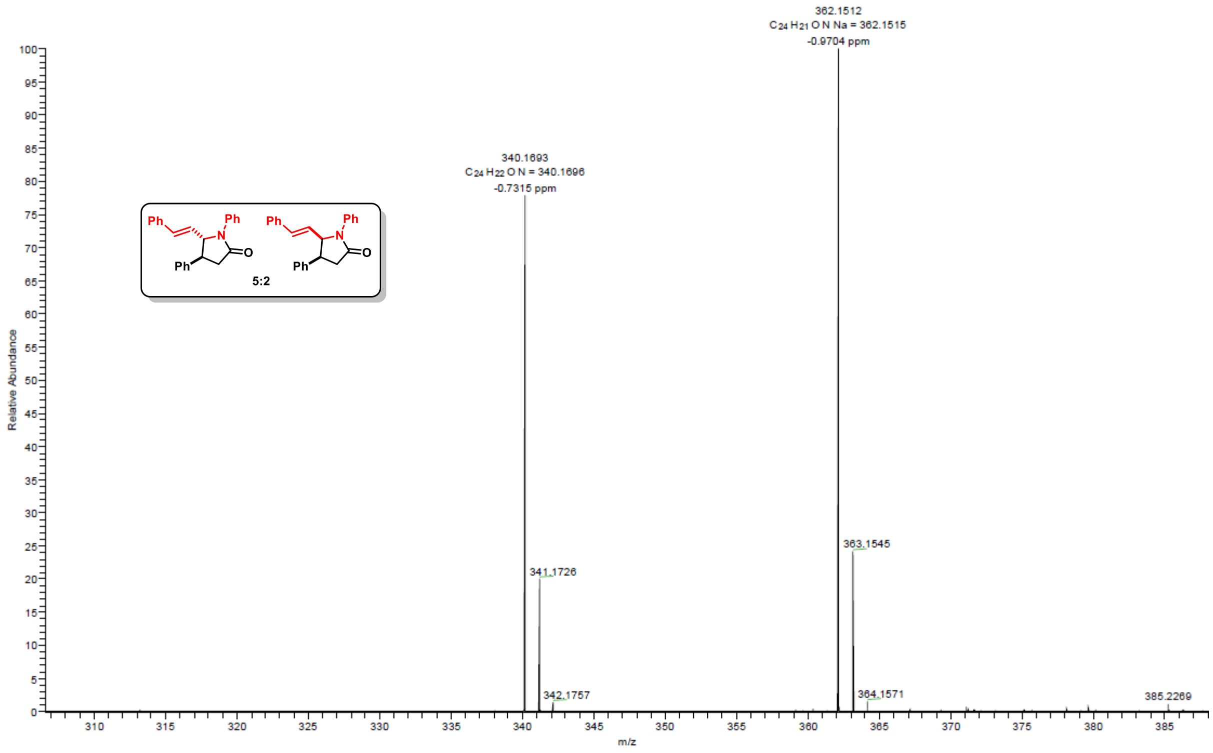

HR-MS Spectrum (ESI+) of **3b** (mixture of *anti*- and *syn*-isomer).

Lactam 3c

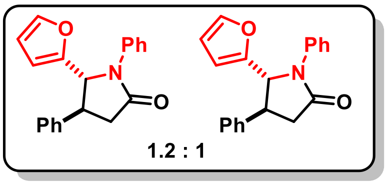

Prepared according to the **General Procedure** from SCP **1a** (54.9 mg, 0.20 mmol, 1.00 eq.) and (furan-2-yl)-*N*-phenylmethanimine (274 mg, 1.60 mmol, 8.00 eq.). Purification by flash column chromatography (SiO_2, *n*-pentane/acetone 10:1) afforded lactam **3c** (24.8 mg, 0.08 mmol, 41%, *anti*/*syn* 1.2:1) as a mixture of two inseparable diastereomers as a yellow oil.

FTIR (ATR): $\tilde{\nu}$ [cm^{-1}] = 1697, 1598, 1497, 1369, 1334, 1207, 1150, 1012, 747, 694.

^{1}H-NMR (500 MHz, CD_2Cl_2): δ_H (ppm) = 7.51–7.02 (m, 10H), 6.27 (dd, *J* = 3.3, 1.8 Hz, 1H), 6.18 (dd, *J* = 3.3, 0.9 Hz, 1H), 6.14 (dd, *J* = 3.3, 1.8 Hz, 1H), 5.98 (dd, *J* = 3.3, 0.8 Hz, 1H), 5.39 (d, *J* = 7.8 Hz, 1H), 5.16 (d, *J* = 5.5 Hz, 1H), 4.10 (dt, *J* = 12.7, 7.9 Hz, 1H), 3.78 (ddd, *J* = 8.9, 7.0, 5.5 Hz, 1H), 3.32 (dd, *J* = 16.4, 12.7 Hz, 1H), 3.19 (dd, *J* = 17.1, 8.9 Hz, 1H), 2.81 (dd, *J* = 7.5, 4.0 Hz, 1H), 2.77 (dd, *J* = 7.5, 4.7 Hz, 1H). *Signals of the anti-isomer are underlined.*

^{13}C-NMR (126 MHz, CD_2Cl_2): δ_C (ppm) = 173.7, 173.2, 152.2, 151.0, 143.2, 142.8, 142.0, 139.0, 138.3, 137.4, 129.4, 129.2, 129.1, 128.6, 128.2, 127.8, 127.6, 127.3, 126.2, 125.7, 124.1, 122.7, 110.7, 110.6, 109.7, 109.4, 65.6, 63.6, 44.5, 44.3, 39.0, 36.3. *Due to overlap, not all aromatic signals are resolved.*

HR-MS (ESI+, Orbitrap): calc. for $C_{20}H_{17}O_2NNa$ $[M+Na]^+$: 326.1152, found: 326.1145.

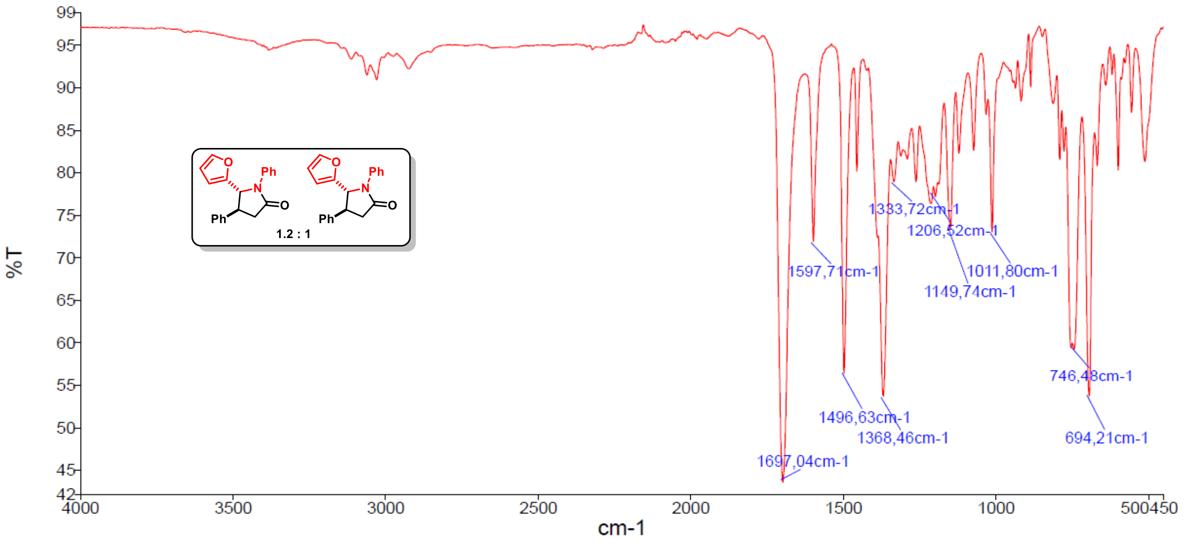

FT-IR Spectrum (ATR, thin film) of **3c** (mixture of *anti*- and *syn*-isomer).

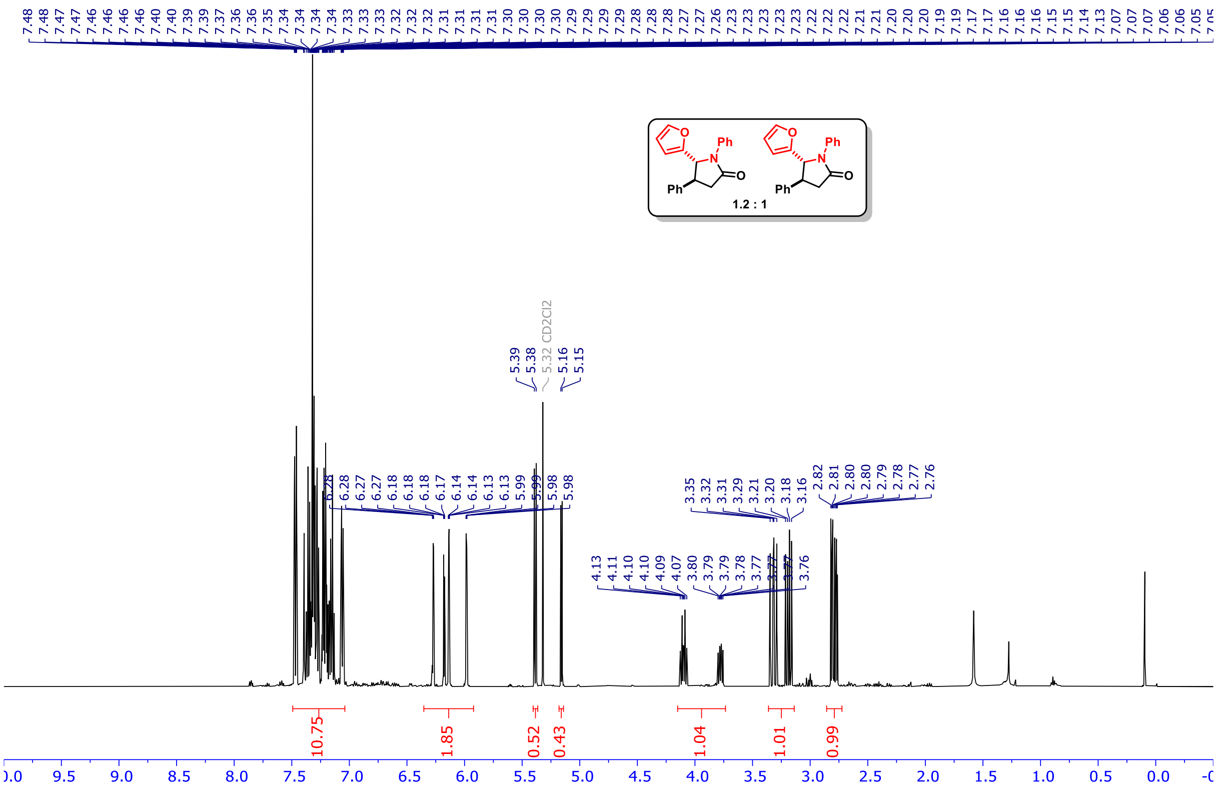

^{1}H-NMR spectrum (500 MHz, CD_2Cl_2) of **3c** (mixture of *anti*- and *syn*-isomer). Signals of identical protons of both diastereomers are integrated together.

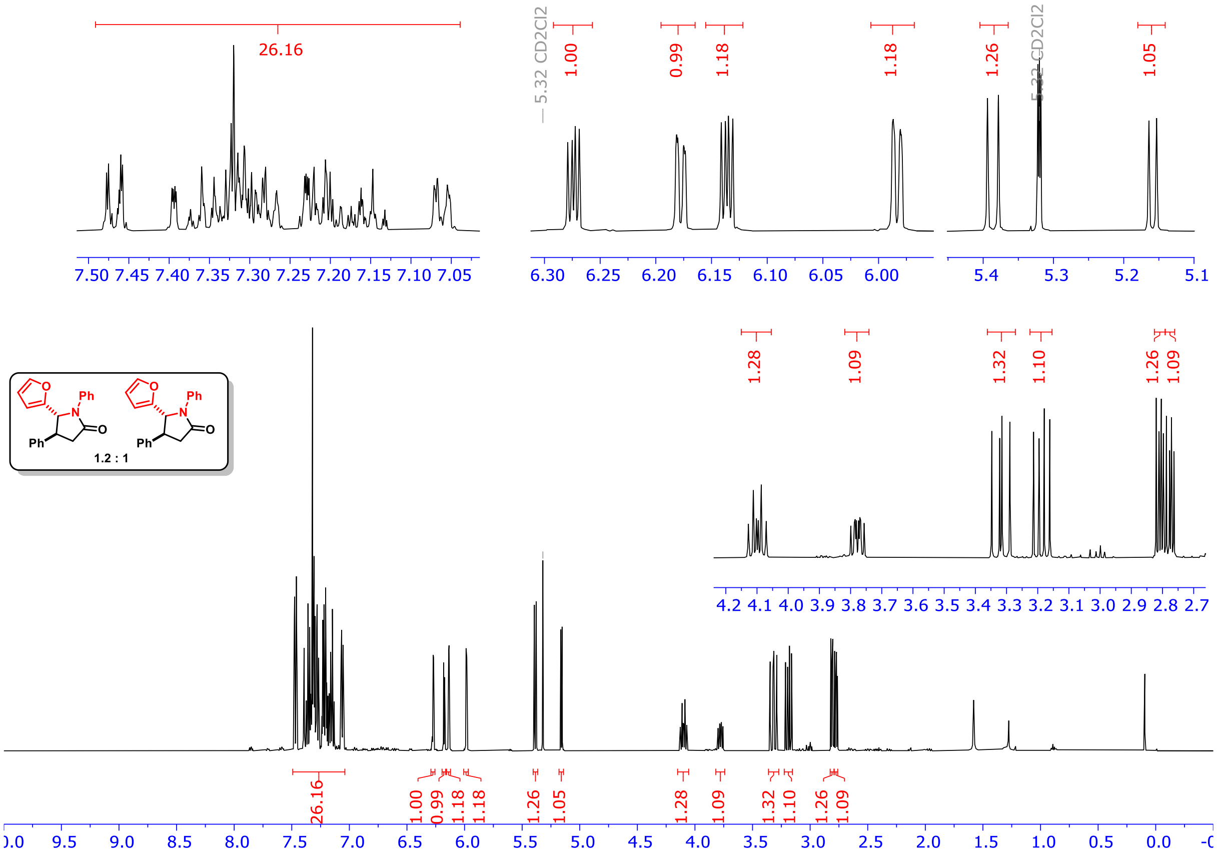

^{1}H-NMR spectrum (500 MHz, CD_2Cl_2) of **3c**. Signals of both diastereomers are integrated separately.

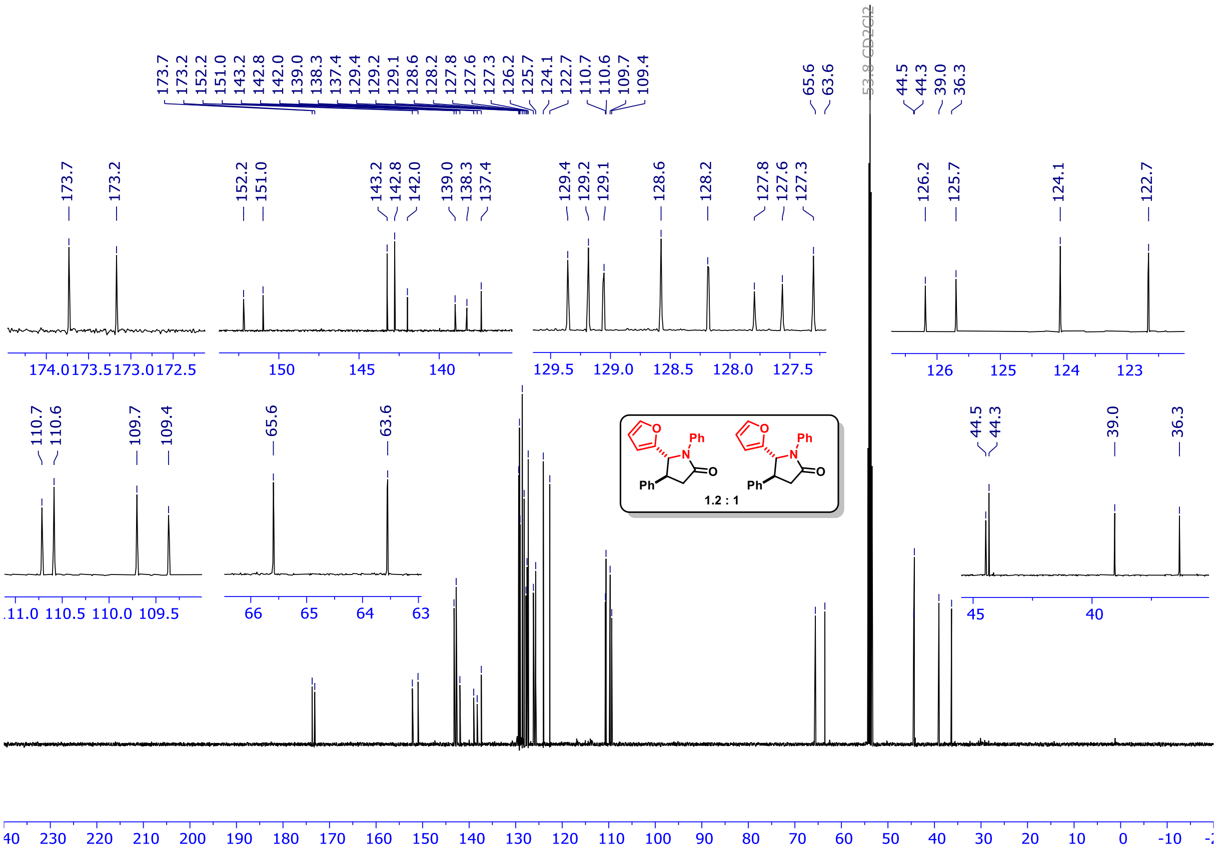

^{13}C-NMR spectrum (126 MHz, CD_2Cl_2) of **3c**.

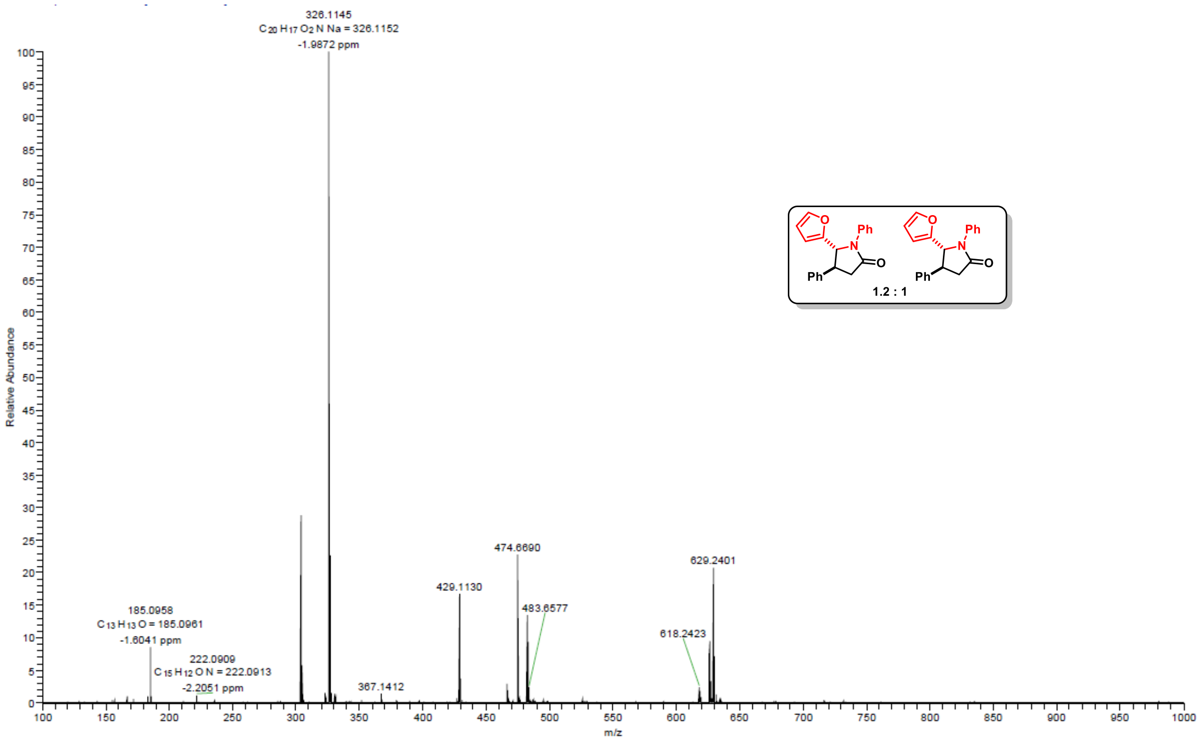

HR-MS Spectrum (ESI+) of **3c** (mixture of *anti*- and *syn*-isomer).

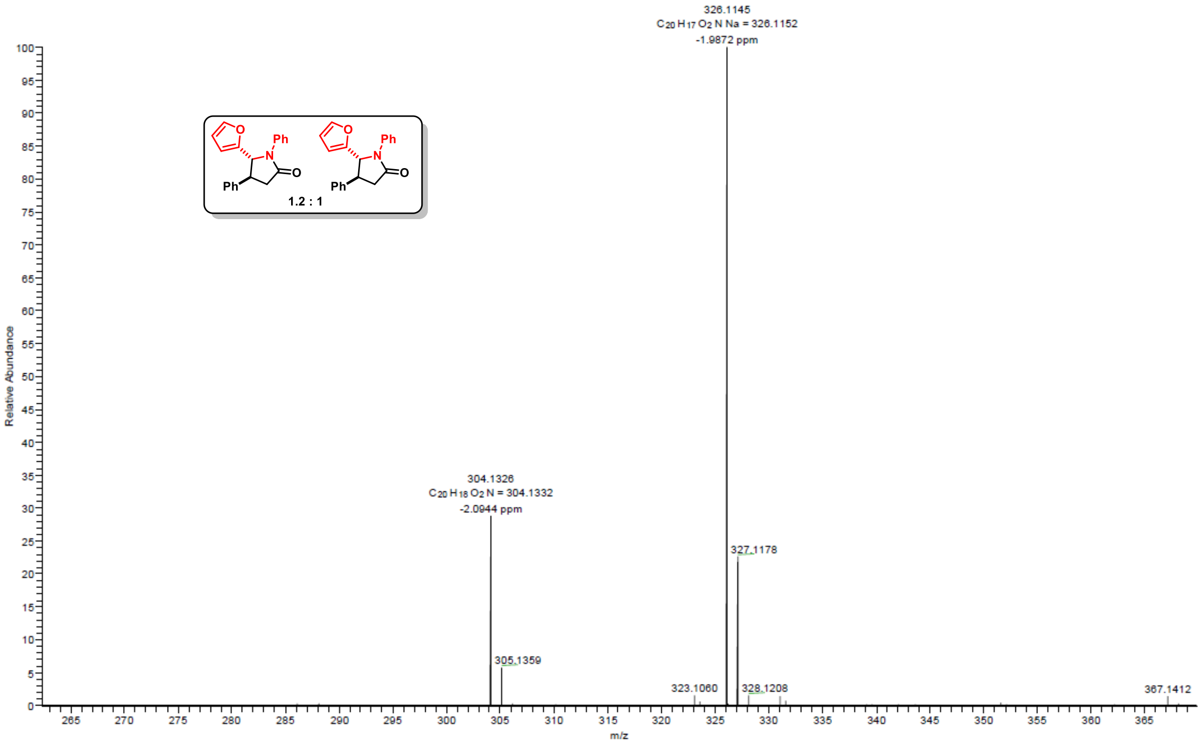

Excerpt of the HR-MS Spectrum (ESI+) of **3c** (mixture of *anti*- and *syn*-isomer).

Ketone Substrate Scope

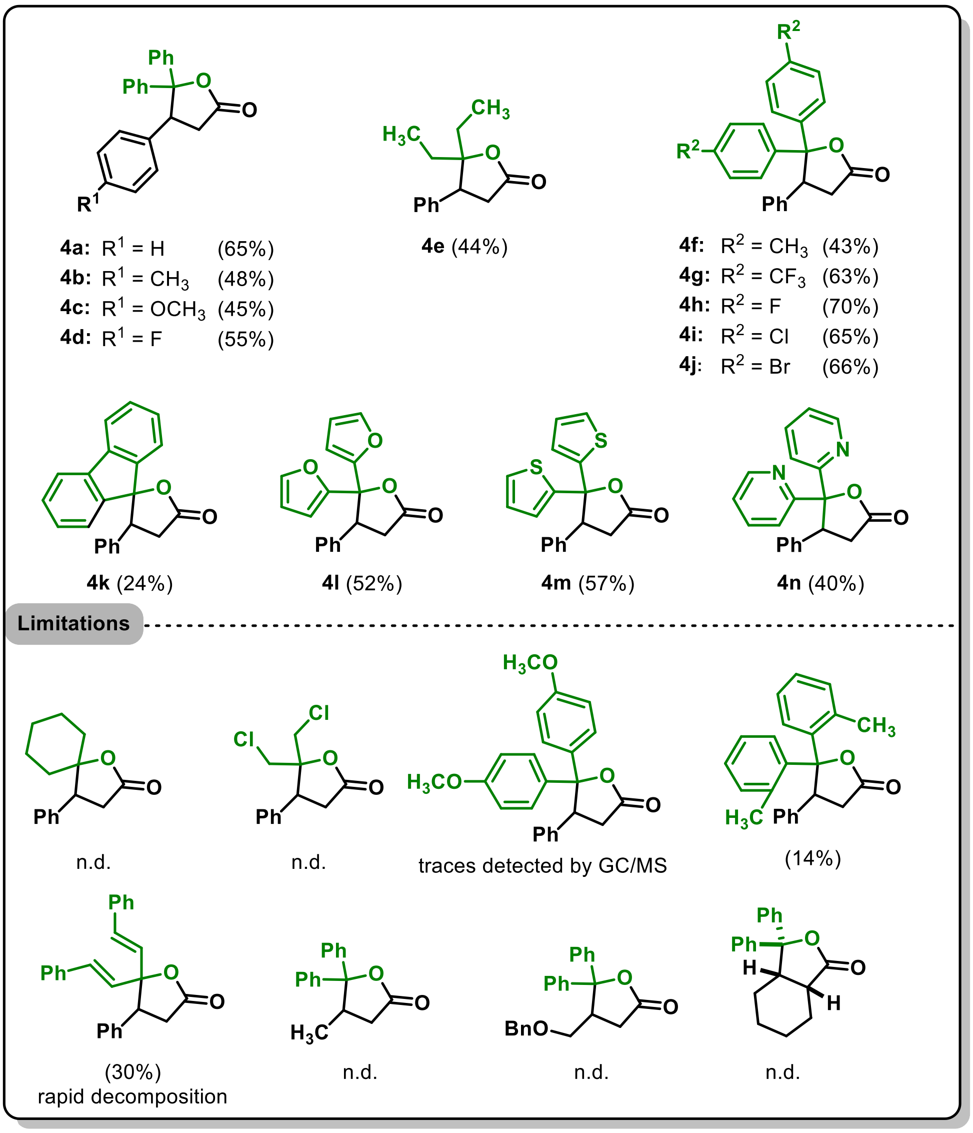

Lactone 4a

Prepared according to the **General Procedure** from SCP **1a** (54.9 mg, 0.20 mmol, 1.00 eq.) and benzophenone (292 mg, 1.60 mmol, 8.00 eq.). Purification by flash column chromatography (SiO_2, *n*-pentane/EtOAc 20:1) afforded lactone **4a** (41.1 mg, 0.13 mmol, 65%) as a colorless solid.

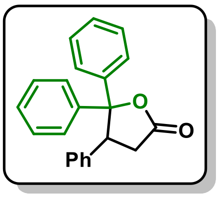

FTIR (ATR): $\tilde{\nu}$ [cm^{-1}] = 1769, 1202, 1136, 978, 965, 890, 746, 719, 704, 691.

^{1}H-NMR (500 MHz, CD_2Cl_2): δ_H (ppm) = 7.70–7.62 (m, 2H), 7.46–7.39 (m, 2H), 7.37–7.31 (m, 1H), 7.17–7.04 (m, 8H), 7.02–6.95 (m, 2H), 4.53 (dd, J = 8.1, 4.2 Hz, 1H), 2.99 (dd, J = 17.5, 8.1 Hz, 1H), 2.76 (dd, J = 17.5, 4.2 Hz, 1H).

^{13}C-NMR (126 MHz, CD_2Cl_2): δ_C (ppm) = 175.8 (qC), 143.8 (qC), 140.8 (qC), 139.4 (qC), 129.1 (CH), 129.0 (CH), 128.7 (CH), 128.5 (CH), 128.1 (CH), 127.6 (CH), 127.5 (CH), 126.6 (CH), 126.4 (CH), 93.1 (qC), 51.1 (CH), 37.9 (CH_2).

HR-MS (ESI+, Orbitrap): calc. for $C_{22}H_{19}O_2$ $[M+H]^+$: 315.1380, found: 315.1380.

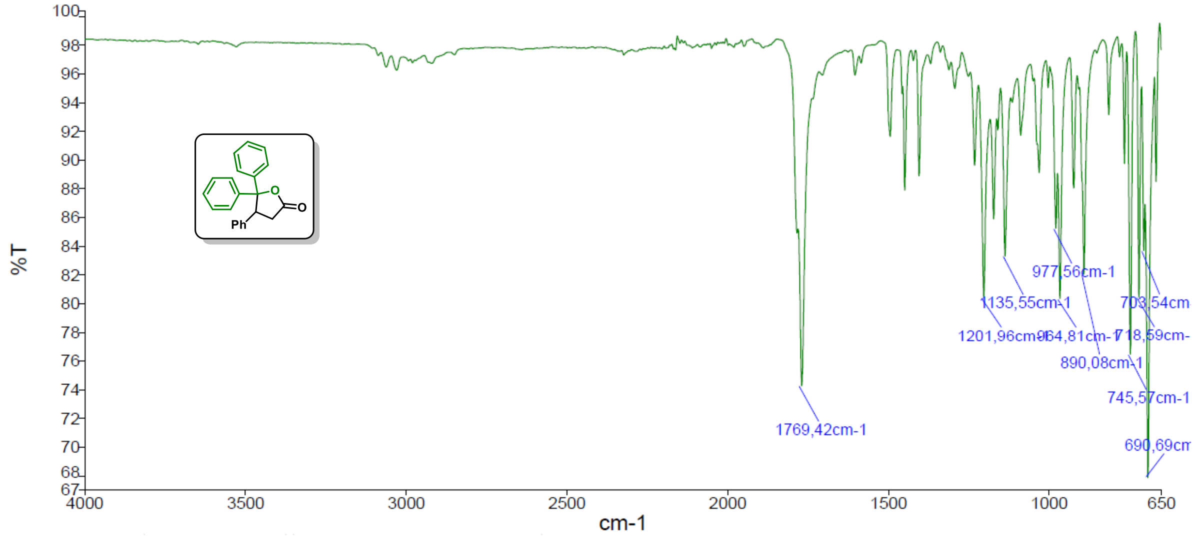

FT-IR Spectrum (ATR, thin film) of **4a**.

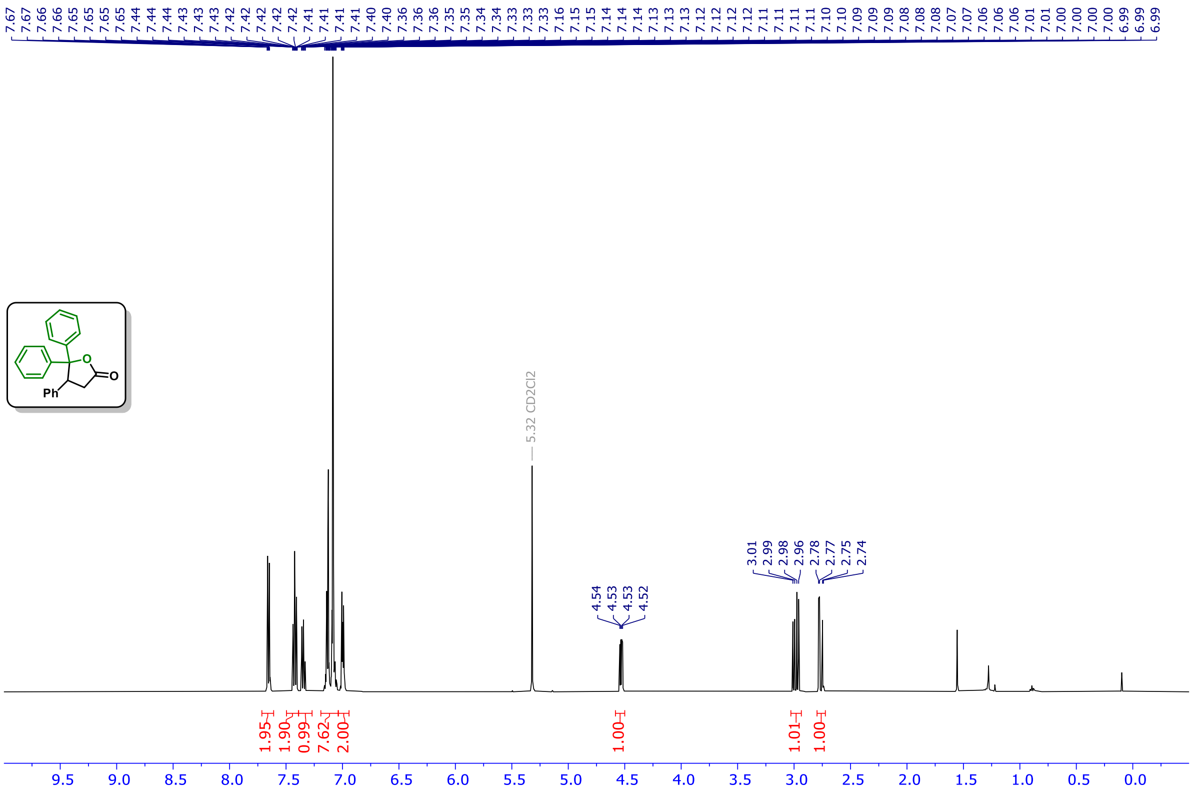

^{1}H-NMR spectrum (500 MHz, CD_2Cl_2) of **4a**.

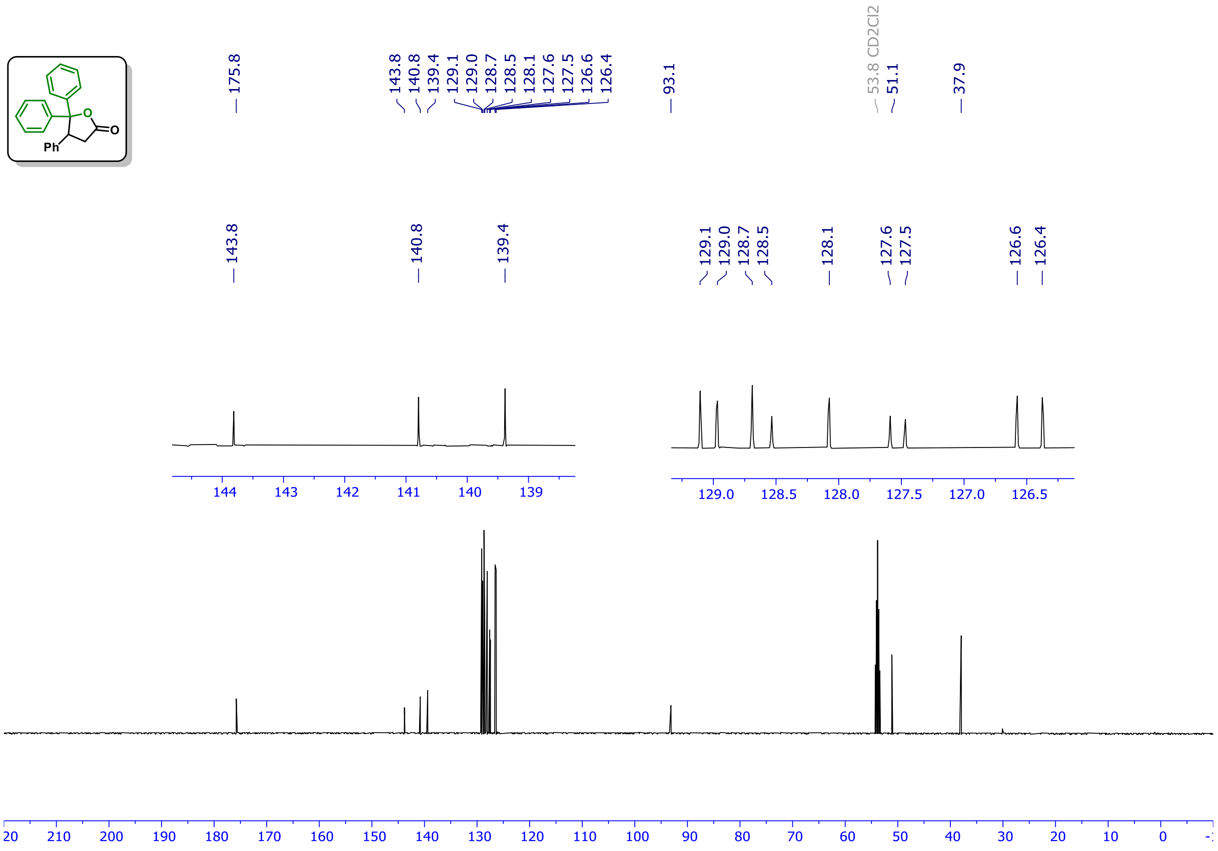

^{13}C-NMR spectrum (126 MHz, CD_2Cl_2) of **4a**.

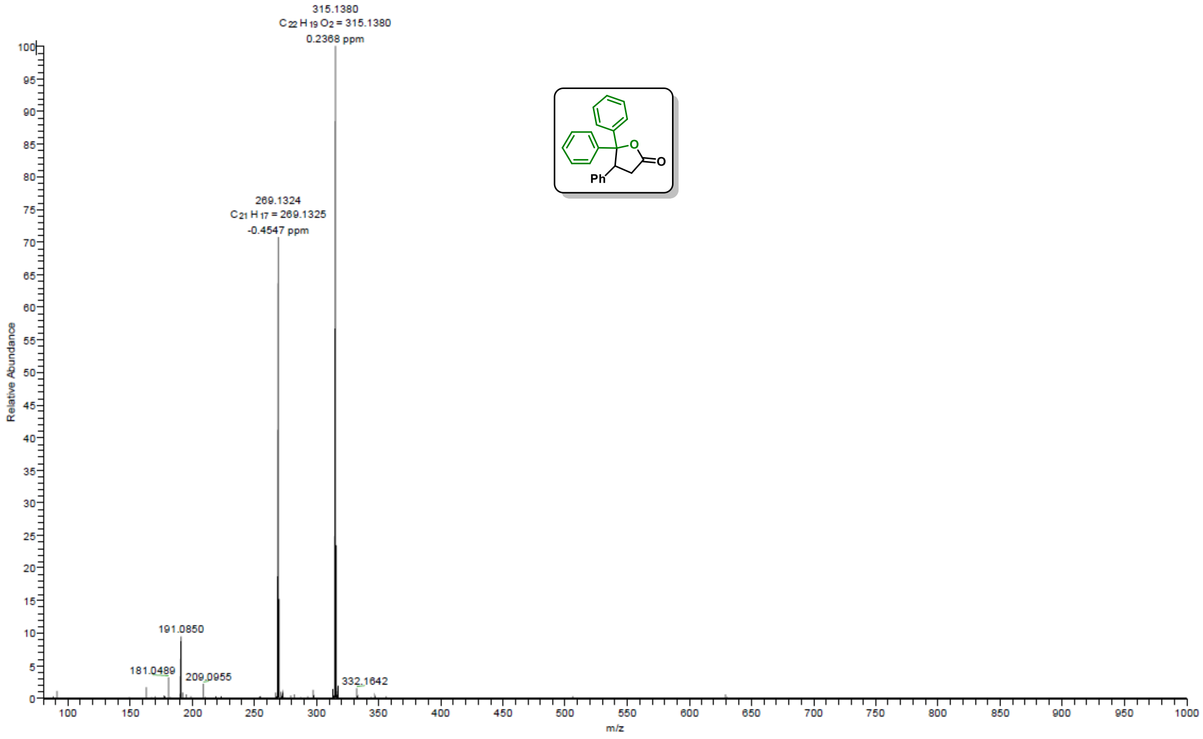

HR-MS Spectrum (ESI+) of **4a**.

Lactone 4b

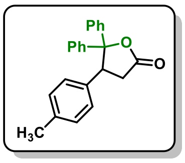

Prepared according to the **General Procedure** from SCP **1b** (57.7 mg, 0.20 mmol, 1.00 eq.) and benzophenone (292 mg, 1.60 mmol, 8.00 eq.). Purification by flash column chromatography (SiO_2, *n*-pentane/EtOAc 20:1) afforded lactone **4b** (31.2 mg, 0.10 mmol, 48%) as a colorless solid.

FTIR (ATR): $\tilde{\nu}$ [cm^{-1}] = 1775, 1448, 1230, 1205, 1139, 981, 969, 759, 727, 699.

^{1}H-NMR (700 MHz, CD_2Cl_2): δ_H (ppm) = 7.67–7.62 (m, 2H), 7.42–7.36 (m, 2H), 7.35–7.28 (m, 1H), 7.12–7.04 (m, 5H), 6.95–6.92 (m, 2H), 6.88–6.73 (m, 2H), 4.46 (dd, *J* = 8.0, 5.2 Hz, 1H), 2.96 (dd, *J* = 17.4, 8.0 Hz, 1H), 2.77 (dd, *J* = 17.4, 5.2 Hz, 1H), 2.24 (s, 3H).

^{13}C-NMR (176 MHz, CD_2Cl_2): δ_C (ppm) = 175.9, 143.4, 140.1, 137.1, 135.4, 129.1, 128.7, 128.6, 128.2, 127.8, 127.3, 126.5, 126.2, 93.0, 50.9, 37.7, 21.1.

HR-MS (ESI+, Orbitrap): calc. for $C_{23}H_{20}O_2Na$ $[M+Na]^+$: 351.1356, found: 351.1352.

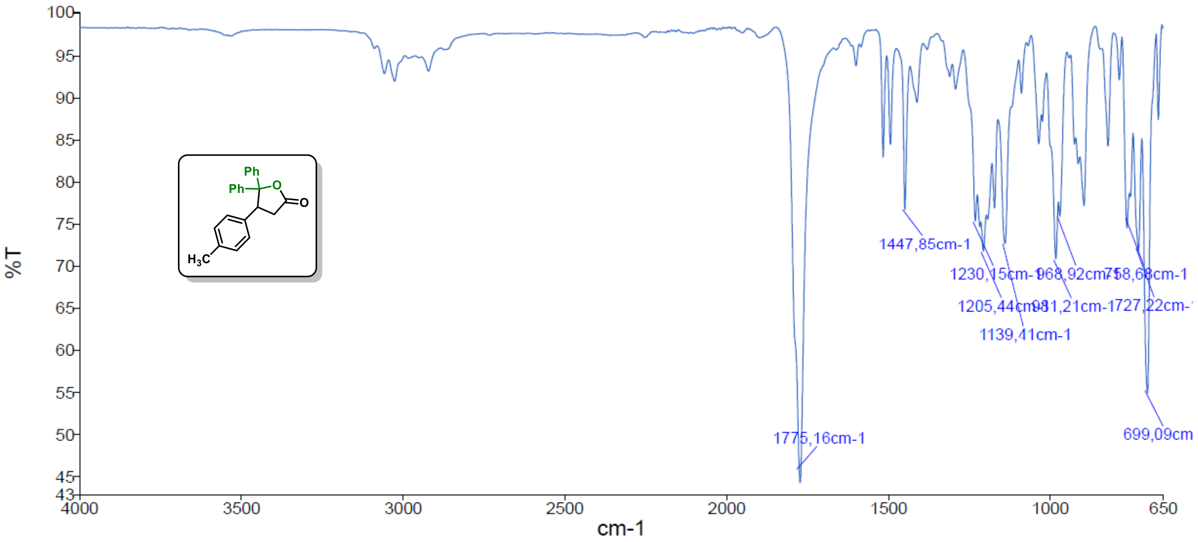

FT-IR Spectrum (ATR, thin film) of **4b**.

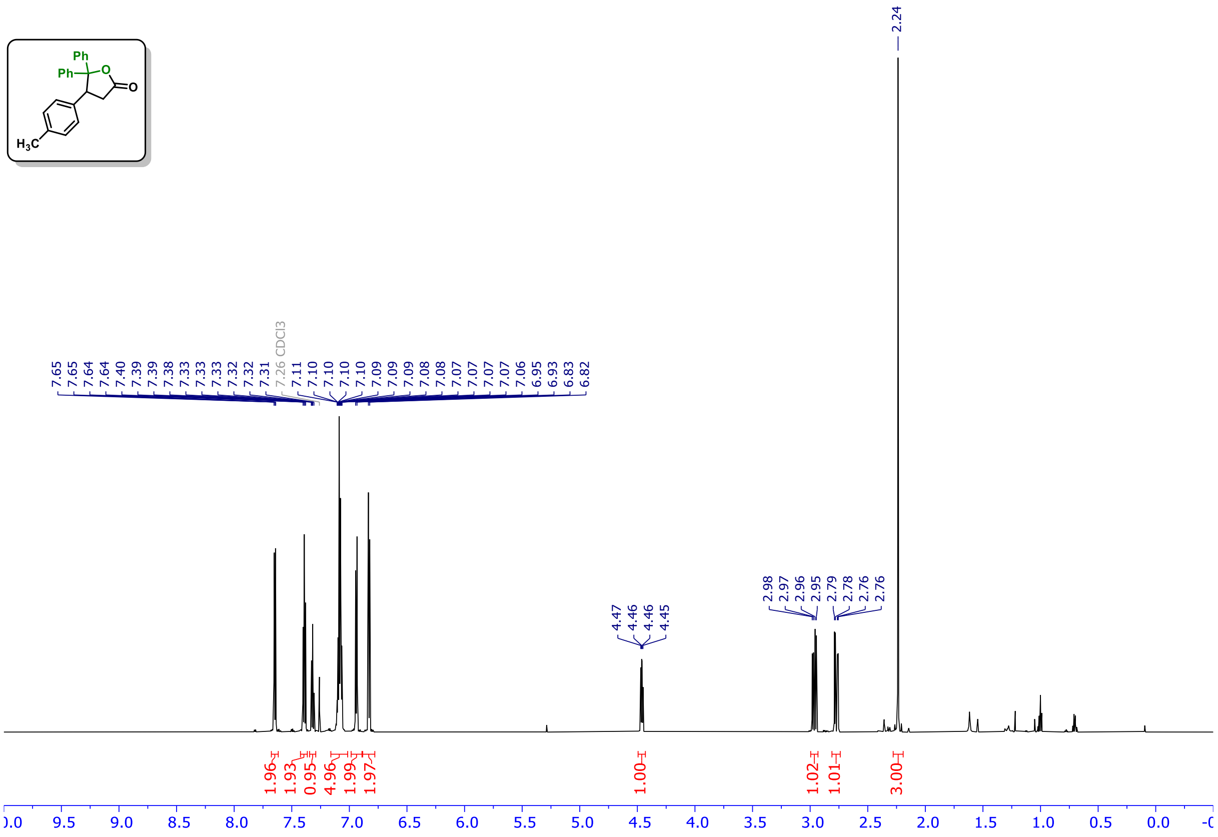

^{1}H-NMR spectrum (700 MHz, $CDCl_3$) of **4b**.

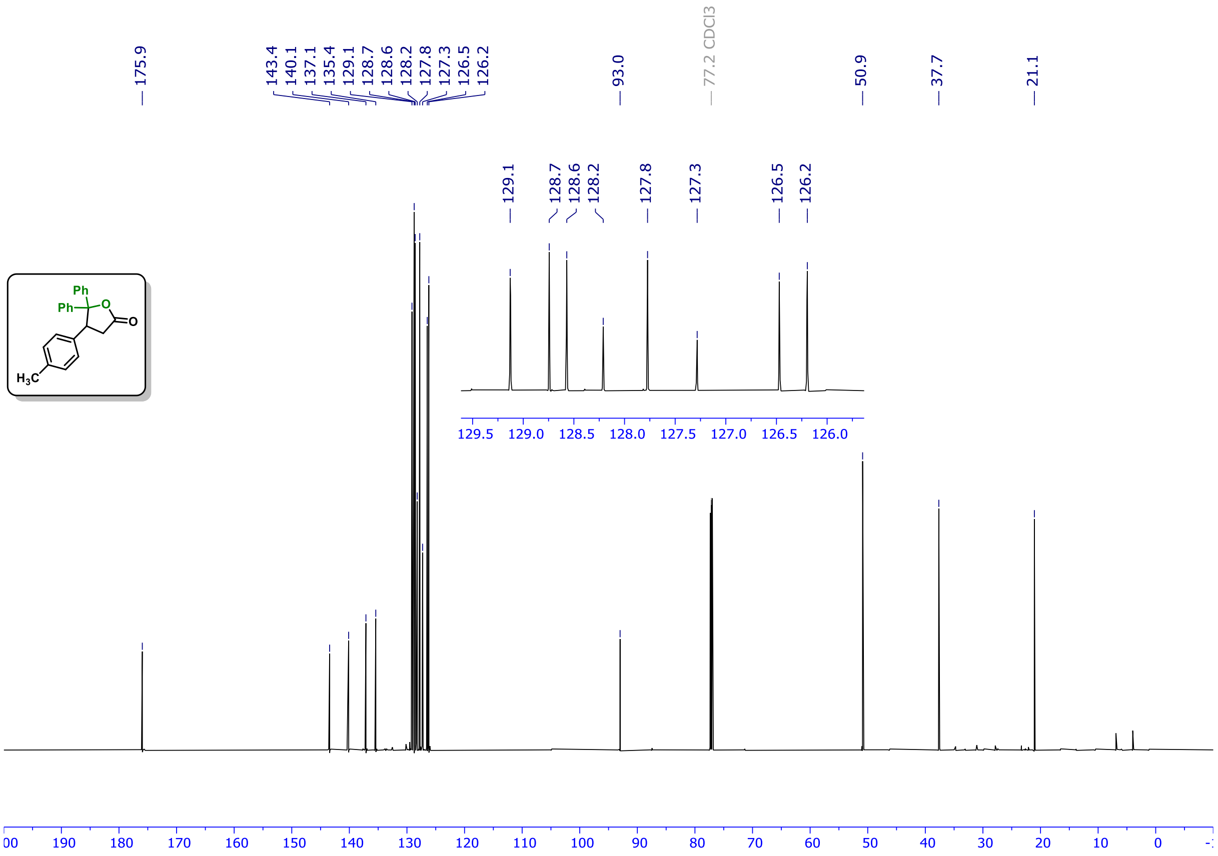

^{13}C-NMR spectrum (176 MHz, $CDCl_3$) of **4b**.

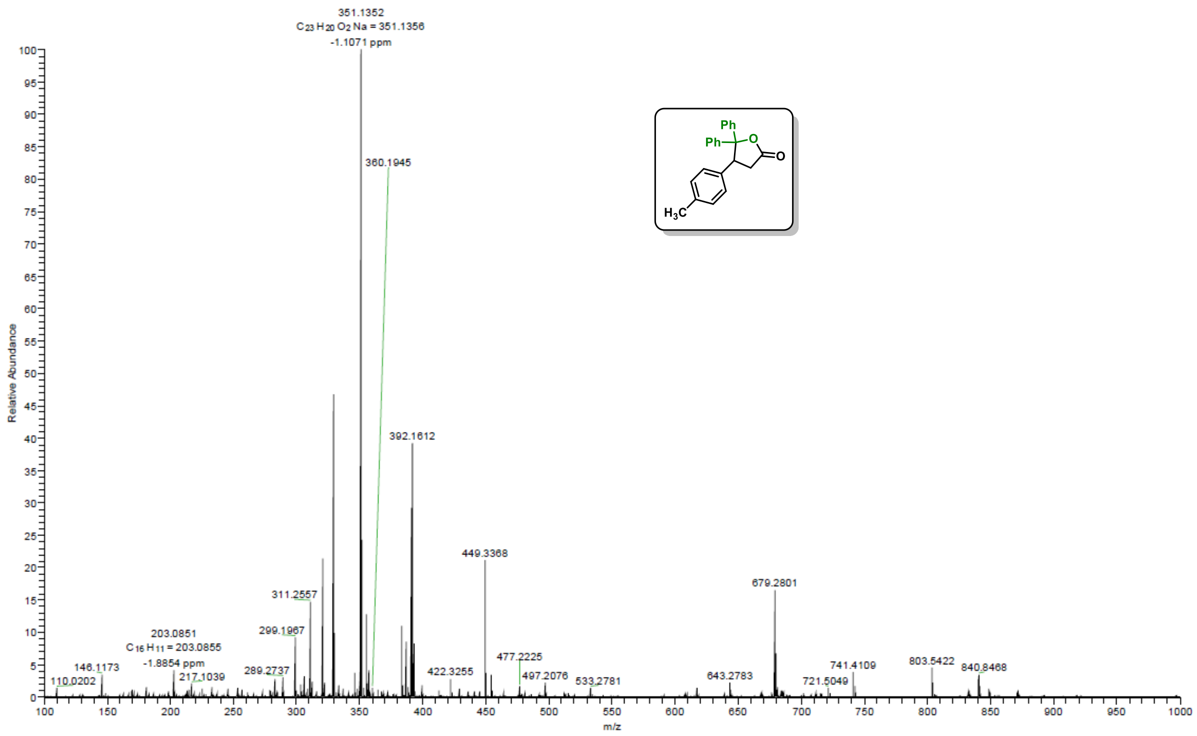

HR-MS Spectrum (ESI+) of **4b**.

Lactone 4c

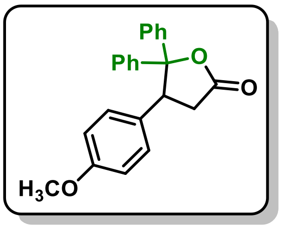

Prepared according to the **General Procedure** from SCP **1c** (60.9 mg, 0.20 mmol, 1.00 eq.) and benzophenone (292 mg, 1.60 mmol, 8.00 eq.). Purification by flash column chromatography (SiO_2, *n*-pentane/EtOAc 10:1 to 2:1) afforded lactone **4c** (31.2 mg, 0.09 mmol, 45%) as a colorless solid.

FTIR (ATR): $\tilde{v}$ [cm^{-1}] = 1775, 1513, 1250, 1230, 1212, 1182, 1140, 1032, 982, 700.

^{1}H-NMR (700 MHz, CD_2Cl_2): δ_H (ppm) = 7.68–7.62 (m, 2H), 7.44–7.39 (m, 2H), 7.36–7.29 (m, 1H), 7.16–7.05 (m, 5H), 6.93–6.85 (m, 2H), 6.71–6.63 (m, 2H), 4.49 (dd, *J* = 8.0, 4.9 Hz, 1H), 3.70 (s, 3H), 2.95 (dd, *J* = 17.5, 8.0 Hz, 1H), 2.72 (dd, *J* = 17.5, 4.9 Hz, 1H).

^{13}C-NMR (176 MHz, CD_2Cl_2): δ_C (ppm) = 175.9, 159.2, 143.9, 140.8, 131.1, 130.0, 129.1, 128.5, 128.1, 127.5, 126.7, 126.4, 114.0, 93.1, 55.5, 50.5, 38.0.

HR-MS (GC-APCI, +, Q-TOF): calc. for $C_{23}H_{21}O_3$ $[M+H]^+$: 345.1485, found: 345.1486.

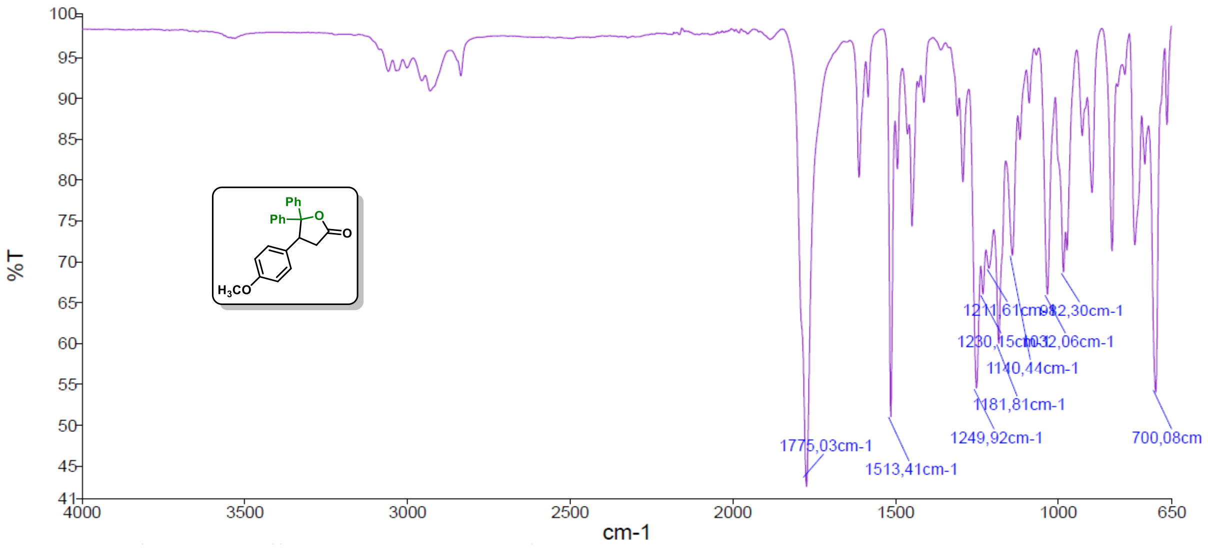

FT-IR Spectrum (ATR, thin film) of **4c**.

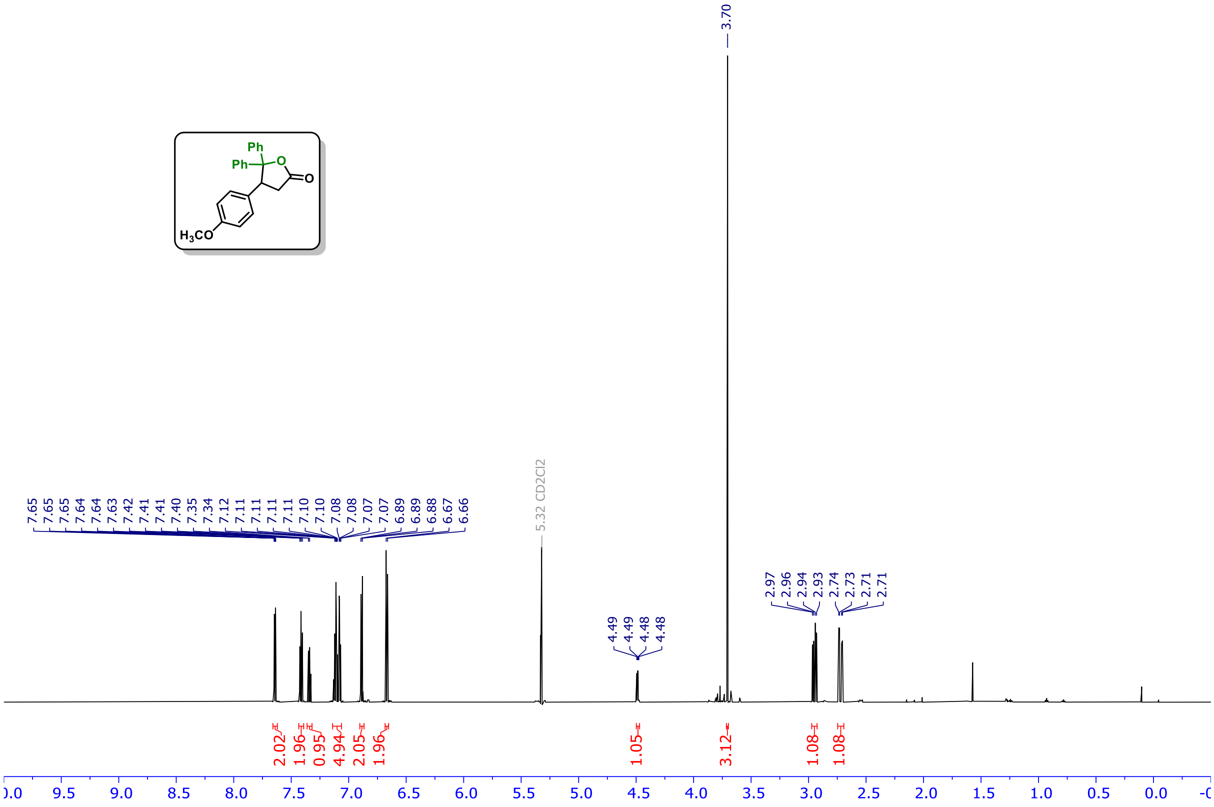

^{1}H-NMR spectrum (700 MHz, CD_2Cl_2) of **4c**.

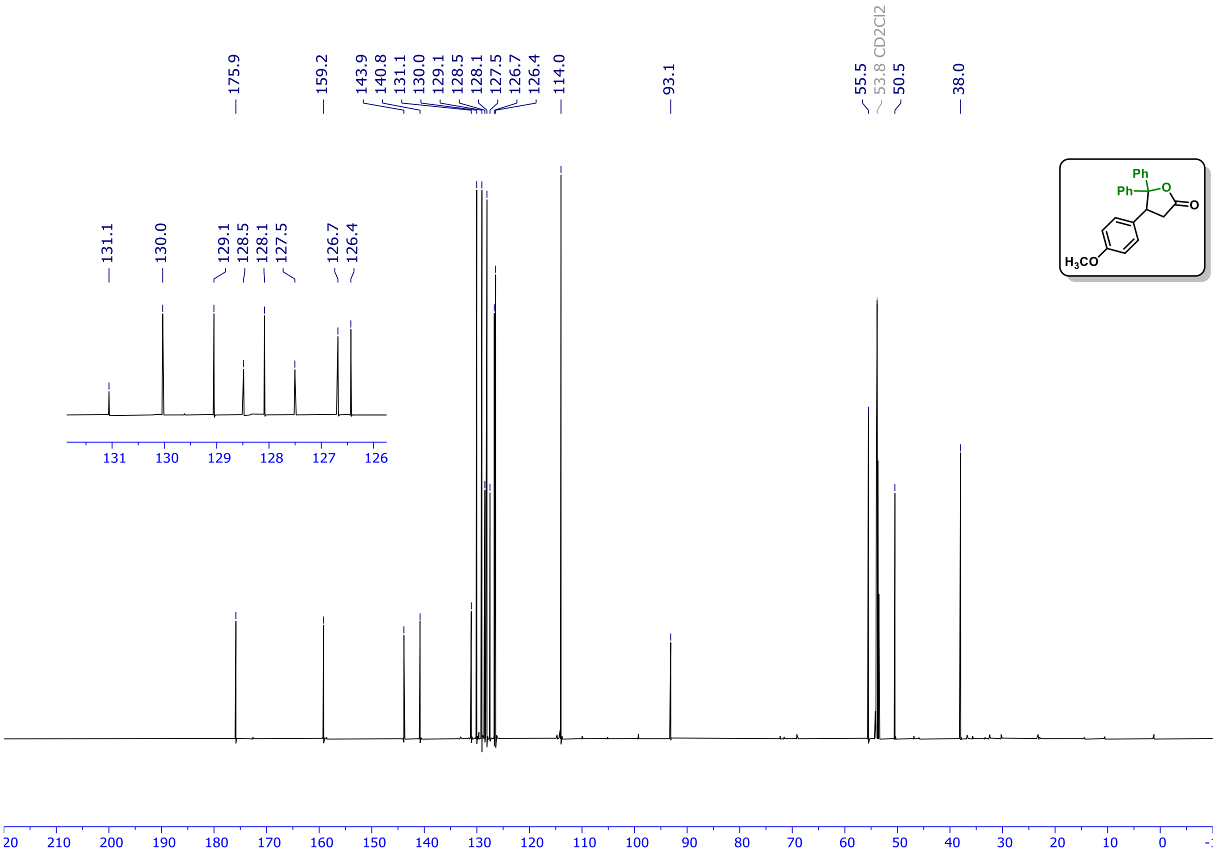

^{13}C-NMR spectrum (176 MHz, CD_2Cl_2) of **4c**.

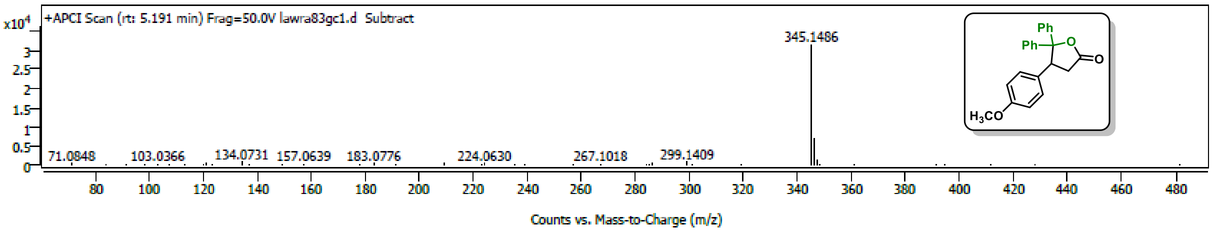

HR-MS Spectrum (APCI,+) of **4c**.

Lactone 4d

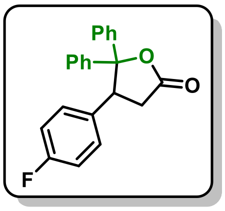

Prepared according to the **General Procedure** from SCP **1d** (58.5 mg, 0.20 mmol, 1.00 eq.) and benzophenone (292 mg, 1.60 mmol, 8.00 eq.). Purification by flash column chromatography (SiO_2, *n*-pentane/acetone 8:1) afforded lactone **4d** (36.5 mg, 0.11 mmol, 55%) as a pale-yellow solid.

FTIR (ATR): $\tilde{\nu}$ [cm^{-1}] = 1779, 1511, 1449, 1228, 1166, 1140, 981, 839, 762, 701.

^{1}**H-NMR** (700 MHz, $CDCl_3$): δ_H (ppm) = 7.65–7.61 (m, 2H), 7.43–7.38 (m, 2H), 7.35–7.31 (m, 1H), 7.11–7.06 (m, 3H), 7.06–7.01 (m, 2H), 6.94–6.88 (m, 2H), 6.85–6.79 (m, 2H), 4.48 (dd, *J* = 8.1, 4.5 Hz, 1H), 2.99 (dd, *J* = 17.5, 8.1 Hz, 1H), 2.75 (dd, *J* = 17.5, 4.5 Hz, 1H).

^{13}C-NMR (176 MHz, CDCl$_3$): δ_C (ppm) = 175.6, 162.0 (d, J = 246 Hz), 143.0, 140.0, 134.5 (d, J = 3.1 Hz), 130.2 (d, J = 8.4 Hz), 128.9, 128.4, 128.0, 127.5, 126.3, 126.2, 115.4 (d, J = 21.4 Hz), 93.0, 50.4, 37.7.

^{19}F-NMR (659 MHz, CDCl$_3$): δ_F (ppm) = −114.7 (m).

HR-MS (GC-APCI, +, Q-TOF): calc. for $C_{22}H_{18}FO_2$ [M+H]$^+$: 333.1285, found: 333.1285.

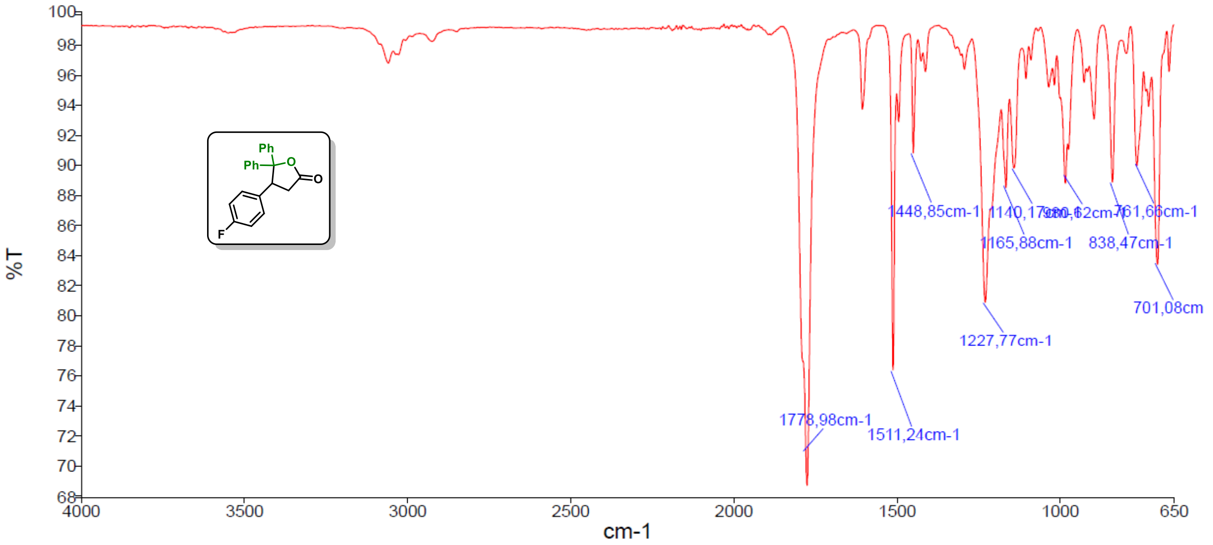

FT-IR Spectrum (ATR, thin film) of **4d**.

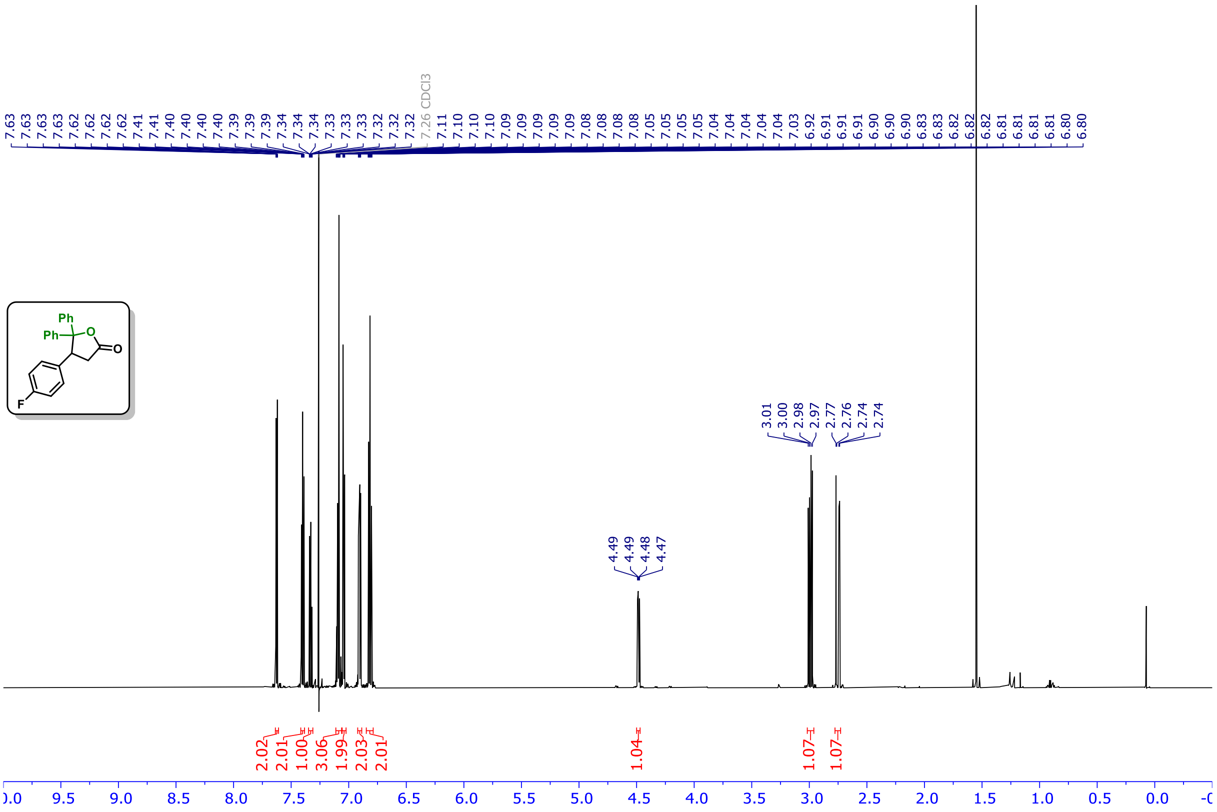

^{1}H-NMR spectrum (700 MHz, CDCl$_3$) of **4d**.

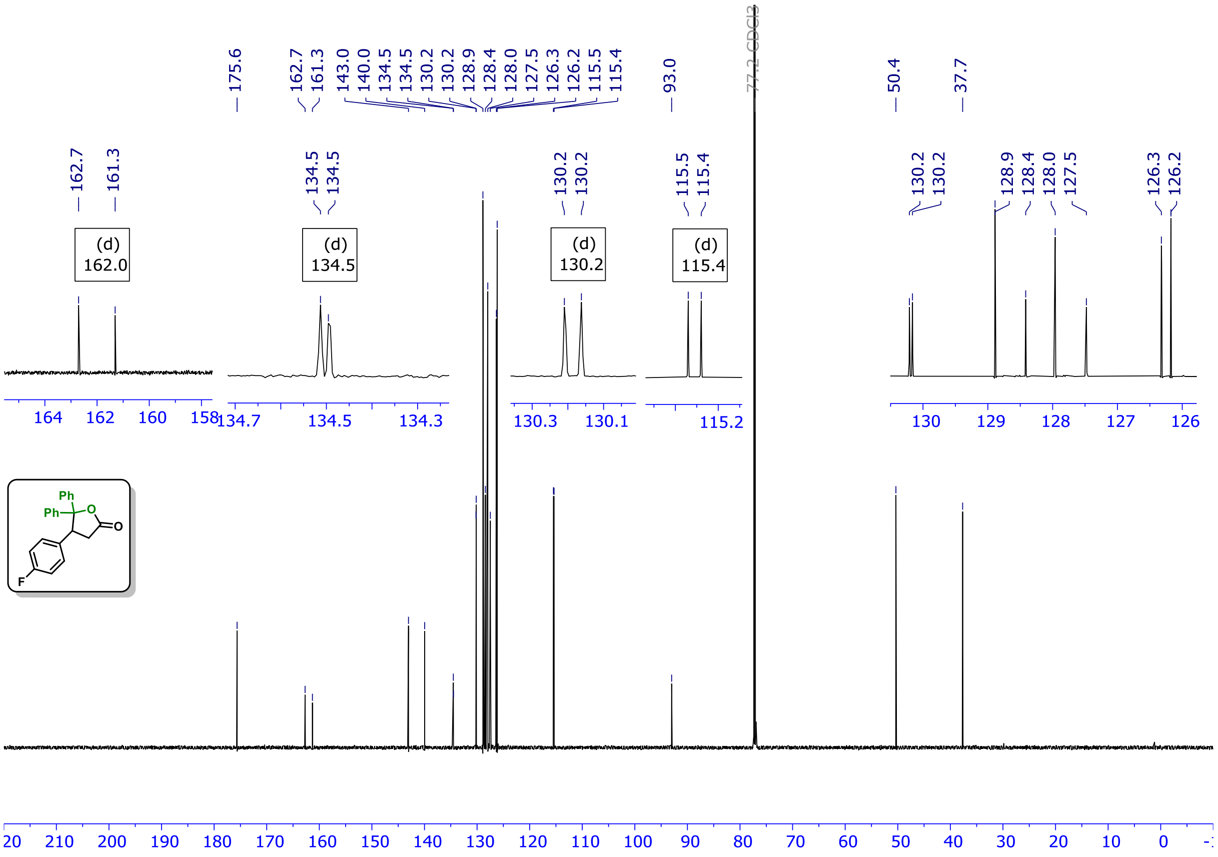

^{13}C-NMR spectrum (176 MHz, CDCl$_3$) of **4d**.

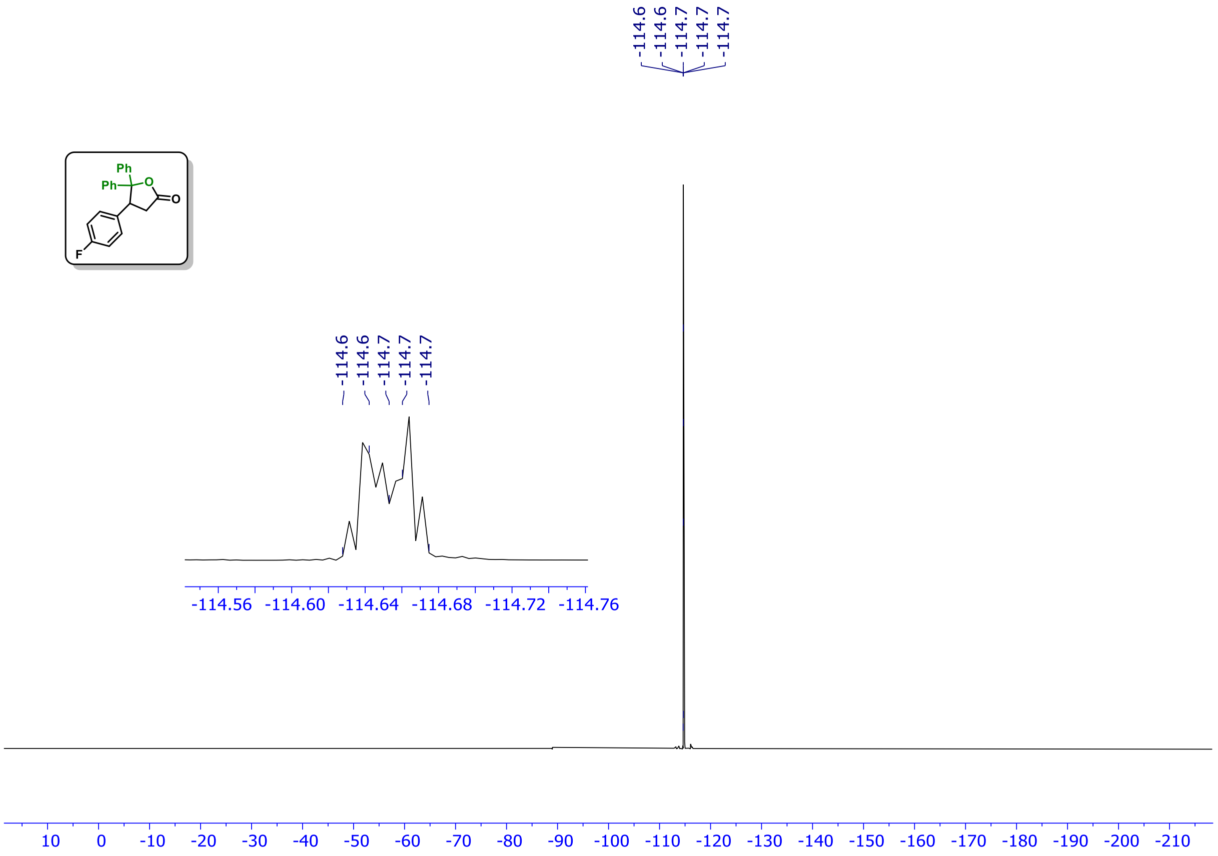

^{19}F-NMR spectrum (659 MHz, CDCl$_3$) of **4d**.

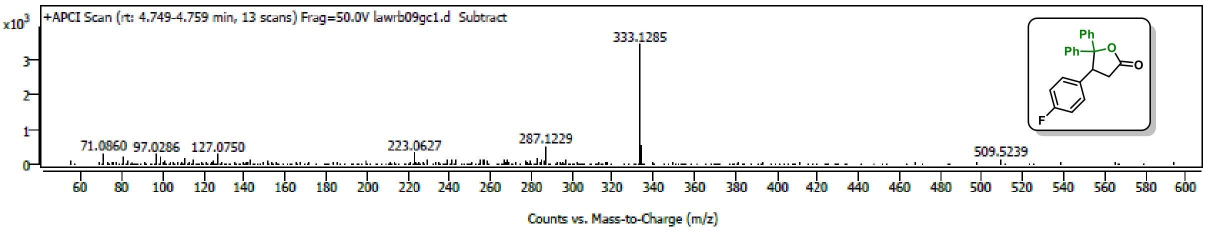

HR-MS Spectrum (APCI,+) of **4d**.

Lactone 4e

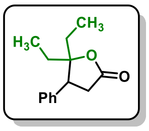

Prepared according to the **General Procedure** from SCP **1a** (54.9 mg, 0.20 mmol, 1.00 eq.) and 3-pentanone (138 mg, 1.60 mmol, 170 µL, 8.00 eq.). Purification by flash column chromatography (SiO_2, *n*-pentane/EtOAc 15:1) afforded lactone **4e** (19.0 mg, 0.09 mmol, 44%) as a colorless oil.

FTIR (ATR): $\tilde{\nu}$ [cm^{-1}] = 2972, 1764, 1456, 1222, 1190, 1109, 935, 922, 766, 702.

^{1}H-NMR (500 MHz, CD_2Cl_2): δ_H (ppm) = 7.37–7.32 (m, 2H), 7.31–7.27 (m, 1H), 7.22–7.18 (m, 2H), 3.66 (t, *J* = 8.6 Hz, 1H), 2.98–2.91 (m, 1H), 2.91–2.84 (m, 1H), 1.91 (dq, *J* = 14.8, 7.5 Hz, 1H), 1.75 (dq, *J* = 14.8, 7.5 Hz, 1H), 1.49 (dq, *J* = 14.8, 7.5 Hz, 1H), 1.16 (dq, *J* = 14.8, 7.5 Hz, 1H), 1.04 (t, *J* = 7.5 Hz, 3H), 0.71 (t, *J* = 7.5 Hz, 3H).

^{13}C-NMR (126 MHz, CD_2Cl_2): δ_C (ppm) = 176.0 (qC), 138.4 (qC), 128.9 (CH), 128.6 (CH), 127.8 (CH), 91.7 (qC), 47.3 (CH), 35.7 (CH_2), 28.8 (CH_2), 27.5 (CH_2), 8.1 (CH_3), 7.9 (CH_3).

HR-MS (GC-APCI, +, Q-TOF): calc. for $C_{14}H_{19}O_2$ $[M+H]^+$: 219.1380, found: 219.1378.

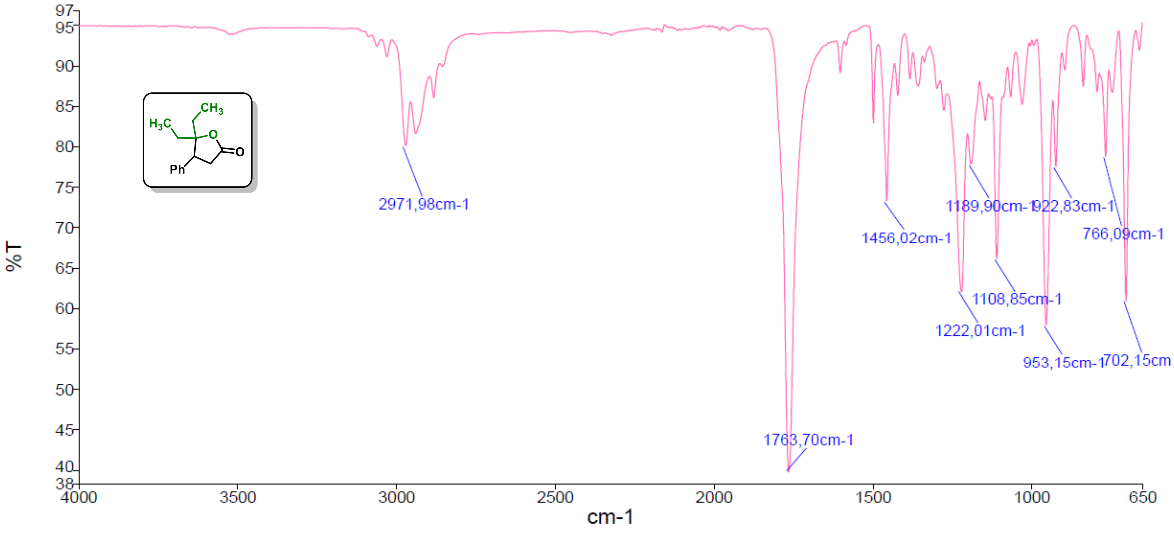

FT-IR Spectrum (ATR, thin film) of **4e**.

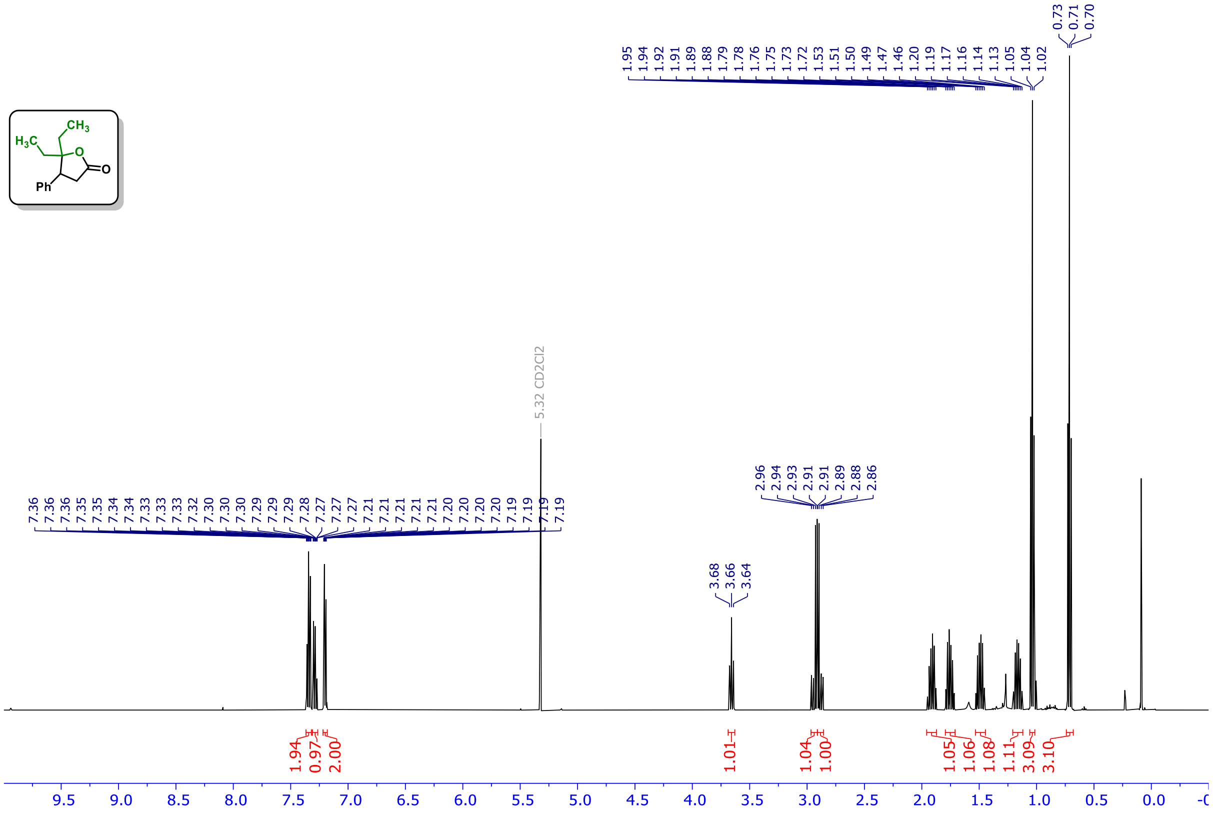

^{1}H-NMR spectrum (500 MHz, CD_2Cl_2) of **4e**.

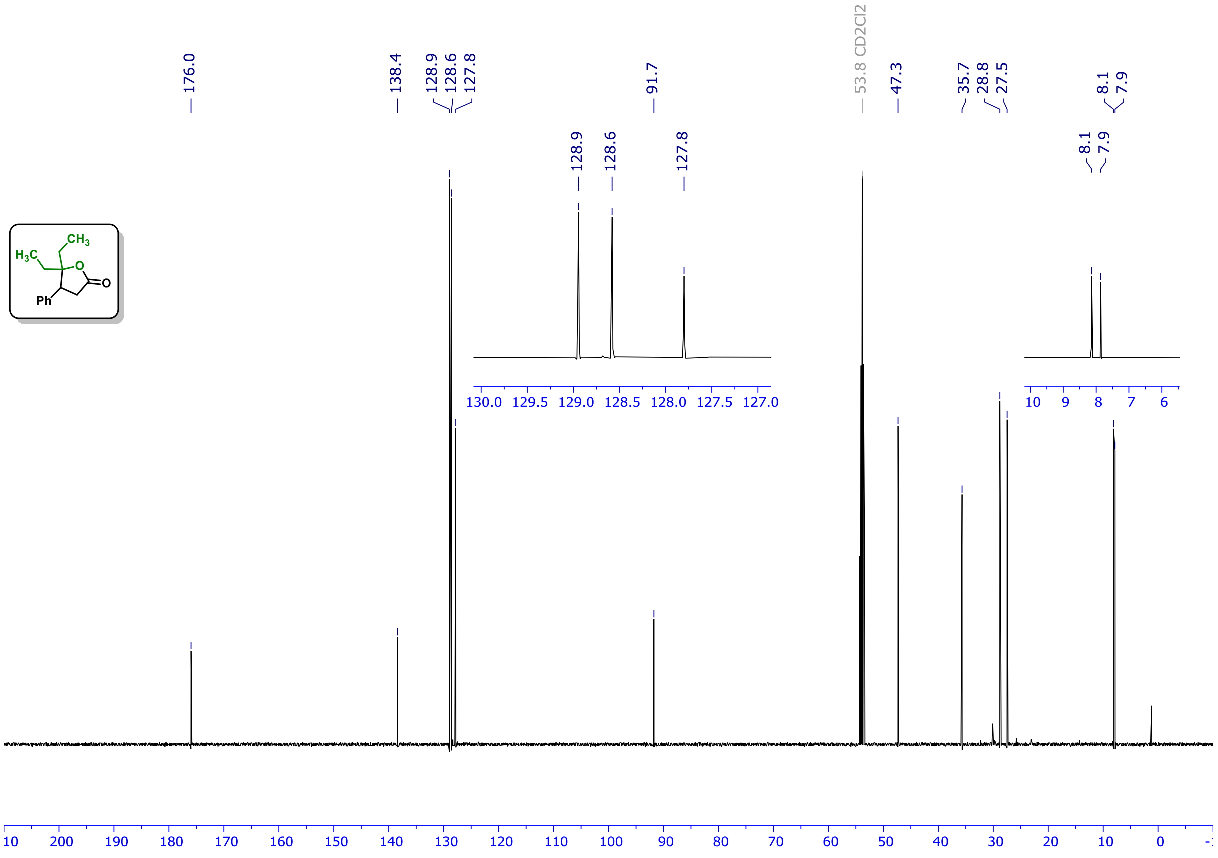

^{13}C-NMR spectrum (126 MHz, CD_2Cl_2) of **4e**.

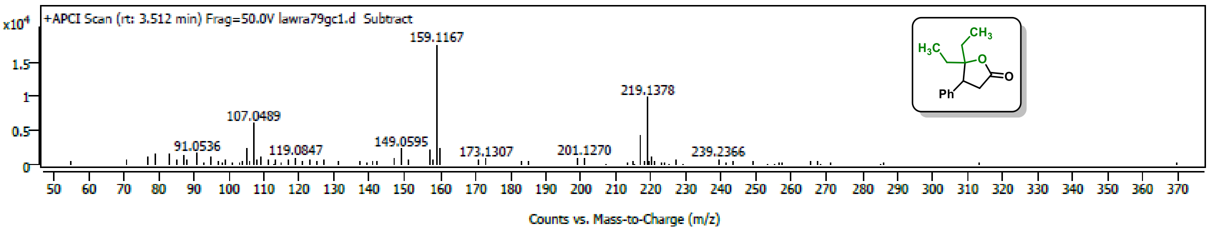

HR-MS Spectrum (APCI,+) of **4e**.

Lactone 4f

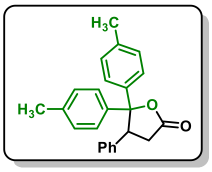

Prepared according to the **General Procedure** from SCP **1a** (54.9 mg, 0.20 mmol, 1.00 eq.) and 4,4´-dimethylbenzophenone (336 mg, 1.60 mmol, 8.00 eq.). Purification by flash column chromatography (SiO_2, *n*-pentane/acetone 20:1) afforded lactone **4f** (29.7 mg, 0.09 mmol, 43%) as a colorless solid.

FTIR (ATR): $\tilde{\nu}$ [cm^{-1}] = 1777, 1509, 1212, 1187, 1140, 978, 897, 812, 726, 700.

^{1}H-NMR (400 MHz, CD_2Cl_2): δ_H (ppm) = 7.52–7.47 (m, 2H), 7.24–7.19 (m, 2H), 7.17–7.11 (m, 3H), 7.03–6.96 (m, 2H), 6.95–6.87 (m, 4H), 4.48 (dd, *J* = 8.0, 4.7 Hz, 1H), 2.96 (dd, *J* = 17.4, 8.0 Hz, 1H), 2.73 (dd, *J* = 17.4, 4.7 Hz, 1H), 2.35 (s, 3H), 2.19 (s, 3H).

^{13}C-NMR (101 MHz, CD_2Cl_2): δ_C (ppm) = 175.9, 141.1, 139.5, 138.5, 138.0, 137.3, 129.7, 129.0, 128.7, 128.7, 127.6, 126.6, 126.3, 93.3, 51.2, 38.0, 21.1, 21.0.

HR-MS (ESI+, Orbitrap): calc. for $C_{24}H_{23}O_2$ $[M+H]^+$: 343.1693, found: 343.1689.

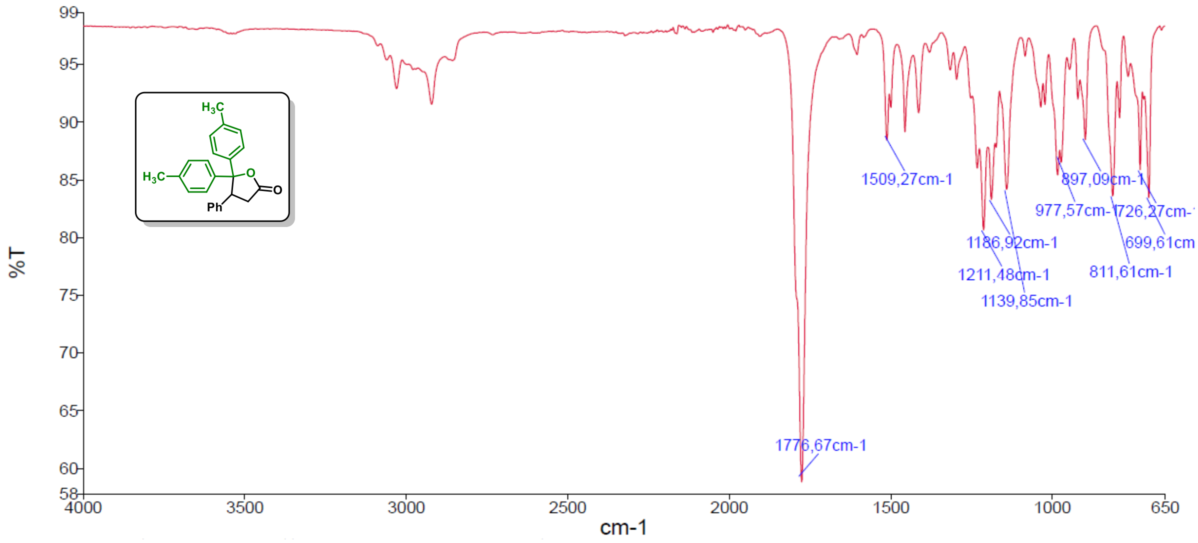

FT-IR Spectrum (ATR, thin film) of **4f**.

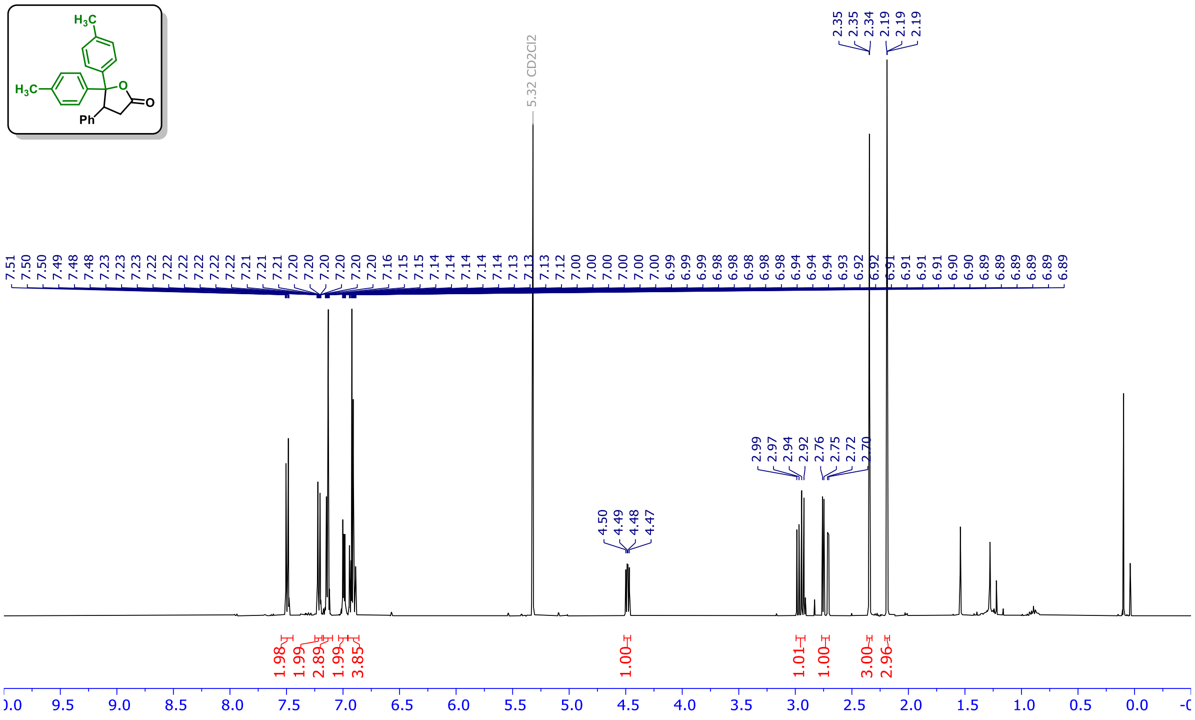

^{1}H-NMR spectrum (400 MHz, CD_2Cl_2) of **4f**.

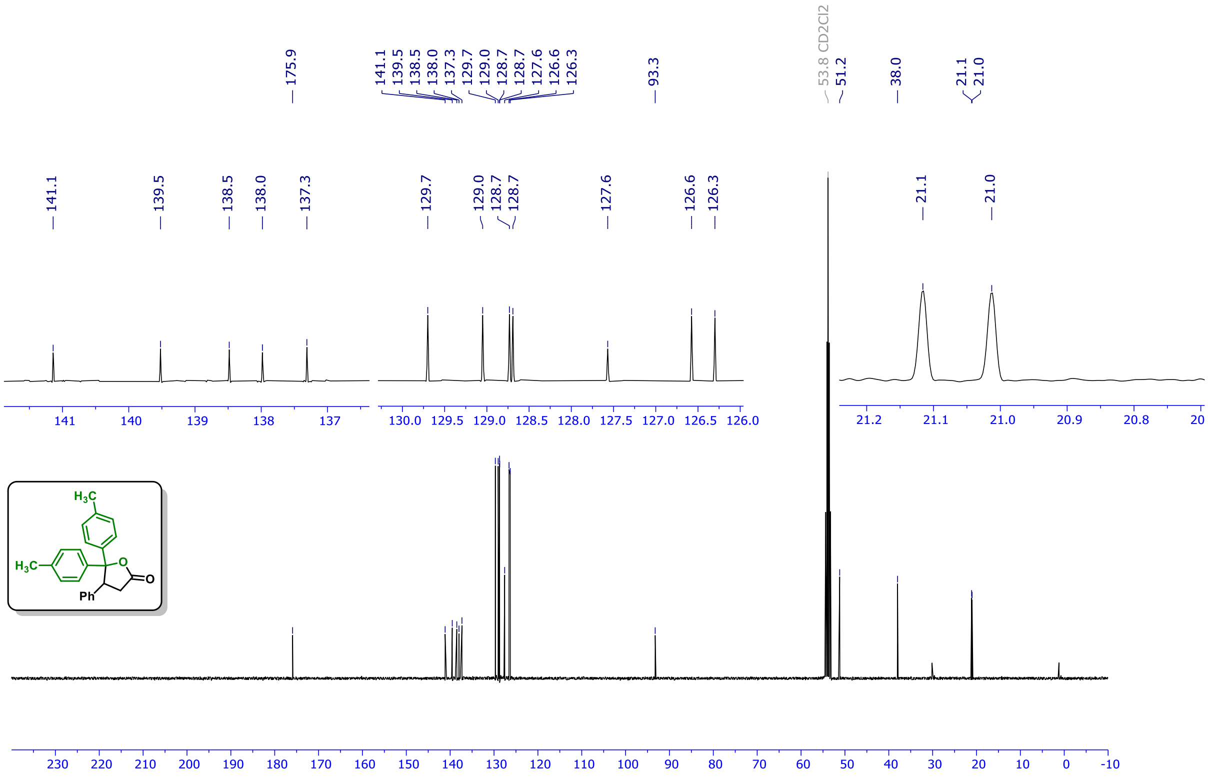

^{13}C-NMR spectrum (101 MHz, CD_2Cl_2) of **4f**.

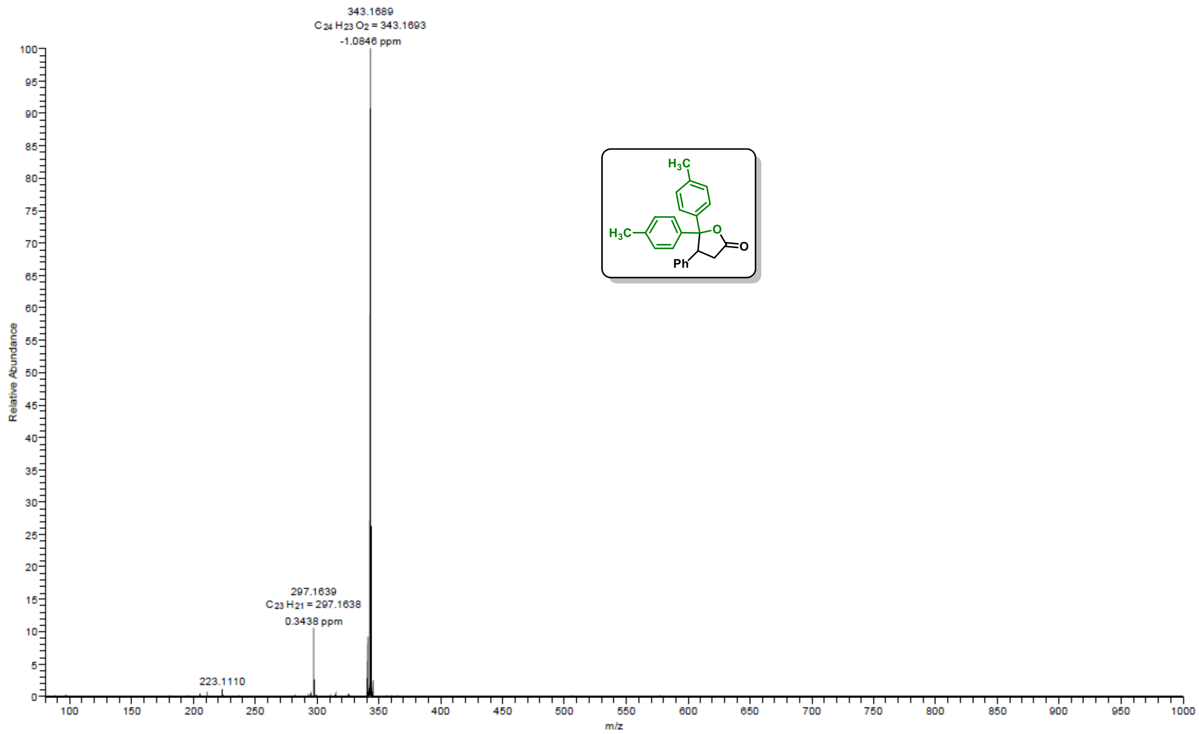

HR-MS Spectrum (ESI+) of **4f**.

Lactone 4g

Prepared according to the **General Procedure** from SCP **1a** (54.9 mg, 0.20 mmol, 1.00 eq.) and 4,4´-bis(4-trifluoromethyl)benzophenone (509 mg, 1.60 mmol, 8.00 eq.). Purification by flash column chromatography (SiO_2, *n*-pentane/EtOAc 20:1 to 2:1) afforded lactone **4g** (57.6 mg, 0.13 mmol, 63%) as a colorless solid.

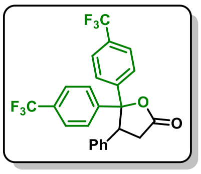

FTIR (ATR): $\tilde{\nu}$ [cm^{-1}] = 1795, 1781, 1324, 1167, 1121, 1070, 1017, 988, 836, 700.

^{1}H-NMR (700 MHz, CD_2Cl_2): δ_H (ppm) = 7.89–7.78 (m, 2H), 7.74–7.61 (m, 2H), 7.39–7.35 (m, 2H), 7.27–7.21 (m, 2H), 7.19–7.10 (m, 3H), 7.03–6.96 (m, 2H), 4.54 (dd, *J* = 8.1, 3.6 Hz, 1H), 3.01 (dd, *J* = 17.6, 8.1 Hz, 1H), 2.82 (dd, *J* = 17.6, 3.6 Hz, 1H).

^{13}C-NMR (176 MHz, CD_2Cl_2): δ_C (ppm) = 174.9 (qC), 147.0 (qC), 144.1 (qC), 138.5 (qC), 130.9 (qC, q, *J* = 32.7 Hz), 129.8 (qC, q, *J* = 32.4 Hz), 129.1 (CH), 128.8 (CH), 128.1 (CH), 127.0 (CH), 127.0 (CH), 126.4 (CH, q, *J* = 3.8 Hz), 125.3 (CH, q, *J* = 3.8 Hz), 124.3 (qC, q, *J* = 272 Hz), 124.3 (qC, q, *J* = 272 Hz), 92.1 (qC), 51.1 (CH), 37.6 (CH_2).

^{19}F-NMR (659 MHz, CD_2Cl_2): δ_F (ppm) = −63.1.

HR-MS (ESI−, Orbitrap): calc. for $C_{24}H_{16}F_6ClO_2$ $[M+Cl]^-$: 485.0748, found: 485.0749.

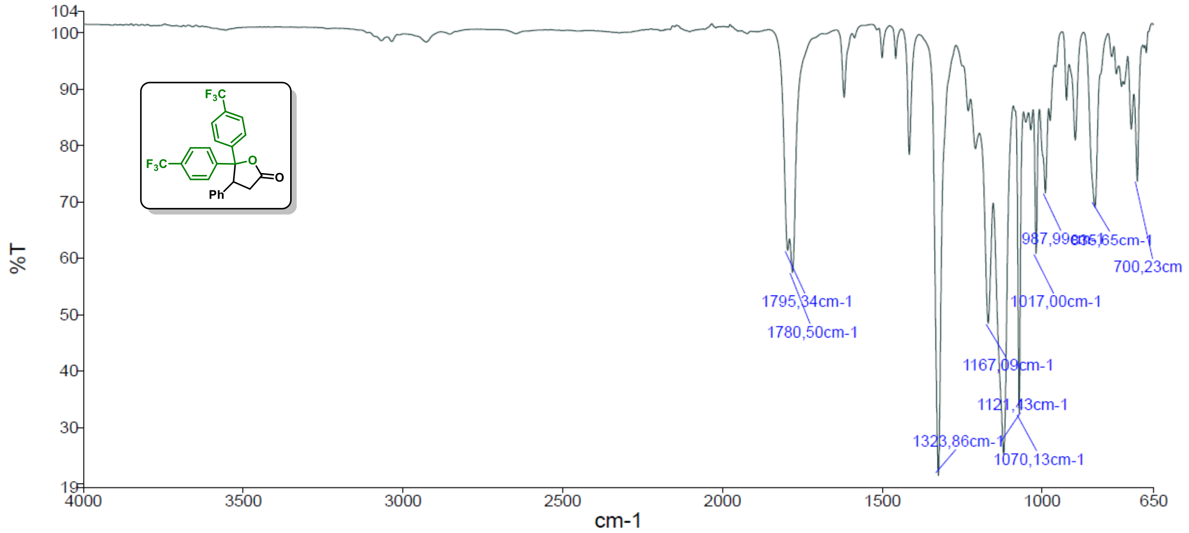

FT-IR Spectrum (ATR, thin film) of **4g**.

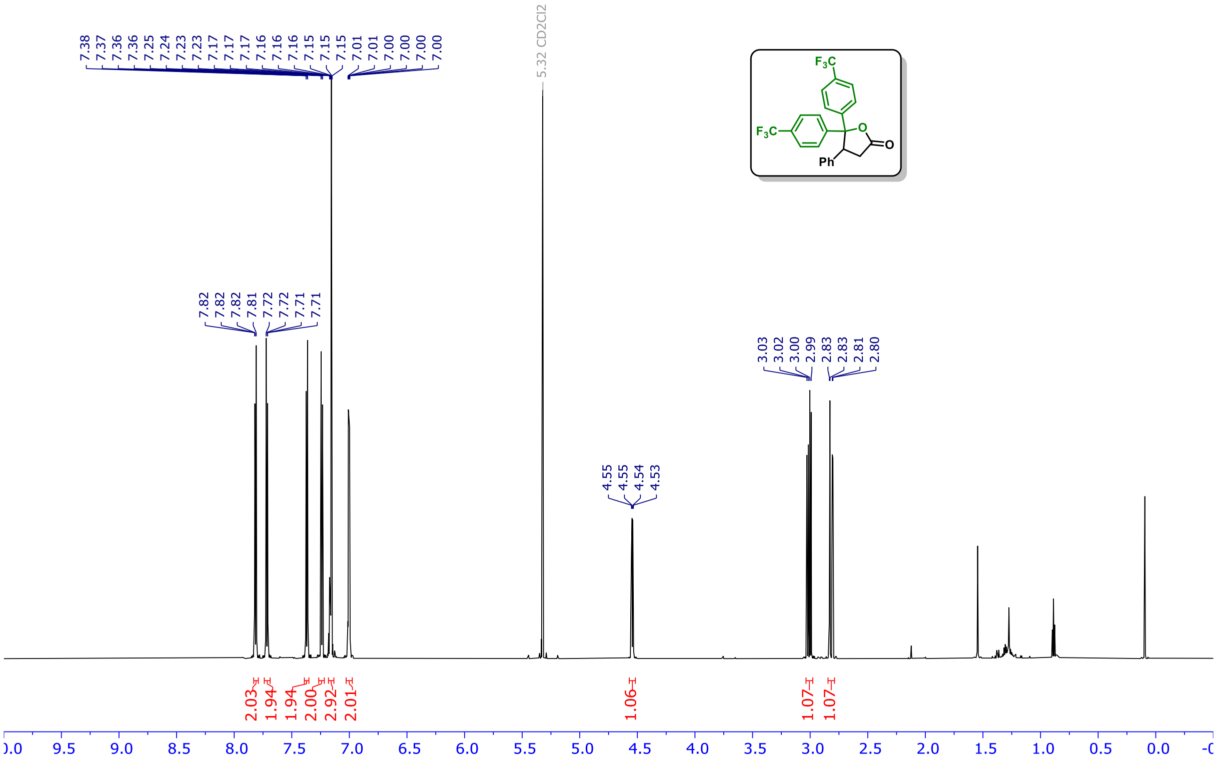

^{1}H-NMR spectrum (700 MHz, CD_2Cl_2) of **4g**.

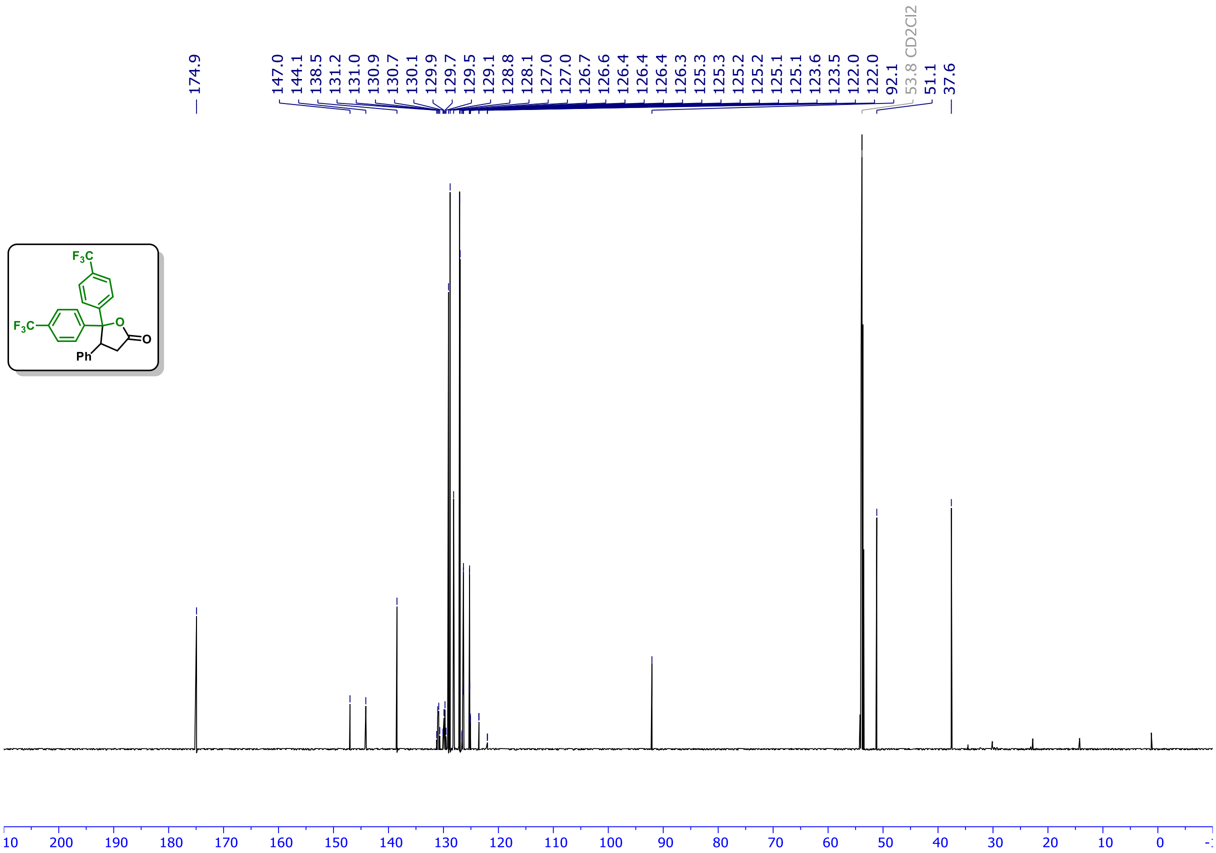

^{13}C-NMR spectrum (176 MHz, CD_2Cl_2) of **4g**.

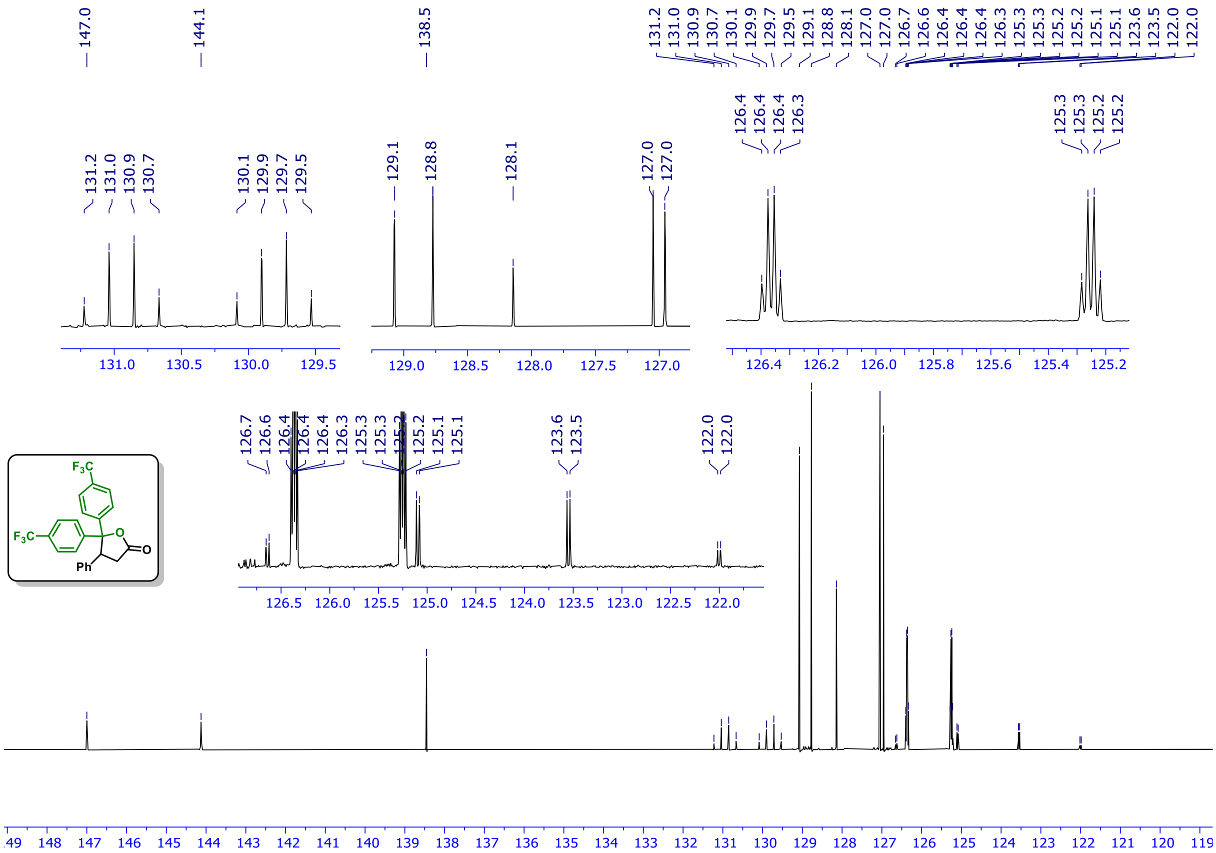

Excerpt of ^{13}C-NMR spectrum (176 MHz, CD_2Cl_2) of **4g**.

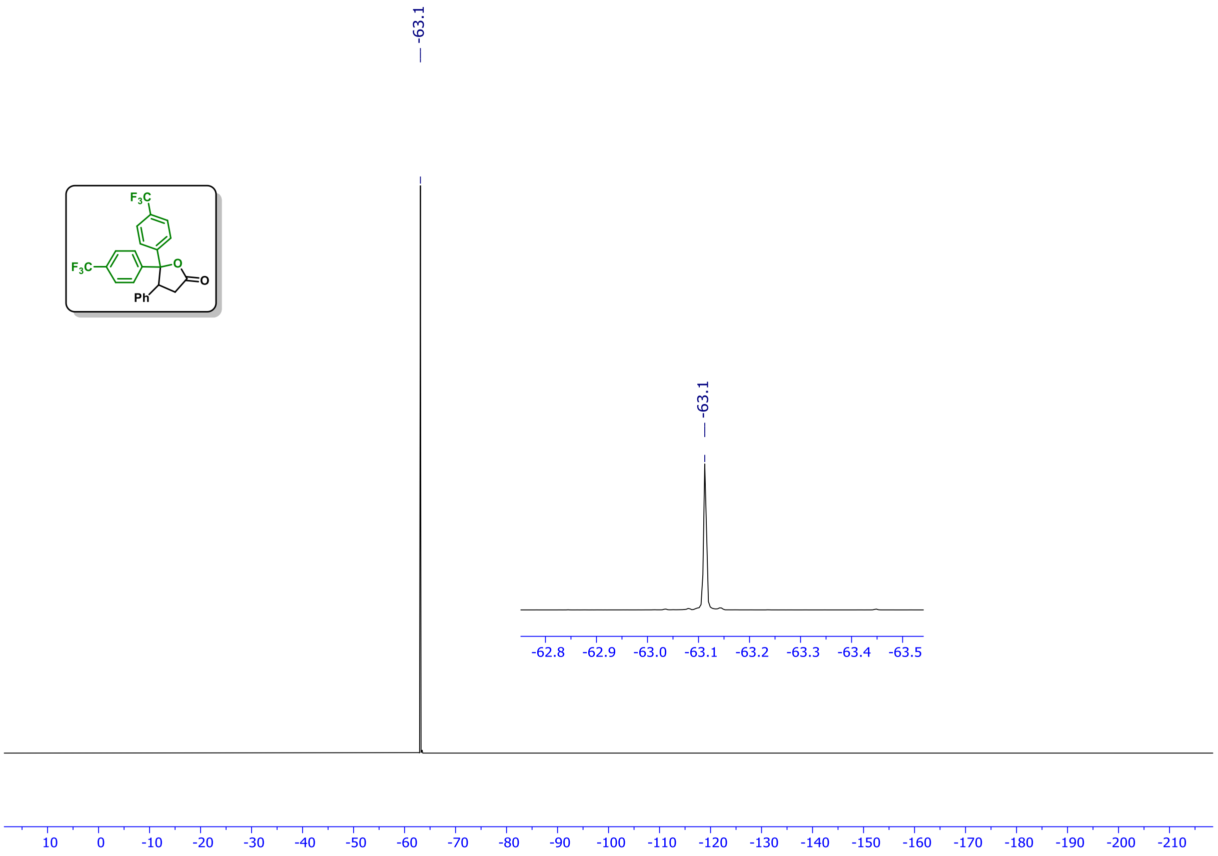

^{19}F-NMR spectrum (659 MHz, CD_2Cl_2) of **4g**.

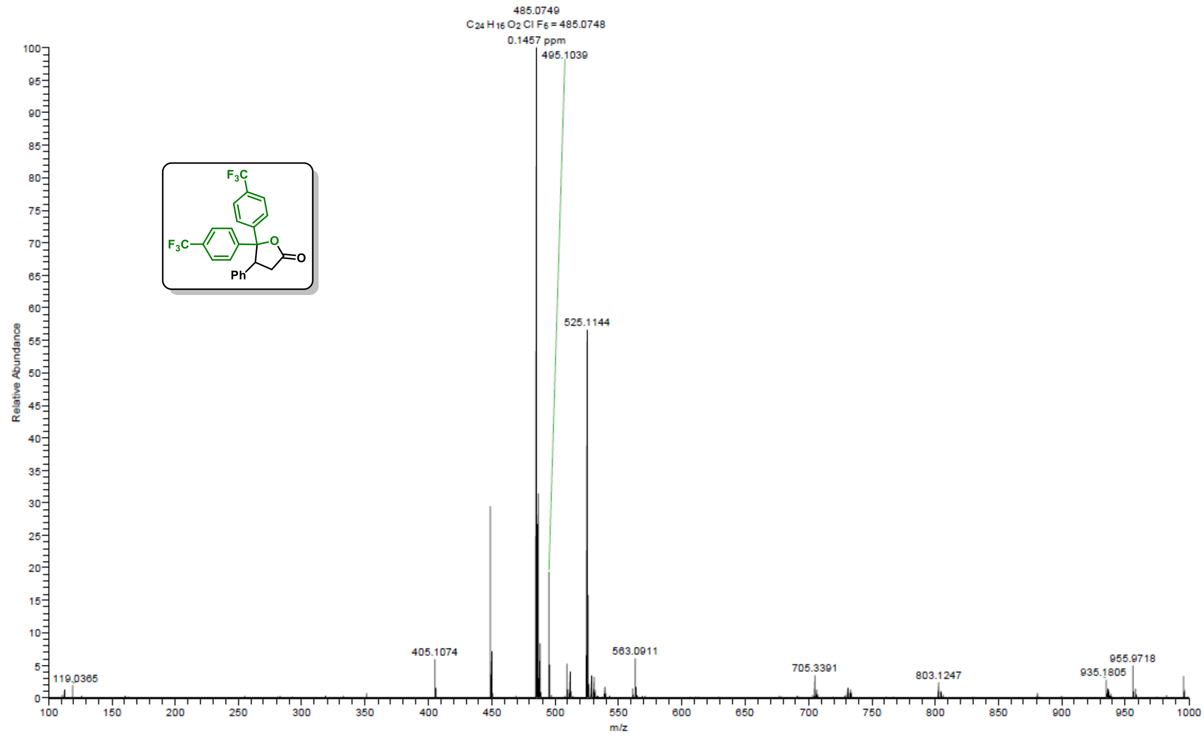

HR-MS Spectrum (ESI−) of **4g**.

Lactone 4h

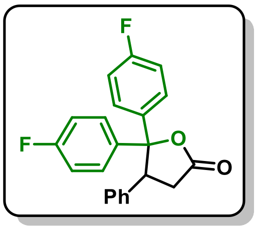

Prepared according to the **General Procedure** from SCP **1a** (54.9 mg, 0.20 mmol, 1.00 eq.) and 4,4´-difluorobenzophenone (349 mg, 1.60 mmol, 8.00 eq.). Purification by flash column chromatography (SiO_2, *n*-pentane/EtOAc 20:1) afforded lactone **4h** (48.7 mg, 0.14 mmol, 70%) as a colorless solid.

FTIR (ATR): $\tilde{\nu}$ [cm^{-1}] = 1767, 1507, 1233, 1199, 1142, 983, 837, 822, 729, 697.

^{1}H-NMR (500 MHz, CD_2Cl_2): δ_H (ppm) = 7.66–7.60 (m, 2H), 7.19–7.09 (m, 5H), 7.04–6.98 (m, 2H), 6.98–6.93 (m, 2H), 6.82–6.75 (m, 2H), 4.49 (dd, *J* = 8.0, 4.6 Hz, 1H), 2.99 (dd, *J* = 17.6, 8.0 Hz, 1H), 2.81 (dd, *J* = 17.6, 4.6 Hz, 1H).

^{13}C-NMR (126 MHz, CD_2Cl_2): δ_C (ppm) = 175.4 (qC), 162.9 (qC, d, *J* = 247 Hz), 162.1 (qC, d, *J* = 246 Hz), 139.4 (qC, d, *J* = 3.3 Hz), 138.7 (qC), 136.5 (qC, d, *J* = 3.4 Hz), 128.9 (CH), 128.8 (CH), 128.6 (CH, d, *J* = 4.4 Hz), 128.5 (CH, d, *J* = 4.3 Hz), 127.9 (CH), 116.0 (d, *J* = 21.8 Hz), 114.9 (d, *J* = 21.8 Hz), 92.4 (qC), 51.2 (CH), 37.5 (CH_2).

^{19}F-NMR (471 MHz, CD_2Cl_2): δ_F (ppm) = −114.3 (quin, *J* = 6.6 Hz), −115.6 (quin, *J* = 7.5 Hz).

HR-MS (ESI+, Orbitrap): calc. for $C_{22}H_{17}O_2F_2$ $[M+H]^+$: 351.1191, found: 351.1185.

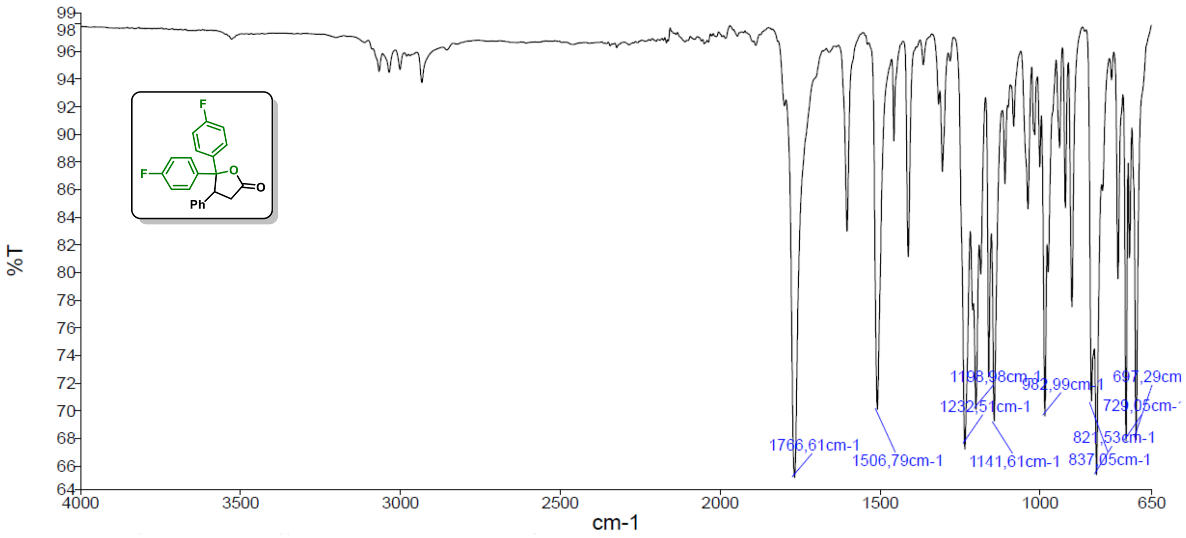

FT-IR Spectrum (ATR, thin film) of **4h**.

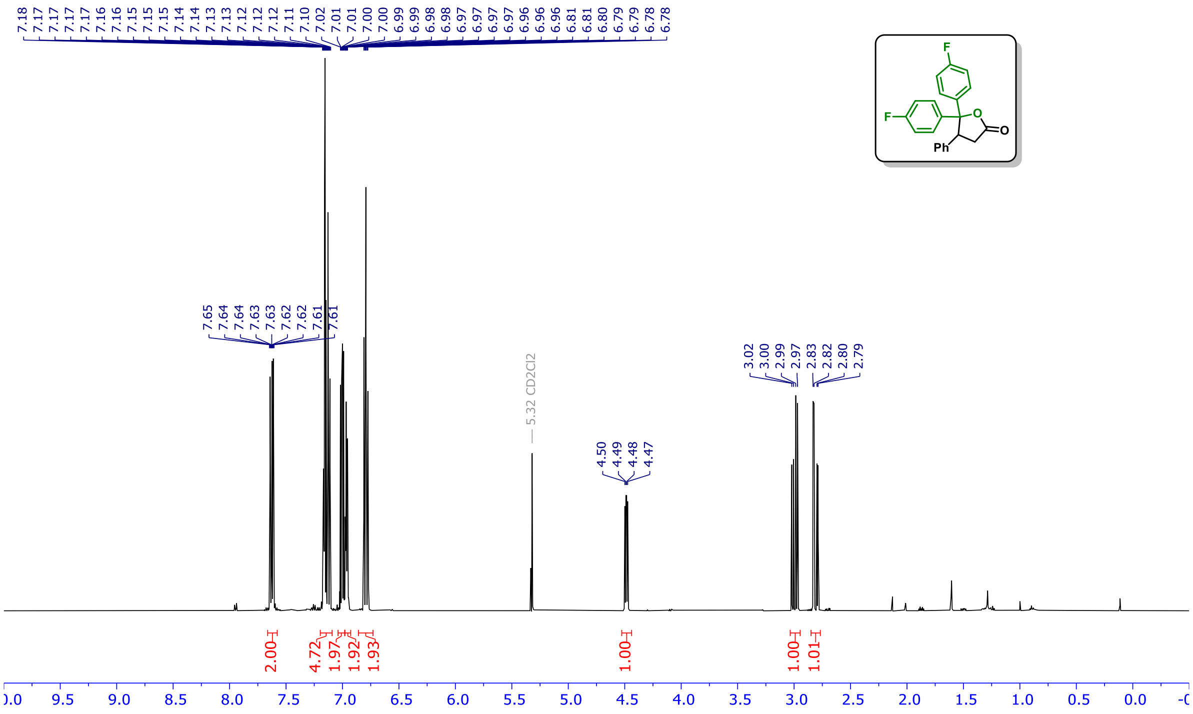

^{1}H-NMR spectrum (500 MHz, CD_2Cl_2) of **4h**.

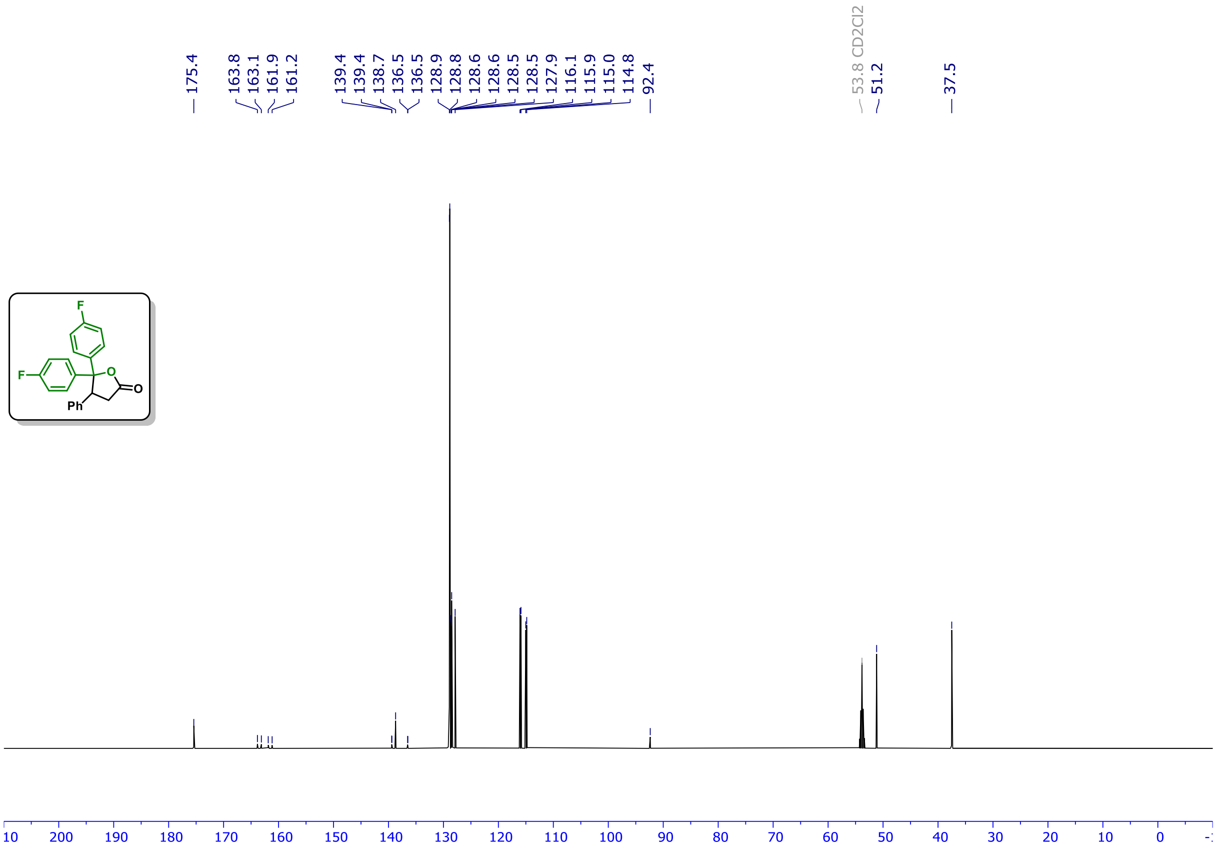

^{13}C-NMR spectrum (126 MHz, CD_2Cl_2) of **4h**.

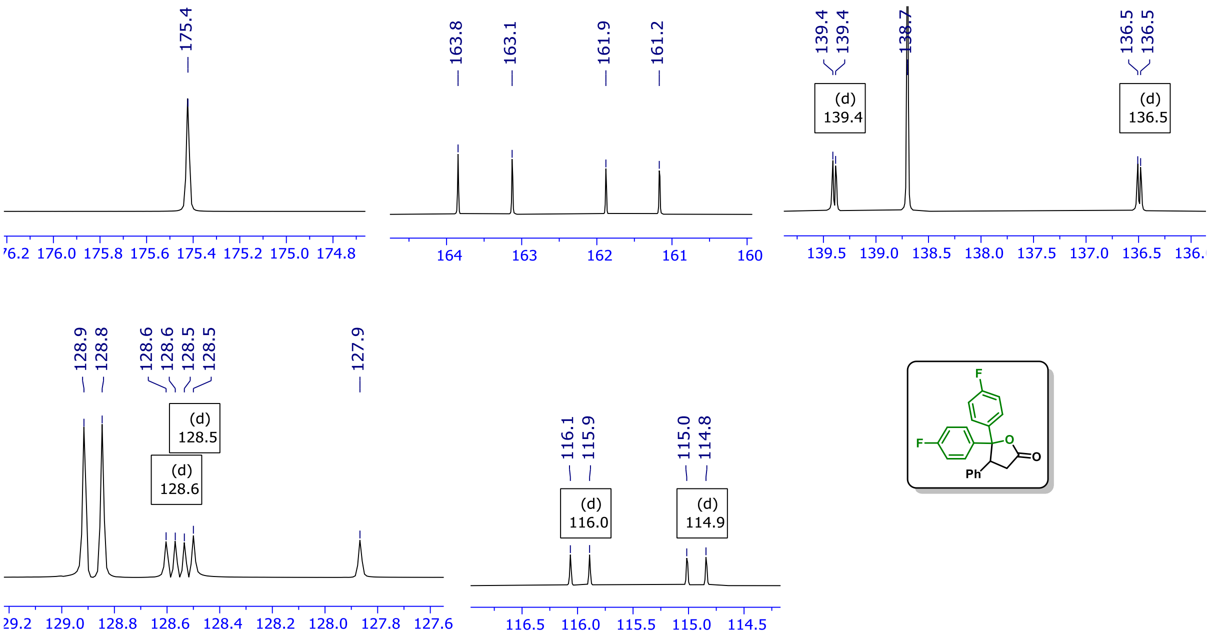

Excerpt of the ^{13}C-NMR spectrum (126 MHz, CD_2Cl_2) of **4h**.

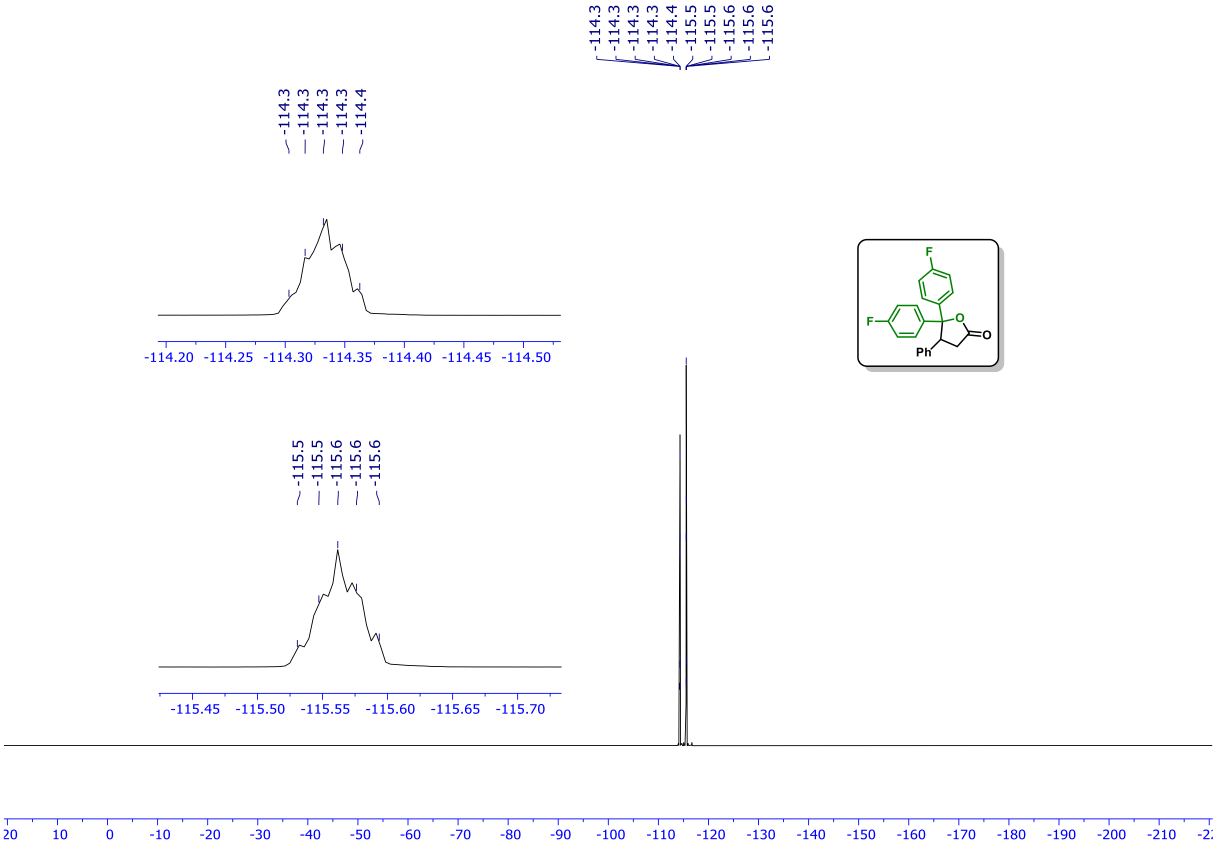

^{19}F-NMR spectrum (471 MHz, CD_2Cl_2) of **4h**.

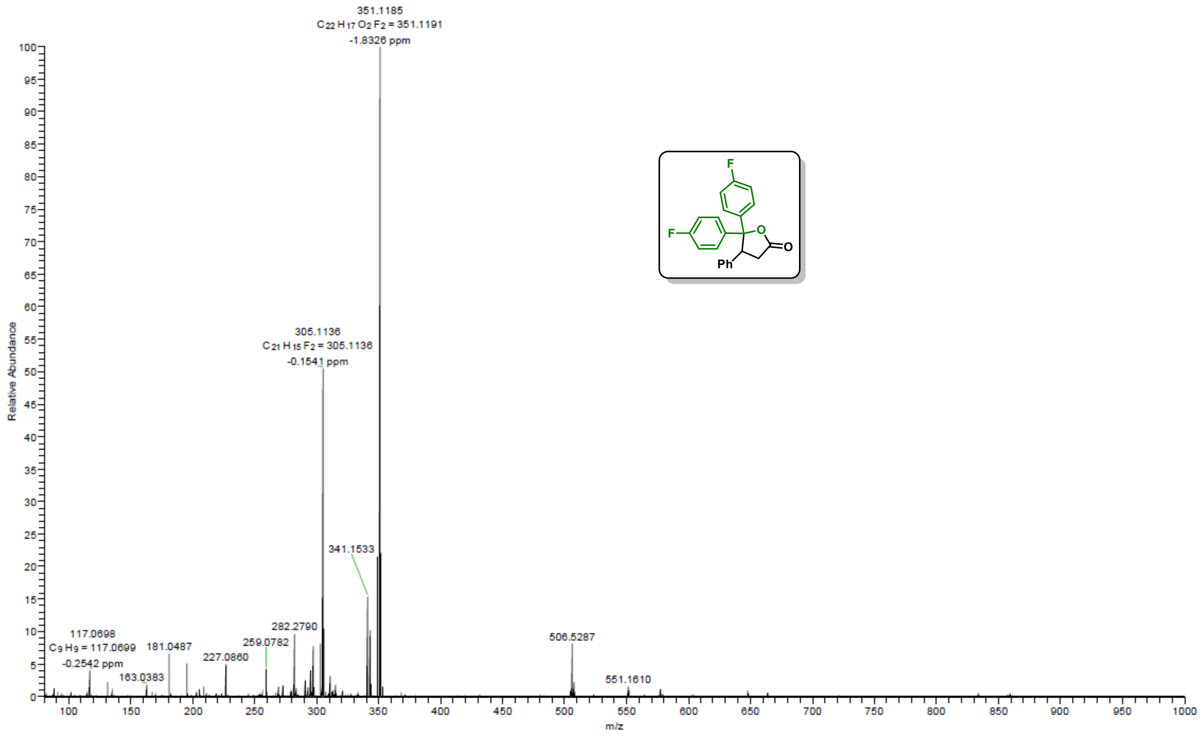

HR-MS Spectrum (ESI+) of **4h**.

Lactone 4i

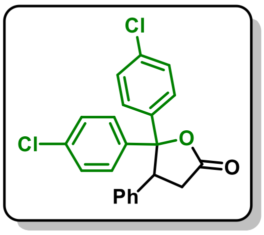

Prepared according to the **General Procedure** from SCP **1a** (54.9 mg, 0.20 mmol, 1.00 eq.) and 4,4´-dichlorobenzophenone (402 mg, 1.60 mmol, 8.00 eq.). Purification by flash column chromatography (SiO_2, *n*-pentane/EtOAc 10:1) afforded lactone **4i** (50.2 mg, 0.13 mmol, 65%) as a colorless solid.

FTIR (ATR): $\tilde{\nu}$ [cm^{-1}] = 1780, 1491, 1204, 1139, 1094, 1013, 983, 894, 819, 701.

^{1}H-NMR (500 MHz, CD_2Cl_2): δ_H (ppm) = 7.60–7.54 (m, 2H), 7.54–7.48 (m, 2H), 7.27–7.21 (m, 2H), 7.19–7.14 (m, 3H), 7.01–6.96 (m, 2H), 6.95–6.88 (m, 2H), 4.45 (dd, *J* = 8.1, 4.2 Hz, 1H), 2.98 (dd, *J* = 17.6, 8.1 Hz, 1H), 2.78 (dd, *J* = 17.6, 4.2 Hz, 1H).

^{13}C-NMR (126 MHz, CD_2Cl_2): δ_C (ppm) = 175.2 (qC), 142.3 (qC), 139.4 (qC), 138.6 (qC), 132.3 (CH), 131.3 (CH), 128.9 (CH), 128.8 (CH), 128.4 (CH), 128.2 (CH), 128.0 (CH), 122.9 (qC), 121.8 (qC), 92.2 (qC), 50.9 (CH), 37.5 (CH_2).

HR-MS (ESI−, Orbitrap): calc. for $C_{22}H_{16}O_2Cl_3$ $[M+Cl]^-$: 417.0221, found: 417.0220.

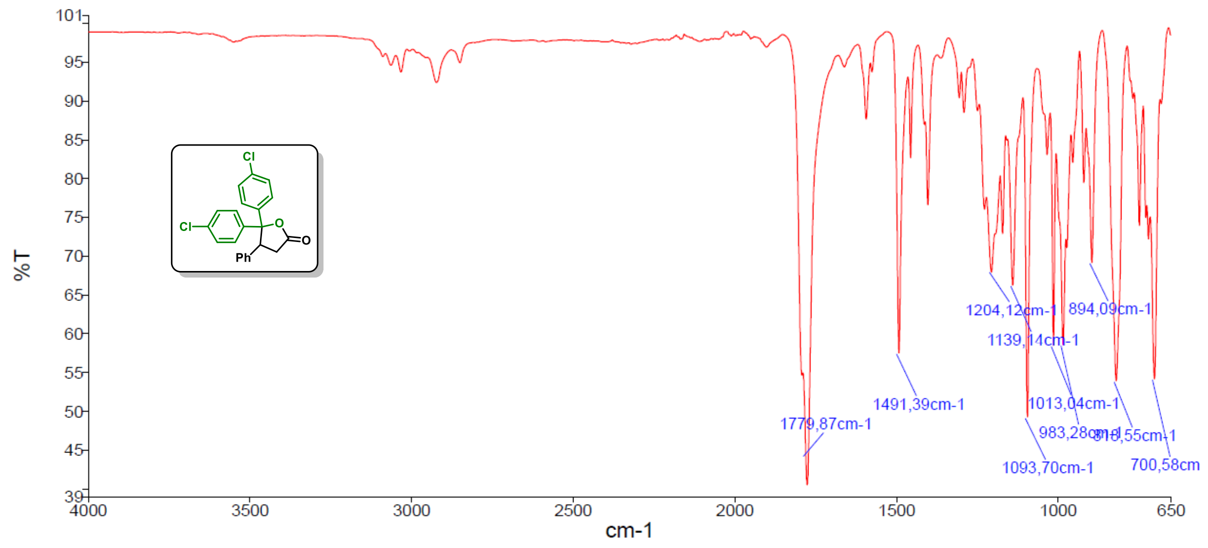

FT-IR Spectrum (ATR, thin film) of **4i**.

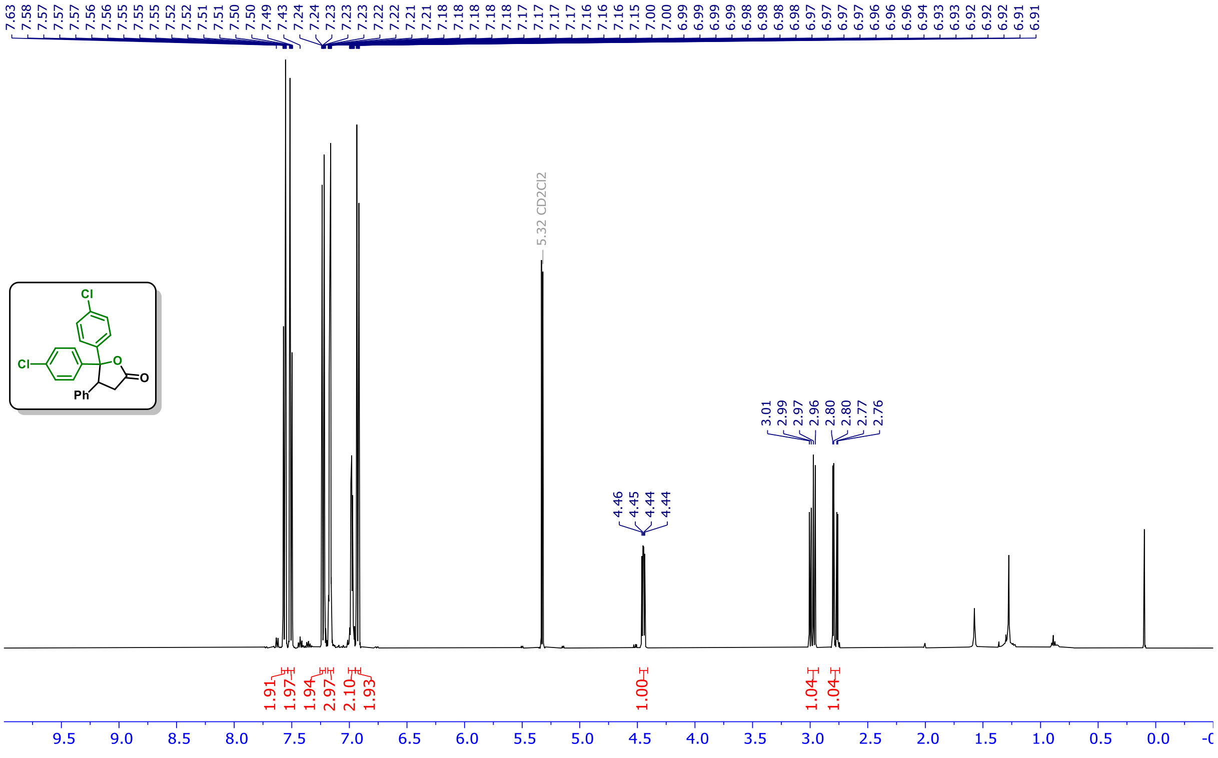

^{1}H-NMR spectrum (500 MHz, CD_2Cl_2) of **4i**.

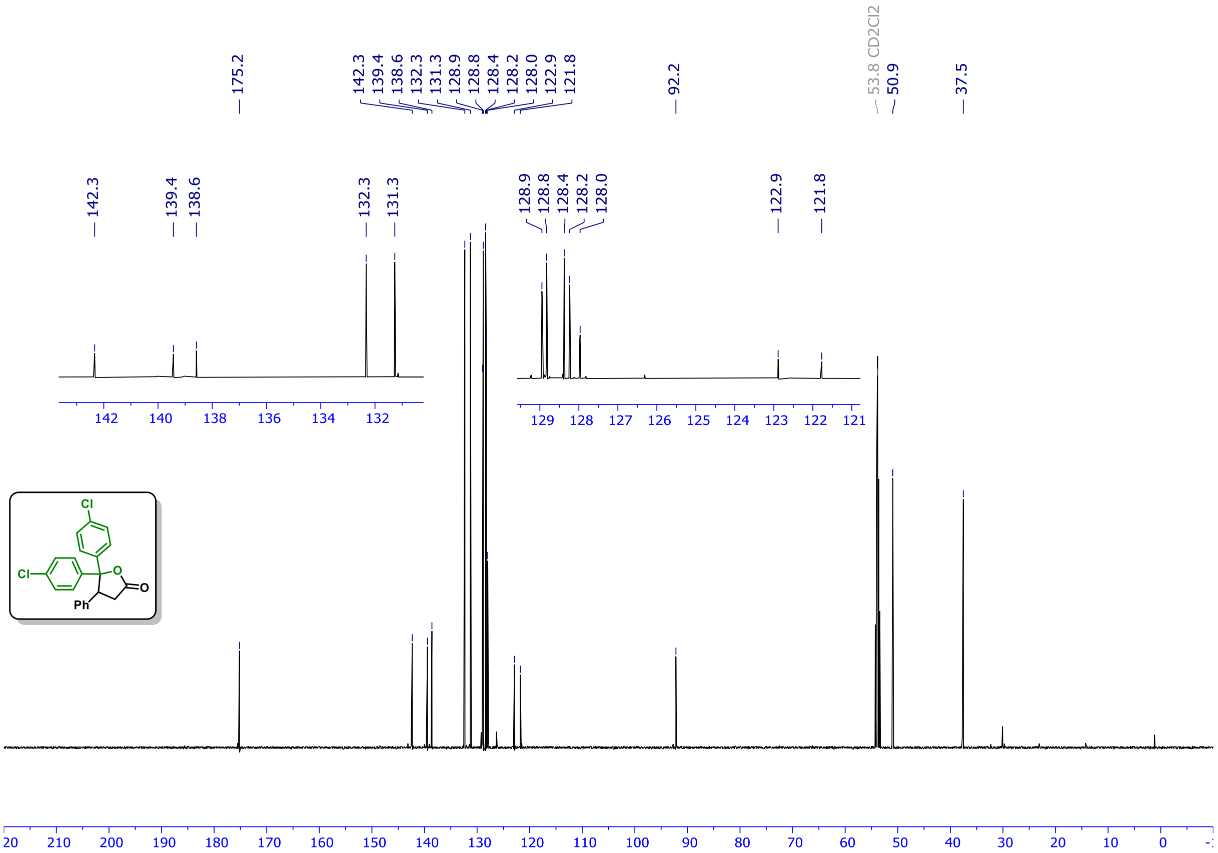

^{13}C-NMR spectrum (126 MHz, CD_2Cl_2) of **4i**.

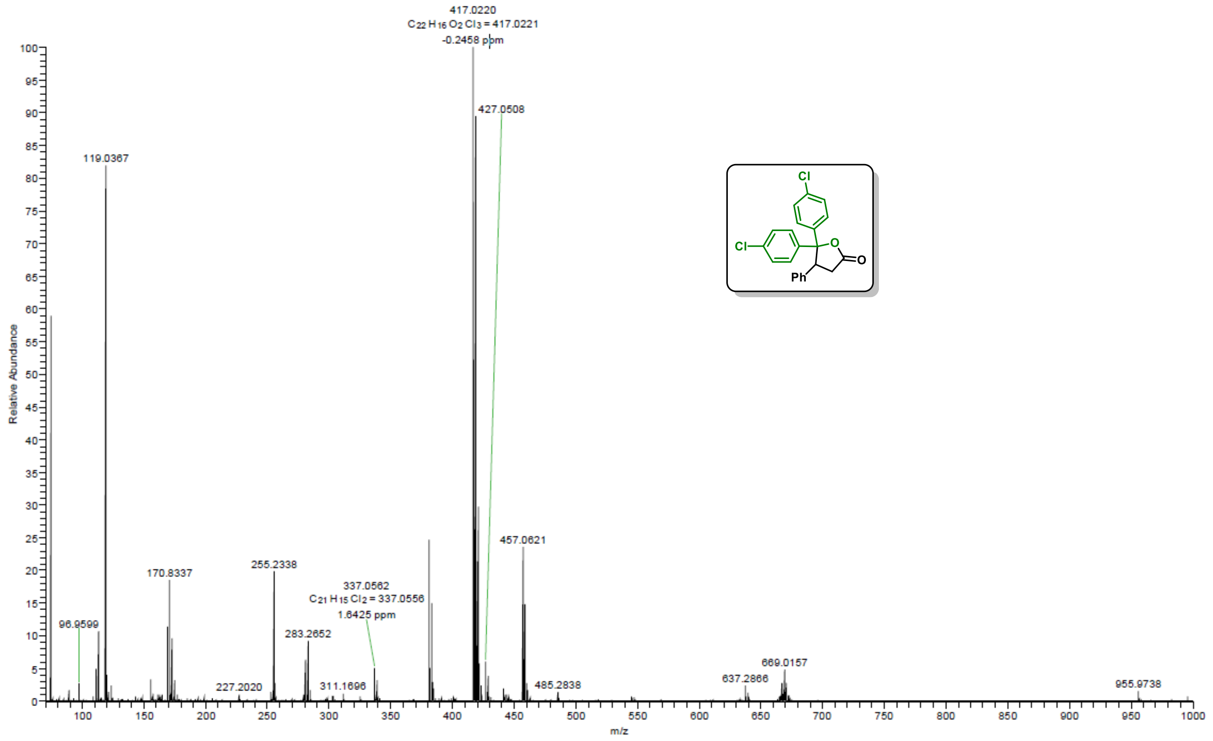

HR-MS Spectrum (ESI−) of **4i**.

Lactone 4j

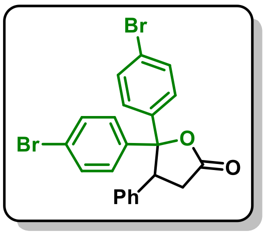

Prepared according to the **General Procedure** from SCP **1a** (54.9 mg, 0.20 mmol, 1.00 eq.) and 4,4´-dibromobenzophenone (544 mg, 1.60 mmol, 8.00 eq.). Purification by flash column chromatography (SiO_2, *n*-pentane/EtOAc 30:1) afforded lactone **4f** (62.3 mg, 0.13 mmol, 66%) as a colorless oil.

FTIR (ATR): $\tilde{\nu}$ [cm^{-1}] = 1780, 1487, 1205, 1139, 1075, 1009, 983, 894, 814, 700.

^{1}H-NMR (500 MHz, CD_2Cl_2): δ_H (ppm) = 7.62–7.53 (m, 2H), 7.45–7.37 (m, 2H), 7.20–7.13 (m, 3H), 7.11–7.05 (m, 2H), 7.02–6.91 (m, 4H), 4.46 (dd, *J* = 8.0, 4.3 Hz, 1H), 2.99 (dd, *J* = 17.6, 8.0 Hz, 1H), 2.79 (dd, *J* = 17.6, 4.3 Hz, 1H).

^{13}C-NMR (126 MHz, CD_2Cl_2): δ_C (ppm) = 175.2 (qC), 141.9 (qC), 139.0 (qC), 138.6 (qC), 134.7 (qC), 133.6 (qC), 129.3 (CH), 128.9 (CH), 128.8 (CH), 128.3 (CH), 128.1 (CH), 128.0 (CH), 128.0 (CH), 92.1 (qC), 51.0 (CH), 37.5 (CH_2).

HR-MS (ESI−, Orbitrap): calc. for $C_{22}H_{16}O_2Br_2Cl$ [M+Cl]$^-$: 506.9191, found: 506.9188.

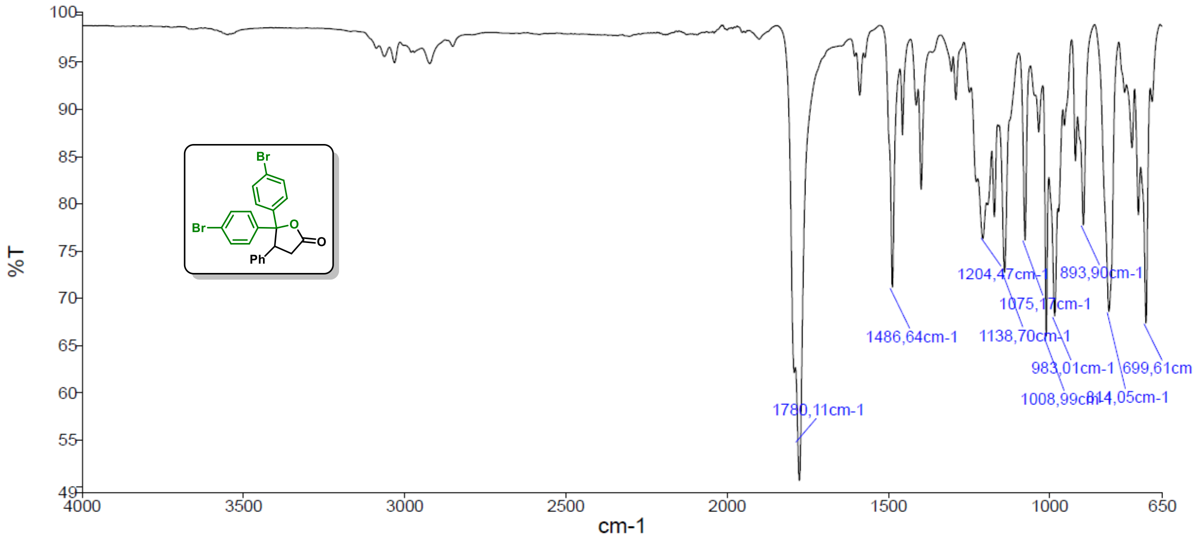

FT-IR Spectrum (ATR, thin film) of **4j**.

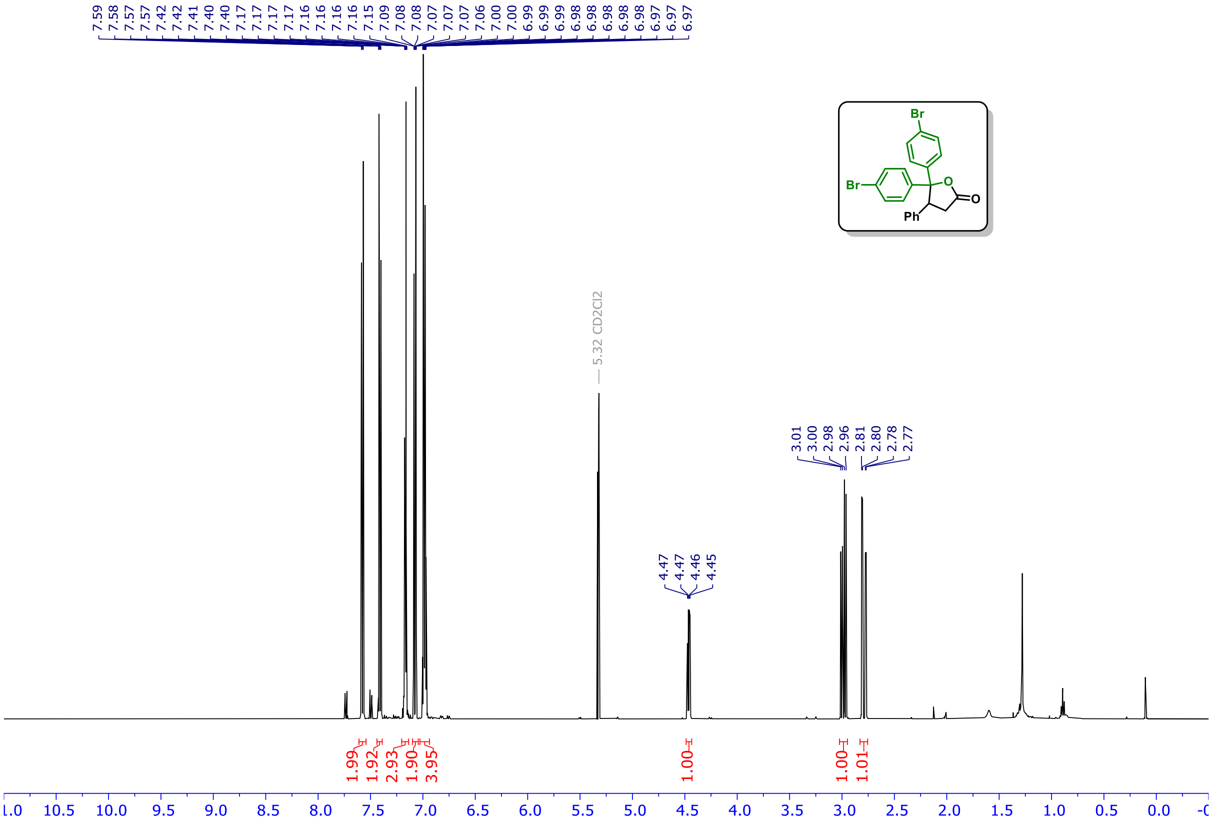

^{1}H-NMR spectrum (500 MHz, CD_2Cl_2) of **4j**.

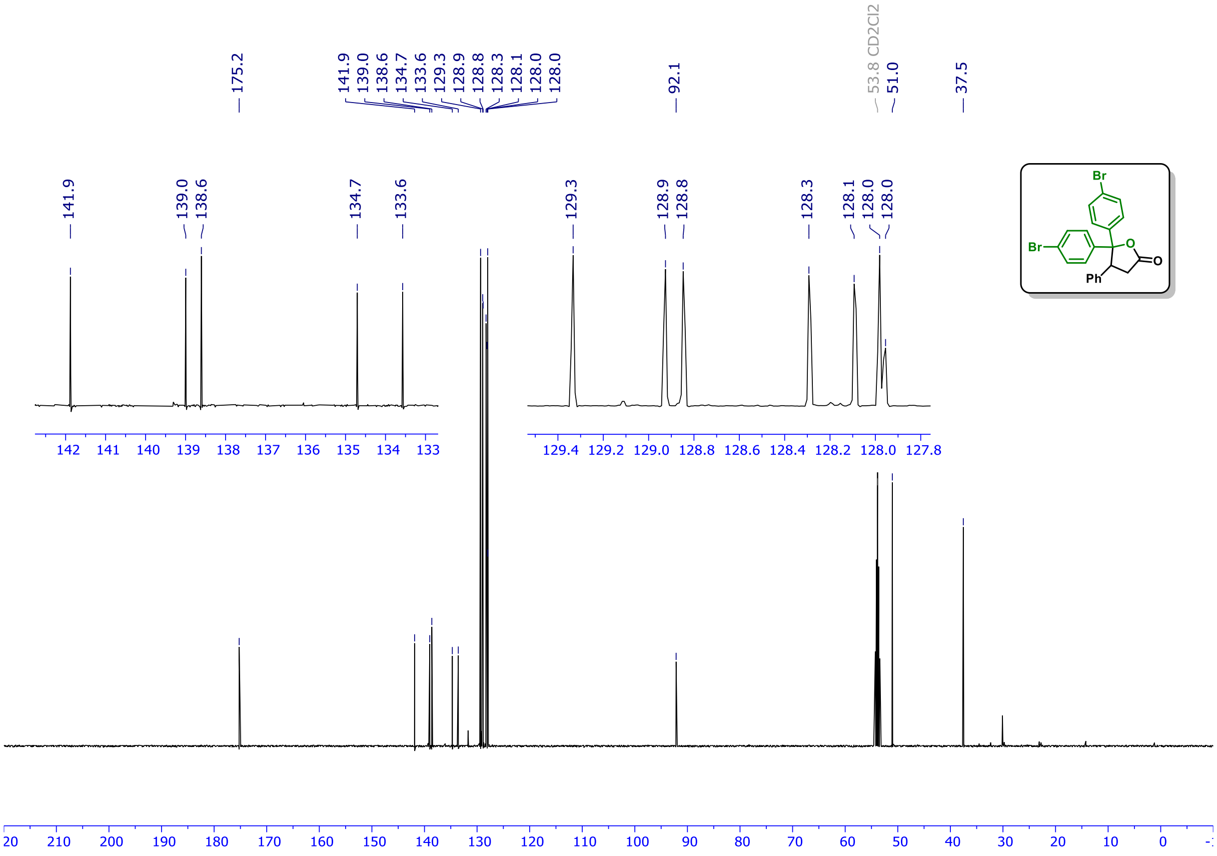

^{13}C-NMR spectrum (126 MHz, CD_2Cl_2) of **4j**.

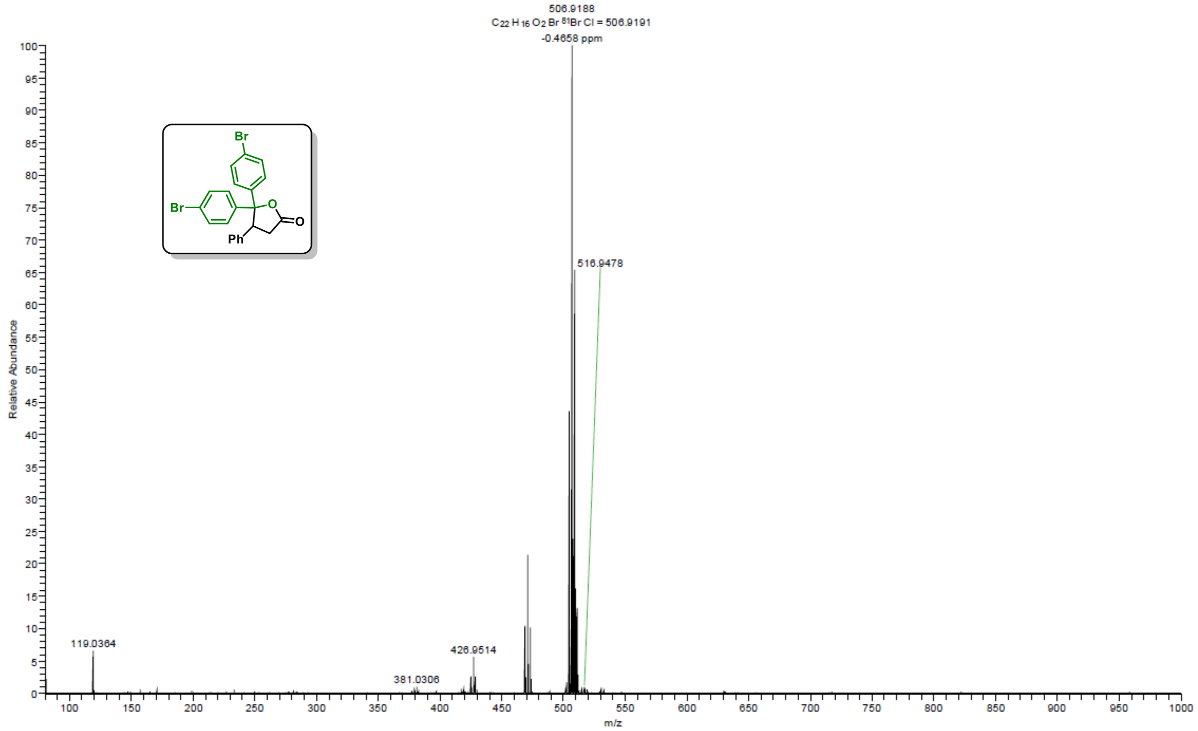

HR-MS Spectrum (ESI−) of **4j**.

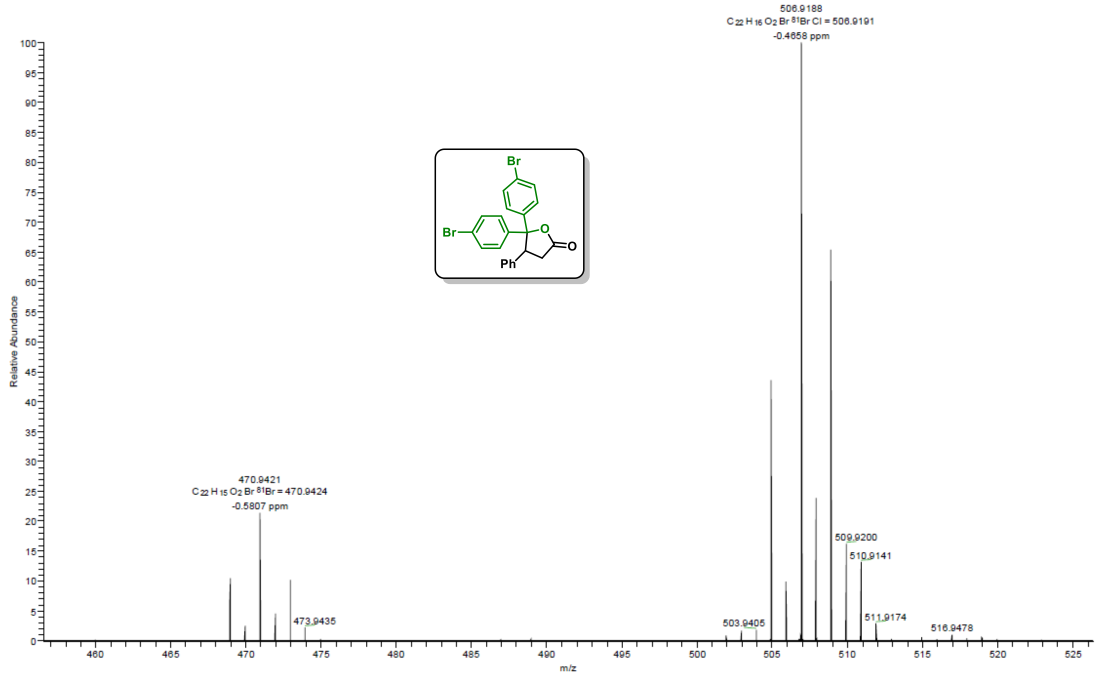

Excerpt of HR-MS Spectrum (ESI−) of **4j**.

Lactone 4k

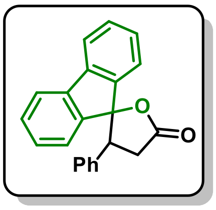

Prepared according to the **General Procedure** from SCP **1a** (54.9 mg, 0.20 mmol, 1.00 eq.) and 9-fluorenone (288 mg, 1.60 mmol, 8.00 eq.). Purification by flash column chromatography (SiO_2, *n*-pentane/acetone 10:1) afforded lactone **4k** (17.0 mg, 0.05 mmol, 27%) as

a colorless solid.

FTIR (ATR): $\tilde{\nu}$ [cm^{-1}] = 1777, 1604, 1496, 1450, 1209, 981, 920, 775, 742, 965.

^{1}H-NMR (500 MHz, CD_2Cl_2): δ_H (ppm) = 7.74–7.70 (m, 1H), 7.60–7.57 (m, 1H), 7.51–7.42 (m, 3H), 7.33 (dt, J = 7.6, 0.9 Hz, 1H), 7.26 (td, J = 7.5, 1.1 Hz, 1H), 7.16 (td, J = 7.5, 1.1 Hz, 1H), 7.06–6.93 (m, 3H), 6.74–6.69 (m, 2H), 4.44 (dd, J = 13.0, 8.4 Hz, 1H), 3.61 (dd, J = 17.5, 13.0 Hz, 1H), 3.19 (dd, J = 17.5, 8.4 Hz, 1H).

^{13}C-NMR (126 MHz, CD_2Cl_2): δ_C (ppm) = 175.7 (qC), 144.3 (qC), 142.6 (qC), 141.1 (qC), 140.6 (qC), 134.8 (qC), 130.8 (CH), 130.2 (CH), 128.9 (CH), 128.3 (CH), 128.0 (CH), 127.8 (CH), 127.6 (CH), 125.0 (CH), 124.3 (CH), 120.6 (CH), 120.6 (CH), 94.3 (qC), 51.0 (CH), 34.1 (CH_2).

HR-MS (ESI+, Orbitrap): calc. for $C_{22}H_{17}O_2$ $[M+H]^+$: 313.1223, found: 313.1227.

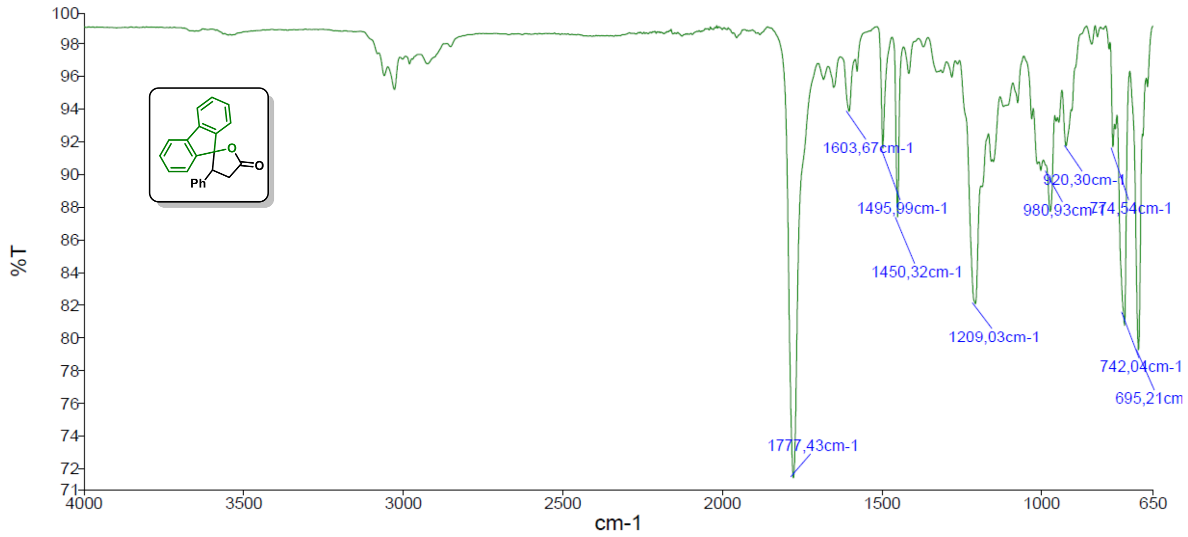

FT-IR Spectrum (ATR, thin film) of **4k**.

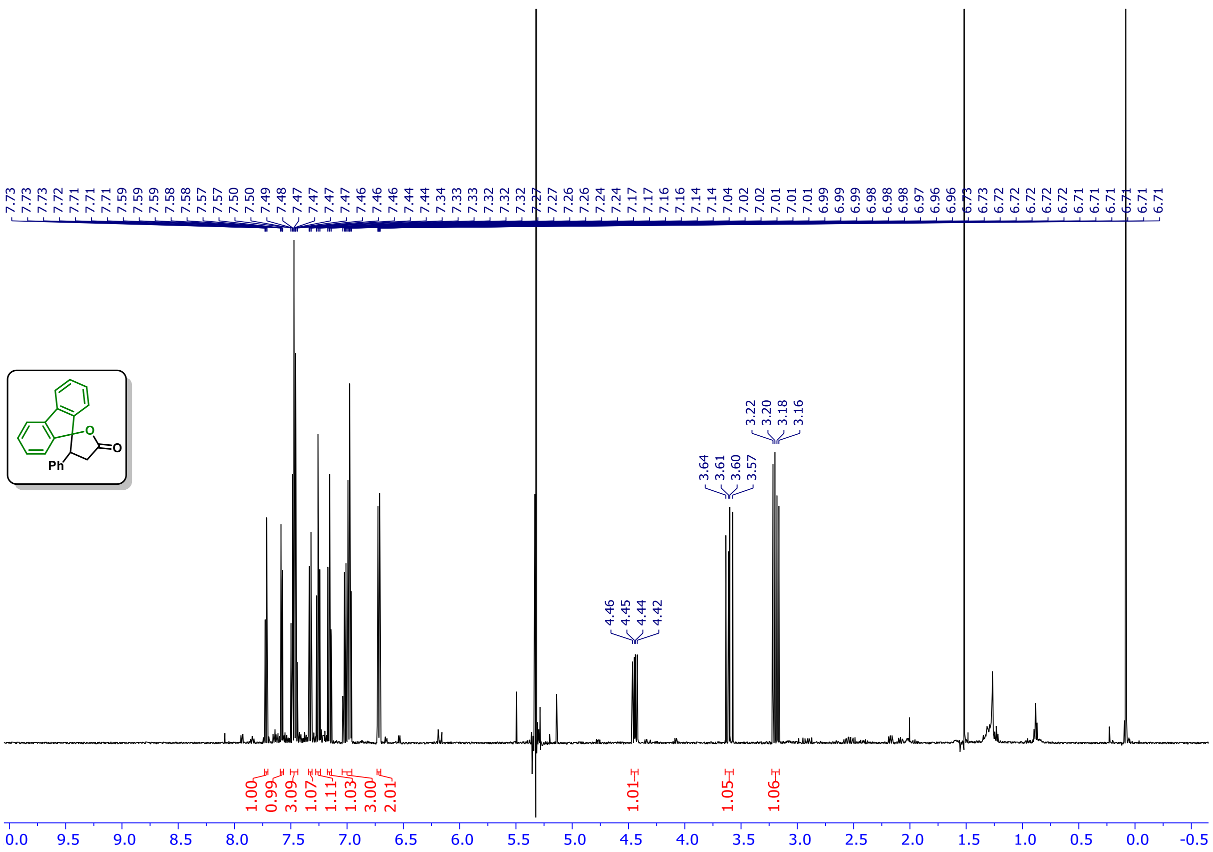

^{1}H-NMR spectrum (500 MHz, CD_2Cl_2) of **4k**.

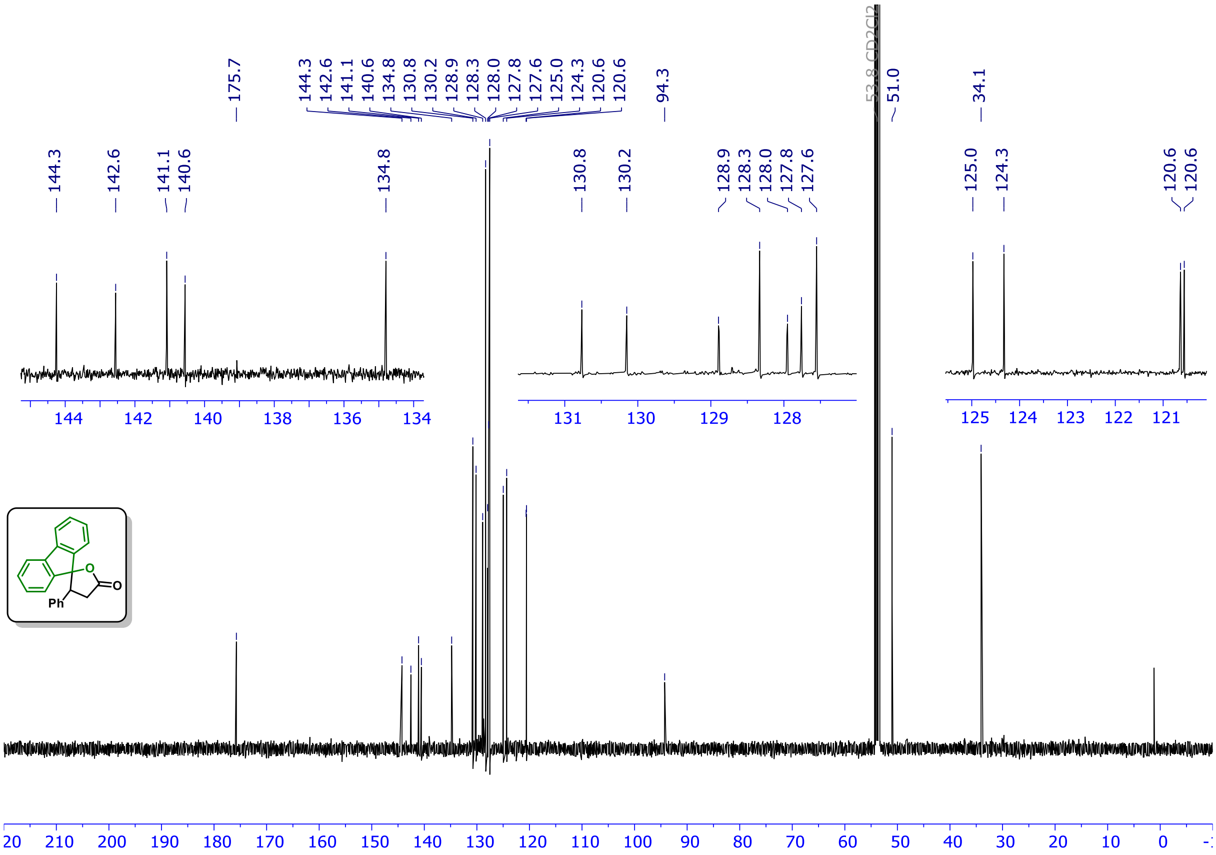

^{13}C-NMR spectrum (126 MHz, CD_2Cl_2) of **4k**.

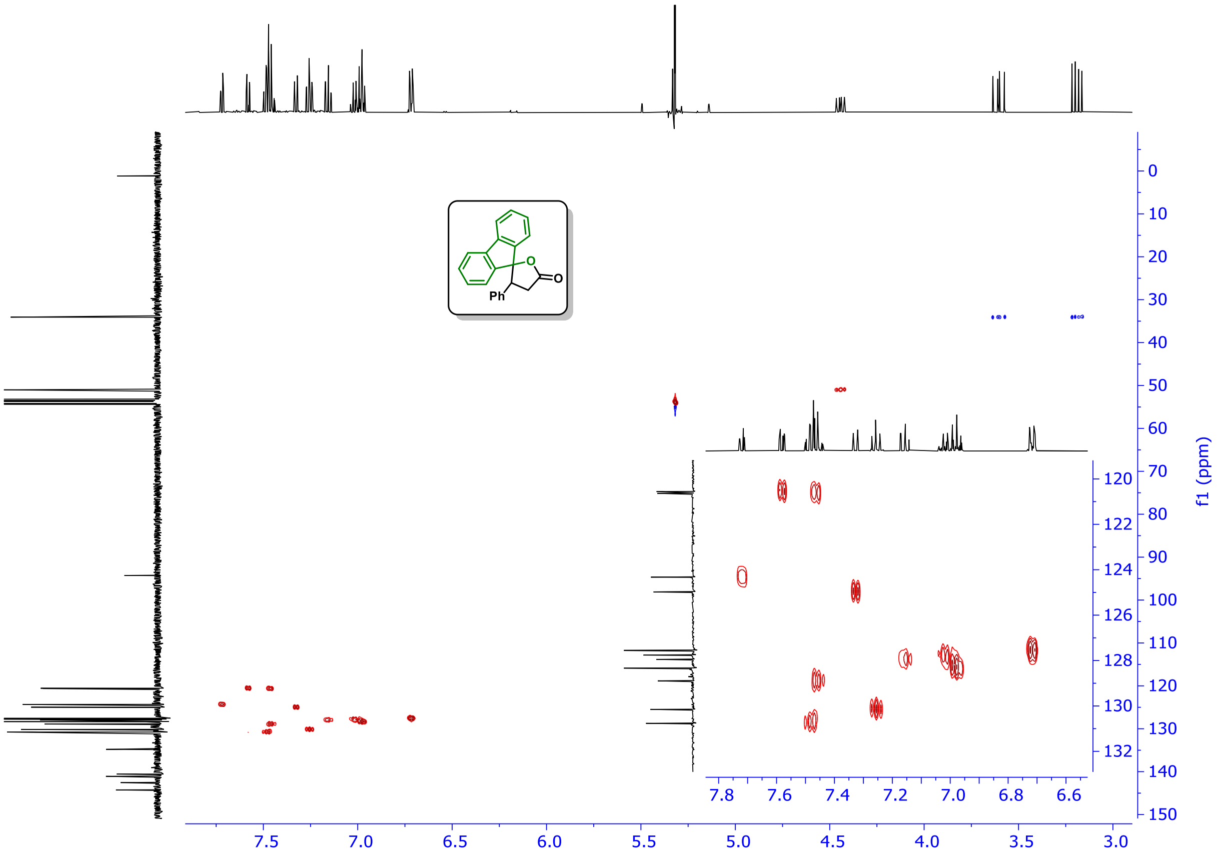

Ed^{1}H,^{13}C-HSQC-spectrum (CD_2Cl_2) of **4k**.

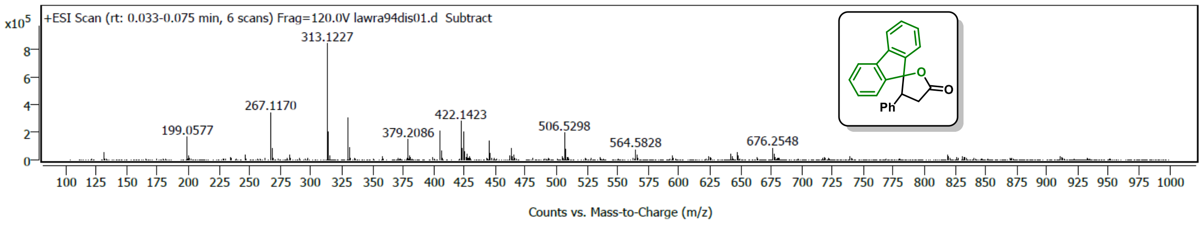

HR-MS Spectrum (ESI+) of **4k**.

Lactone 4l

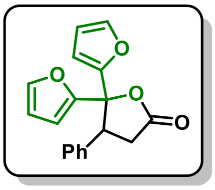

Prepared according to the **General Procedure** from SCP **1a** (54.9 mg, 0.20 mmol, 1.00 eq.) and di(furan-2-yl)methanone (259 mg, 1.60 mmol, 8.00 eq.). Purification by flash column chromatography (SiO_2, *n*-pentane/EtOAc 20:1) afforded lactone **4l** (30.9 mg, 0.10 mmol, 52%) as a colorless solid.

FTIR (ATR): $\tilde{\nu}$ [cm^{-1}] = 1785, 1499, 1206, 1159, 980, 914, 885, 818, 747, 699.

^{1}H-NMR (700 MHz, CD_3CN): δ_H (ppm) = 7.65 (dd, *J* = 1.9, 0.8 Hz, 1H), 7.35 (dd, *J* = 1.9, 0.8 Hz, 1H), 7.22–7.16 (m, 3H), 6.95–6.90 (m, 2H), 6.66 (dd, *J* = 3.4, 0.8 Hz, 1H), 6.53 (dd, *J* = 3.4, 1.9 Hz, 1H), 6.31 (dd, *J* = 3.4, 1.9 Hz, 1H), 6.09 (dd, *J* = 3.4, 0.8 Hz, 1H), 4.66 (dd, *J* = 11.7, 8.4 Hz, 1H), 3.24 (dd, *J* = 17.5, 11.7 Hz, 1H), 3.00 (dd, *J* = 17.5, 8.4 Hz, 1H).

^{13}C-NMR (176 MHz, CD_3CN): δ_C (ppm) = 175.4 (qC), 151.6 (qC), 150.4 (qC), 145.2 (CH), 144.2 (CH), 136.6 (qC), 129.2 (CH), 129.1 (CH), 128.7 (CH), 112.5 (CH), 112.0 (CH), 111.5 (CH), 111.4 (CH), 85.5 (qC), 49.9 (CH), 34.7 (CH_2).

HR-MS (GC-APCI, +, Q-TOF): calc. for $C_{18}H_{15}O_4$ $[M+H]^+$: 295.0965, found: 295.0965.

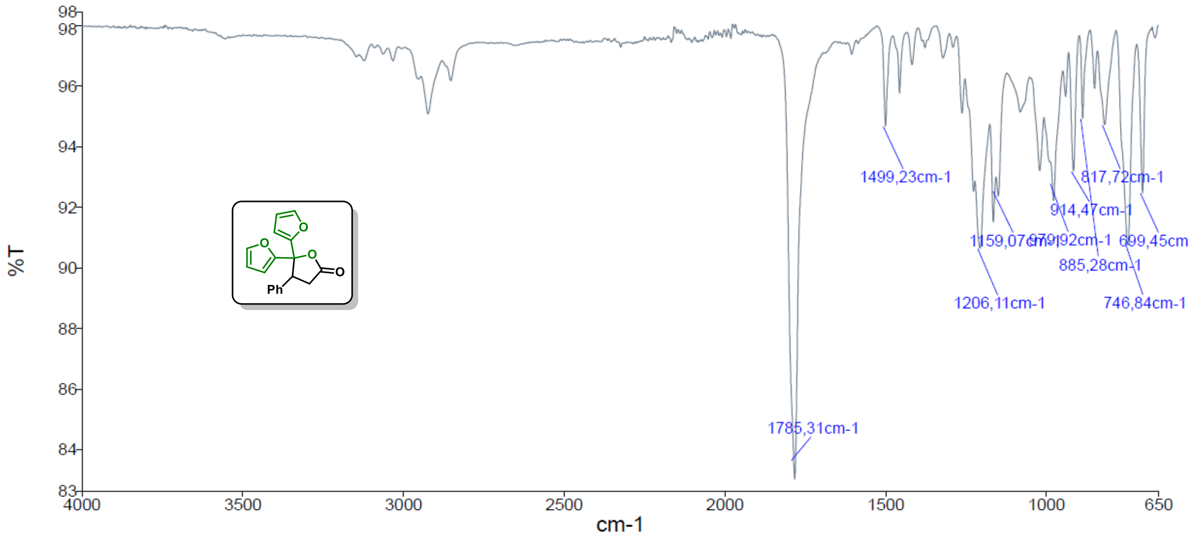

FT-IR Spectrum (ATR, thin film) of **4l**.

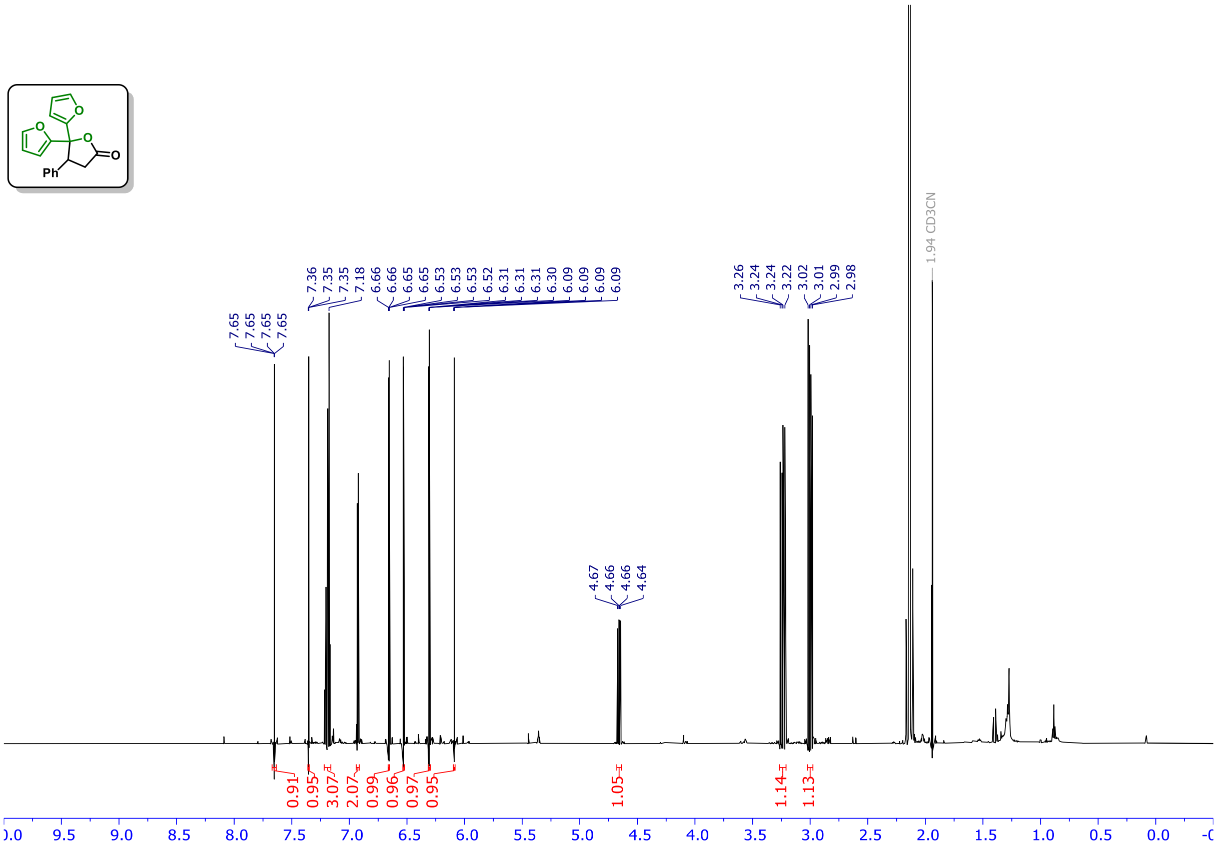

^{1}H-NMR spectrum (700 MHz, CD_3CN) of **4l**.

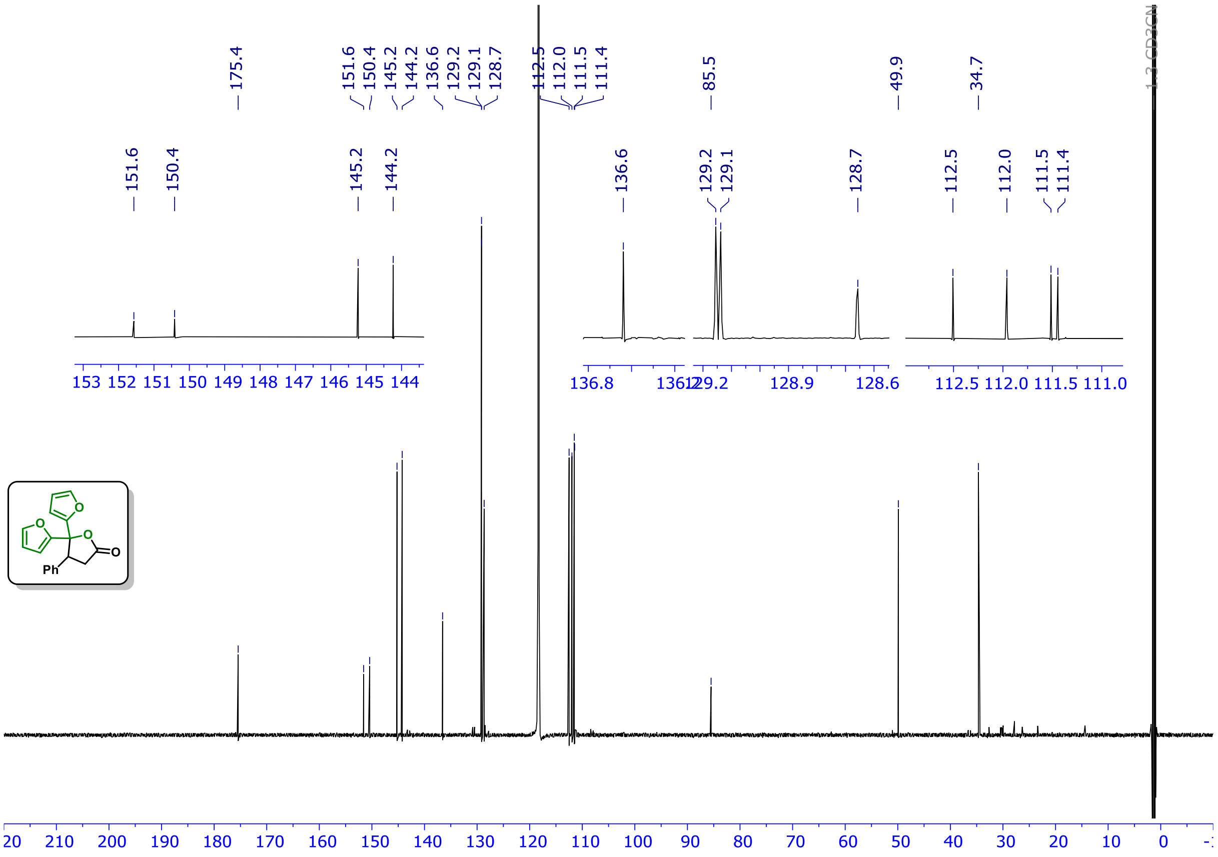

^{13}C-NMR spectrum (176 MHz, CD_3CN) of **4l**.

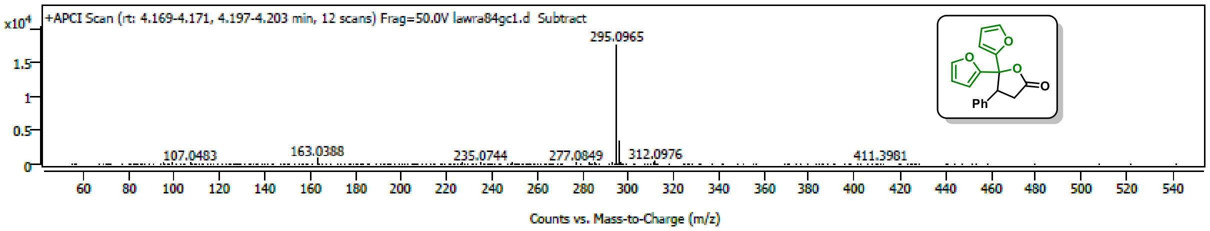

HR-MS Spectrum (APCI,+) of **4l**.

Lactone 4m

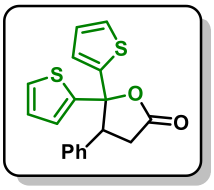

Prepared according to the **General Procedure** from SCP **1a** (54.9 mg, 0.20 mmol, 1.00 eq.) and di(thiophen-2-yl)methanone (311 mg, 1.60 mmol, 8.00 eq.). Purification by flash column chromatography (SiO_2, *n*-pentane/EtOAc 10:1) afforded lactone **4m** (37.3 mg, 0.11 mmol, 57%) as a colorless solid.

FTIR (ATR): $\tilde{\nu}$ [cm^{-1}] = 1772, 1235, 1211, 1141, 948, 896, 796, 726, 716, 696.

^{1}**H-NMR** (500 MHz, CD_2Cl_2): δ_H (ppm) = 7.43 (dd, J = 5.2, 1.2 Hz, 1H), 7.30–7.17 (m, 5H), 7.05 (dd, J = 5.1, 3.7 Hz, 1H), 7.00–6.95 (m, 2H), 6.81 (dd, J = 5.1, 3.7 Hz, 1H), 6.38 (dd, J = 3.7, 1.3 Hz, 1H), 4.48 (dd, J = 9.4, 8.0 Hz, 1H), 3.10–2.92 (m, 2H).

^{13}C-NMR (126 MHz, CD_2Cl_2): δ_C (ppm) = 174.4 (qC), 146.7 (qC), 142.3 (qC), 136.0 (qC), 129.0 (CH), 128.7 (CH), 128.4 (CH), 127.5 (CH), 127.4 (CH), 127.3 (CH), 127.2 (CH), 127.0 (CH), 126.0 (CH), 89.8 (qC), 54.4 (CH), 35.1 (CH_2).

HR-MS (GC-APCI, +, Q-TOF): calc. for $C_{18}H_{15}O_2S_2$ $[M+H]^+$: 327.0508, found: 327.0512.

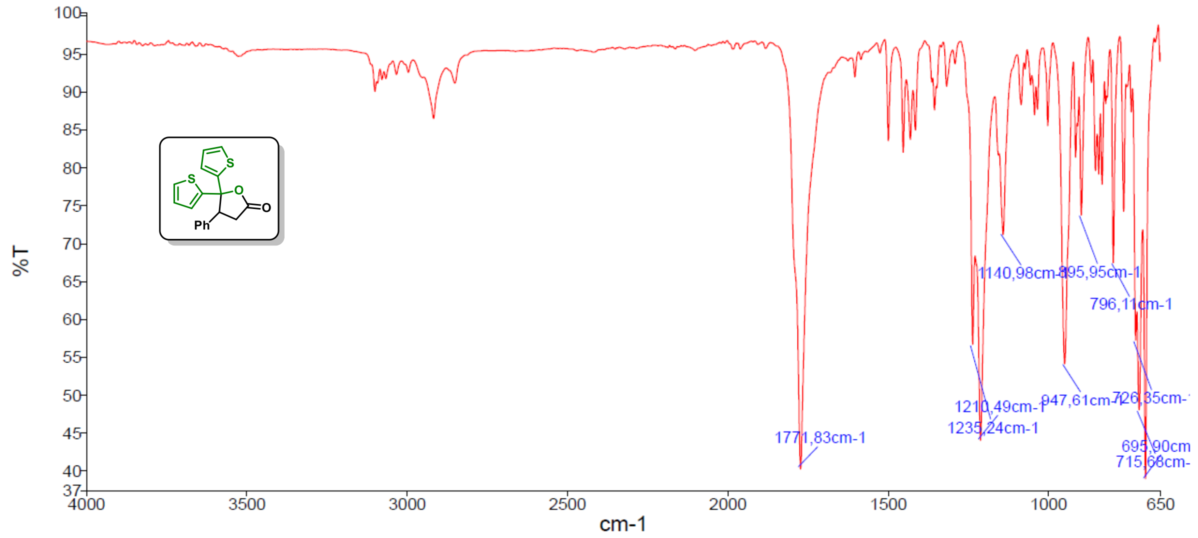

FT-IR Spectrum (ATR, thin film) of **4m**.

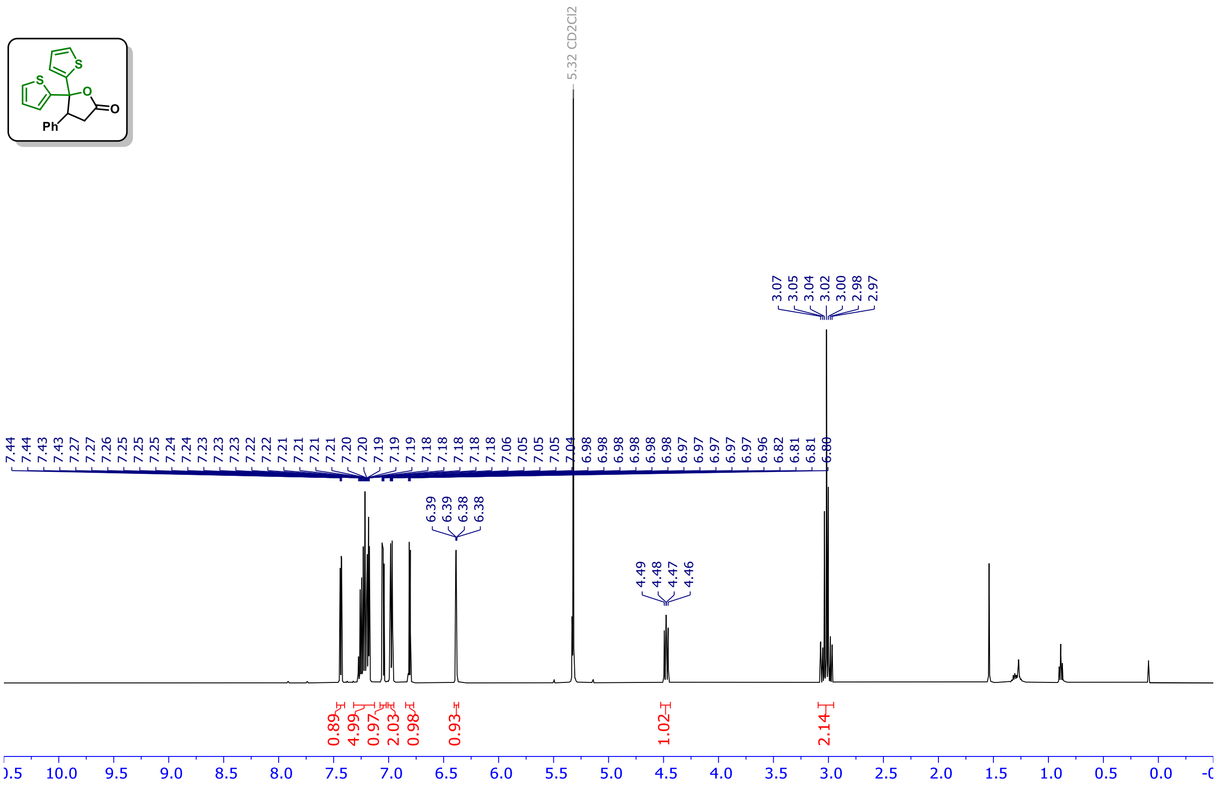

^{1}H-NMR spectrum (500 MHz, CD_2Cl_2) of **4m**.

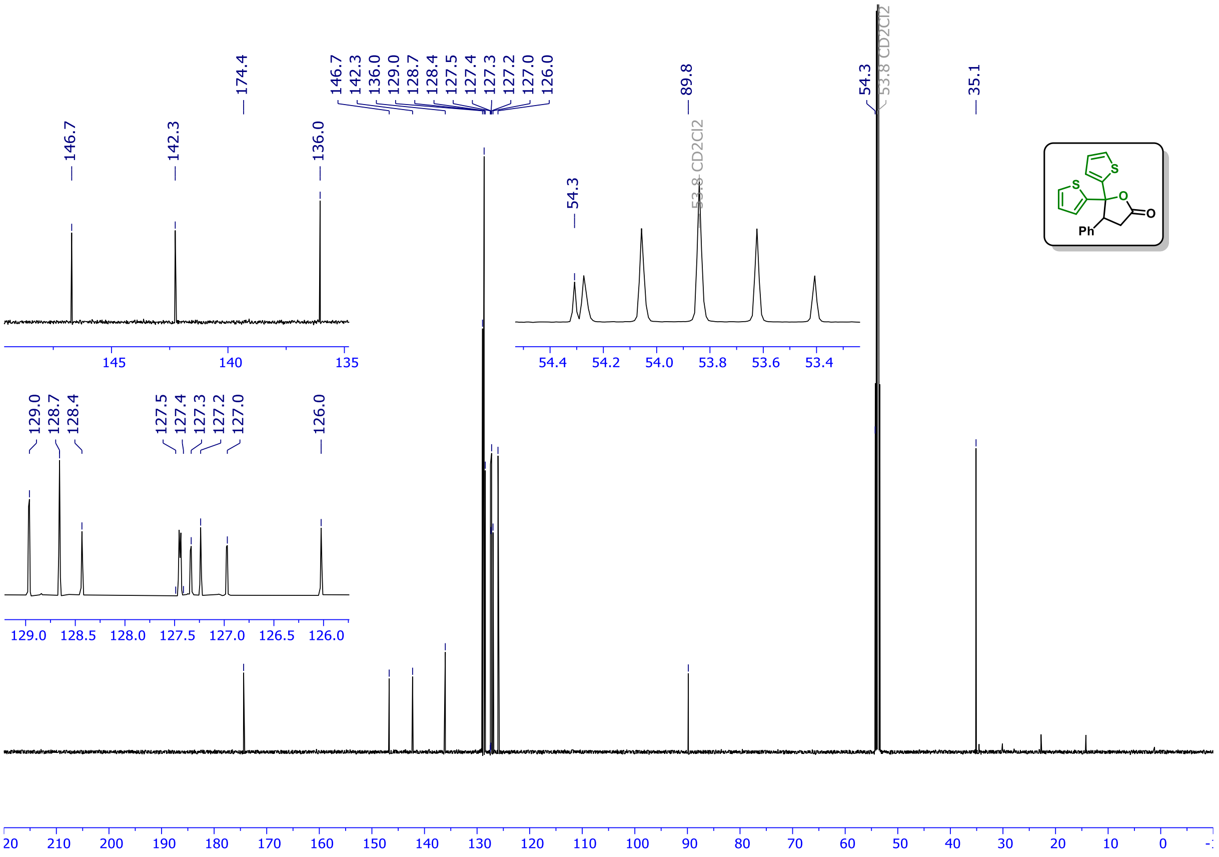

^{13}C-NMR spectrum (126 MHz, CD_2Cl_2) of **4m**.

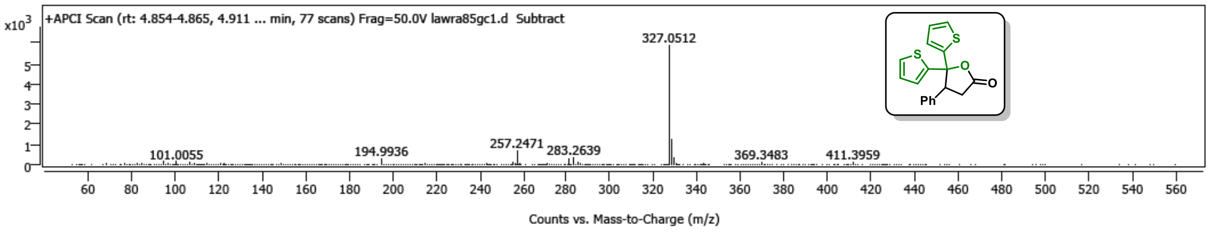

HR-MS Spectrum (APCI,+) of **4m**.

Lactone 4n

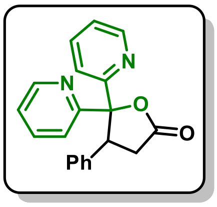

Prepared according to the **General Procedure** from SCP **1a** (54.9 mg, 0.20 mmol, 1.00 eq.) and di(pyridin-2-yl)methanone (295 mg, 1.60 mmol, 8.00 eq.). Purification by flash column chromatography (SiO_2, *n*-pentane/EtOAc 1:1) afforded lactone **4n** (25.5 mg, 0.08 mmol, 40%) as a colorless solid.

FTIR (ATR): $\tilde{\nu}$ [cm^{-1}] = 1779, 1587, 1431, 1212, 1175, 1139, 1005, 993, 748, 698.

^{1}H-NMR (500 MHz, CD_3CN): δ_H (ppm) = 8.69 (ddd, *J* = 4.9, 1.8, 1.0 Hz, 1H), 8.34 (ddd, *J* = 4.7, 1.8, 1.2 Hz, 1H), 7.76 (ddd, J = 7.5, 7.3, 1.8 Hz, 1H), 7.44 (td, *J* = 7.8, 1.8 Hz, 1H), 7.40–7.33 (m, 2H), 7.09–7.01 (m, 7H), 5.22 (dd, *J* = 8.7, 5.6 Hz, 1H), 3.11 (dd, *J* = 17.6, 8.7 Hz, 1H), 2.93 (dd, *J* = 17.6, 5.6 Hz, 1H).

^{13}C-NMR (126 MHz, CD_3CN): δ_C (ppm) = 177.0 (qC), 160.8 (qC), 159.6 (qC), 149.4 (CH), 149.2 (CH), 140.2 (qC), 138.2 (CH), 137.1 (CH), 129.8 (CH), 128.8 (CH), 127.7 (CH), 124.6 (CH), 124.2 (CH), 123.4 (CH), 122.3 (CH), 94.0 (qC), 48.9 (CH), 37.3 (CH_2).

HR-MS (ESI+, Orbitrap): calc. for $C_{20}H_{17}O_2N_2$ $[M+H]^+$: 317.1285, found: 317.1291.

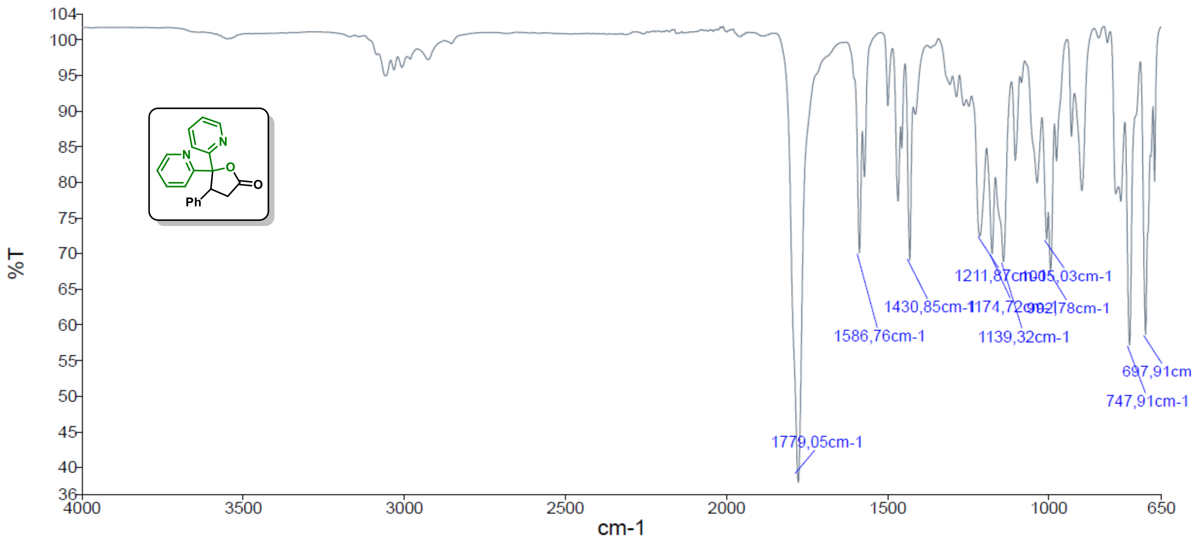

FT-IR Spectrum (ATR, thin film) of **4n**.

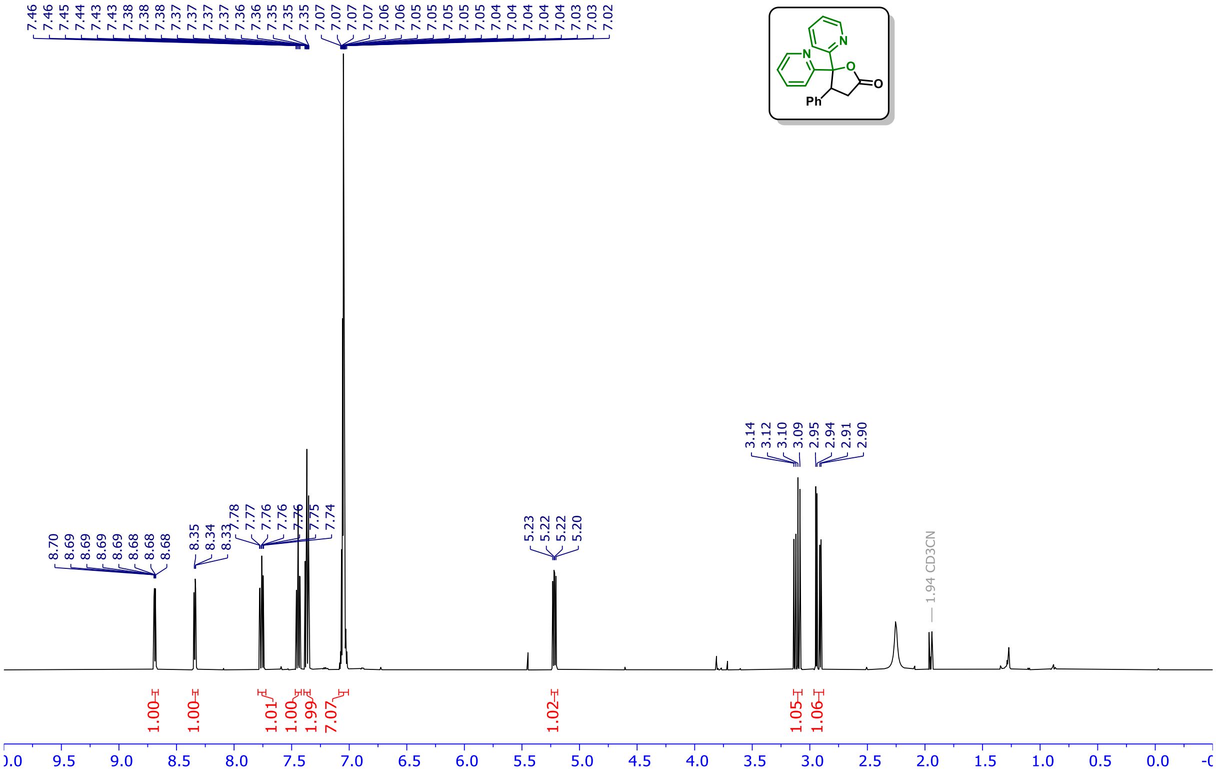

^{1}H-NMR spectrum (500 MHz, CD_3CN) of **4n**.

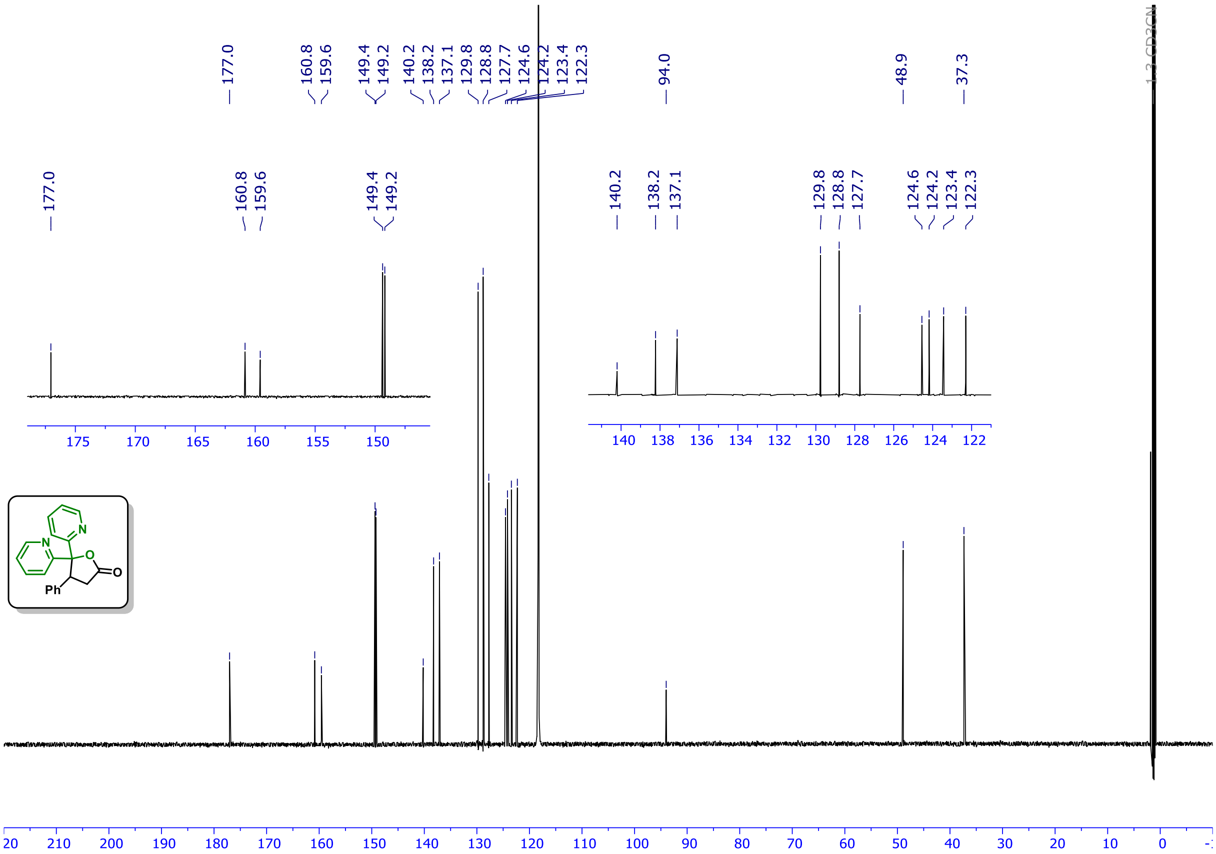

^{13}C-NMR spectrum (126 MHz, CD_3CN) of **4n**.

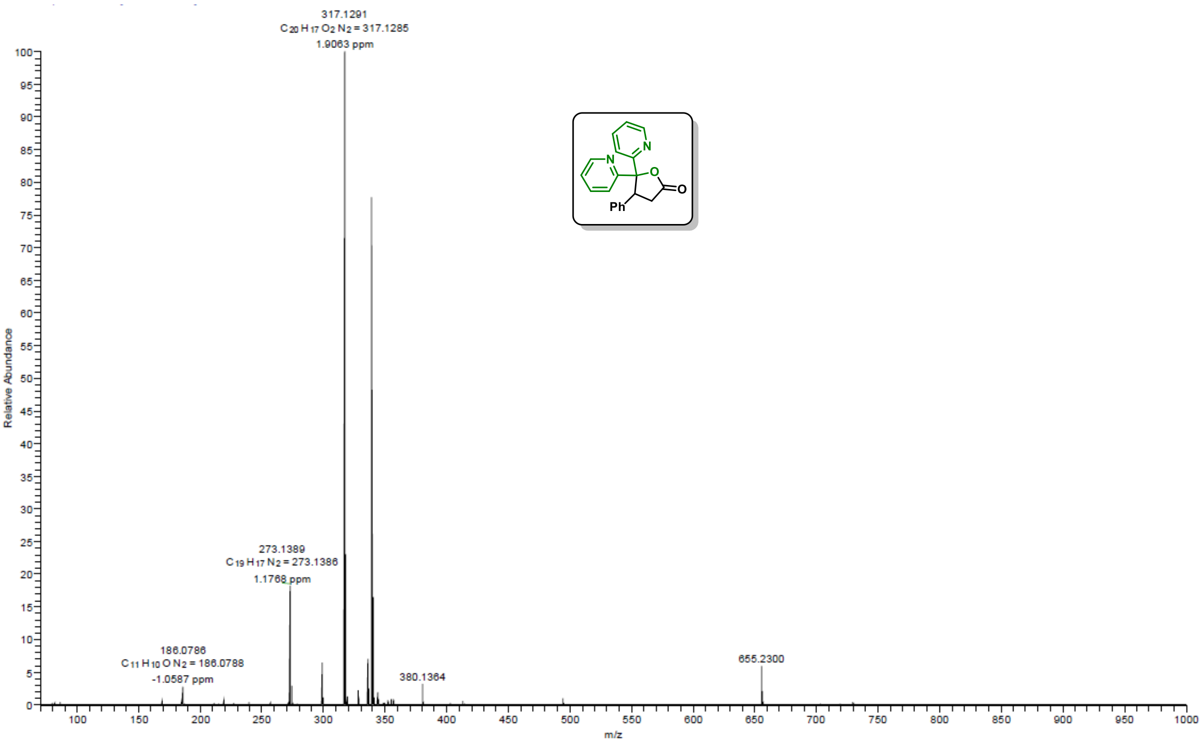

HR-MS Spectrum (ESI+) of **4n**.

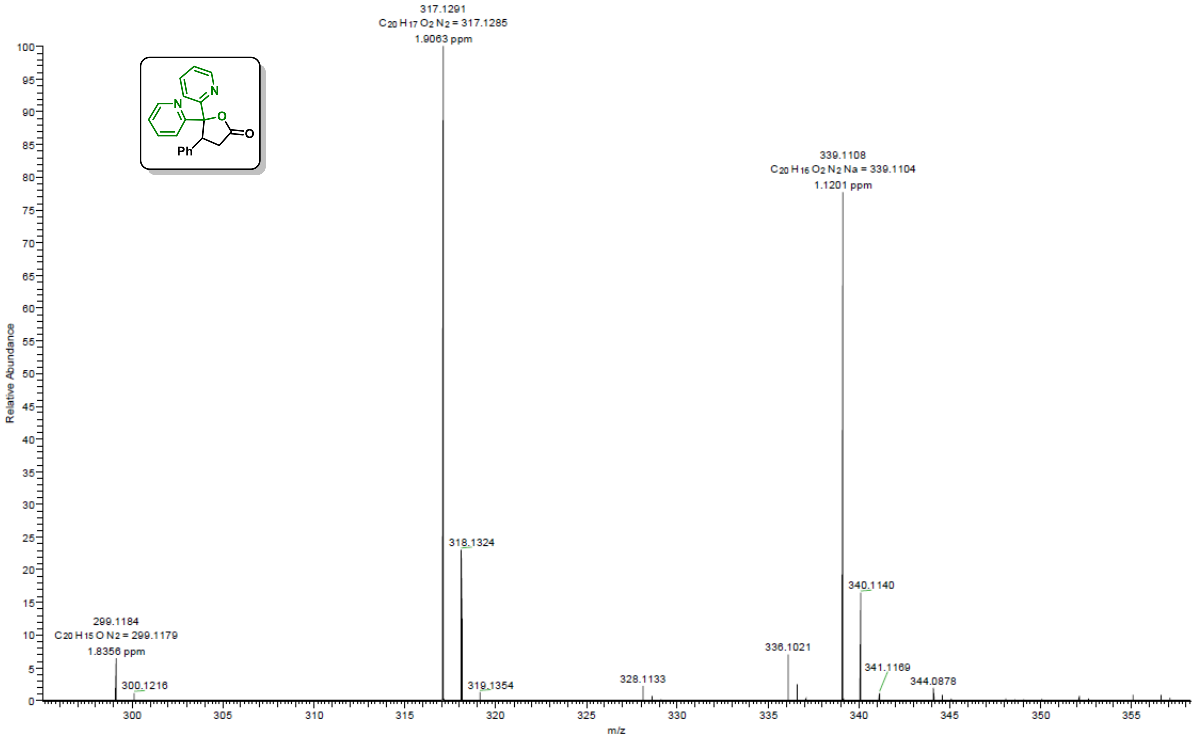

Excerpt of HR-MS Spectrum (ESI+) of **4n**.

DFT Studies

Methods

All calculations were performed with the ORCA program (versions 6.0.1).[1] Structures were pre-optimized by Grimme's tight-binding method XTB as implemented in the ORCA program suite.[2] In order to identify the global minimum, the pre-optimized structures were subjected to the GOAT module of ORCA. The global minimum geometry was further optimized by r^2scan-3c.[3] For the calculation of free energy contributions of translation, rotations and harmonic vibrations (G^{RRHO}, computed with r^2scan-3c) the temperature was set to 195 K and the quasi-RRHO approach for low-energy frequencies was chosen.[4] Single point calculations were performed with the hybrid functional PW6B95, def2-TZVP as basis set[5] and D4 as dispersion correction.[6] Solvent effects were taken into account by applying the CPCM solvation model (THF as solvent).[7]

The SCF convergence criteria were set to tight (Keyword: TightSCF) and DefGrid3 was used as integration grid in ORCA.

Results

Pathway without Activation of the Cyclopropanone

Initially, we examined the uncatalyzed reaction between cyclopropanone **A** and benzaldehyde.

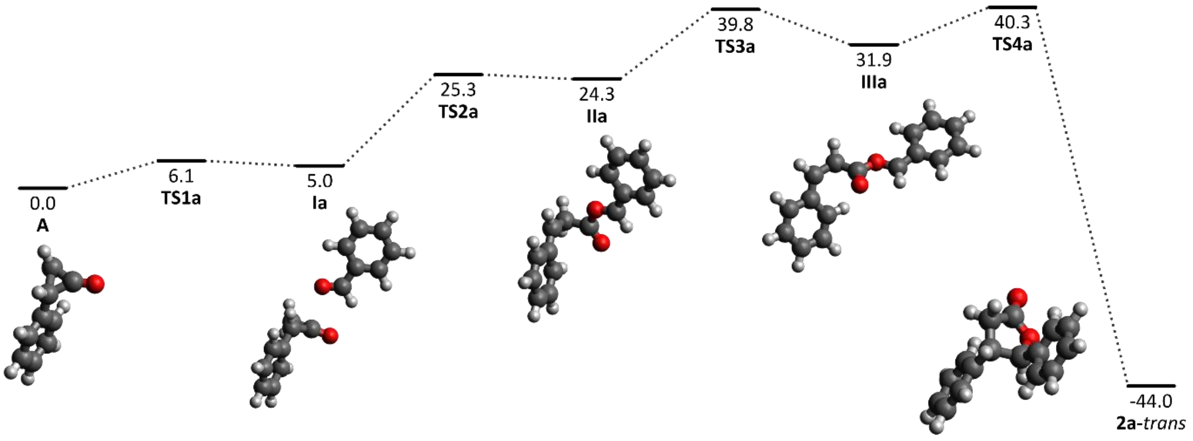

Figure S1. Gibb's Free Energy Reaction Profile of the uncatalyzed reaction of cyclopropanone **A** and benzaldehyde (PW6B95, def2-TZVP-D4//r^{2}scan-3c, CPCM(THF)).

Table S1. DFT-calculated energies and thermostatistical contributions of species in the uncatalyzed reaction of cyclopropanone **A** and benzaldehyde.

Species	E(PW6B95-D4) [E_h]	G^{RRHO}_{298}(r^{2}scan-3c) [E_h]	ΔG(195) [kcal/mol]	
**A**	-423.6617855	0.1217577	0.0	
benzaldehyde	-346.1610082	0.0909196	0.0	ΔΔG = 6.1 kcal/mol
**TS1a**	-769.8272906	0.22685339	6.1	
**Ia**	-769.8278592	0.22562338	5.0	ΔΔG = 20.3 kcal/mol
**TS2a**	-769.7993073	0.22945861	25.3	
**IIa**	-769.8010603	0.22970733	24.3	ΔΔG =15.5 kcal/mol
**TS3a**	-769.7749548	0.22824910	39.8	
**IIIa**	-769.7867322	0.22750735	31.9	ΔΔG = 8.3 kcal/mol
**TS4a**	-769.7741749	0.22823466	40.3	
**2a-*trans***	-769.9150213	0.23475610	-44.0	

The formation of adduct **Ia**, characterized by a C(cyclopropanone)–O(benzaldehyde) distance of 2.7 Å ($\Delta G^‡ = 6.1$ kcal mol^{-1}), represents the first step of the process. Subsequent nucleophilic attack of benzaldehyde on the carbonyl carbon of cyclopropanone gives intermediate **IIa** ($\Delta G^‡ = 20.3$ kcal mol^{-1}). Rearrangement of this intermediate affords the linear zwitterionic species IIIa ($\Delta G^‡ = 15.5$ kcal mol^{-1}), which finally undergoes ring closure to form product **2a-*trans***. Although these calculated energy values are consistent with the proposed mechanism, the relatively high barrier for the nucleophilic attack step suggests that the

reaction would proceed very slowly under cryogenic conditions. This observation highlights the need for initial activation of the carbonyl group.

Activation of the Cyclopropanone by a TBS Moiety

Therefore, alternative mechanisms were explored to better account for the observed reaction outcome. Given the use of TBSOTf in catalytic amounts, attention was directed to the potential role of a TBS moiety in facilitating the transformation.

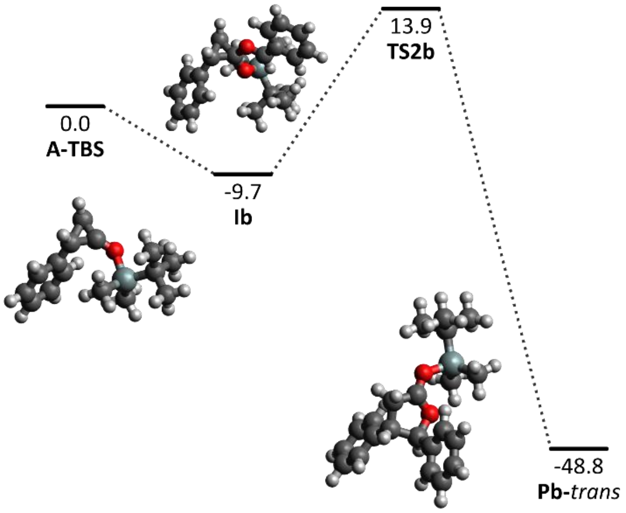

Figure S2. Gibb's Free Energy Reaction Profile of the TBS-catalyzed reaction of cyclopropanone **A** and benzaldehyde (PW6B95, def2-TZVP-D4//r2scan-3c, CPCM(THF)).

Table S2. DFT-calculated energies and thermostatistical contributions of species in the TBS-catalyzed reaction of cyclopropanone **A** and benzaldehyde.

Species	E(PW6B95-D4) [E_h]	G^{RRHO}_{298}(r^2scan-3c) [E_h]	ΔG(195) [kcal/mol]	
**A-TBS**	-951.3925099	0.3130769	0.0	
benzaldehyde	-346.1610082	0.09091963	0.0	
**TS1b**	Barrierless Approach			
**Ib**	-1297.5888260	0.4238599	-9.7	ΔΔG = 23.6 kcal/mol
**TS2b**	-1297.5498074	0.4224392	13.9	
**Pb-*trans***	-1297.6540787	0.4268269	-48.8	

Here, the TBS-group coordinates to the carbonyl oxygen. In a barrierless approach, nucleophilic attack by the carbonyl oxygen of benzaldehyde generates intermediate **Ib**. To access the experimentally observed product, a rearrangement with a calculated activation barrier of 23.6 kcal/mol is required. This substantial barrier indicates that the transformation

would proceed extremely slow at -78 °C. Thus, it is unlikely that this pathway is responsible for product formation under the given conditions.

Activation of the Cyclopropanone by Lithium Ions

Next, we considered the possibility that lithium ions present in the reaction mixture could coordinate to the carbonyl group of cyclopropanone. Such coordination is expected to activate the cyclopropanone, thereby facilitating nucleophilic attack by the aldehyde. To investigate this pathway, it was important to account for lithium ion coordination not only to the substrate, but also to solvent molecules (THF), which are abundant in the reaction environment. Modeling solvation exclusively through an implicit solvent approach would be insufficient; thus, explicit inclusion of THF molecules was employed. Pathways involving lithium coordinated by two or three THF molecules were subsequently calculated.

Inclusion of two THF molecules

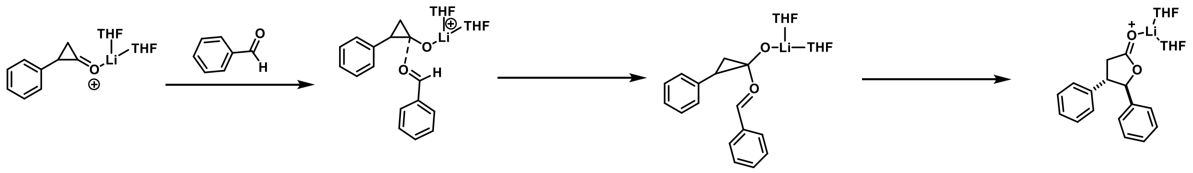

Ac Ic IIc Pc-trans

Scheme S1. Li-catalyzed Reaction of cyclopropanone A with benzaldehyde considering two explicit THF molecules (PW6B95, def2-TZVP-D4//r^{2}scan-3c, CPCM(THF)).

Table S3. DFT-calculated energies and thermostatistical contributions of species in the Li-catalyzed reaction of cyclopropanone **A** and benzaldehyde with two explicit THF molecules.

Species	E(PW6B95-D4) [E_h]	G^{RRHO}_{298}(r^{2}scan-3c) [E_h]	ΔG(195) [kcal/mol]	
**Ac**	-896.8422631	0.3505383	0.0	
benzaldehyde	-346.1610018	0.0909196	0.0	
**Ic**	-1243.0154806	0.4576824	2.5	ΔΔG = 3.8 kcal/mol
**TS2c**	-1243.0122162	0.4604617	6.3	
**IIc**	-1243.0123328	0.4616342	7.0	ΔΔG = 17.3 kcal/mol
**TS3c**	-1242.9833200	0.4601816	24.3	
**Pc-*trans***	-1243.0995290	0.4640553	-46.2	

Initially, the reactants form an adduct **Ic**, after which nucleophilic attack of benzaldehyde at the cyclopropanone carbonyl yields intermediate **IIc** (ΔG‡ = 3.8 kcal/mol). Subsequent re-arrangement affords the product **Pc-*trans***. Given an activation barrier of 17.3 kcal/mol, this transformation is still expected to proceed very slowly at −78°C. So, the case where three THF molecules are coordinating the lithium atom was investigated.

*Note: The Gibbs free energy of **IIc** is calculated to be slightly higher than that of **TS2c**, which is counterintuitive at first glance, but the small difference lies within the expected uncertainty of the applied electronic-structure method and thermochemical corrections. Nevertheless, both intrinsic reaction coordinate (IRC) and nudged elastic band (NEB) calculations unequivocally identify **TS2c** as the transition state connecting **Ic** and **IIc**.*[8,9,10]

Inclusion of three THF molecules

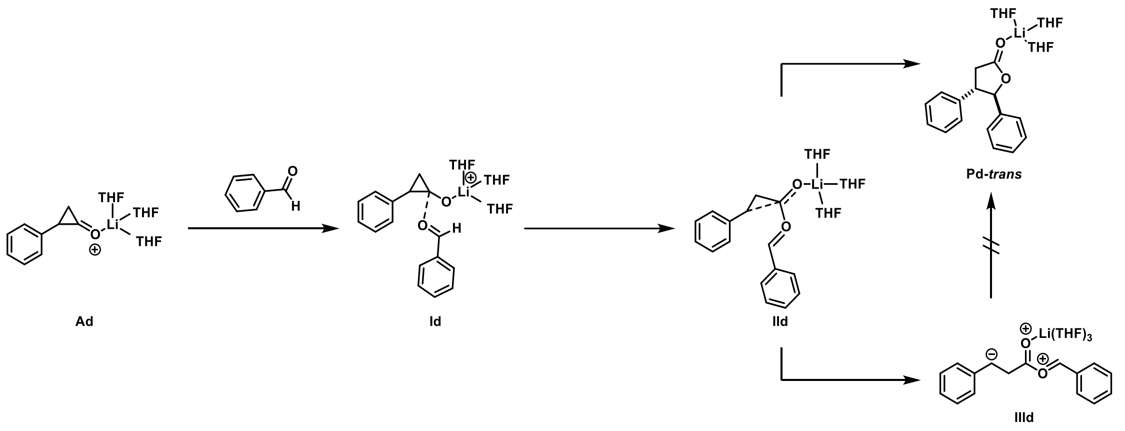

Scheme S2. Li-catalyzed Reaction of cyclopropanone **A** with benzaldehyde considering three explicit THF molecules (PW6B95, def2-TZVP-D4//r^2scan-3c, CPCM(THF)).

Table 4. DFT-calculated energies and thermostatistical contributions of species in the Li-catalyzed reaction of cyclopropanone A and benzaldehyde with three explicit THF molecules.

Species	E(PW6B95-D4) [E_h]	G^{RRHO}_{298}(r^2scan-3c) [E_h]	ΔG(195) [kcal/mol]	
**Ad**	-1129.7035559	0.4653440	0	
benzaldehyde	-346.1610018	0.0909196	0	ΔΔG =3.6 kcal/mol
**TS1d**	-1475.8751657	0.5713697	3.6	
**Id**	-1475.8751657	0.5713697	2.8	ΔΔG = 16.6 kcal/mol
**TS2d**	-1475.8510910	0.5736996	19.4	
**IId**	-1475.8507695	0.5743657	20.0	ΔΔG = 20.0 kcal/mol
**TS3d**	-1475.8180750	0.5729847	40.0	
**Pd-*trans***	-1475.9591592	0.5796320	-44.7	
**IId**	-1475.8507695	0.5743657	20.0	ΔΔG = 12.8 kcal/mol
**TS4d**	-1475.8286335	0.5726520	32.8	
**IIId**	-1475.8322920	0.5728170	30.6	

Initially, the reactants form an adduct **Id**, followed by nucleophilic attack of benzaldehyde at the cyclopropanone carbonyl group to yield intermediate **IId**. Notably, the activation barrier for this step is 16.6 kcal/mol, which is significantly higher than that for the formation of **IIc**. A

final rearrangement of intermediate **IId**, associated with an activation barrier of 20.0 kcal/mol, affords the product **Pd-*trans***.

Instead of direct formation of the five-membered ring, intermediate IId can also rearrange to the ring-opened intermediate **IIId** via an activation barrier of 12.8 kcal/mol. Nevertheless, no direct path from there to product P**d-*trans*** was found, leading to the conclusion that this pathway is not available here.

*Note: The Gibbs free energy of **IId** is calculated to be slightly higher than that of **TS2d**, which is counterintuitive at first glance, but the small difference lies within the expected uncertainty of the applied electronic-structure method and thermochemical corrections. Nevertheless, both intrinsic reaction coordinate (IRC) and nudged elastic band (NEB) calculations unequivocally identify **TS2c** as the transition state connecting **Id** and **IId**.*[8,9,10]

Since the reaction was performed in a highly diluted solution, it must be assumed that the coordination number of THF molecules to lithium can vary throughout the course of the reaction. Consequently, energy differences between intermediates featuring different numbers of coordinating solvent molecules were systematically compared.

Table S5. DFT-calculated energies (in kcal/mol) of different numbers of explicit solvent molecules (PW6B95, def2-TZVP-D4//r^{2}scan-3c, CPCM(THF)).

Species	no coordinated Lithium	Coordination of $Li(THF)_2$	Coordination of $Li(THF)_3$
**CP**	0.0	6.5	-0.8
**Int1**	0.0	4.0	-3.0
**Int2**	0.0	-10.9	-5.2
**Product**	0.0	66.3	-1.6

Proposed Mechanism

This comparison clearly highlights that equilibria between different coordination spheres of lithium are likely under reaction conditions. Notably, only for the product does the energy difference between lithium coordinated by two versus three THF molecules become substantial. Consequently, it can be inferred that the actual reaction pathway involves dynamic dissociation and association of THF molecules to lithium. On this basis, the following reaction pathway is proposed.

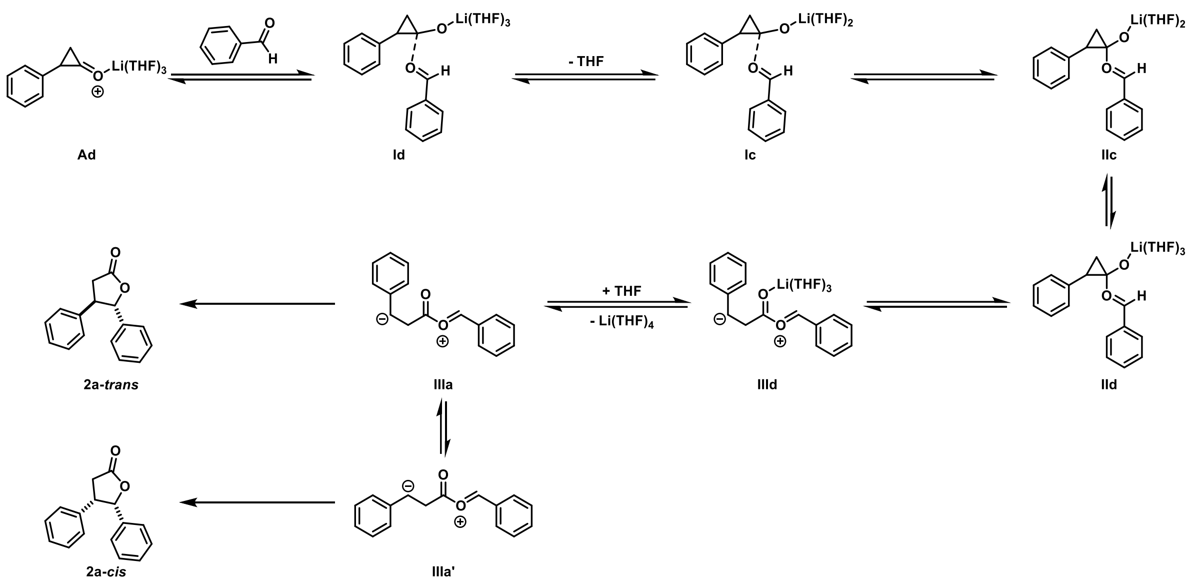

Scheme S3. Final proposed mechanism between cyclopropanone **Ad** and benzaldehyde.

Table 6. DFT-calculated energies and thermostatistical contributions of species in the Li-catalyzed reaction of cyclopropanone A and benzaldehyde with a varying number of explicit THF molecules.

Species	E(PW6B95-D4) [E_h]	$G^{RRHO}{}_{298}$(r^2scan-3c) [E_h]	ΔG(195) [kcal/mol]	
**Ad**	-1129.7035559	0.4653440	0	
benzaldehyde	-346.1610018	0.0909196	0	ΔΔG =3.6 kcal/mol
**TS1d**	-1475.8751657	0.5713697	3.6	
**Id**	-1475.8751657	0.5713697	2.8	
**Ic**	-1475.8491968	0.5565636	9.8	ΔΔG =3.8 kcal/mol
**TS2c**	-1475.8459325	0.5593429	13.6	
**IIc**	-1475.8460490	0.5605154	14.3	
**IId**	-1475.8507695	0.5743657	20.0	ΔΔG =12.8 kcal/mol
**TS4d**	-1475.8286335	0.5726520	32.8	
**IIId**	-1.475.8322920	0.5728170	30.6	
**IIIa**	-1475.8266680	0.5706031	34.2	ΔΔG =6.9 kcal/mol
**TS4a**	-1475.8141108	0.5713304	41.1	
**2a-*trans***	-1475.9549573	0.5778518	-43.2	
**IIIa**	-1475.8266680	0.5706031	34.2	ΔΔG =6.5 kcal/mol
**TS6e**	-1475.8148865	0.5713780	40.7	
**IIIa'**	-1475.8266681	0.5706031	32.8	ΔΔG =5.2 kcal/mol
**TS4a'**	-1475.8200263	0.5723118	38.0	
**2a-*cis***	-1475.9561386	0.5786819	-43.4	

The reaction initiates with cyclopropanone **Ad**, which forms an adduct **Id** upon interaction with benzaldehyde. Subsequently, dissociation of one THF molecule from the lithium ion occurs, enabling nucleophilic attack of benzaldehyde at the activated cyclopropanone.

Following this attack, reassociation of a THF molecule to the lithium ion takes place, after which the ring-opening step leads to intermediate **IIId**. Notably, this ring-opening step is identified as the rate-determining step, exhibiting an activation barrier of 12.8 kcal/mol. Subsequently, the lithium ion decoordinates from the carbonyl group, and a final rearrangement (ΔΔG = 6.9 kcal/mol) yields the product **2a-*trans***. Alternatively, rotation of the benzylic anion moiety, with a barrier of 6.5 kcal/mol, generates intermediate **IIIa'**, from which rearrangement leads to the formation of product **2a-*cis***.

DFT-optimized (r^{2}scan-3c) intermediates and transition state structures.

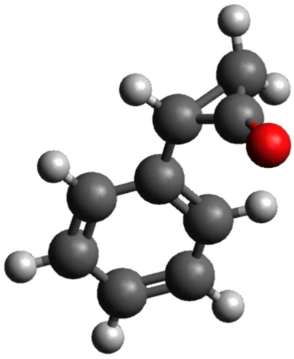

A

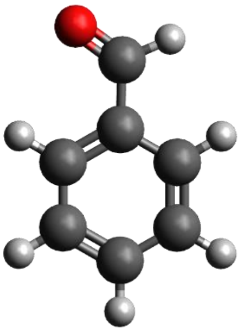

benzaldehyde

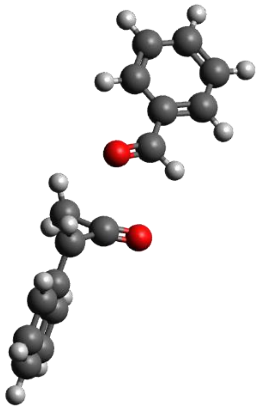

TS1a

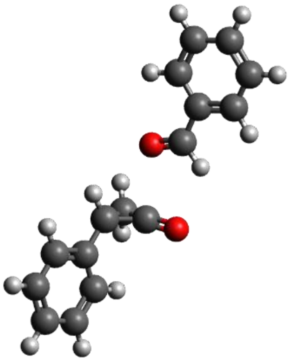

Ia

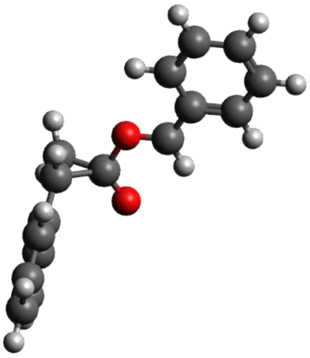

TS2a

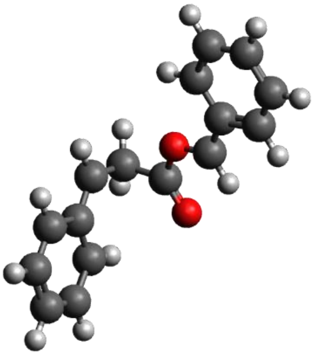

IIa

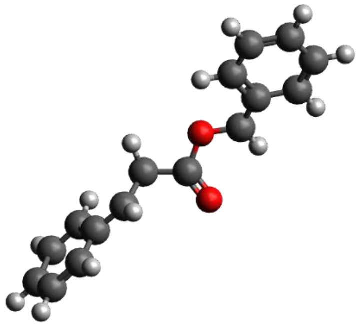

TS3a

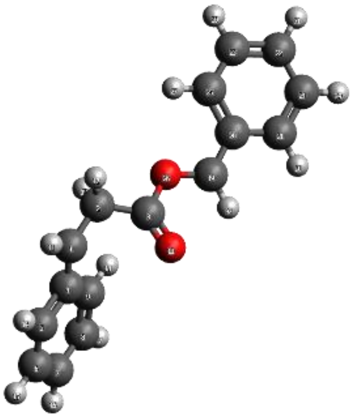

IIIa

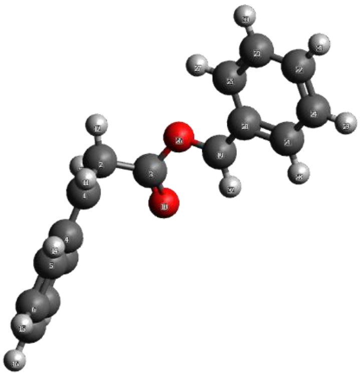

TS4a

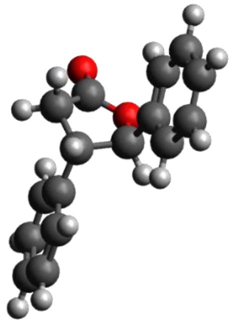

2a-trans

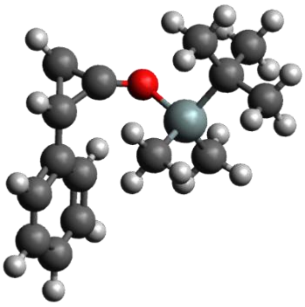

A-TBS

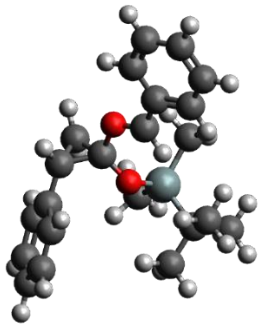

Ib

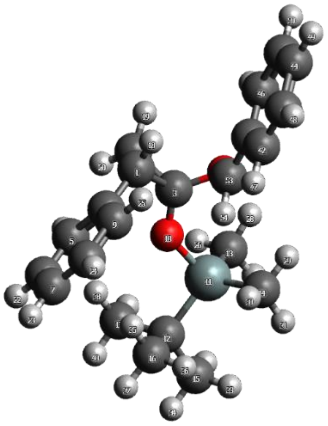

TS2b

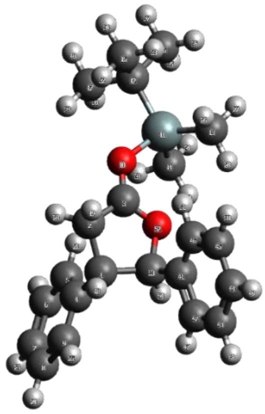

Pb-*trans*

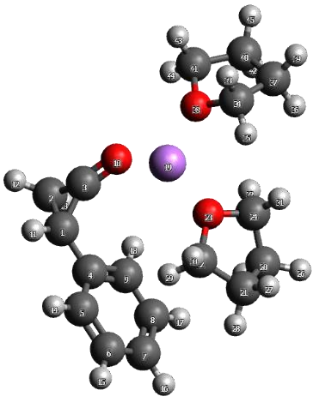

A-c

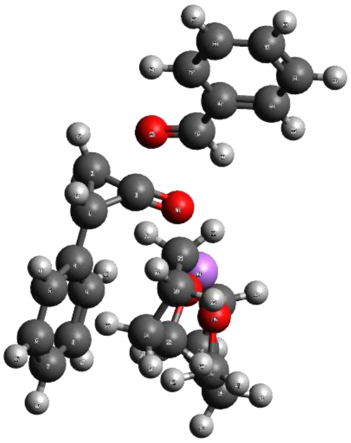

Ic

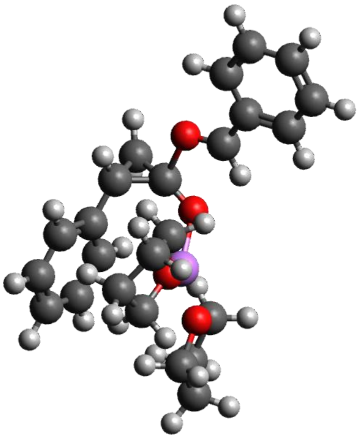

TS2c

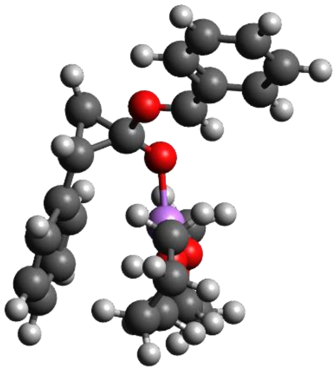

IIc

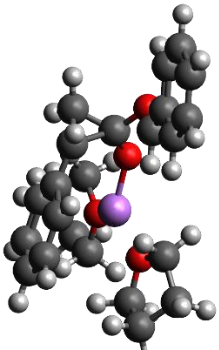

TS3c

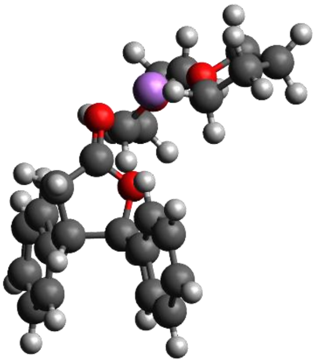

Pc-*trans*

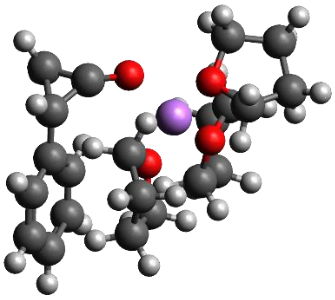

A-d

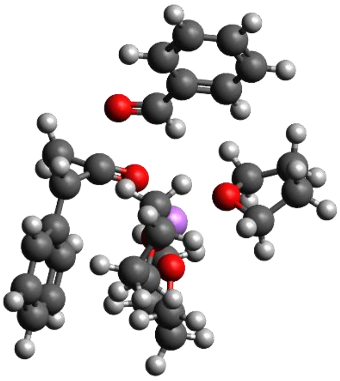

TS1d

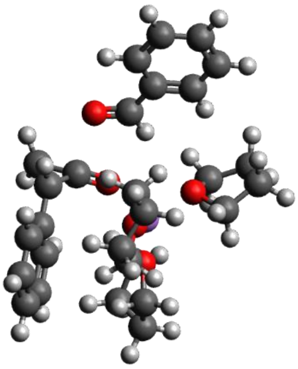

Id

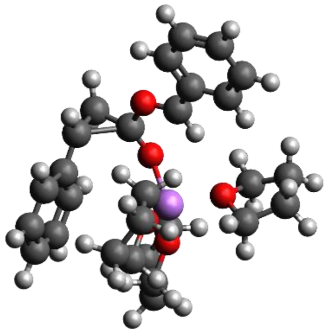

TS2d

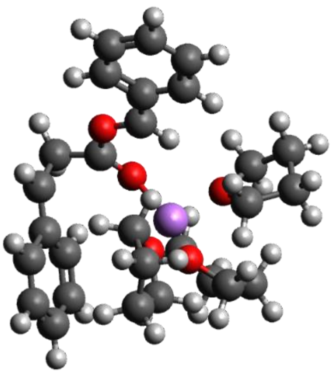

IId

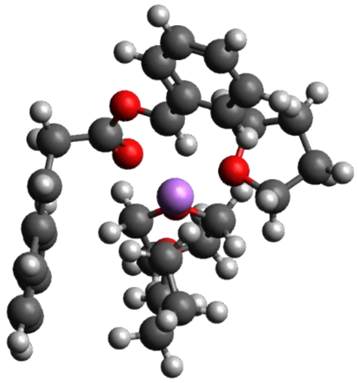

TS3d

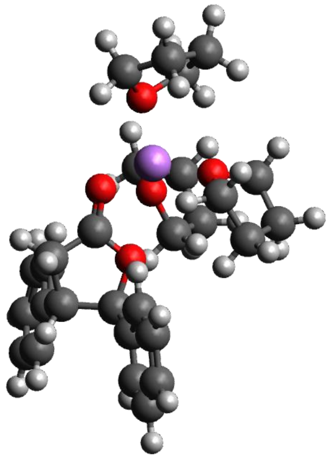

Pd-*trans*

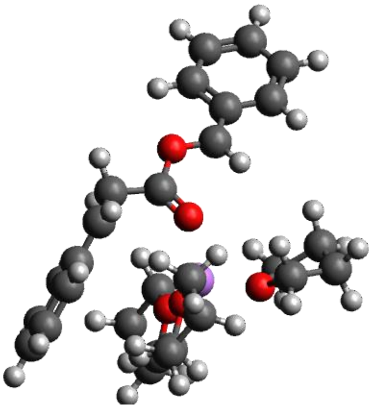

TS4d

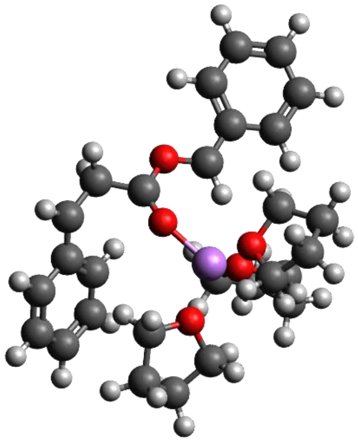

IIId

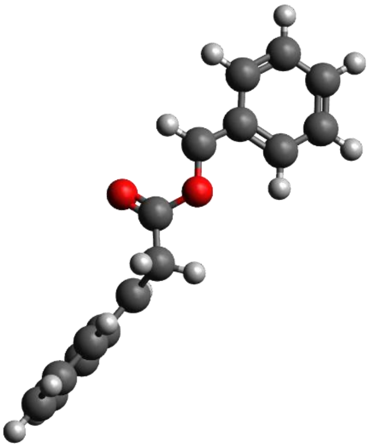

TS6e

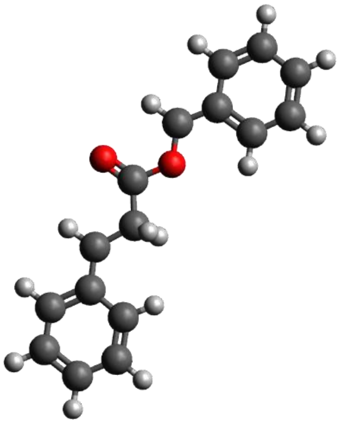

IIIa’

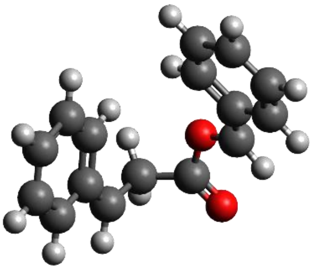

TS4a’

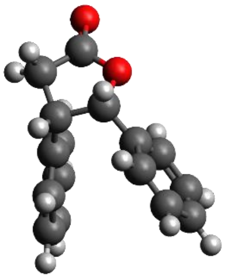

2a-cis

DFT optimized (r^2scan-3c) cartesian coordinate

A

E(r^2scan-3c) = -422.851227 (conv)

Lowest Freq. = 29.19 cm^{-1}

18

C	-2.4863	1.3376	2.1525
C	-3.1445	0.7086	3.4428
C	-3.1840	0.0376	2.1574
C	-1.0168	1.4155	1.9542
C	-0.4768	2.4659	1.2037
C	0.8963	2.5538	0.9925
C	1.7514	1.5923	1.5252
C	1.2212	0.5409	2.2708
C	-0.1504	0.4501	2.4828
O	-3.4226	-0.9687	1.5410
H	-3.0544	2.1801	1.7592
H	-4.0260	1.2311	3.8081
H	-2.4973	0.2890	4.2132
H	-1.1388	3.2217	0.7881
H	1.2985	3.3795	0.4118
H	2.8229	1.6609	1.3612
H	1.8790	-0.2164	2.6883
H	-0.5427	-0.3822	3.0625

benzaldehyde

E(r^2scan-3c) = -345.504849 (conv)

Lowest Freq. = 113.23 cm^{-1}

14

C	-1.2260	0.1592	0.0001
C	-1.2056	-1.2270	-0.0000
C	0.0148	-1.9084	-0.0001
C	1.2172	-1.2040	-0.0000
C	1.2007	0.1861	0.0001
C	-0.0192	0.8721	0.0002
H	-2.1644	0.7065	0.0002
H	-2.1373	-1.7855	-0.0001
H	0.0254	-2.9949	-0.0002
H	2.1622	-1.7390	-0.0001
H	2.1317	0.7491	0.0002
C	-0.0059	2.3428	0.0005
O	-1.0021	3.0473	-0.0004
H	1.0084	2.7978	-0.0005

TS1a

E(r^2scan-3c) = -768.360899 (conv)

Lowest Freq. = -16.18 cm^{-1}

32

C	-2.3383	0.3784	-0.8766
C	-2.1790	0.6354	0.6732
C	-1.1197	0.0387	-0.1170
C	-2.3279	1.4781	-1.8737
C	-1.5794	2.6459	-1.6765
C	-1.5793	3.6550	-2.6337
C	-2.3207	3.5158	-3.8057
C	-3.0630	2.3556	-4.0124
C	-3.0654	1.3445	-3.0557
O	0.0363	-0.2945	-0.1850
H	-3.0047	-0.4529	-1.1003
H	-2.7947	0.0024	1.3073
H	-2.0366	1.6583	1.0221
H	-0.9878	2.7730	-0.7729
H	-0.9936	4.5543	-2.4639
H	-2.3192	4.3056	-4.5513
H	-3.6467	2.2363	-4.9212
H	-3.6497	0.4428	-3.2209
C	-0.9310	-3.6130	2.3998
C	0.3363	-4.1445	2.6658
C	0.6169	-4.6806	3.9170
C	-0.3682	-4.6849	4.9025
C	-1.6346	-4.1540	4.6414
C	-1.9186	-3.6187	3.3948
H	1.0961	-4.1327	1.8873
H	1.5991	-5.0938	4.1262
H	-0.1509	-5.1032	5.8815
H	-2.3949	-4.1614	5.4170
H	-2.8965	-3.2008	3.1736
O	-2.2571	-2.5519	0.7254
C	-1.1944	-3.0468	1.0711
H	-0.3367	-3.0861	0.3671

Ia

E(r^2scan-3c) = -768.361773 (conv)

Lowest Freq. = 3.32 cm^{-1}

32

C	-2.3645	0.8034	-0.4441
C	-2.3725	1.5807	0.9304
C	-1.1968	0.8728	0.4589
C	-2.3459	1.4949	-1.7567
C	-1.7357	2.7444	-1.9282
C	-1.7225	3.3598	-3.1756
C	-2.3132	2.7392	-4.2750
C	-2.9178	1.4947	-4.1150
C	-2.9326	0.8771	-2.8677
O	-0.0035	0.7193	0.5475
H	-2.9414	-0.1179	-0.4083
H	-2.9994	1.1377	1.6988
H	-2.3420	2.6703	0.9145
H	-1.2638	3.2448	-1.0861
H	-1.2455	4.3295	-3.2892
H	-2.3021	3.2225	-5.2477
H	-3.3839	1.0019	-4.9640
H	-3.4094	-0.0927	-2.7485
C	-0.7801	-3.2472	2.7274
C	0.4943	-3.7570	3.0029
C	0.6288	-4.9675	3.6725
C	-0.5091	-5.6686	4.0664
C	-1.7833	-5.1630	3.7931
C	-1.9221	-3.9561	3.1259
H	1.3738	-3.1989	2.6894
H	1.6159	-5.3655	3.8876
H	-0.4060	-6.6152	4.5897
H	-2.6638	-5.7175	4.1045
H	-2.9041	-3.5476	2.9052
O	-1.9437	-1.4224	1.7275
C	-0.8889	-1.9663	2.0201
H	0.0769	-1.4893	1.7499

TS2a

E(r^2scan-3c) = -768.345203 (conv)

Lowest Freq. = -340.25 cm^{-1}

32

C	-2.7863	0.6904	-0.0284
C	-2.5777	1.4389	1.2522
C	-1.3542	0.6209	1.0431
C	-2.5051	1.1961	-1.3512
C	-1.8609	2.4264	-1.5920
C	-1.6022	2.8492	-2.8897
C	-1.9712	2.0644	-3.9821
C	-2.6051	0.8405	-3.7605
C	-2.8629	0.4101	-2.4673
O	-0.2088	0.9562	0.7120
H	-3.3875	-0.2107	0.0343
H	-3.2341	1.1562	2.0718
H	-2.3921	2.5087	1.1828
H	-1.5588	3.0541	-0.7600
H	-1.1064	3.8029	-3.0510
H	-1.7672	2.4019	-4.9941
H	-2.8981	0.2191	-4.6026
H	-3.3561	-0.5451	-2.3019
C	-0.6309	-2.7469	2.3597
C	0.5457	-3.5048	2.5486

C	0.4727	-4.7860	3.0693
C	-0.7657	-5.3338	3.4084
C	-1.9368	-4.5917	3.2233
C	-1.8801	-3.3107	2.7048
H	1.5064	-3.0718	2.2820
H	1.3807	-5.3640	3.2127
H	-0.8208	-6.3390	3.8159
H	-2.8976	-5.0239	3.4872
H	-2.7884	-2.7346	2.5594
O	-1.5539	-0.6996	1.6540
C	-0.5036	-1.4365	1.8282
H	0.4582	-1.0138	1.5410

IIa

E(r^2scan-3c) = -768.351473 (conv)

Lowest Freq. = 19.56 cm^{-1}

32

C	-3.0967	0.7781	-0.0133
C	-2.6879	1.4376	1.2580
C	-1.3327	0.8008	1.3689
C	-2.6363	1.1441	-1.2958
C	-1.8178	2.2822	-1.5410
C	-1.4137	2.5998	-2.8272
C	-1.7984	1.8124	-3.9155
C	-2.6010	0.6866	-3.6978
C	-3.0082	0.3529	-2.4203
O	-0.2507	1.2584	1.0714
H	-3.6976	-0.1231	0.0715
H	-3.3387	1.1672	2.0912
H	-2.5727	2.5223	1.2034
H	-1.5059	2.9128	-0.7152
H	-0.7903	3.4753	-2.9888
H	-1.4776	2.0714	-4.9201
H	-2.9036	0.0667	-4.5377
H	-3.6280	-0.5256	-2.2576
C	-0.5721	-2.6431	2.2977
C	0.5828	-3.4593	2.4118
C	0.4777	-4.7637	2.8583
C	-0.7688	-5.2945	3.2043
C	-1.9156	-4.5004	3.0967
C	-1.8314	-3.1940	2.6511
H	1.5516	-3.0468	2.1422
H	1.3702	-5.3775	2.9406
H	-0.8468	-6.3192	3.5552
H	-2.8841	-4.9129	3.3655
H	-2.7253	-2.5839	2.5705
O	-1.4945	-0.5576	1.6998
C	-0.4194	-1.3243	1.8310
H	0.5395	-0.8967	1.5542

TS3a

E(r^2scan-3c) = -768.330741 (conv)

Lowest Freq. = -31.83 cm^{-1}

32

C	2.1296	-0.0817	0.1414
C	0.8166	-0.2520	0.8198
C	-0.2568	-0.6928	-0.1470
C	3.3619	-0.5120	0.6313
C	4.5437	-0.2827	-0.1465
C	5.7782	-0.7251	0.2773
C	5.9118	-1.4083	1.4946
C	4.7770	-1.6324	2.2832
C	3.5312	-1.1945	1.8780
O	-0.1385	-1.5081	-1.0263
H	2.1233	0.4719	-0.7954
H	0.4677	0.6649	1.3214
H	0.8590	-1.0302	1.5948
H	4.4462	0.2430	-1.0931
H	6.6552	-0.5412	-0.3377
H	6.8866	-1.7521	1.8265
H	4.8770	-2.1500	3.2337
H	2.6758	-1.3642	2.5239
C	-2.5762	-0.5174	-0.4987

C	-3.5923	0.3874	-0.7851
C	-4.7949	-0.0898	-1.3889
C	-5.7178	2.1423	-1.3745
C	-4.5437	2.6280	-0.7856
C	-5.8294	0.7771	-1.6721
C	-3.4936	1.7806	-0.4919
O	-1.4583	-0.0764	0.1529
H	-2.5864	2.1670	-0.0391
H	-4.8846	-1.1481	-1.6199
H	-6.7376	0.3967	-2.1315
H	-4.4550	3.6869	-0.5575
H	-6.5359	2.8193	-1.6006
H	-2.6208	-1.5742	-0.7383

IIIa

E(r^2scan-3c) = -768.339765 (conv)

Lowest Freq. = 16.96 cm^{-1}

32

C	2.6972	1.1830	0.5428
C	1.2382	1.4047	0.5084
C	0.4657	0.5435	-0.4602
C	3.3189	-0.0522	0.7627
C	4.7452	-0.1401	0.7267
C	5.3981	-1.3311	0.9688
C	4.6728	-2.4938	1.2616
C	3.2760	-2.4358	1.3106
C	2.6047	-1.2528	1.0622
O	0.8860	-0.1815	-1.3256
H	3.3361	2.0463	0.3788
H	0.9984	2.4503	0.2798
H	0.7661	1.2094	1.4883
H	5.3145	0.7587	0.5021
H	6.4838	-1.3665	0.9312
H	5.1895	-3.4300	1.4499
H	2.7079	-3.3327	1.5438
H	1.5206	-1.2410	1.1185
C	-1.7746	-0.0573	-0.9013
C	-3.1221	0.3131	-0.8742
C	-4.0560	-0.4455	-1.6358
C	-5.8627	0.9561	-0.8605
C	-4.9563	1.7144	-0.1101
C	-5.3987	-0.1242	-1.6209
C	-3.6087	1.4097	-0.1077
O	-0.8918	0.6276	-0.1468
H	-2.9132	2.0050	0.4749
H	-3.6976	-1.2843	-2.2268
H	-6.0983	-0.7140	-2.2066
H	-5.3152	2.5549	0.4777
H	-6.9193	1.2056	-0.8542
H	-1.3859	-0.8675	-1.5092

TS4a

E(r^2scan-3c) = -768.327947 (conv)

Lowest Freq. = -41.66 cm^{-1}

32

C	1.7443	0.5269	0.9485
C	0.6644	0.2159	1.9293
C	-0.3504	-0.7492	1.3321
C	2.9041	-0.2347	0.7580
C	3.8712	0.1894	-0.2074
C	5.0289	-0.5281	-0.4298
C	5.2924	-1.7000	0.2912
C	4.3641	-2.1366	1.2441
C	3.1986	-1.4322	1.4799
O	-0.1840	-1.9215	1.1474
H	1.6163	1.4241	0.3487
H	0.1438	1.1241	2.2416
H	1.0658	-0.2891	2.8157

H	3.6764	1.0981	-0.7722
H	5.7423	-0.1780	-1.1716
H	6.2051	-2.2612	0.1157
H	4.5601	-3.0444	1.8091
H	2.4955	-1.8016	2.2196
C	-2.1132	-0.4883	-0.1810
C	-3.0732	0.3344	-0.7633
C	-3.6877	-0.0863	-1.9802
C	-5.0694	1.8939	-1.9912
C	-4.4711	2.3240	-0.8002
C	-4.6679	0.6832	-2.5714
C	-3.4917	1.5691	-0.1857
O	-1.5289	-0.0988	0.9899
H	-3.0311	1.9110	0.7352
H	-3.3761	-1.0244	-2.4322
H	-5.1286	0.3480	-3.4964
H	-4.7803	3.2644	-0.3520
H	-5.8391	2.4966	-2.4634
H	-1.7806	-1.4286	-0.6081

2a-*trans*
E(r^2scan-3c) = -768.453022 (conv)
Lowest Freq. = 8.46 cm^{-1}
32

C	0.5000	0.3936	0.4605
C	0.1328	0.6609	1.9208
C	-0.6469	-0.5610	2.3422
C	1.8430	-0.2706	0.2585
C	2.5523	-0.0185	-0.9199
C	3.7652	-0.6527	-1.1723
C	4.2870	-1.5549	-0.2480
C	3.5883	-1.8138	0.9283
C	2.3760	-1.1759	1.1807
O	-0.9195	-0.9438	3.4542
H	0.4613	1.3064	-0.1402
H	-0.5391	1.5236	2.0120
H	0.9837	0.8275	2.5845
H	2.1490	0.6867	-1.6436
H	4.3057	-0.4383	-2.0902
H	5.2348	-2.0494	-0.4409
H	3.9886	-2.5129	1.6574
H	1.8556	-1.3912	2.1108
C	-0.6686	-0.5496	0.0248
C	-1.8392	0.1935	-0.5695
C	-1.7483	0.6043	-1.9030
C	-3.9110	1.6607	-1.7437
C	-4.0072	1.2491	-0.4180
C	-2.7767	1.3348	-2.4867
C	-2.9761	0.5171	0.1696
O	-1.0536	-1.2448	1.2394
H	-3.0712	0.1901	1.2008
H	-0.8658	0.3466	-2.4855
H	-2.6969	1.6449	-3.5248
H	-4.8907	1.4932	0.1653
H	-4.7178	2.2274	-2.1997
H	-0.3136	-1.3152	-0.6727

A-TBS
E(r^2scan-3c) = -949.809123 (conv)
Lowest Freq. = 18.87 cm^{-1}
40

C	-2.2499	1.3917	2.3020
C	-2.9976	0.4156	3.4087
C	-2.9264	0.1576	2.0233
C	-0.7910	1.5248	2.1002
C	-0.2708	2.7574	1.6915
C	1.0962	2.9010	1.4845
C	1.9516	1.8193	1.6838
C	1.4372	0.5921	2.0981
C	0.0714	0.4412	2.3038
O	-2.8591	-0.8501	1.2816
H	-2.8347	2.3084	2.2673
H	-3.8117	0.9944	3.8339
H	-2.4828	-0.2760	4.0779
H	-0.9397	3.6001	1.5378
H	1.4950	3.8611	1.1707
H	3.0194	1.9339	1.5226
H	2.1019	-0.2507	2.2631
H	-0.3148	-0.5207	2.6339
Si	-2.3837	-0.9607	-0.4560
C	-3.9200	-1.7758	-1.1805
C	-0.8773	-2.0408	-0.3875
C	-2.0655	0.7673	-1.0559
C	-3.6968	-1.9168	-2.6995
C	-5.1583	-0.9016	-0.9238
C	-4.1275	-3.1686	-0.5627
H	-2.8901	1.4489	-0.8223
H	-1.9562	0.7389	-2.1467
H	-0.0229	-1.4947	0.0249
H	-0.6163	-2.3629	-1.4024
H	-1.0529	-2.9350	0.2187
H	-2.8130	-2.5228	-2.9317
H	-3.5880	-0.9433	-3.1908
H	-4.5664	-2.4173	-3.1454
H	-5.3671	-0.7934	0.1469
H	-6.0370	-1.3703	-1.3861
H	-5.0499	0.0992	-1.3573
H	-3.2786	-3.8315	-0.7628
H	-5.0220	-3.6305	-1.0013
H	-4.2769	-3.1172	0.5216
H	-1.1390	1.1740	-0.6392

Ib
E(r^2scan-3c) = -1295.350881 (conv)
Lowest Freq. = 16.48 cm^{-1}
54

C	-2.5933	0.6895	-0.2185
C	-2.0924	1.5448	0.9355
C	-1.2949	0.3756	0.4736
C	-2.5108	1.1425	-1.6353
C	-2.5188	2.4935	-1.9833
C	-2.4127	2.8767	-3.3189
C	-2.2948	1.9136	-4.3169
C	-2.2909	0.5615	-3.9757
C	-2.3984	0.1787	-2.6437
O	-0.1213	0.4096	-0.1692
Si	1.2764	1.3249	0.2536
C	2.5584	0.6453	-0.9546
C	0.9219	3.1367	-0.0300
C	1.6546	0.9785	2.0508
C	3.9383	1.2165	-0.5834
C	2.6111	-0.8898	-0.8816
C	2.1904	1.0753	-2.3857
H	-3.4145	0.0171	0.0227
H	-2.6153	1.4590	1.8824
H	-1.6782	2.5214	0.7101
H	-2.6159	3.2528	-1.2122
H	-2.4239	3.9316	-3.5782
H	-2.2093	2.2137	-5.3574
H	-2.2010	-0.1955	-4.7497
H	-2.3898	-0.8756	-2.3766
H	0.3243	3.2856	-0.9362
H	1.8647	3.6819	-0.1552
H	0.3926	3.5858	0.8167
H	0.7718	1.1748	2.6723
H	1.9738	-0.0557	2.2212
H	2.4554	1.6392	2.4019
H	3.9520	2.3125	-0.6139
H	4.2536	0.8993	0.4177
H	4.6912	0.8576	-1.2984
H	1.6663	-1.3415	-1.2050
H	2.8387	-1.2449	0.1315
H	3.4012	-1.2657	-1.5462
H	1.1984	0.7088	-2.6771
H	2.1958	2.1658	-2.4936
H	2.9210	0.6660	-3.0970
C	-0.6763	-2.7961	2.2968
C	0.3644	-3.7495	2.2774

C	0.2894	-4.8598	3.0991
C	-0.8138	-5.0229	3.9375
C	-1.8494	-4.0797	3.9619
C	-1.7902	-2.9669	3.1488
H	1.2145	-3.6023	1.6164
H	1.0833	-5.5993	3.0920
H	-0.8719	-5.8948	4.5828
H	-2.6980	-4.2263	4.6222
H	-2.5846	-2.2277	3.1574
O	-1.4355	-0.7723	1.3920
C	-0.5412	-1.6829	1.4350
H	0.3319	-1.5849	0.7875

TS2b
E(r^2scan-3c) = -1295.314980 (conv)
Lowest Freq. = -328.67 cm^{-1}
54

C	-2.1538	-0.2184	-0.1231
C	-2.2946	1.0814	0.6062
C	-0.8950	0.6734	0.8103
C	-1.8323	-0.3668	-1.5417
C	-1.6044	0.7207	-2.3972
C	-1.2948	0.5093	-3.7357
C	-1.1978	-0.7847	-4.2422
C	-1.4236	-1.8738	-3.4008
C	-1.7386	-1.6675	-2.0647
O	0.0809	1.1835	0.1102
Si	1.7520	1.4578	0.5052
C	2.4059	2.1710	-1.1141
C	1.7614	2.6547	1.9326
C	2.5509	-0.1685	0.9514
C	3.8642	2.6158	-0.8976
C	2.3559	1.0973	-2.2149
C	1.5653	3.3848	-1.5447
H	-2.6666	-1.0567	0.3355
H	-2.9326	1.0566	1.4853
H	-2.3831	1.9886	0.0114
H	-1.6758	1.7380	-2.0264
H	-1.1266	1.3625	-4.3869
H	-0.9531	-0.9446	-5.2882
H	-1.3572	-2.8860	-3.7893
H	-1.9190	-2.5177	-1.4105
H	1.2398	3.5844	1.6825
H	2.7919	2.9051	2.2092
H	1.2784	2.2108	2.8102
H	2.2164	-0.5286	1.9297
H	2.3517	-0.9404	0.2006
H	3.6375	-0.0297	1.0029
H	3.9413	3.4073	-0.1431
H	4.5070	1.7832	-0.5881
H	4.2695	3.0136	-1.8378
H	1.3364	0.7271	-2.3750
H	2.9996	0.2424	-1.9784
H	2.7073	1.5257	-3.1637
H	0.5225	3.1086	-1.7356
H	1.5771	4.1777	-0.7880
H	1.9752	3.8057	-2.4730
C	-1.3407	-2.2428	2.2887
C	-1.2445	-3.5905	1.8773
C	-1.8851	-4.5749	2.6063
C	-2.6252	-4.2292	3.7398
C	-2.7301	-2.8960	4.1484
C	-2.1000	-1.8988	3.4277
H	-0.6598	-3.8411	0.9961
H	-1.8127	-5.6136	2.3001
H	-3.1293	-5.0052	4.3086
H	-3.3134	-2.6438	5.0283
H	-2.1849	-0.8584	3.7241
O	-0.5524	0.0035	2.0014
C	-0.6561	-1.2650	1.5305
H	-0.0067	-1.5457	0.7029

Pb-*trans*
E(r^2scan-3c) = -1295.416572 (conv)
Lowest Freq. = 14.64
54

C	-2.3605	-0.6491	-0.3619
C	-1.7364	0.7386	-0.1427
C	-0.2990	0.4363	0.0426
C	-2.5552	-1.0143	-1.8154
C	-1.6122	-0.6849	-2.7938
C	-1.7919	-1.0914	-4.1129
C	-2.9173	-1.8294	-4.4716
C	-3.8622	-2.1594	-3.5037
C	-3.6803	-1.7555	-2.1839
O	0.6315	1.2820	-0.0097
Si	2.4001	1.0180	0.2044
C	3.0433	2.7555	-0.1127
C	2.5862	0.4208	1.9532
C	2.8596	-0.2309	-1.0923
C	2.4538	3.7392	0.9123
C	4.5776	2.7244	0.0320
C	2.6743	3.2074	-1.5363
H	-3.3134	-0.7329	0.1651
H	-2.0903	1.2120	0.7840
H	-1.8930	1.4521	-0.9558
H	-0.7267	-0.1026	-2.5465
H	-1.0520	-0.8258	-4.8626
H	-3.0590	-2.1409	-5.5025
H	-4.7460	-2.7293	-3.7761
H	-4.4211	-2.0142	-1.4309
H	2.0716	1.0818	2.6584
H	3.6495	0.4061	2.2200
H	2.1959	-0.5950	2.0722
H	2.4974	-1.2297	-0.8310
H	2.4573	0.0457	-2.0727
H	3.9513	-0.2797	-1.1790
H	1.3608	3.7892	0.8482
H	2.7268	3.4699	1.9386
H	2.8454	4.7463	0.7167
H	5.0439	2.0433	-0.6894
H	4.8889	2.4263	1.0400
H	4.9786	3.7293	-0.1550
H	3.1034	2.5461	-2.2974
H	1.5890	3.2451	-1.6845
H	3.0696	4.2169	-1.7113
C	-1.6430	-1.9444	1.7558
C	-2.3958	-3.0996	1.9819
C	-2.7915	-3.4343	3.2722
C	-2.4281	-2.6250	4.3466
C	-1.6684	-1.4800	4.1244
C	-1.2785	-1.1355	2.8326
H	-2.6720	-3.7371	1.1451
H	-3.3754	-4.3345	3.4396
H	-2.7298	-2.8909	5.3554
H	-1.3735	-0.8507	4.9589
H	-0.6758	-0.2438	2.6839
O	-0.0530	-0.8137	0.2825
C	-1.3263	-1.5906	0.3320
H	-1.1298	-2.4847	-0.2642

Ac
E(r^2scan-3c) = -895.128200 (conv)
Lowest Freq. = 13.73 cm^{-1}
45

C	-2.3388	1.6033	1.8392
C	-3.6231	1.5188	2.7719
C	-3.4380	0.6633	1.6299
C	-0.9940	1.1002	2.2215
C	0.1449	1.7118	1.6872
C	1.4146	1.2400	2.0074
C	1.5629	0.1524	2.8646
C	0.4322	-0.4594	3.4025
C	-0.8385	0.0065	3.0818
O	-3.7725	-0.3699	1.0846
H	-2.3495	2.5096	1.2351

H	-4.2588	2.3978	2.6973
H	-3.5682	1.0600	3.7600
H	0.0335	2.5627	1.0199
H	2.2903	1.7274	1.5880
H	2.5537	-0.2143	3.1165
H	0.5392	-1.3046	4.0765
H	-1.7081	-0.4880	3.5086
Li	-2.9958	-1.5843	-0.1677
C	0.2324	-3.6585	1.2101
C	1.0402	-2.7793	0.2491
C	0.0271	-1.7176	-0.1687
O	-1.2872	-2.3411	-0.0150
C	-1.1289	-3.6788	0.5380
H	0.1617	-3.1882	2.1971
H	0.6507	-4.6619	1.3250
H	1.3663	-3.3651	-0.6172
H	1.9206	-2.3322	0.7186
H	0.0586	-0.8413	0.4881
H	0.1367	-1.3993	-1.2105
H	-1.1609	-4.4098	-0.2820
H	-1.9659	-3.8611	1.2198
H	-4.4171	-3.5422	-2.9045
C	-3.8997	-3.7486	-1.9572
H	-2.8190	-3.7167	-2.1186
H	-3.6150	-5.5042	-0.7175
C	-4.4085	-5.0348	-1.3056
O	-4.2349	-2.7025	-1.0137
H	-4.7440	-5.7526	-2.0583
C	-5.5609	-4.5579	-0.3824
C	-5.5640	-3.0329	-0.5404
H	-5.3650	-4.8403	0.6552
H	-6.2925	-2.7070	-1.2952
H	-5.7306	-2.4824	0.3884
H	-6.5263	-4.9806	-0.6717

Ic

E(r^{2}scan-3c) = -1240.647817 (conv)

Lowest Freq. = 10.31 cm^{-1}

59

C	-1.2619	1.6311	2.7802
C	-1.9772	1.1952	4.1309
C	-2.4146	0.7196	2.8382
C	0.0564	1.1170	2.3353
C	0.9030	1.9545	1.6002
C	2.1365	1.4967	1.1476
C	2.5422	0.1921	1.4186
C	1.7052	-0.6495	2.1491
C	0.4706	-0.1949	2.6020
O	-2.8492	-0.2846	2.2801
H	-1.4597	2.6738	2.5481
H	-2.4833	2.0141	4.6308
H	-1.4985	0.4605	4.7789
H	0.5909	2.9744	1.3891
H	2.7830	2.1625	0.5824
H	3.5054	-0.1662	1.0670
H	2.0162	-1.6666	2.3711
H	-0.1630	-0.8660	3.1778
Li	-2.0206	-0.8866	0.6748
C	-1.6767	2.2181	-2.1118
C	-0.2195	1.7576	-1.9997
C	-0.3596	0.3530	-1.4264
O	-1.5997	0.3663	-0.6528
C	-2.2824	1.6372	-0.8454
H	-2.1535	1.7827	-2.9973
H	-1.7802	3.3054	-2.1551
H	0.3290	2.3999	-1.3021
H	0.3048	1.7553	-2.9588
H	-0.4635	-0.4038	-2.2137
H	0.4597	0.0768	-0.7541
H	-2.0886	2.2781	0.0260
H	-3.3558	1.4335	-0.9173
C	-0.2268	-4.3307	1.9665
C	0.1609	-4.4676	0.4904
C	-0.1771	-3.0887	-0.0599
O	-1.2975	-2.6124	0.7464
C	-1.5021	-3.5094	1.8787
H	0.5446	-3.7808	2.5178
H	-0.3915	-5.2930	2.4582
H	-0.4521	-5.2358	0.0060
H	1.2150	-4.7172	0.3439
H	0.6568	-2.3864	0.0686
H	-0.4912	-3.0961	-1.1083
H	-2.3759	-4.1397	1.6678
H	-1.7014	-2.8966	2.7632
C	-4.8922	1.8109	1.6827
C	-6.0063	2.5528	1.1061
C	-6.9018	1.8697	0.2721
C	-8.1451	3.9055	-0.0375
C	-7.2537	4.5905	0.7938
C	-7.9707	2.5478	-0.2991
C	-6.1853	3.9203	1.3661
O	-4.0375	2.2979	2.4164
H	-5.4833	4.4365	2.0142
H	-6.7528	0.8099	0.0784
H	-8.6677	2.0231	-0.9453
H	-7.4008	5.6484	0.9898
H	-8.9814	4.4371	-0.4828
H	-4.8348	0.7355	1.4165

TS2c

E(r^{2}scan-3c) = -1240.647180 (conv)

Lowest Freq. = -107.97 cm^{-1}

59

C	-1.4148	1.8214	2.8604
C	-2.2021	1.3175	4.1074
C	-2.6384	0.9507	2.7541
C	-0.1170	1.2545	2.4276
C	0.7386	2.0320	1.6359
C	1.9423	1.5167	1.1671
C	2.3129	0.2082	1.4707
C	1.4707	-0.5752	2.2558
C	0.2678	-0.0596	2.7319
O	-2.8481	-0.1406	2.1695
H	-1.5401	2.8839	2.6700
H	-2.7158	2.0928	4.6662
H	-1.7696	0.5135	4.6993
H	0.4563	3.0537	1.3931
H	2.5937	2.1400	0.5603
H	3.2520	-0.1954	1.1032
H	1.7516	-1.5943	2.5073
H	-0.3649	-0.6848	3.3571
Li	-1.6864	-0.7569	0.8243
C	-1.5673	2.1896	-2.1564
C	-0.1088	1.7237	-2.1017
C	-0.2454	0.3048	-1.5691
O	-1.4203	0.3260	-0.7040
C	-2.1394	1.5795	-0.8860
H	-2.0694	1.7771	-3.0388
H	-1.6708	3.2777	-2.1704
H	0.4624	2.3404	-1.3991
H	0.3872	1.7491	-3.0755
H	-0.4284	-0.4164	-2.3763
H	0.6114	-0.0223	-0.9710
H	-1.9539	2.2180	-0.0111
H	-3.2083	1.3499	-0.9505
C	-0.2341	-4.4213	1.8999
C	0.1246	-4.5280	0.4141
C	-0.1064	-3.1043	-0.0745
O	-1.1784	-2.5752	0.7619
C	-1.4380	-3.4940	1.8640
H	0.5874	-3.9621	2.4618
H	-0.4727	-5.3865	2.3542
H	-0.5549	-5.2226	-0.0924
H	1.1527	-4.8568	0.2420
H	0.7829	-2.4766	0.0717
H	-0.4277	-3.0427	-1.1190
H	-2.3643	-4.0423	1.6474
H	-1.5728	-2.9009	2.7739
C	-4.8020	1.6817	1.5941
C	-5.9099	2.4625	1.1129

C	-6.8166	1.8454	0.2346
C	-8.0723	3.8925	0.1249
C	-7.1718	4.5113	0.9986
C	-7.8964	2.5634	-0.2570
C	-6.0917	3.8045	1.4948
O	-3.9415	2.1341	2.3734
H	-5.3844	4.2708	2.1736
H	-6.6649	0.8079	-0.0523
H	-8.6010	2.0929	-0.9354
H	-7.3225	5.5472	1.2863
H	-8.9182	4.4551	-0.2599
H	-4.6991	0.6377	1.2593

IIc

E(r^2scan-3c) = -1240.647741 (conv)

Lowest Freq. = 17.68 cm^{-1}

59

C	-1.4911	1.8609	2.9041
C	-2.2882	1.2963	4.0853
C	-2.7525	1.0011	2.7025
C	-0.2010	1.3064	2.4417
C	0.6188	2.0921	1.6191
C	1.8171	1.5950	1.1191
C	2.2187	0.2947	1.4197
C	1.4138	-0.4967	2.2351
C	0.2173	0.0023	2.7451
O	-2.8477	-0.1205	2.1021
H	-1.6104	2.9302	2.7495
H	-2.8174	2.0256	4.6904
H	-1.8754	0.4466	4.6222
H	0.3106	3.1063	1.3758
H	2.4400	2.2254	0.4902
H	3.1532	-0.0956	1.0269
H	1.7199	-1.5090	2.4850
H	-0.3852	-0.6290	3.3927
Li	-1.6400	-0.7543	0.8421
C	-1.5561	2.1185	-2.2172
C	-0.1018	1.6374	-2.1908
C	-0.2439	0.2261	-1.6407
O	-1.3876	0.2753	-0.7378
C	-2.1071	1.5273	-0.9279
H	-2.0815	1.7025	-3.0842
H	-1.6488	3.2074	-2.2405
H	0.4915	2.2538	-1.5064
H	0.3725	1.6479	-3.1756
H	-0.4640	-0.4987	-2.4357
H	0.6263	-0.1109	-1.0682
H	-1.9056	2.1778	-0.0653
H	-3.1775	1.3004	-0.9719
C	-0.1932	-4.4088	1.9569
C	0.1661	-4.5385	0.4730
C	-0.0690	-3.1235	-0.0383
O	-1.1425	-2.5841	0.7883
C	-1.3992	-3.4846	1.9056
H	0.6271	-3.9392	2.5117
H	-0.4300	-5.3672	2.4264
H	-0.5116	-5.2429	-0.0223
H	1.1950	-4.8676	0.3062
H	0.8187	-2.4909	0.0978
H	-0.3893	-3.0795	-1.0841
H	-2.3244	-4.0386	1.6993
H	-1.5351	-2.8764	2.8053
C	-4.7829	1.6025	1.5435
C	-5.8597	2.4373	1.1209
C	-6.7871	1.8948	0.2106
C	-7.9920	3.9749	0.2290
C	-7.0731	4.5201	1.1327
C	-7.8498	2.6662	-0.2312
C	-6.0088	3.7614	1.5815
O	-3.9101	2.0112	2.3641
H	-5.2892	4.1729	2.2821
H	-6.6620	0.8728	-0.1370
H	-8.5684	2.2535	-0.9320
H	-7.1976	5.5407	1.4808
H	-8.8255	4.5791	-0.1176
H	-4.6796	0.5805	1.1646

TS3c

E(r^2scan-3c) = -1240.623173 (conv)

Lowest Freq. = -430.93 cm^{-1}

59

C	-1.9910	0.4547	0.5708
C	-2.2827	1.2161	1.8331
C	-0.8549	0.8371	1.8989
C	-1.5901	1.0579	-0.6896
C	-1.3919	2.4421	-0.8581
C	-0.9440	2.9578	-2.0723
C	-0.6823	2.1133	-3.1483
C	-0.8856	0.7404	-2.9997
C	-1.3291	0.2211	-1.7918
O	0.1660	1.5340	1.7136
H	-2.4126	-0.5439	0.5105
H	-2.9664	0.7326	2.5260
H	-2.4254	2.2932	1.7554
H	-1.6175	3.1296	-0.0467
H	-0.8074	4.0313	-2.1767
H	-0.3308	2.5182	-4.0927
H	-0.6935	0.0697	-3.8330
H	-1.4784	-0.8515	-1.6856
C	-0.8624	-2.6912	2.0974
C	-0.4934	-3.7010	1.1820
C	-0.8429	-5.0155	1.4324
C	-1.5607	-5.3343	2.5875
C	-1.9365	-4.3385	3.4954
C	-1.5989	-3.0200	3.2567
H	0.0679	-3.4350	0.2900
H	-0.5610	-5.7972	0.7343
H	-1.8354	-6.3673	2.7808
H	-2.5008	-4.6025	4.3843
H	-1.8965	-2.2350	3.9445
O	-0.6559	-0.4055	2.6974
C	-0.4653	-1.3606	1.8212
H	0.0956	-1.1270	0.9164
Li	0.9669	2.4894	0.3328
C	3.1024	1.0762	-2.9472
C	3.4267	-0.0492	-1.9596
C	2.4094	0.1965	-0.8545
O	2.2055	1.6405	-0.8306
C	2.8119	2.2395	-2.0123
H	2.2093	0.8276	-3.5321
H	3.9240	1.2965	-3.6337
H	4.4466	0.0627	-1.5744
H	3.3243	-1.0465	-2.3953
H	1.4461	-0.2805	-1.0806
H	2.7483	-0.1155	0.1385
H	3.7331	2.7534	-1.7068
H	2.1044	2.9676	-2.4219
C	1.5824	6.6836	0.4188
C	0.2570	6.4819	1.1600
C	0.3948	5.0521	1.6569
O	1.1417	4.3592	0.6145
C	1.7764	5.3356	-0.2632
H	2.3921	6.8725	1.1325
H	1.5531	7.5053	-0.3014
H	-0.5883	6.5665	0.4670
H	0.1109	7.1898	1.9800
H	0.9698	5.0010	2.5910
H	-0.5586	4.5316	1.7918
H	1.2727	5.2983	-1.2381
H	2.8267	5.0531	-0.3868

Pc-*trans*

E(r^2scan-3c) = -1240.733418 (conv)

Lowest Freq. = 9.72 cm^{-1}

59

C	-2.1341	2.8272	2.4729
C	-1.4161	1.7113	3.2347
C	-1.3819	0.5827	2.2476

C	-1.2225	3.7330	1.6741
C	-1.6970	4.9926	1.2928
C	-0.9264	5.8322	0.4958
C	0.3333	5.4225	0.0615
C	0.8154	4.1726	0.4371
C	0.0440	3.3348	1.2406
O	-0.6605	-0.4031	2.2352
H	-2.7593	3.4322	3.1350
H	-2.0087	1.3733	4.0948
H	-0.4168	1.9586	3.5973
H	-2.6802	5.3167	1.6281
H	-1.3078	6.8103	0.2164
H	0.9372	6.0777	-0.5596
H	1.7989	3.8458	0.1106
H	0.4547	2.3710	1.5292
Li	-0.6429	-1.6311	0.7731
C	1.3067	0.4590	-2.2868
C	1.7935	-0.9695	-2.5523
C	0.6950	-1.7950	-1.9039
O	0.2953	-1.0278	-0.7314
C	0.7428	0.3561	-0.8750
H	0.5172	0.7292	-2.9966
H	2.1037	1.2040	-2.3522
H	2.7522	-1.1508	-2.0533
H	1.9025	-1.1938	-3.6165
H	-0.1738	-1.9016	-2.5674
H	1.0180	-2.7834	-1.5632
H	1.5087	0.5441	-0.1118
H	-0.1138	1.0151	-0.6987
C	-3.7441	-4.3159	1.4848
C	-3.4645	-4.6434	0.0145
C	-2.0567	-4.0949	-0.1751
O	-1.9557	-2.9619	0.7410
C	-3.1203	-2.9368	1.6194
H	-3.2371	-5.0314	2.1421
H	-4.8098	-4.3086	1.7277
H	-4.1724	-4.1161	-0.6346
H	-3.5180	-5.7127	-0.2054
H	-1.2918	-4.8292	0.1069
H	-1.8590	-3.7329	-1.1891
H	-3.8026	-2.1496	1.2712
H	-2.7743	-2.6982	2.6300
C	-3.0557	1.9988	1.5211
C	-4.4160	1.6953	2.0904
C	-5.4377	2.6240	1.8743
C	-6.9503	1.2926	3.2002
C	-5.9373	0.3619	3.4118
C	-6.6971	2.4259	2.4294
C	-4.6729	0.5617	2.8617
O	-2.3018	0.7625	1.2908
H	-3.8984	-0.1812	3.0292
H	-5.2441	3.5049	1.2656
H	-7.4849	3.1525	2.2524
H	-6.1292	-0.5270	4.0060
H	-7.9357	1.1334	3.6286
H	-3.1496	2.4802	0.5438

Ad

E(r^2scan-3c) = -1127.549533 (conv)

Lowest Freq. = 19.79 cm^{-1}

58

C	-2.4547	1.3322	2.0991
C	-3.1444	0.8347	3.4405
C	-3.1306	0.0415	2.2370
C	-0.9837	1.3994	1.9096
C	-0.4308	2.4459	1.1654
C	0.9452	2.5237	0.9700
C	1.7867	1.5561	1.5130
C	1.2421	0.5097	2.2556
C	-0.1324	0.4288	2.4514
O	-3.2738	-1.0651	1.7594
H	-3.0268	2.1319	1.6306
H	-4.0300	1.4027	3.7155
H	-2.5299	0.4881	4.2725
H	-1.0831	3.2054	0.7417
H	1.3604	3.3458	0.3935
H	2.8605	1.6175	1.3616
H	1.8906	-0.2501	2.6830
H	-0.5383	-0.3986	3.0301
Li	-2.5716	-1.6242	0.0087
C	-1.8755	1.1587	-2.9014
C	-0.4168	1.0959	-2.4356
C	-0.4252	-0.0807	-1.4630
O	-1.7937	-0.1811	-0.9695
C	-2.6169	0.8217	-1.6192
H	-2.0732	0.3976	-3.6653
H	-2.1562	2.1379	-3.2985
H	-0.1481	2.0201	-1.9121
H	0.2903	0.9424	-3.2549
H	-0.1798	-1.0272	-1.9611
H	0.2415	0.0643	-0.6059
H	-2.6986	1.7000	-0.9627
H	-3.6099	0.3877	-1.7715
C	-0.9039	-4.3313	2.2584
C	0.4246	-4.0796	1.5375
C	0.0657	-2.9666	0.5556
O	-1.3684	-3.0926	0.3100
C	-1.8978	-4.1769	1.1207
H	-1.0825	-3.5657	3.0230
H	-0.9576	-5.3175	2.7271
H	0.7380	-4.9810	0.9995
H	1.2306	-3.7812	2.2130
H	0.2466	-1.9720	0.9809
H	0.5895	-3.0485	-0.4024
H	-1.9545	-5.0875	0.5077
H	-2.9057	-3.8961	1.4433
H	-4.5598	-2.3305	-3.0395
C	-3.8055	-2.7247	-2.3424
H	-2.8160	-2.3586	-2.6324
H	-2.9430	-4.6446	-1.8681
C	-3.9030	-4.2401	-2.2031
O	-4.0865	-2.2272	-1.0154
H	-4.1665	-4.7201	-3.1493
C	-4.9865	-4.4334	-1.1138
C	-5.1924	-3.0236	-0.5283
H	-4.6522	-5.1382	-0.3481
H	-6.1294	-2.5752	-0.8852
H	-5.1687	-2.9835	0.5639
H	-5.9212	-4.8120	-1.5354

TS1d

E(r^2scan-3c) = 1473.066492 (conv)

Lowest Freq. = -3.26 cm^{-1}

72

C	-1.2318	1.3540	2.9339
C	-1.9273	0.7173	4.2140
C	-2.3105	0.3622	2.8679
C	0.1372	1.0093	2.4794
C	0.9546	2.0141	1.9509
C	2.2381	1.7209	1.4992
C	2.7234	0.4174	1.5660
C	1.9158	-0.5902	2.0920
C	0.6325	-0.2998	2.5428
O	-2.7618	-0.5392	2.1810
H	-1.5156	2.3963	2.8127
H	-2.5078	1.4308	4.7903
H	-1.4006	-0.0459	4.7884
H	0.5797	3.0333	1.8979
H	2.8604	2.5146	1.0947
H	3.7249	0.1871	1.2142
H	2.2869	-1.6098	2.1507
H	0.0176	-1.1014	2.9463
Li	-2.2714	-0.8777	0.3232
C	-1.5448	2.6007	-1.8449
C	-0.0799	2.1875	-1.6687
C	-0.1988	0.7372	-1.2135
O	-1.4854	0.6400	-0.5367
C	-2.2027	1.8944	-0.6711
H	-1.9444	2.2187	-2.7916
H	-1.6932	3.6833	-1.8117

H	0.3917	2.7922	-0.8862
H	0.5080	2.2817	-2.5854
H	-0.2095	0.0421	-2.0632
H	0.5840	0.4414	-0.5074
H	-2.0901	2.4693	0.2593
H	-3.2620	1.6616	-0.8277
C	-0.0274	-4.2315	1.4046
C	0.4198	-4.1583	-0.0587
C	-0.0028	-2.7482	-0.4461
O	-1.1989	-2.4726	0.3363
C	-1.3569	-3.4937	1.3630
H	0.6837	-3.7023	2.0495
H	-0.1368	-5.2556	1.7711
H	-0.1173	-4.9007	-0.6599
H	1.4941	-4.3133	-0.1893
H	0.7652	-2.0119	-0.1743
H	-0.2553	-2.6368	-1.5055
H	-2.1788	-4.1595	1.0666
H	-1.6175	-2.9944	2.3014
C	-4.8257	1.6295	1.6517
C	-5.8486	2.3887	0.9342
C	-6.5611	1.7401	-0.0821
C	-7.7909	3.7646	-0.4973
C	-7.0816	4.4157	0.5163
C	-7.5319	2.4286	-0.7981
C	-6.1108	3.7336	1.2323
O	-4.1156	2.0755	2.5436
H	-5.5492	4.2233	2.0225
H	-6.3440	0.6973	-0.3034
H	-8.0868	1.9296	-1.5869
H	-7.2929	5.4570	0.7410
H	-8.5505	4.3056	-1.0547
H	-4.7056	0.5758	1.3237
H	-6.2074	-3.2440	-1.5398
H	-5.4432	-1.9825	-3.4174
C	-5.9720	-2.2089	-1.2794
H	-6.8942	-1.7302	-0.9398
C	-5.3537	-1.4391	-2.4732
H	-3.2852	-2.1284	-2.3347
C	-3.8968	-1.2538	-2.0618
C	-4.8830	-2.1603	-0.1930
H	-4.3512	-3.1199	-0.1174
H	-5.8339	-0.4631	-2.5929
O	-3.9489	-1.1416	-0.6225
H	-5.2475	-1.8711	0.7964
H	-3.4263	-0.3460	-2.4484

Id

E(r^2scan-3c) = -1473.066491 (conv)

Lowest Freq. = 2.71 cm^{-1}

72

C	-1.2342	1.3503	2.9338
C	-1.9321	0.7091	4.2104
C	-2.3129	0.3591	2.8622
C	0.1356	1.0069	2.4804
C	0.9536	2.0132	1.9557
C	2.2381	1.7215	1.5058
C	2.7237	0.4179	1.5707
C	1.9152	-0.5911	2.0926
C	0.6309	-0.3023	2.5415
O	-2.7634	-0.5393	2.1709
H	-1.5171	2.3932	2.8160
H	-2.5139	1.4212	4.7871
H	-1.4072	-0.0562	4.7836
H	0.5785	3.0324	1.9042
H	2.8608	2.5163	1.1042
H	3.7260	0.1886	1.2206
H	2.2867	-1.6107	2.1496
H	0.0152	-1.1050	2.9416
Li	-2.2719	-0.8821	0.3145
C	-1.5364	2.5986	-1.8434
C	-0.0712	2.1858	-1.6689
C	-0.1886	0.7351	-1.2142
O	-1.4769	0.6349	-0.5406
C	-2.1924	1.8908	-0.6696

H	-1.9369	2.2172	-2.7899
H	-1.6852	3.6811	-1.8090
H	0.4006	2.7905	-0.8864
H	0.5162	2.2808	-2.5858
H	-0.1960	0.0398	-2.0637
H	0.5931	0.4410	-0.5061
H	-2.0759	2.4636	0.2617
H	-3.2525	1.6603	-0.8234
C	-0.0304	-4.2232	1.4236
C	0.4303	-4.1567	-0.0359
C	-0.0005	-2.7531	-0.4381
O	-1.2048	-2.4803	0.3328
C	-1.3654	-3.4966	1.3638
H	0.6699	-3.6839	2.0719
H	-0.1352	-5.2456	1.7960
H	-0.0947	-4.9082	-0.6365
H	1.5071	-4.3034	-0.1550
H	0.7595	-2.0085	-0.1666
H	-0.2457	-2.6528	-1.5004
H	-2.1786	-4.1710	1.0628
H	-1.6399	-2.9939	2.2964
C	-4.8277	1.6289	1.6469
C	-5.8525	2.3879	0.9318
C	-6.5672	1.7392	-0.0829
C	-7.7992	3.7631	-0.4944
C	-7.0876	4.4144	0.5176
C	-7.5402	2.4273	-0.7962
C	-6.1147	3.7326	1.2310
O	-4.1171	2.0743	2.5387
H	-5.5514	4.2224	2.0200
H	-6.3500	0.6966	-0.3049
H	-8.0969	1.9281	-1.5836
H	-7.2988	5.4555	0.7430
H	-8.5605	4.3038	-1.0497
H	-4.7069	0.5757	1.3172
H	-6.2027	-3.2392	-1.5598
H	-5.4462	-1.9640	-3.4313
C	-5.9724	-2.2042	-1.2943
H	-6.8973	-1.7310	-0.9544
C	-5.3550	-1.4271	-2.4837
H	-3.2877	-2.1200	-2.3480
C	-3.8979	-1.2453	-2.0723
C	-4.8853	-2.1561	-0.2057
H	-4.3539	-3.1159	-0.1294
H	-5.8345	-0.4498	-2.5961
O	-3.9497	-1.1374	-0.6326
H	-5.2517	-1.8673	0.7831
H	-3.4262	-0.3369	-2.4562

TS2d

E(r^2scan-3c) = -1473.054004 (conv)

Lowest Freq. = -304.92 cm^{-1}

72

C	-1.4888	1.6484	3.2208
C	-2.3608	0.7780	4.0651
C	-2.9328	0.5151	2.7256
C	-0.2395	1.2584	2.6358
C	0.5201	2.2316	1.9462
C	1.7472	1.9181	1.3851
C	2.2530	0.6195	1.4757
C	1.5111	-0.3578	2.1400
C	0.2826	-0.0526	2.7101
O	-2.7761	-0.4773	1.9894
H	-1.7745	2.6927	3.1449
H	-3.0040	1.3129	4.7613
H	-1.8982	-0.1043	4.5045
H	0.1332	3.2455	1.8735
H	2.3176	2.6887	0.8733
H	3.2158	0.3733	1.0374
H	1.8990	-1.3699	2.2196
H	-0.2723	-0.8331	3.2210
Li	-2.1403	-0.9319	0.2552
C	-1.4645	2.5083	-2.0086
C	-0.0093	2.0321	-1.9705
C	-0.1588	0.5764	-1.5495

O	-1.3463	0.5333	-0.7105
C	-2.0581	1.7958	-0.8022
H	-1.9547	2.1704	-2.9291
H	-1.5653	3.5945	-1.9361
H	0.5516	2.5887	-1.2118
H	0.5027	2.1316	-2.9312
H	-0.3222	-0.0819	-2.4140
H	0.6873	0.2056	-0.9622
H	-1.8932	2.3576	0.1287
H	-3.1269	1.5794	-0.9089
C	-0.0043	-4.1867	1.6333
C	0.5664	-4.2269	0.2119
C	0.1336	-2.8752	-0.3405
O	-1.1287	-2.5727	0.3162
C	-1.3468	-3.5074	1.4111
H	0.6307	-3.5749	2.2843
H	-0.1133	-5.1778	2.0816
H	0.1066	-5.0412	-0.3598
H	1.6523	-4.3514	0.1863
H	0.8551	-2.0881	-0.0835
H	-0.0348	-2.8732	-1.4224
H	-2.1167	-4.2287	1.1041
H	-1.7074	-2.9377	2.2731
C	-4.6871	1.2736	1.3624
C	-5.6258	2.2553	0.9685
C	-6.4144	1.9863	-0.1739
C	-7.5031	4.1205	0.0712
C	-6.7255	4.3972	1.2009
C	-7.3426	2.9141	-0.6133
C	-5.7929	3.4817	1.6525
O	-3.9206	1.4828	2.3992
H	-5.1889	3.6972	2.5282
H	-6.2804	1.0452	-0.7019
H	-7.9456	2.7026	-1.4912
H	-6.8544	5.3383	1.7273
H	-8.2322	4.8473	-0.2743
H	-4.5536	0.3456	0.8095
H	-6.3079	-3.2171	-1.5216
H	-5.2994	-2.2533	-3.4598
C	-5.8578	-2.2414	-1.3213
H	-6.6247	-1.5967	-0.8823
C	-5.2578	-1.5878	-2.5937
H	-3.1199	-2.0671	-2.5670
C	-3.8029	-1.2827	-2.2075
C	-4.6618	-2.3602	-0.3766
H	-4.1321	-3.3153	-0.5150
H	-5.7943	-0.6695	-2.8475
O	-3.7884	-1.2774	-0.7635
H	-4.9029	-2.2287	0.6818
H	-3.4417	-0.3080	-2.5452

IId

E(r^{2}scan-3c) = -1473.057181 (conv)
Lowest Freq. = 15.26 cm^{-1}
72

C	-1.4550	2.0039	3.4272
C	-2.3639	1.0590	4.1339
C	-2.9922	0.5931	2.8638
C	-0.2331	1.6456	2.8125
C	0.5160	2.6418	2.1286
C	1.7293	2.3437	1.5375
C	2.2393	1.0419	1.5848
C	1.5171	0.0450	2.2450
C	0.3058	0.3318	2.8524
O	-2.6988	-0.4007	2.1997
H	-1.8026	3.0272	3.3157
H	-3.0686	1.5623	4.7965
H	-1.8742	0.2281	4.6444
H	0.1235	3.6551	2.0875
H	2.2878	3.1263	1.0309
H	3.1907	0.8092	1.1154
H	1.9087	-0.9683	2.2868
H	-0.2324	-0.4586	3.3643
Li	-2.0394	-0.8466	0.4543
C	-1.1851	2.4178	-1.9926
C	0.2235	1.8155	-1.9432
C	-0.0386	0.4384	-1.3487
O	-1.1910	0.6040	-0.4762
C	-1.7853	1.9113	-0.6896
H	-1.7422	2.0209	-2.8496
H	-1.1850	3.5095	-2.0496
H	0.8633	2.4021	-1.2746
H	0.7013	1.7552	-2.9245
H	-0.2950	-0.2939	-2.1272
H	0.7902	0.0530	-0.7460
H	-1.5206	2.5573	0.1591
H	-2.8741	1.7909	-0.7239
C	-0.2219	-4.5891	1.1110
C	-0.7744	-4.6300	-0.3171
C	-0.6825	-3.1674	-0.7277
O	-0.9095	-2.4075	0.4925
C	-0.8215	-3.2955	1.6437
H	0.8722	-4.5251	1.0947
H	-0.5130	-5.4549	1.7115
H	-1.8194	-4.9608	-0.3127
H	-0.2031	-5.2819	-0.9832
H	0.3158	-2.9180	-1.1119
H	-1.4384	-2.8653	-1.4613
H	-1.8335	-3.4521	2.0414
H	-0.2068	-2.8060	2.4054
C	-4.6747	1.2258	1.3502
C	-5.6738	2.1345	0.9526
C	-6.4539	1.8127	-0.1866
C	-7.6836	3.8725	0.0560
C	-6.9202	4.2016	1.1813
C	-7.4430	2.6749	-0.6232
C	-5.9260	3.3529	1.6320
O	-3.9401	1.5033	2.4201
H	-5.3370	3.6136	2.5054
H	-6.2684	0.8781	-0.7100
H	-8.0359	2.4174	-1.4960
H	-7.1092	5.1330	1.7072
H	-8.4620	4.5467	-0.2884
H	-4.4636	0.3055	0.8112
H	-5.8966	-3.4245	-1.6295
H	-3.9938	-2.7909	-3.0461
C	-5.4608	-2.4322	-1.4871
H	-6.2689	-1.7183	-1.2889
C	-4.6150	-1.9665	-2.6769
H	-2.7563	-0.7917	-2.5093
C	-3.7452	-0.8850	-2.0484
C	-4.4436	-2.4071	-0.3571
H	-3.8425	-3.3273	-0.3337
H	-5.2127	-1.5815	-3.5072
O	-3.5678	-1.2866	-0.6602
H	-4.8787	-2.2361	0.6333
H	-4.2412	0.0952	-2.0619

TS3d

E(r^{2}scan-3c) = -1473.028883 (conv)
Lowest Freq. = -34.70 cm^{-1}
72

C	0.9079	2.1748	2.8472
C	-0.0767	1.2306	3.4608
C	-0.8591	0.5362	2.3663
C	2.2051	1.8380	2.4447
C	3.0404	2.8464	1.8663
C	4.3224	2.5626	1.4434
C	4.8468	1.2693	1.5696
C	4.0545	0.2655	2.1389
C	2.7682	0.5304	2.5696
O	-0.4358	-0.3654	1.6829
H	0.5787	3.2021	2.7169
H	-0.7748	1.7608	4.1130
H	0.4250	0.4413	4.0312
H	2.6427	3.8532	1.7631
H	4.9300	3.3515	1.0074
H	5.8570	1.0503	1.2378
H	4.4580	-0.7380	2.2514
H	2.1815	-0.2704	3.0096

Li	0.1207	-1.1694	-0.0040
C	1.8939	1.8267	-2.3004
C	3.1667	1.0508	-1.9441
C	2.6155	-0.2523	-1.3730
O	1.2959	0.0713	-0.8479
C	0.9750	1.4547	-1.1489
H	1.4765	1.4720	-3.2501
H	2.0558	2.9058	-2.3676
H	3.7350	1.5884	-1.1773
H	3.8184	0.8761	-2.8041
H	2.4909	-1.0221	-2.1455
H	3.2254	-0.6523	-0.5552
H	1.1715	2.0680	-0.2578
H	-0.0911	1.5049	-1.3939
C	3.0231	-3.9618	0.1226
C	2.0331	-4.7960	-0.6976
C	0.9132	-3.7976	-0.9630
O	0.9162	-2.8928	0.1811
C	2.0863	-3.1535	1.0058
H	3.5984	-3.2963	-0.5315
H	3.7191	-4.5704	0.7057
H	1.6621	-5.6379	-0.1023
H	2.4619	-5.1857	-1.6244
H	1.1021	-3.2072	-1.8700
H	-0.0781	-4.2565	-1.0338
H	1.7720	-3.7234	1.8906
H	2.4952	-2.1889	1.3240
C	-2.5399	1.2061	0.8943
C	-3.6257	2.0324	0.6357
C	-4.0673	2.1676	-0.7152
C	-5.8592	3.6182	-0.0019
C	-5.4327	3.5033	1.3274
C	-5.1665	2.9432	-1.0160
C	-4.3388	2.7285	1.6560
O	-2.1064	1.0684	2.1930
H	-4.0129	2.6470	2.6876
H	-3.5272	1.6457	-1.5012
H	-5.4940	3.0346	-2.0477
H	-5.9688	4.0311	2.1112
H	-6.7214	4.2309	-0.2459
H	-1.9967	0.6585	0.1309
H	-4.3792	-2.6988	-1.8027
H	-2.4291	-2.8557	-3.2879
C	-3.6460	-1.8888	-1.7724
H	-4.1778	-0.9379	-1.6484
C	-2.7436	-1.8433	-3.0101
H	-0.6043	-1.3032	-2.9826
C	-1.5540	-1.0359	-2.5077
C	-2.6315	-2.0758	-0.6555
H	-2.3573	-3.1321	-0.5288
H	-3.2209	-1.3754	-3.8750
O	-1.4525	-1.3391	-1.0865
H	-2.9505	-1.6673	0.3096
H	-1.7225	0.0440	-2.6177

Pd-*trans*
E(r^2scan-3c) = -1473.152186 (conv)
Lowest Freq. = 9.77 cm^{-1}
72

C	-2.6054	2.5927	2.5560
C	-2.5036	1.2381	3.2632
C	-2.5555	0.2492	2.1349
C	-1.2745	3.1971	2.1670
C	-1.1750	4.5870	2.0499
C	0.0094	5.1842	1.6300
C	1.1158	4.3971	1.3178
C	1.0277	3.0127	1.4319
C	-0.1586	2.4156	1.8536
O	-2.1929	-0.9151	2.1176
H	-3.1720	3.3169	3.1470
H	-3.3778	1.0544	3.9010
H	-1.6080	1.0997	3.8718
H	-2.0355	5.2055	2.2962
H	0.0704	6.2661	1.5526
H	2.0434	4.8610	0.9946

H	1.8876	2.3913	1.1980
H	-0.1941	1.3320	1.9383
Li	-1.6937	-1.6702	0.3768
C	-0.1605	0.5950	-2.6632
C	0.9501	-0.4178	-2.2957
C	0.8340	-0.5268	-0.7810
O	-0.5812	-0.3883	-0.5302
C	-1.0269	0.6866	-1.3924
H	-0.7429	0.2484	-3.5210
H	0.2550	1.5752	-2.9120
H	1.9440	-0.0876	-2.6090
H	0.7504	-1.3923	-2.7530
H	1.1532	-1.4856	-0.3649
H	1.3702	0.2902	-0.2752
H	-0.8740	1.6402	-0.8680
H	-2.0964	0.5443	-1.5655
C	-2.0682	-5.0821	1.0741
C	-1.5378	-5.1936	-0.3758
C	-0.4652	-4.0921	-0.4711
O	-0.6412	-3.2602	0.7026
C	-1.0544	-4.1666	1.7515
H	-2.1403	-6.0520	1.5733
H	-3.0576	-4.6139	1.0857
H	-2.3406	-5.0324	-1.0994
H	-1.0962	-6.1744	-0.5715
H	0.5485	-4.5142	-0.4515
H	-0.5647	-3.4458	-1.3487
H	-1.4576	-3.5657	2.5702
H	-0.1758	-4.7264	2.1044
C	-3.4326	2.2124	1.2859
C	-4.9214	2.3679	1.4481
C	-5.4832	3.6212	1.1898
C	-7.6604	2.7911	1.8146
C	-7.1054	1.5399	2.0668
C	-6.8449	3.8329	1.3756
C	-5.7401	1.3274	1.8868
O	-3.0832	0.8155	1.0393
H	-5.3243	0.3423	2.0776
H	-4.8495	4.4332	0.8391
H	-7.2719	4.8101	1.1692
H	-7.7358	0.7217	2.4033
H	-8.7253	2.9537	1.9537
H	-3.0846	2.7689	0.4102
H	-6.2138	-0.9996	-1.4449
H	-4.9849	-1.7127	-3.3655
C	-5.1306	-0.9184	-1.3210
H	-4.9000	0.0821	-0.9424
C	-4.3573	-1.2010	-2.6310
H	-3.3396	-3.1434	-2.5422
C	-3.1922	-2.1117	-2.1948
C	-4.5834	-1.9669	-0.3618
H	-5.0899	-2.9359	-0.4877
H	-3.9951	-0.2751	-3.0858
O	-3.1990	-2.1037	-0.7477
H	-4.6074	-1.6776	0.6920
H	-2.2068	-1.7635	-2.5183

TS4d
E(r^2scan-3c) = -1473.040095 (conv)
Lowest Freq. = -20.20 cm^{-1}
72

C	-0.9340	2.0844	2.0234
C	-2.2581	1.7966	2.6103
C	-3.2806	1.4518	1.5580
C	0.2264	1.3203	2.1774
C	1.4383	1.7614	1.5594
C	2.6117	1.0493	1.6923
C	2.6426	-0.1357	2.4390
C	1.4707	-0.5916	3.0536
C	0.2857	0.1080	2.9295
O	-3.0849	0.9670	0.4541
H	-0.8848	2.9452	1.3599
H	-2.6515	2.6813	3.1340
H	-2.2495	0.9869	3.3508
H	1.4203	2.6820	0.9812
H	3.5175	1.4095	1.2115

H	3.5663	-0.6977	2.5374
H	1.4885	-1.5142	3.6281
H	-0.6112	-0.2837	3.3981
Li	-1.8773	-0.2141	-0.4762
C	0.6498	2.0058	-2.9106
C	1.7265	1.0837	-2.3283
C	0.9019	0.0262	-1.5980
O	-0.3635	0.6670	-1.2643
C	-0.3725	2.0187	-1.7870
H	0.2135	1.5701	-3.8171
H	1.0230	3.0065	-3.1445
H	2.3503	1.6363	-1.6165
H	2.3749	0.6425	-3.0895
H	0.6815	-0.8391	-2.2362
H	1.3737	-0.3147	-0.6700
H	-0.0790	2.7171	-0.9910
H	-1.3927	2.2497	-2.1079
C	-1.5113	-3.3224	2.4044
C	-0.7315	-3.9057	1.2218
C	-0.4052	-2.6575	0.4127
O	-1.5078	-1.7367	0.6503
C	-2.3429	-2.2449	1.7254
H	-0.8249	-2.8734	3.1312
H	-2.1337	-4.0606	2.9171
H	-1.3680	-4.5848	0.6435
H	0.1707	-4.4437	1.5245
H	0.5192	-2.1810	0.7648
H	-0.3355	-2.8376	-0.6654
H	-3.2623	-2.6636	1.2931
H	-2.6041	-1.4086	2.3820
C	-5.5882	1.5152	1.1516
C	-6.8673	1.4532	1.7066
C	-7.9692	1.1833	0.8453
C	-9.4934	1.3454	2.7099
C	-8.4203	1.6053	3.5703
C	-9.2545	1.1351	1.3464
C	-7.1256	1.6597	3.0915
O	-4.5377	1.8152	1.9589
H	-6.2996	1.8580	3.7666
H	-7.7845	1.0212	-0.2136
H	-10.0851	0.9306	0.6767
H	-8.6058	1.7648	4.6289
H	-10.5062	1.3049	3.0987
H	-5.3716	1.3280	0.1062
H	-6.0621	-1.7423	-2.7223
H	-4.2296	-1.4780	-4.3459
C	-5.2887	-1.0004	-2.5067
H	-5.7672	-0.1177	-2.0658
C	-4.4743	-0.5976	-3.7404
H	-2.3270	-0.0762	-3.7329
C	-3.2184	-0.0301	-3.0998
C	-4.2151	-1.5482	-1.5710
H	-4.0583	-2.6254	-1.7096
H	-4.9846	0.1336	-4.3729
O	-2.9794	-0.8663	-1.9371
H	-4.4248	-1.3474	-0.5140
H	-3.3788	1.0080	-2.7721

IIId

E(r^2scan-3c) = -1473.042690 (conv)

Lowest Freq. = 14.00 cm-1

72

C	2.3138	-0.0836	1.5091
C	0.9988	0.5569	1.3098
C	0.3877	0.4169	-0.0541
C	2.6340	-1.4084	1.1903
C	3.9717	-1.8706	1.3927
C	4.3339	-3.1693	1.1018
C	3.3896	-4.0755	0.6016
C	2.0688	-3.6564	0.4115
C	1.6897	-2.3562	0.6901
O	0.9318	0.0167	-1.0669
H	3.0980	0.5298	1.9436
H	1.0446	1.6298	1.5369
H	0.2382	0.1556	2.0040

H	4.7092	-1.1710	1.7783
H	5.3600	-3.4904	1.2598
H	3.6788	-5.0960	0.3693
H	1.3287	-4.3599	0.0389
H	0.6532	-2.0689	0.5464
C	-1.7060	0.6206	-1.1184
C	-2.8989	1.3432	-1.1965
C	-3.7205	1.1715	-2.3466
C	-5.3332	2.7284	-1.4532
C	-4.5348	2.9102	-0.3179
C	-4.9146	1.8544	-2.4635
C	-3.3363	2.2369	-0.1792
O	-0.9467	0.7293	-0.0030
H	-2.7215	2.3877	0.7021
H	-3.3952	0.4910	-3.1299
H	-5.5314	1.7122	-3.3463
H	-4.8590	3.5911	0.4643
H	-6.2721	3.2647	-1.5505
H	-1.3499	-0.0380	-1.9046
H	3.0587	-4.4066	-2.5237
C	3.5764	-3.8997	-3.3467
H	0.0304	-3.5243	-2.2419
H	3.5920	-1.7315	-1.3337
H	-2.0754	-4.6074	-2.7800
H	1.6083	-3.2808	-4.1361
H	3.9712	-4.6526	-4.0342
H	5.2072	-3.3781	-1.9610
C	2.6369	-2.9227	-4.0315
C	-0.8423	-2.8626	-2.3360
C	4.6571	-2.9560	-2.8063
C	3.8577	-1.7179	-2.3957
C	-2.0011	-3.5492	-3.0432
O	2.6137	-1.7695	-3.1568
H	-0.8731	-4.0133	-4.8402
H	-2.9472	-3.0566	-2.7900
C	-1.6546	-3.3156	-4.5175
H	3.0197	-2.6245	-5.0191
O	-0.4807	-1.7421	-3.1927
H	4.3729	-0.7791	-2.6288
H	5.3716	-2.7085	-3.5988
H	-2.5143	-3.4200	-5.1845
C	-1.1180	-1.8915	-4.4935
H	-0.3685	-1.6843	-5.2648
H	-1.9281	-1.1531	-4.5695
O	1.0879	0.7304	-4.1802
H	2.9845	0.7181	-5.0226
H	1.7813	-0.3156	-5.8453
C	1.9441	0.6549	-5.3560
C	1.5070	1.8095	-6.2459
C	0.0090	1.8963	-5.9366
H	-0.5365	1.1057	-6.4653
H	2.0118	2.7353	-5.9473
H	1.7192	1.6187	-7.3010
H	-0.4316	2.8617	-6.1991
Li	1.0825	-0.6613	-2.8622
H	-0.9402	1.1725	-4.0859
C	-0.0155	1.6436	-4.4359
H	0.1558	2.5656	-3.8644
H	-1.0972	-2.4649	-1.3476

TS6e

E(r^2scan-3c) = -768.330775 (conv)

Lowest Freq. = -28.65 cm^{-1}

32

C	2.0829	0.0267	0.4274
C	0.7509	-0.5436	0.7629
C	-0.1576	-0.5788	-0.4435
C	3.3151	-0.4779	0.8411
C	4.5171	0.1813	0.4229
C	5.7593	-0.3023	0.7729
C	5.8793	-1.4557	1.5611
C	4.7219	-2.1117	1.9972
C	3.4675	-1.6417	1.6598
O	0.1522	-0.8981	-1.5633
H	2.0833	0.9474	-0.1527

H	0.2338	0.0104	1.5627
H	0.8299	-1.5838	1.1087
H	4.4309	1.0737	-0.1920
H	6.6526	0.2164	0.4350
H	6.8595	-1.8317	1.8375
H	4.8092	-2.9991	2.6187
H	2.5907	-2.1615	2.0326
C	-2.4339	-0.4333	-1.0179
C	-3.5182	0.4369	-1.0342
C	-4.5823	0.2018	-1.9568
C	-5.7674	2.1336	-1.1208
C	-4.7313	2.3804	-0.2114
C	-5.6788	1.0375	-1.9901
C	-3.6230	1.5584	-0.1579
O	-1.4528	-0.2550	-0.0822
H	-2.8235	1.7605	0.5473
H	-4.5165	-0.6476	-2.6318
H	-6.4797	0.8437	-2.6985
H	-4.7978	3.2316	0.4609
H	-6.6332	2.7878	-1.1531
H	-2.3212	-1.2742	-1.6935

IIIa'

E(r^{2}scan-3c) = -768.341754 (conv)

Lowest Freq. = 22.37 cm^{-1}

32

C	2.6498	0.7404	0.8599
C	1.2224	1.0749	0.7361
C	0.4240	0.1708	-0.1371
C	3.5643	1.4752	1.6258
C	4.9308	1.0637	1.6821
C	5.8603	1.7628	2.4263
C	5.4830	2.9009	3.1498
C	4.1495	3.3258	3.1120
C	3.2056	2.6384	2.3724
O	0.8084	-0.7979	-0.7481
H	3.0090	-0.1339	0.3238
H	1.0578	2.0980	0.3536
H	0.7071	1.0897	1.7136
H	5.2314	0.1807	1.1232
H	6.8931	1.4253	2.4496
H	6.2164	3.4484	3.7339
H	3.8504	4.2084	3.6717
H	2.1779	2.9902	2.3615
C	-1.7950	-0.0720	-0.8973
C	-3.1172	0.3791	-0.9123
C	-4.0740	-0.3335	-1.6903
C	-5.8092	1.2027	-1.0112
C	-4.8809	1.9139	-0.2417
C	-5.3913	0.0777	-1.7326
C	-3.5573	1.5212	-0.1841
O	-0.8871	0.6041	-0.1600
H	-2.8466	2.0800	0.4160
H	-3.7526	-1.2074	-2.2512
H	-6.1077	-0.4780	-2.3314
H	-5.2034	2.7872	0.3189
H	-6.8463	1.5216	-1.0483
H	-1.4431	-0.9393	-1.4446

TS4a'

E(r^{2}scan-3c) = -768.332248 (conv)

Lowest Freq. = -26.07 cm^{-1}

32

C	2.4997	-0.4163	1.6772
C	1.3799	-1.1817	2.3008
C	0.3408	-1.6168	1.2783
C	2.4897	0.9612	1.4523
C	3.6000	1.5825	0.7912
C	3.6131	2.9349	0.5336
C	2.5368	3.7505	0.9214
C	1.4462	3.1747	1.5766
C	1.4105	1.8165	1.8433
O	0.3766	-2.6184	0.6192
H	3.3688	-0.9877	1.3640
H	0.8624	-0.6057	3.0769
H	1.7706	-2.1052	2.7381
H	4.4366	0.9588	0.4858
H	4.4673	3.3762	0.0264
H	2.5572	4.8173	0.7213
H	0.6121	3.7989	1.8877
H	0.5657	1.4022	2.3815
C	-1.3243	-0.5805	-0.0169
C	-1.9411	0.6222	-0.3361
C	-2.5564	0.7656	-1.6174
C	-3.1753	3.0395	-1.0875
C	-2.5830	2.9151	0.1727
C	-3.1604	1.9510	-1.9739
C	-1.9800	1.7327	0.5600
O	-0.6776	-0.6796	1.1837
H	-1.5472	1.6373	1.5484
H	-2.5348	-0.0722	-2.3095
H	-3.6223	2.0457	-2.9526
H	-2.6027	3.7547	0.8624
H	-3.6503	3.9716	-1.3777
H	-1.2489	-1.4261	-0.6916

2a-*cis*

E(r^{2}scan-3c) = -768.454342 (conv)

Lowest Freq. = 37.11 cm^{-1}

32

C	-4.3777	2.2521	0.0178
C	-4.8926	1.0603	-0.7920
C	-3.7004	0.1373	-0.8876
C	-4.5550	2.1195	1.5121
C	-4.5474	0.8811	2.1594
C	-4.6286	0.8054	3.5481
C	-4.7217	1.9662	4.3105
C	-4.7418	3.2058	3.6741
C	-4.6600	3.2784	2.2877
O	-3.6692	-1.0390	-1.1594
H	-4.8216	3.1958	-0.3124
H	-5.1466	1.3658	-1.8154
H	-5.7569	0.5451	-0.3680
H	-4.4752	-0.0424	1.5904
H	-4.6192	-0.1663	4.0341
H	-4.7856	1.9056	5.3934
H	-4.8230	4.1183	4.2584
H	-4.6697	4.2486	1.7960
C	-2.8740	2.2438	-0.4154
C	-1.9103	2.8594	0.5516
C	-1.6669	4.2312	0.4659
C	-0.2707	4.1222	2.4297
C	-0.4991	2.7499	2.5080
C	-0.8556	4.8620	1.4046
C	-1.3175	2.1193	1.5754
O	-2.5656	0.8341	-0.6217
H	-1.4994	1.0514	1.6451
H	-2.1207	4.8090	-0.3366
H	-0.6744	5.9307	1.3307
H	-0.0399	2.1661	3.3010
H	0.3664	4.6119	3.1609
H	-2.7921	2.7388	-1.3925

Synthesis of Starting Materials

Cyclopropanone Ketal SI-1

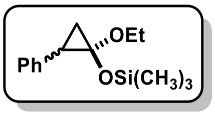

Cyclopropanone Ketal **SI-1a** was prepared according to a previously reported procedure[11]

A solution of LiHMDS in THF (1 M, 45.7 mmol, 45.7 mL, 1.50 eq) was diluted in anhydrous THF (100 mL) under argon atmosphere. Ethyl phenylacetate **SI-2a** (5.00 g, 30.5 mmol, 4.86 mL, 1.00 eq.) was added dropwise and after 15 min of stirring at room temperature the solution was cooled to −78 °C. $(CH_3)_3SiCl$ (19.9 g, 183 mmol, 23.1 mL, 6.00 eq) was slowly added and the mixture was stirred at −78 °C for 1 h. After the cooling was removed the mixture warmed up to room temperature and all volatiles were removed under reduced pressure. The residue was diluted with *n*-pentane (50 mL) and filtered through a pad of celite and the solvent was removed under reduced pressure. The residue was fractionally distilled in vacuum (b.p. = 95 °C/0.7 mbar) affording the silyl ketene acetal (6.23 g, 26.4 mmol, 87%) as a mixture of two isomers as a colorless liquid and was directly submitted to the next step.

FTIR (ATR): $\tilde{\nu}$ [cm^{-1}] = 1645, 1251, 1235, 1185, 1081, 1056.

^{1}H-NMR (400 MHz, $CDCl_3$): δ_H (ppm) = 7.47–7.39 (m, 2H), 7.28–7.18 (m, 2H), 7.06–6.97 (m, 1H), 4.71 (s, 1H), 4.06 (q, *J* = 7.1 Hz, 2H), 1.35 (t, *J* = 7.1 Hz, 3H), 0.33 (s, 6H), 0.31 (s, 3H).

^{13}C-NMR (101 MHz, $CDCl_3$): δ_C (ppm) = 154.4, 137.0, 128.1, 126.6, 123.8, 86.7, 79.3, 62.7, 15.1, 0.6, 0.0.

HRMS (ESI+, Orbitrap): calc. for $C_{13}H_{20}O_2NaSi$ $[M+Na]^+$: 259.1077, found 259.1074.

The silyl ketene acetal (5.37 g, 22.7 mmol, 1.00 eq.) was dissolved in anhydrous Et_2O (0.14 M, 160 mL) under argon atmosphere and cooled to −78 °C. A solution of Et_2Zn in Toluene (1.5 M, 34.1 mmol, 22.7 mL, 1.50 eq) was added dropwise and after 15 min of stirring at −78 °C the cooling was removed and the solution allowed to warm to room temperature (ca. 15 min). CH_2I_2 (9.14 g, 34.1 mmol, 2.75 mL, 1.50 eq) was added dropwise and the mixture was stirred at room temperature for 1 h. The reaction was carefully stopped by slow addition (vigorous gas formation!) of sat. aq. NH_4Cl solution (50 mL) and the suspension was filtered through a pad of celite. The mixture was extracted with EtOAc (3 x 50 ml) and the comb. org. phases were dried over Na_2SO_4, filtered and concentrated under vacuum. The residue was filtered through a short pad of silica (*n*-pentane/EtOAc 4:1) and the solvent was removed under reduced pressure to afford cyclopropanone ketal **SI-1a** (5.65 g, 22.6 mmol, >99%) as a mixture of two diastereomers as a colorless liquid.

***R*$_f$** = 0.3 (*n*-pentane/EtOAc 50:1).

FTIR (ATR): $\tilde{\nu}$ [cm^{-1}] = 1249, 1215, 1187, 1054, 1014.

^{1}H-NMR (400 MHz, $CDCl_3$): δ_H (ppm) = 7.34–7.20 (m, 2H), 7.20–7.07 (m, 3H), 3.72 (q, *J* = 7.1 Hz, 2H), 2.29 (ddd, *J* = 10.2, 7.2, 5.2 Hz, 1H), 1.48 (dd, *J* = 10.7, 6.0 Hz, 1H), 1.43–1.30 (m, 1H), 0.97 (t, *J* = 7.1 Hz, 3H), 0.21 (s, 9H).

^{13}C-NMR (101 MHz, $CDCl_3$): δ_C (ppm) = 138.4/138.2, 128.0/128.0, 127.9/127.5, 125.8/125.7, 89.6/89.1, 62.2/61.9, 31.5/30.7, 21.4/20.9, 15.3/15.0, 0.89/0.53.

HRMS (ESI+, Orbitrap): calc. for $C_{14}H_{26}O_2NSi$ [M+NH$_4$]$^+$: 268.1727, found 268.1726.

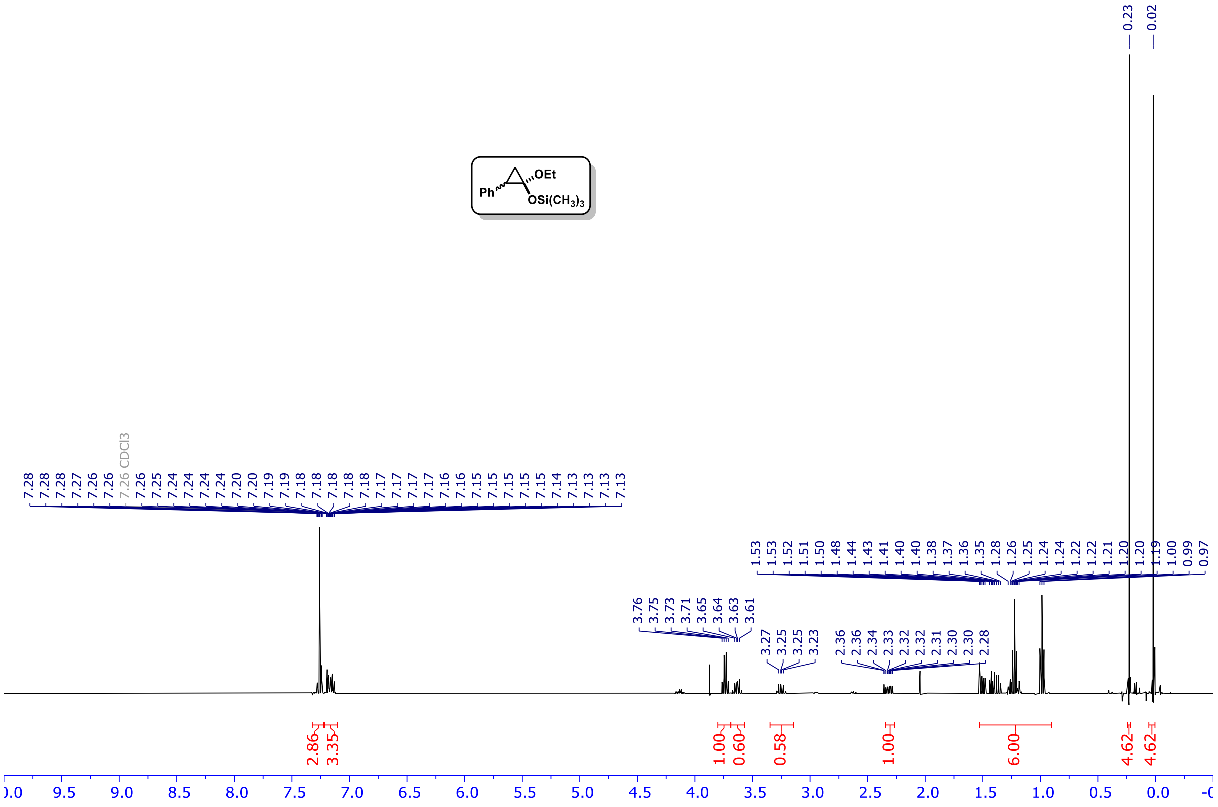

^{1}H-NMR spectrum (400 MHz, $CDCl_3$) of **SI-1a**.

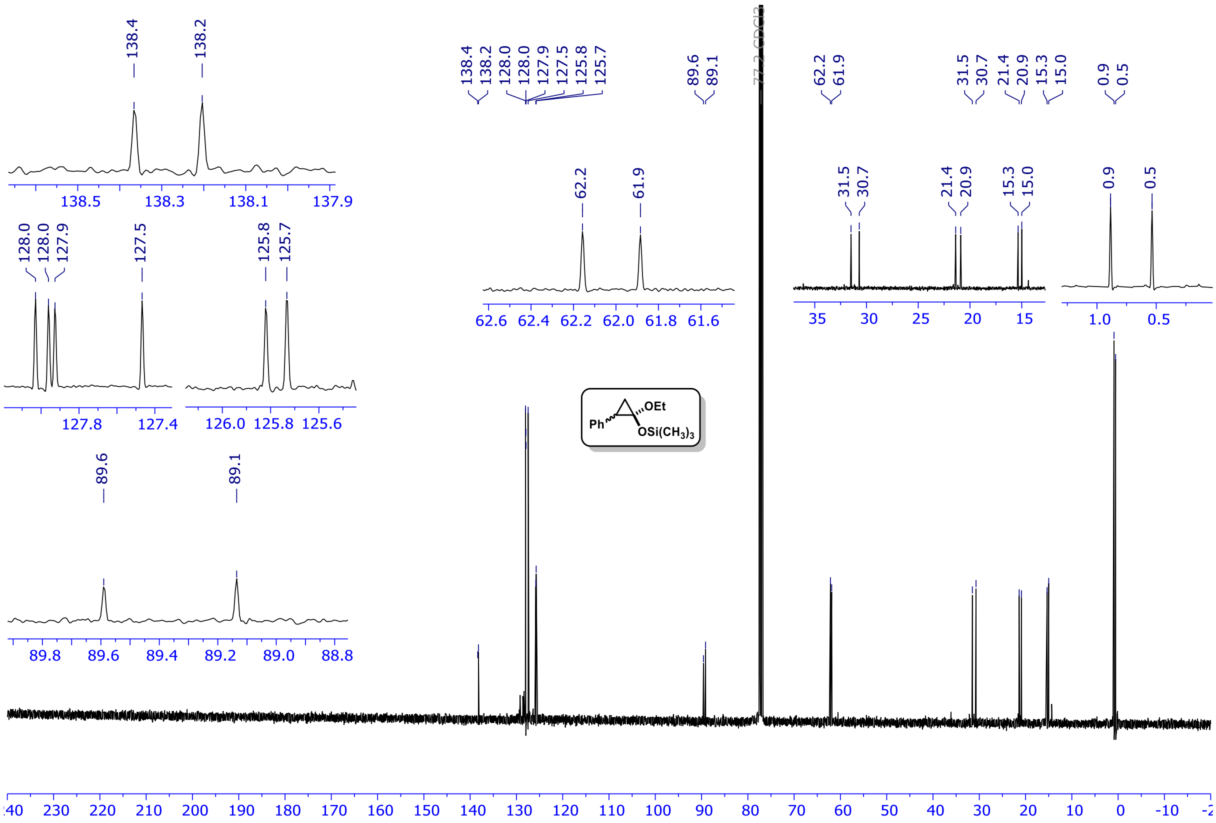

^{13}C-NMR spectrum (101 MHz, $CDCl_3$) of **SI-1a**.

SCP 1a

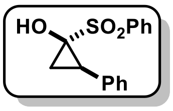

Cyclopropanone ketal **SI-1a** (2.70 g, 10.8 mmol, 1.00 eq.) was dissolved in anhydr. MeOH (9 mL), aq. HCl (37%, four drops) was added and the solution was stirred at room temperature for 1 h. $PhSO_2Na$ (3.56 g, 21.6 mmol, 2.00 eq.), HCO_2H (108 mmol, 4.07 mL, 10.0 eq.) and H_2O (20 mL) were added consecutively and the mixture was stirred at room temperature for 48 h. EtOAc (20 mL) was added, the phases were separated and the organic phase was washed with sat. aq. NaCl solution (3 x 30 ml). The aqueous phase was extracted with EtOAc (3 x 30 ml) and the combined organic phase was dried over $MgSO_4$, filtered and concentrated in vacuo. The residue was dissolved in $CHCl_3$ (4 mL) by ultrasonication and precipitated by addition of *n*-pentane (20 mL). The precipitate was filtered off, washed with *n*-pentane (20 mL) and dried under vacuum to afford SCP **1a** (1.10 g, 4.01 mmol, 37%) as a colorless solid.

FTIR (ATR): $\tilde{\nu}$ [cm^{-1}] = 3385, 1447, 1299, 1139, 1074.

^{1}H-NMR (400 MHz, $CDCl_3$): δ_H (ppm) = 8.02–7.95 (m, 2H), 7.71–7.66 (m, 1H), 7.63–7.56 (m, 2H), 7.34–7.26 (m, 3H), 7.17–7.13 (m, 2H), 3.28 (dd, *J* = 10.8, 8.0 Hz, 1H), 3.13 (s (br), 1H), 2.04 (dd, *J* = 10.8, 6.8 Hz, 1H), 1.61 (dd, *J* = 8.0, 6.8 Hz, 1H).

^{13}C-NMR (101 MHz, $CDCl_3$): δ_C (ppm) = 137.3, 134.0, 133.0, 129.3, 129.1, 128.8, 128.6, 127.6, 74.0, 28.5, 18.0

HR-MS (GC-APCI, +, Q-TOF): calc. for $C_{15}H_{15}O_3S$ $[M+H]^+$: 275.0736, found: 275.0737.

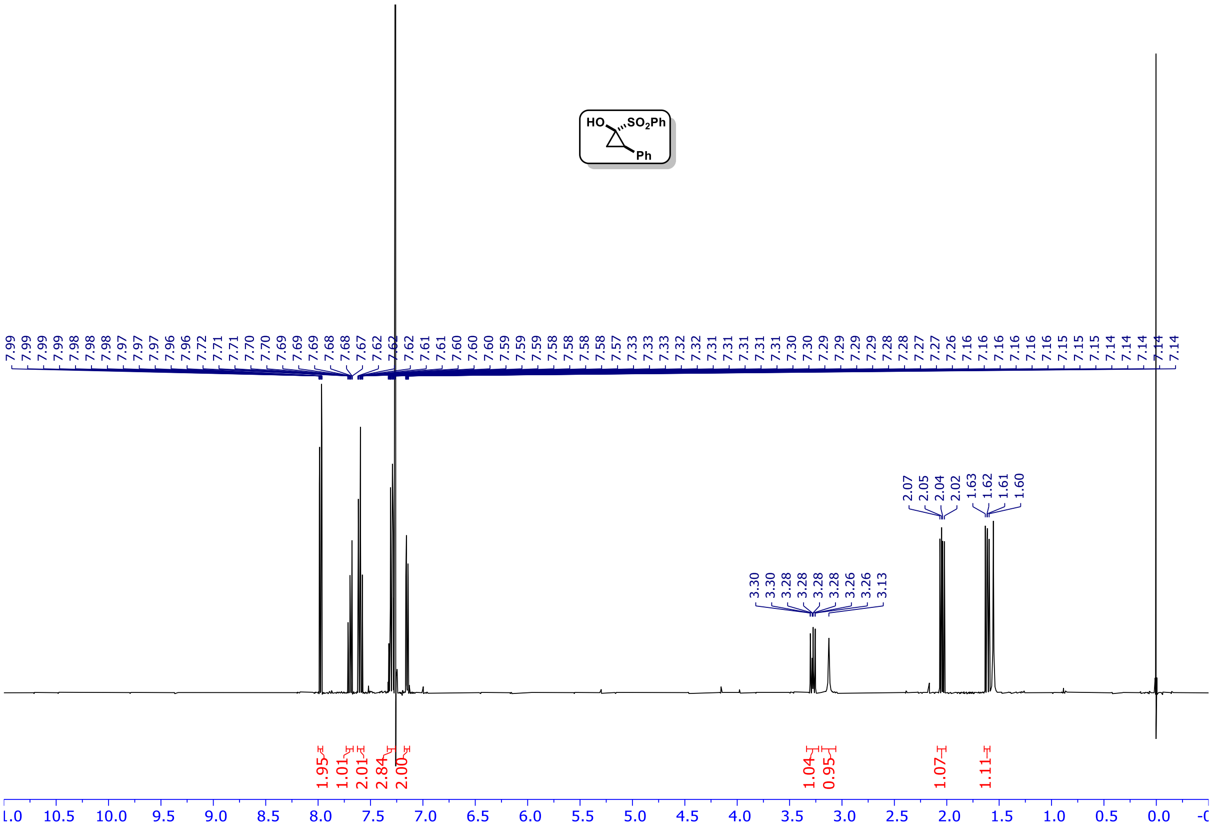

^{1}H-NMR spectrum (400 MHz, $CDCl_3$) of **1a**.

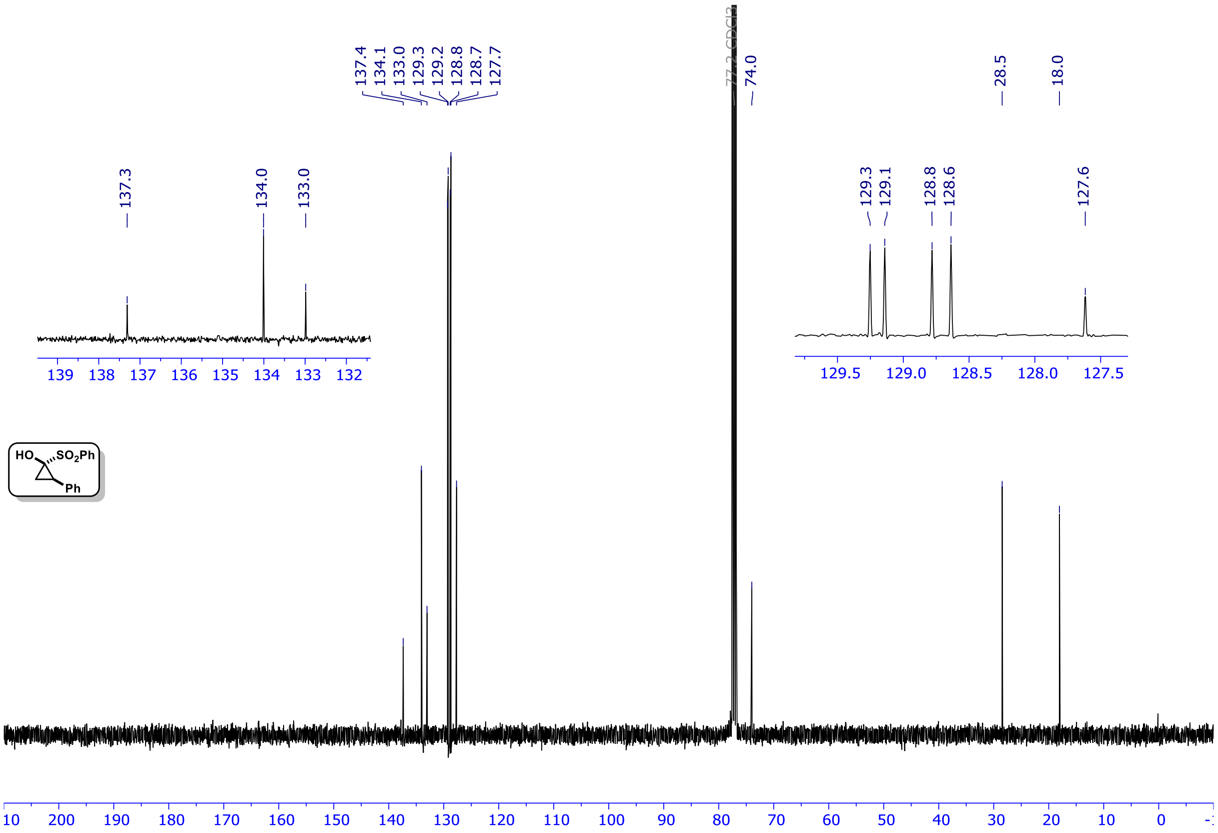

^{13}C-NMR spectrum (101 MHz, $CDCl_3$) of **1a**.

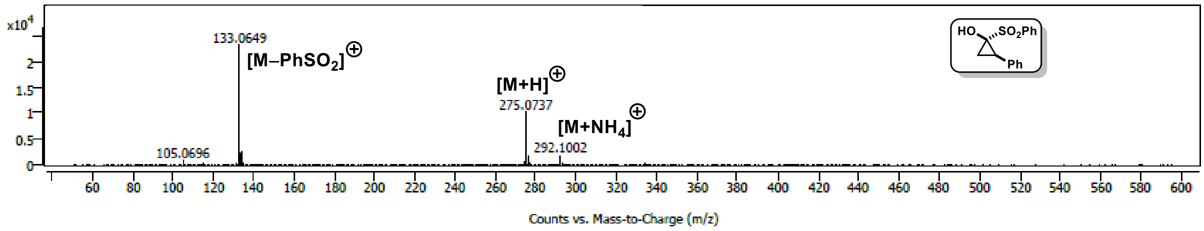

HR-MS Spectrum (APCI,+) of **1a**.

SCP 1b

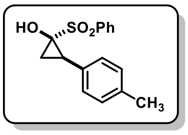

SCP **1b** was prepared according to a previously reported procedure.[12]

FTIR (ATR): $\tilde{\nu}$ [cm^{-1}] = 3385, 2926, 1776, 1759, 1517, 1449, 1300, 1175, 1140, 750, 724, 693.

^{1}H-NMR (500 MHz, $CDCl_3$): δ_H (ppm) = 8.00–7.92 (m, 2H), 7.73–7.65 (m, 1H), 7.63–7.55 (m, 2H), 7.16–7.07 (m, 2H), 7.06–6.99 (m, 2jH), 3.24 (dd, J = 10.8, 8.0 Hz, 1H), 2.31 (s, 3H), 2.02 (dd, J = 10.8, 6.8 Hz, 1H), 1.58 (dd, J = 8.0, 6.8 Hz, 1H).

^{13}C-NMR (126 MHz, $CDCl_3$): δ_C (ppm) = 137.5 (qC), 137.3 (qC), 134.0 (CH), 129.8 (qC), 129.4 (CH), 129.3 (CH), 129.2 (CH), 128.7 (CH), 73.8 (qC), 28.2 (CH), 21.2 (CH_3), 18.0 (CH_2).

HR-MS (GC-APCI, +, Q-TOF): calc. for $C_{16}H_{17}O_3S$ [M+H]$^+$: 289.0893, found: 289.0890.

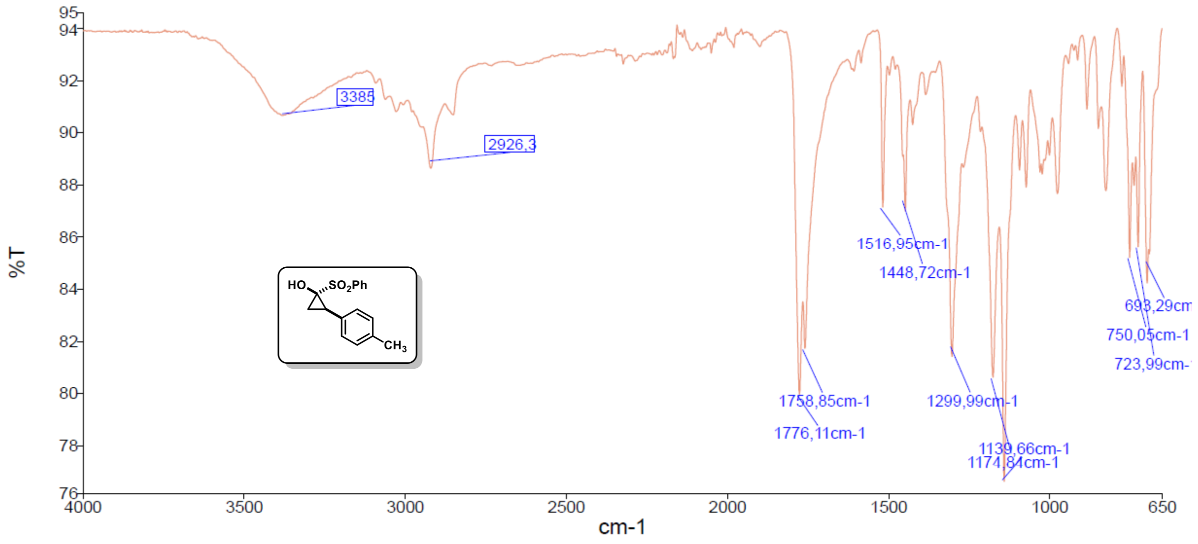

FT-IR Spectrum (ATR, thin film) of **1b**.

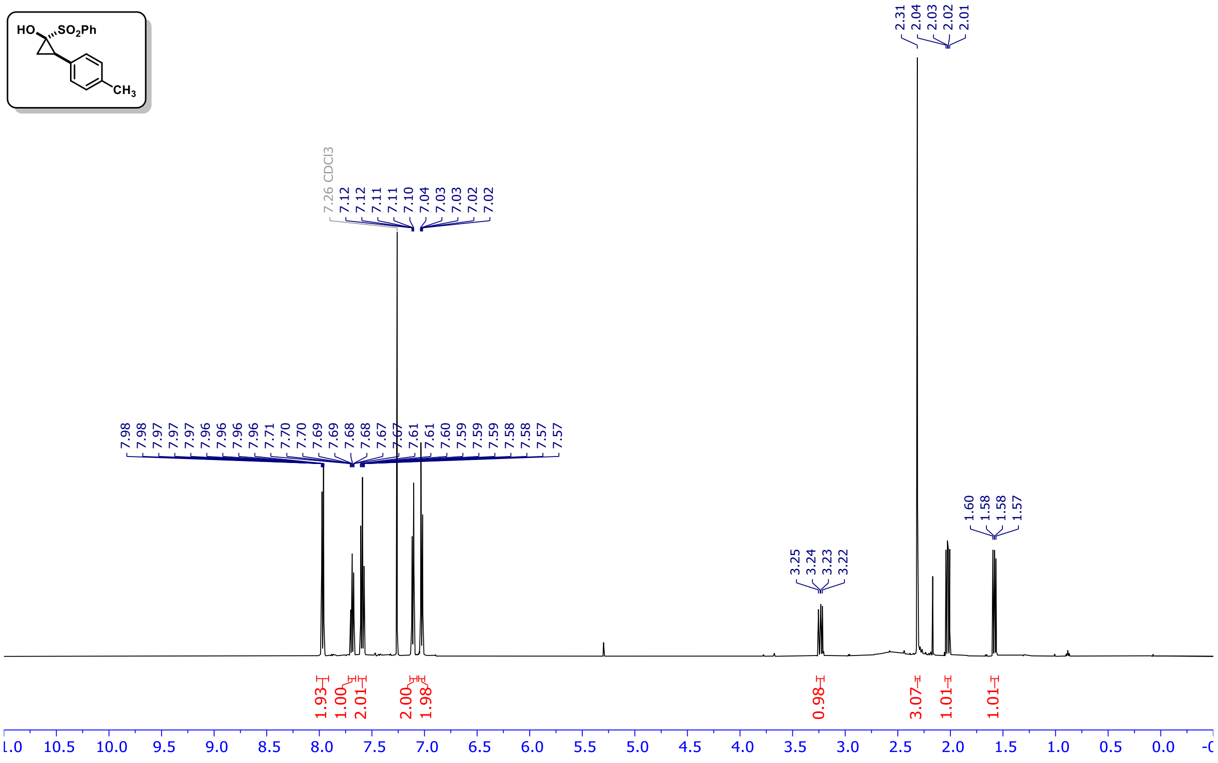

^{1}H-NMR spectrum (500 MHz, $CDCl_3$) of **1b**.

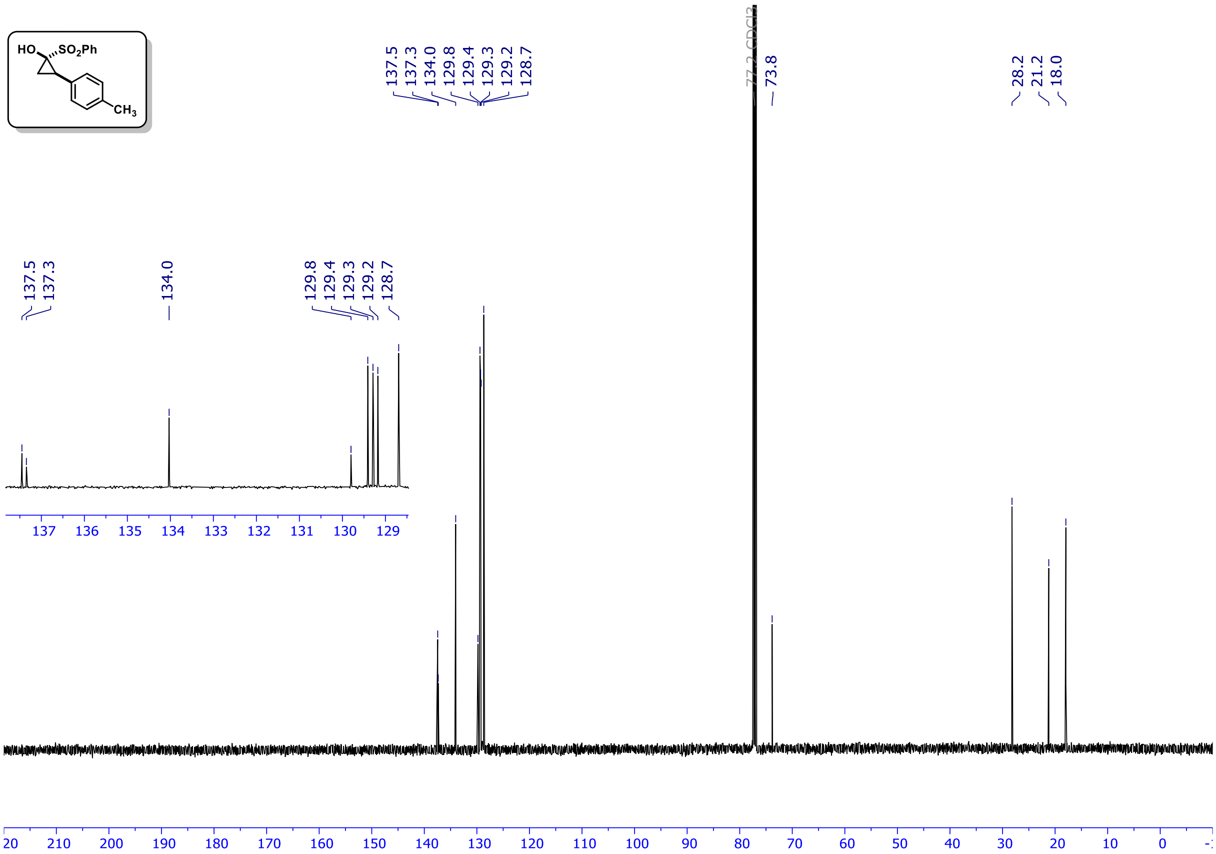

^{13}C-NMR spectrum (126 MHz, $CDCl_3$) of **1b**.

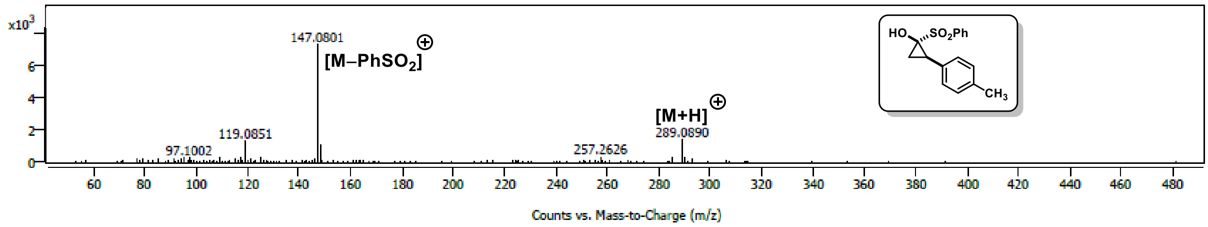

HR-MS Spectrum (APCI,+) of **1b**.

SCP 1c

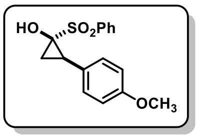

SCP **1c** was prepared according to a previously reported procedure.[12]

FTIR (ATR): $\tilde{\nu}$ [cm^{-1}] = 3346, 1513, 1304, 1239, 1189, 1171, 1138, 1125, 1078, 1030, 834.

^{1}**H-NMR** (700 MHz, $CDCl_3$): δ_H (ppm) = 8.00–7.85 (m, 2H), 7.76–7.65 (m, 1H), 7.62–7.53 (m, 2H), 7.10–7.00 (m, 2H), 6.88–6.78 (m, 2H), 3.76 (s, 3H), 3.25 (s, 1H), 3.20 (dd, J = 10.9, 8.0 Hz, 1H), 2.02 (dd, J = 10.9, 6.7 Hz, 1H), 1.54 (dd, J = 8.0, 6.7 Hz, 1H).

^{13}**C-NMR** (176 MHz, $CDCl_3$): δ_C (ppm) = 159.1, 137.3, 134.0, 129.8, 129.3, 129.1, 124.8, 114.1, 73.8, 55.4, 27.9, 18.0.

HR-MS (GC-APCI, +, Q-TOF): calc. for $C_{16}H_{20}NO_4S$ $[M+NH_4]^+$: 322.1108, found: 322.1108.

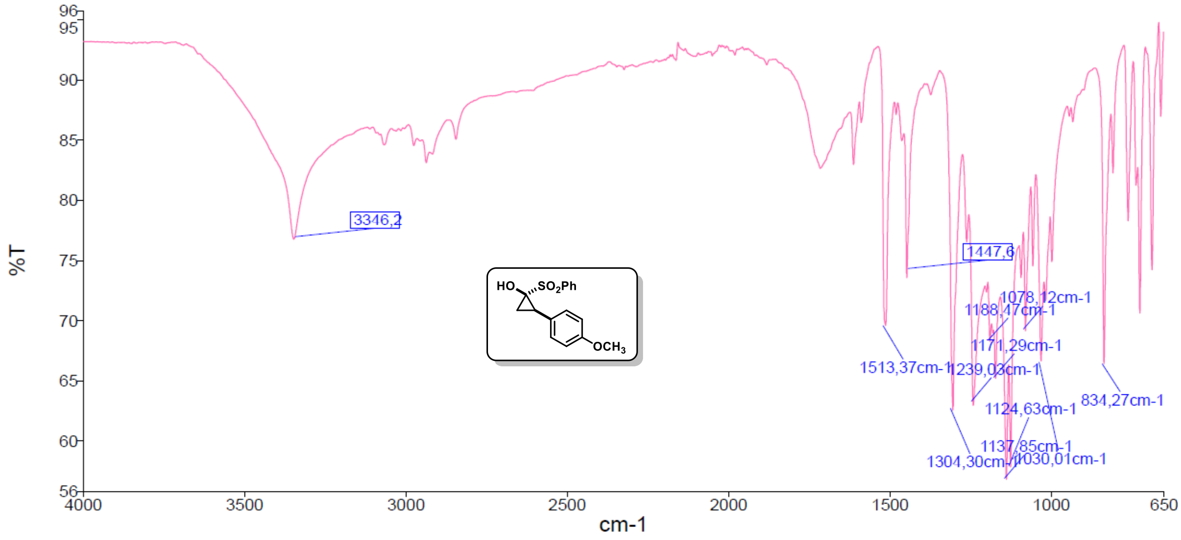

FT-IR Spectrum (ATR, thin film) of **1c**.

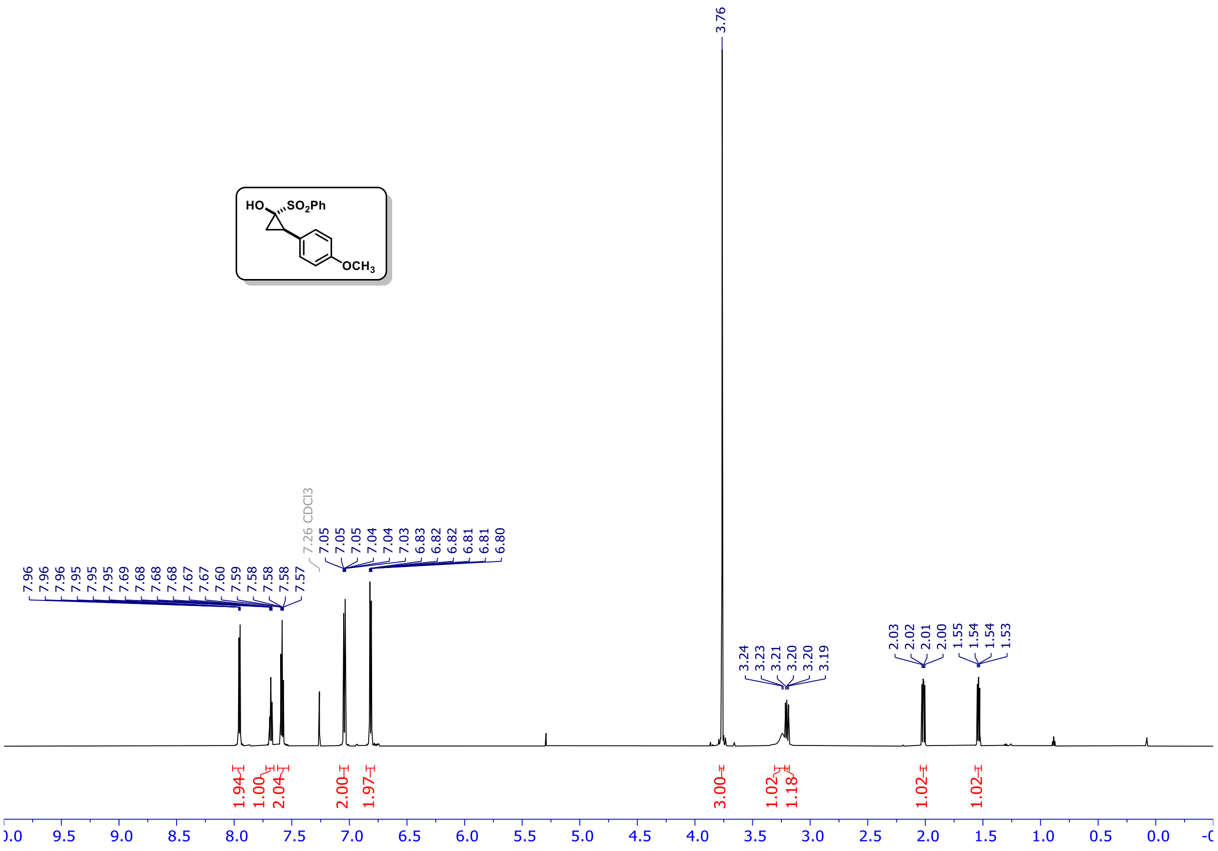

^{1}H-NMR spectrum (700 MHz, $CDCl_3$) of **1c**.

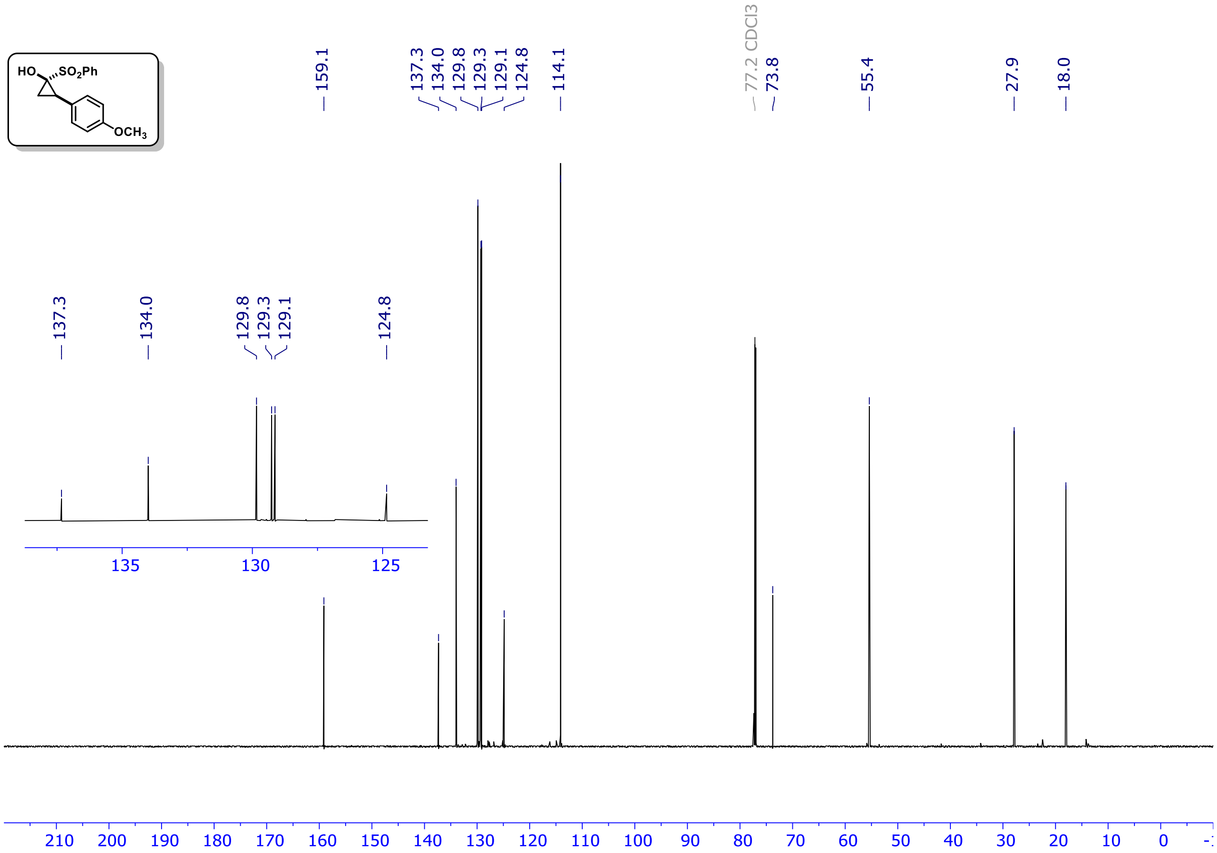

^{13}C-NMR spectrum (176 MHz, $CDCl_3$) of **1c**.

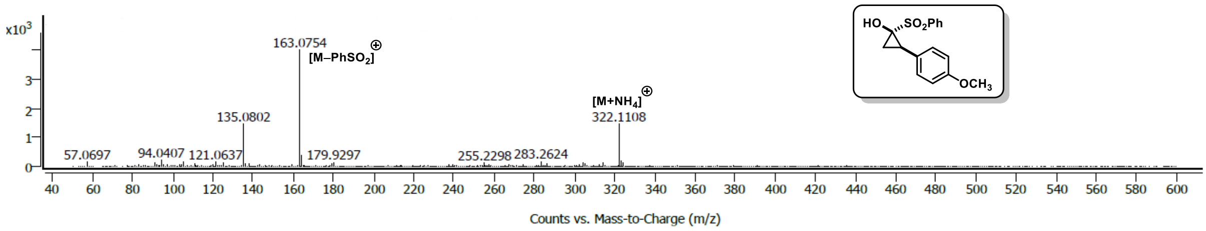

HR-MS Spectrum (APCI,+) of **1c**.

SCP 1d

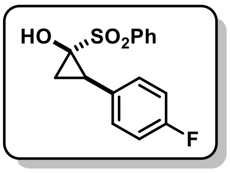

SCP **1d** was prepared according to a previously reported procedure.[12]

FTIR (ATR): $\tilde{\nu}$ [cm^{-1}] = 3379, 1510, 1292, 1248, 1139, 1088, 842, 725, 685, 570, 524.

^{1}H{^{19}F}-NMR (500 MHz, CD_2Cl_2): δ_H (ppm) = 8.04–7.88 (m, 2H), 7.74–7.69 (m, 1H), 7.64–7.60 (m, 2H), 7.12–7.10 (m, 2H), 7.05–6.86 (m, 2H), 3.42 (s(br), 1H), 3.18 (dd, J = 10.9, 7.9 Hz, 1H), 2.06 (dd, J = 10.9, 6.8 Hz, 1H), 1.69–1.47 (m, 1H).

^{13}C-NMR (126 MHz, CD_2Cl_2): δ_C (ppm) = 162.6 (d, J = 245 Hz), 137.5, 134.4, 130.7 (d, J = 8.6 Hz), 129.6, 129.4 (d, J = 3.2 Hz), 129.4, 115.6 (d, J = 21.8 Hz), 74.1, 28.1, 18.3.

^{19}F-NMR (471 MHz, CD_2Cl_2): δ_F (ppm) = −115.8 (m).

HR-MS (APCI, +, Orbitrap): calc. for $C_{15}H_{14}FO_3S$ $[M+H]^+$: 293.0642, found: 293.0639.

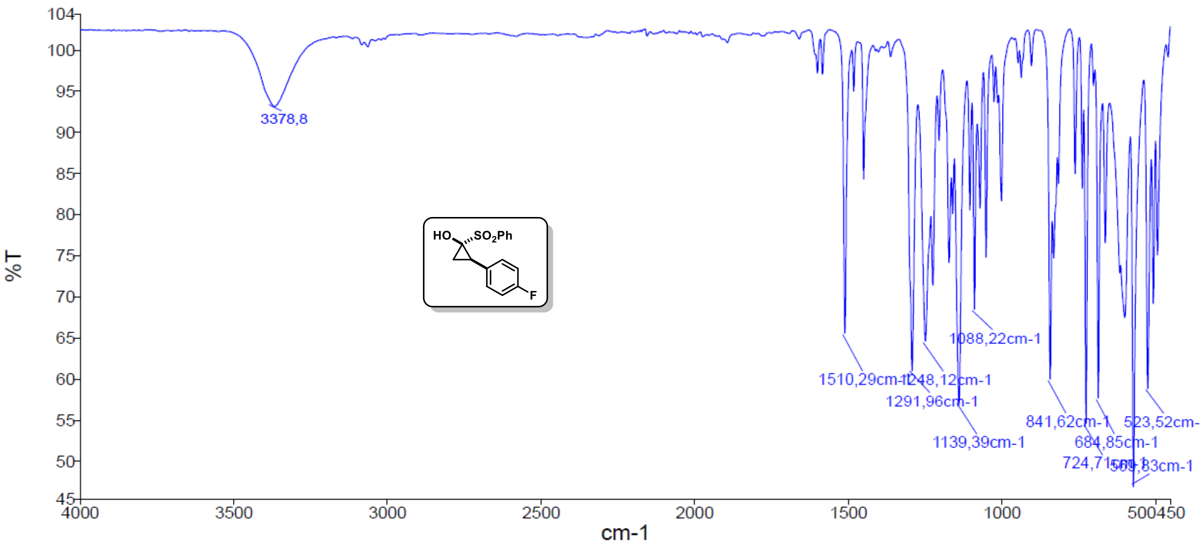

FT-IR Spectrum (ATR, thin film) of **1d**.

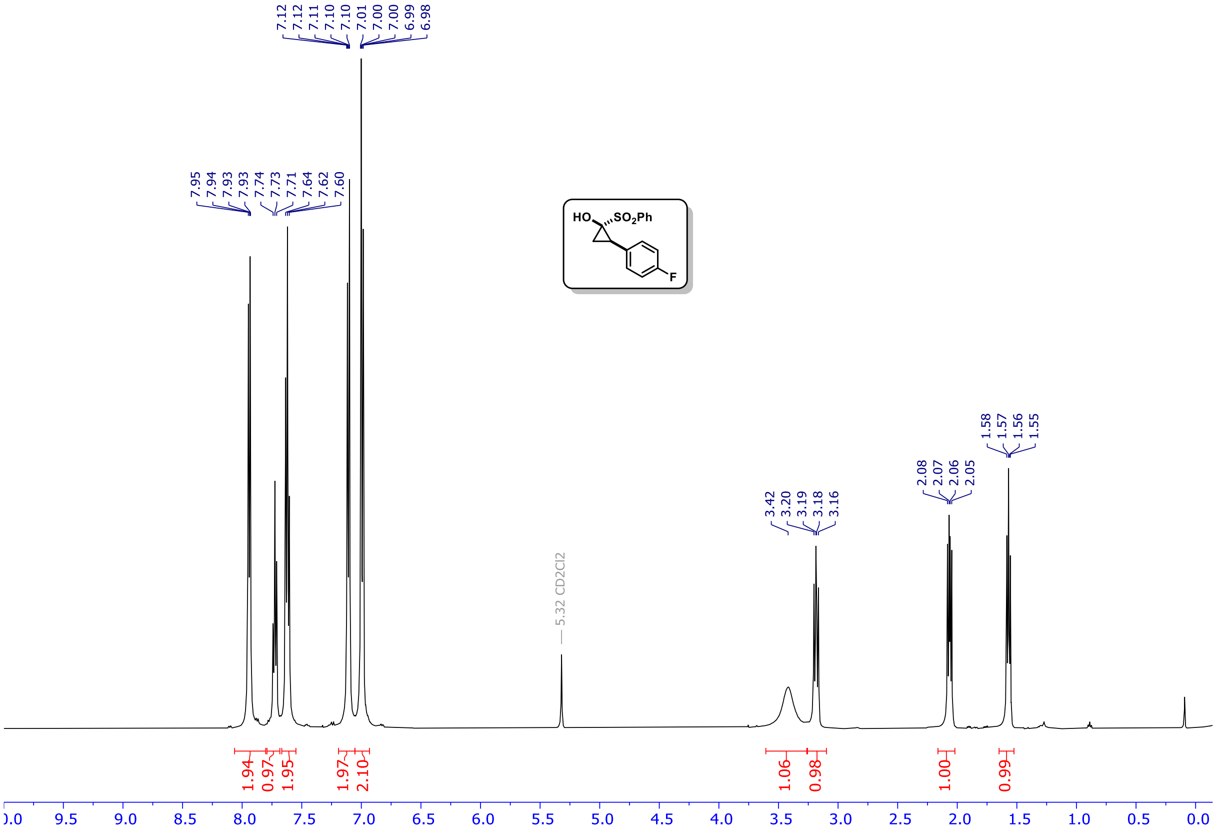

$^1H\{^{19}F\}$-NMR spectrum (500 MHz, CD_2Cl_2) of **1d**.

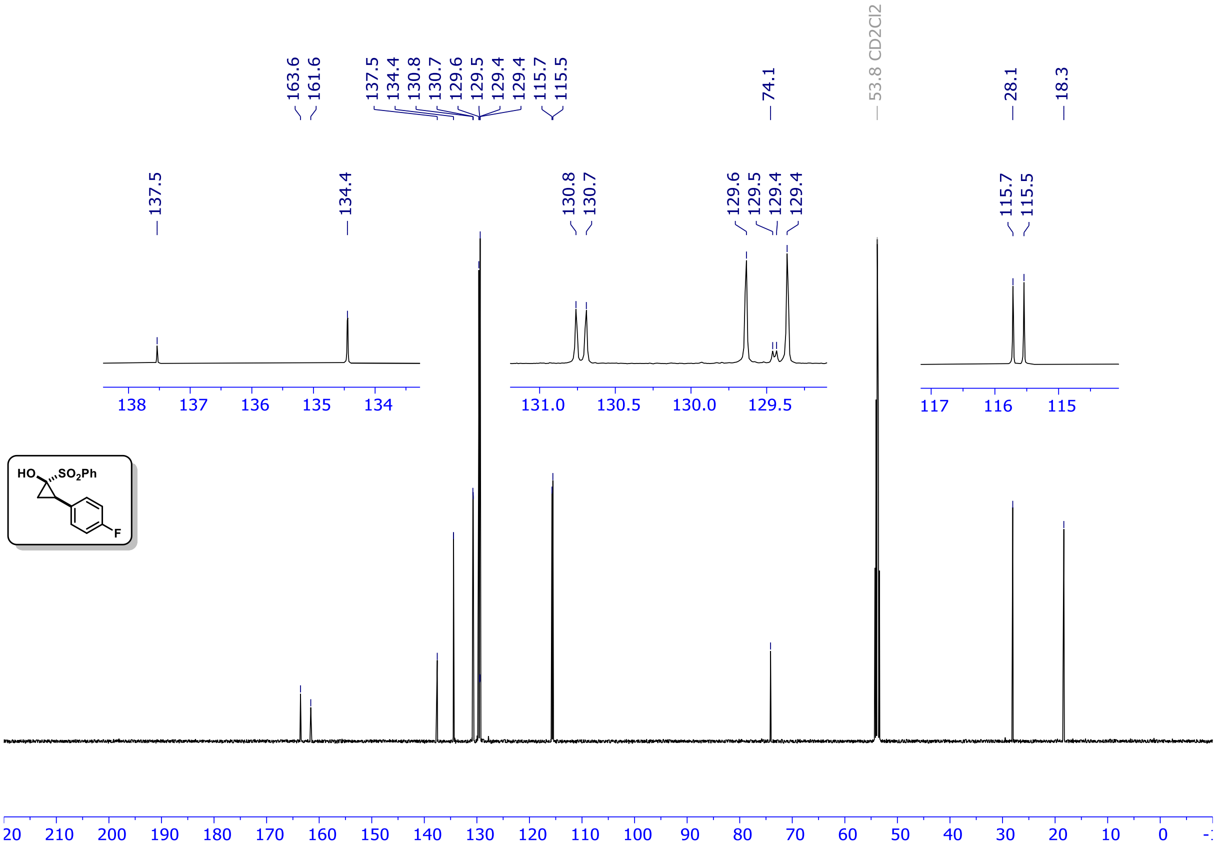

^{13}C-NMR spectrum (126 MHz, CD_2Cl_2) of **1d**.

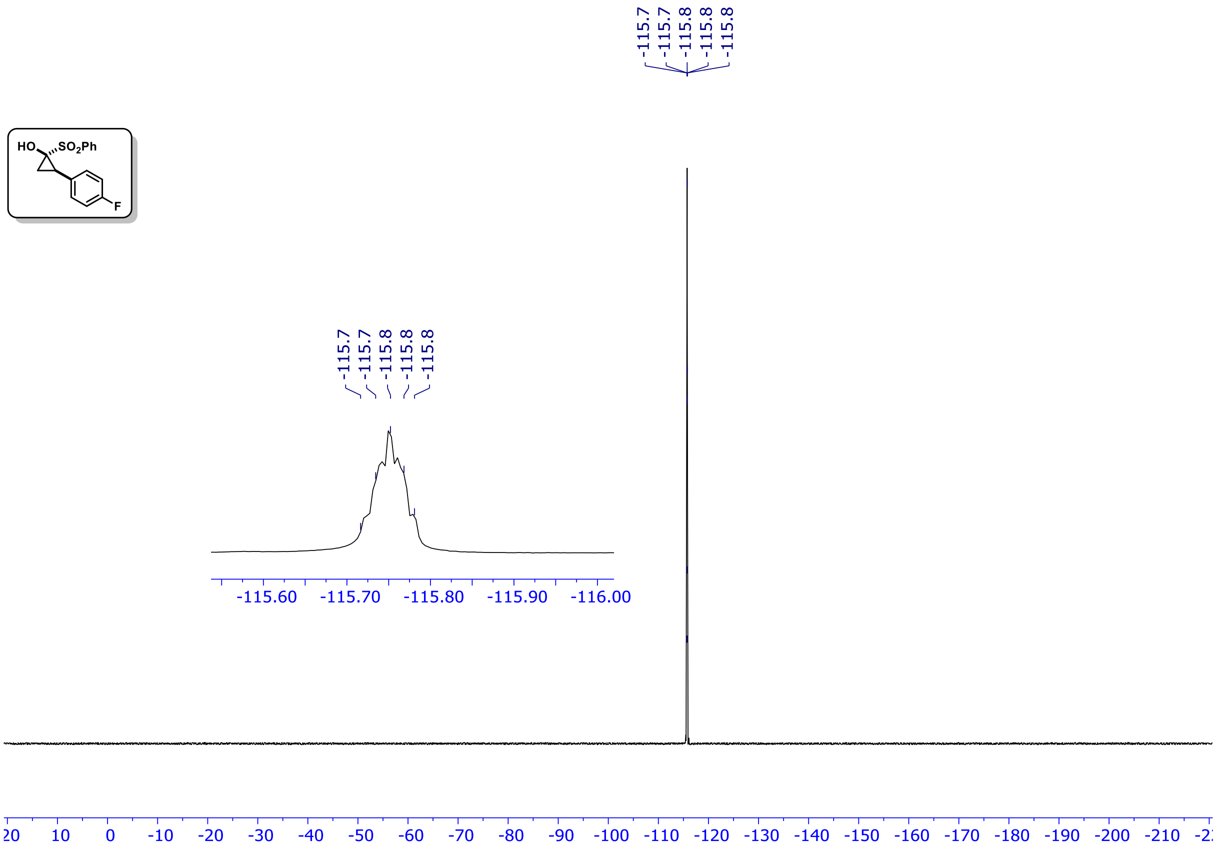

^{19}F-NMR spectrum (471 MHz, CD_2Cl_2) of **1d**.

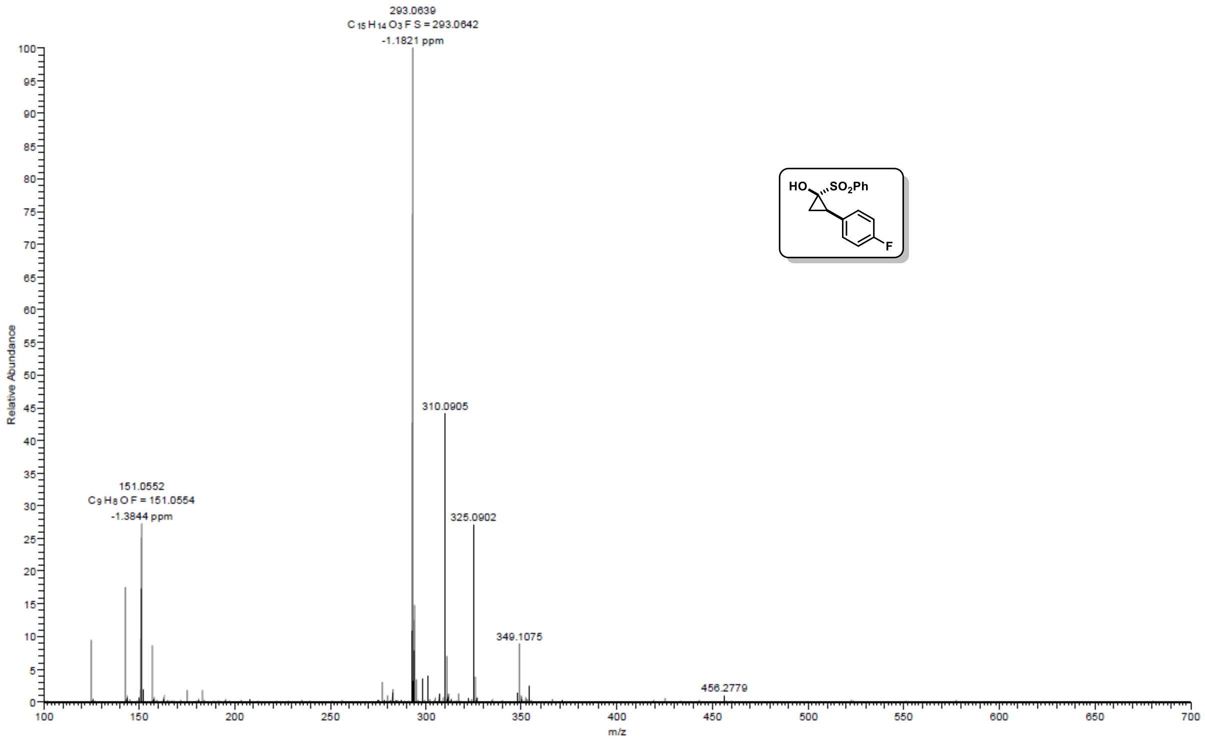

HR-MS Spectrum (APCI,+) of **1d**.

Synthesis of Aryl-substituted SCPs

Our synthesis of racemic SCP **1a** starts from commercially available ethyl phenylacetate **SI-2a**, which is efficiently transferred to the cyclopropanone ketal **SI-1a** following a slightly modified procedure previously reported (Scheme S4).[11] The intermediate silyl ketene acetal (a mixture of two stereoisomers) is conveniently purified by simple distillation and thereafter directly submitted to Simmons-Smith cyclopropanation, affording **SI-1a** as a mixture of two diastereomers (which eliminate in the next step). In this sequence, we prepared up to 5.65 g (22.6 mmol) of the cyclopropanone ketene acetal **SI-1a** in a single reaction batch which was sufficiently pure for submission to the final step after simple filtration through a pad of silica gel. In a manner analogous to the synthesis of unsubstituted SCP,[13,12] **SI-1a** in situ forms a cyclopropanone upon acid-catalyzed methanolysis which is intercepted diastereoselectively by sulfinate to yield the SCP **1a.** After an aqueous work-up, **1a** is conveniently purified by simple precipitation from a $CHCl_3$/*n*-pentane mixture, thereby eliminating the need for column chromatography throughout the entire sequence.

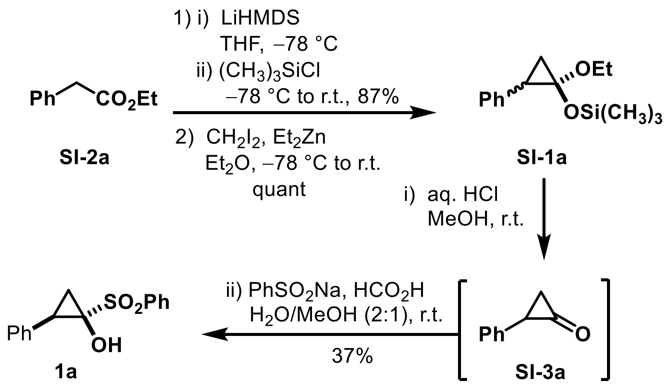

Scheme S4. Synthesis of racemic SCP 1a.

The moderate yield of the final step is attributed to an incomplete conversion of the in situ formed cyclopropanone hemiketal (upon methanolysis of the silyl group) to SCP **1a**. A brief screening of reaction conditions to increase the yield for the transformation of **SI-1a** to SCP **1a** (Table S7) was unfruitful. Furthermore, the propensity of cyclopropanone ketene acetals to eliminate to the neat cyclopropanone **SI-3a** by replacing OEt with a stronger leaving group (pentafluorophenol or trifluoroethanol) did not result in an enhanced yield.

Table S7. Optimization Conditions for the Transformation of Cyclopropanone Ketal SI-1a to SCP 1a.

Entry	Solvent	Acid	$PhSO_2Na$/eq	*T*	Duration	Yield of 1a
1	$MeOH/H_2O$ (1:2, 0.4 M)	HCO_2H (10 eq)	2.0	r.t.	48 h	37%
**2**	$MeOH/H_2O$ (1:2, 0.4 M)	HCO_2H (10 eq)	4.0	r.t.	48 h	21%
**3**	$MeOH/H_2O$ (1:2, 0.4 M)	*p*-TsOH (10 eq)	2.0	r.t.	48 h	n.d.
**4**	$MeOH/H_2O$ (1:1, 0.1 M)	HCO_2H (10 eq)	2.0	r.t.	48 h	n.d.
**5**	$MeOH/H_2O$ (1:2, 0.4 M)	HCO_2H (10 eq)	2.0	50 °C	48 h	traces
**6**	$MeOH/H_2O$ (1:2, 0.4 M)	HCO_2H (10 eq)	2.0	100 °C (MW)	10 min	n.d.
**7**	HCO_2H/H_2O (1:2, 0.4 M)	–	2.0	r.t.	48 h	n.d.
**8**	$MeOH/H_2O$ (1:2, 0.4 M)	HCO_2H (20 eq)	2.0	r.t.	48 h	17%
**9**	$MeOH/H_2O$ (1:2, 0.4 M)	HCO_2H (5.0 eq)	2.0	r.t.	48 h	traces
**10**	$MeOH/H_2O$ (1:2, 0.4 M)	HCO_2H (1.0 eq)	2.0	r.t.	48 h	n.d.

Subsequently, when this method was applied to various 2-aryl substituted cyclopropanone ketene acetals **SI-1b** to **e**, the desired SCPs were not observed. Instead, the 1-aryl-1-sulfonyl acetone derivates **SI-4d** and **SI-4e** were isolated (Scheme S5a). This outcome can be

explained by the equilibrium of 2-subsituted cyclopropanones and oxy allyl intermediates **SI-5**, which is favored by electron-donating substituents on the aromatic ring that stabilize the positive charge in the benzylic position (Scheme S5b).[14]

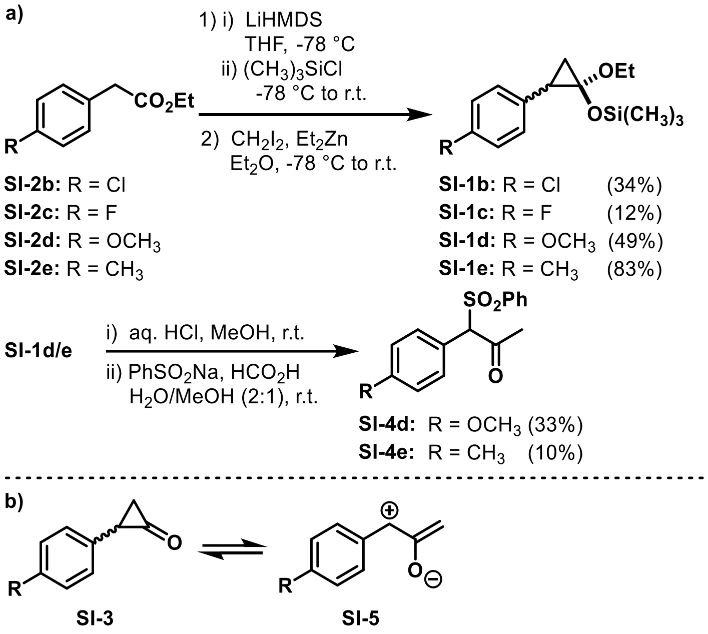

Scheme S5. Studies towards the Synthesis of various 2-aryl substituted SCPs. a) Synthesis of cyclopropanone ketene acetals and subsequent reaction with $PhSO_2Na/HCO_2H$ upon acid-catalyzed methanolysis. b) Equilibrium between Cyclopropanones and Oxy allyls.

1-(4-Methoxyphenyl)-1-sulfonyl acetone SI-4d

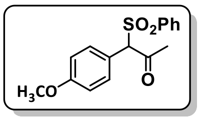

The MeO-substituted cyclopropenone ketene acetal **SI-1d** was reacted according to our procedure for the synthesis of SCPs. However, after precipitation from $CHCl_3$/*n*-pentane, not the desired SCP but the acetone derivative **SI-4d** was obtained as a colorless solid.

FTIR (ATR): $\tilde{\nu}$ [cm^{-1}] = 1716, 1509, 1449, 1362, 1310, 1291, 1276, 1262, 1180, 1143, 1110, 1083, 1018.

^{1}H-NMR (500 MHz, $CDCl_3$): δ_H (ppm) = 7.72–7.54 (m, 3H), 7.47–7.35 (m, 2H), 7.16–7.09 (m, 2H), 6.89–6.77 (m, 2H), 5.17 (s, 1H), 3.80 (s, 3H), 2.36 (s, 3H).

^{13}C-NMR (126 MHz, $CDCl_3$): δ_C (ppm) = 198.3, 160.8, 137.0, 134.1, 131.8, 130.0, 128.7, 119.8, 114.3, 79.8, 55.5, 31.7.

HRMS (APCI, +, Orbitrap): calc. for $C_{16}H_{20}O_4SN$ $[M+NH_4]^+$: 322.1108, found:322.1110.

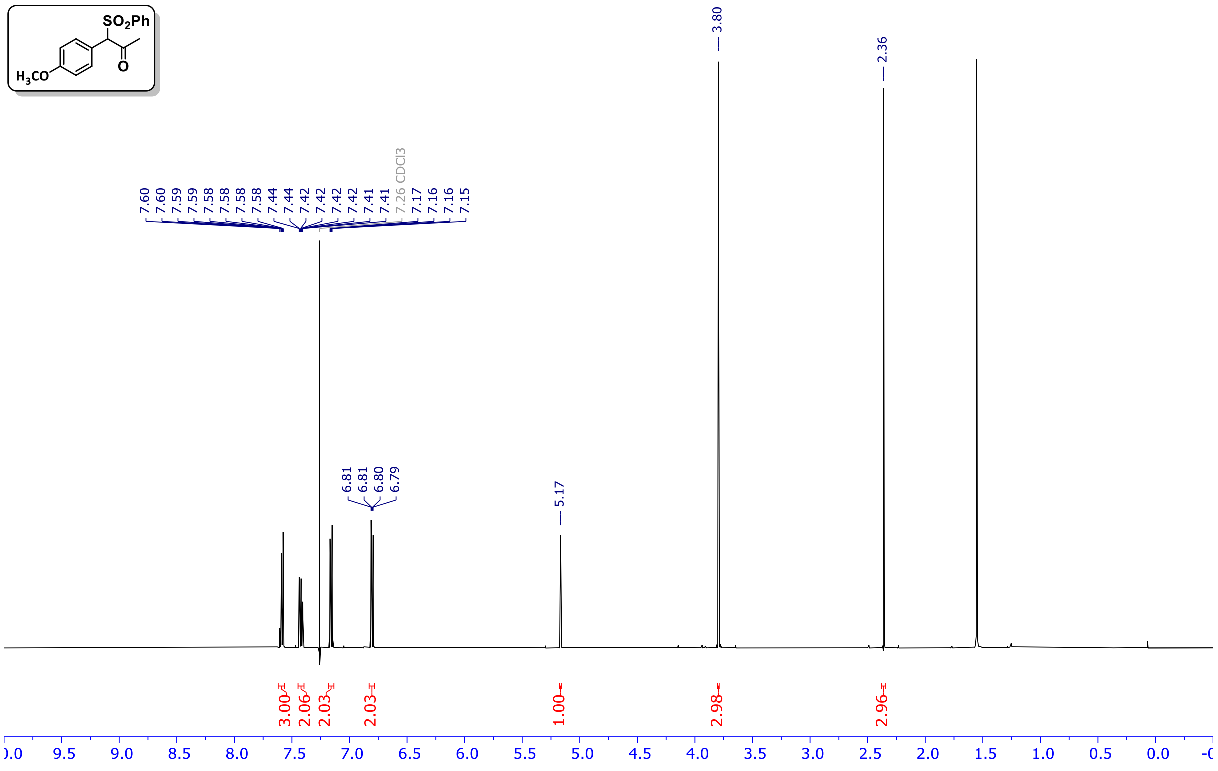

^{1}H-NMR spectrum (500 MHz, $CDCl_3$) of **SI-4d**.

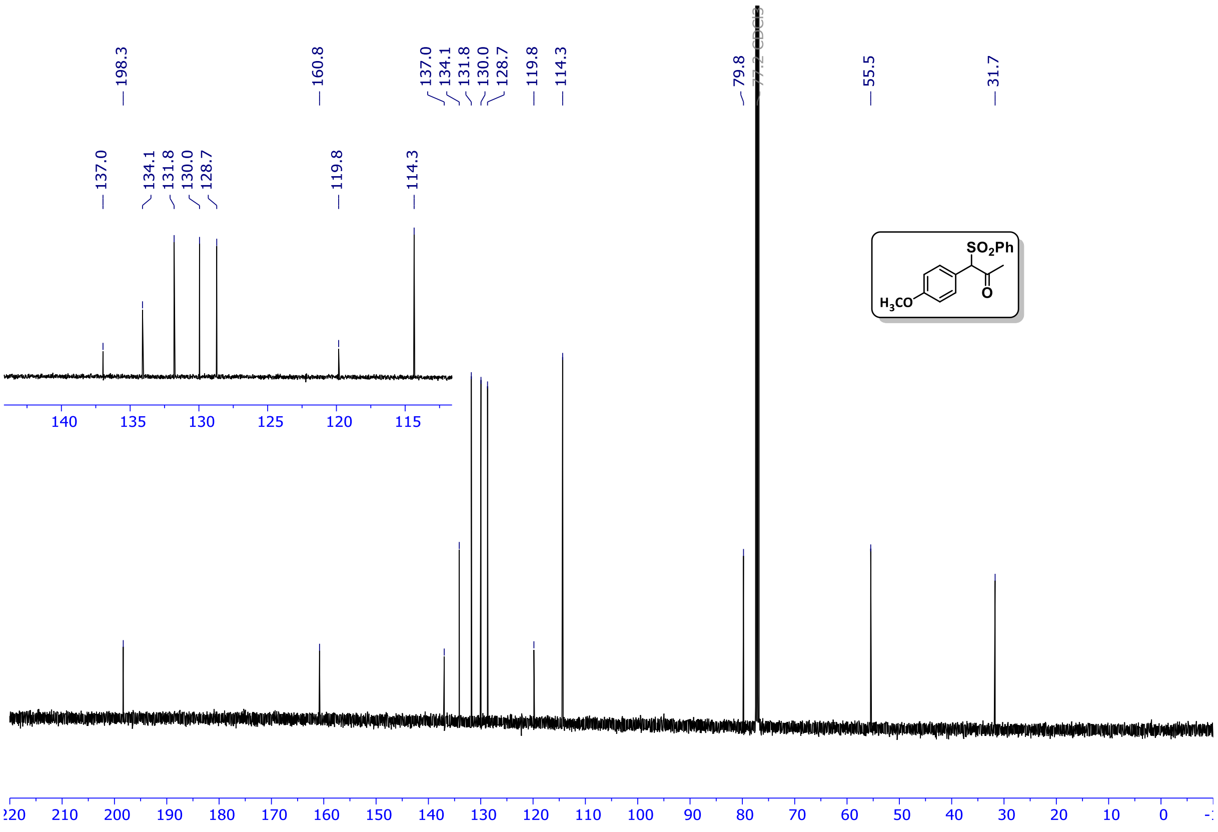

^{13}C-NMR spectrum (126 MHz, $CDCl_3$) of **SI-4d**.

1-(4-Methylphenyl)-1-sulfonyl acetone SI-4e

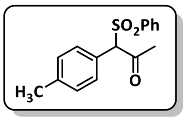

The Me-substituted cyclopropenone ketene acetal **SI-1e** was reacted according to our procedure for the synthesis of SCPs. However, after precipitation from $CHCl_3$/*n*-pentane, not the desired SCP but the acetone derivative **SI-4e** was obtained as a colorless solid.

FTIR (ATR): $\tilde{\nu}$ [cm^{-1}] = 1718, 1510, 1448, 1420, 1359, 1315, 1308, 1297, 1275, 1231, 1177, 1145, 1080, 1024.

^{1}H-NMR (500 MHz, $CDCl_3$): δ_H (ppm) = 7.81–7.53 (m, 3H), 7.52–7.37 (m, 2H), 7.19–7.00 (m, 4H), 5.18 (s, 1H), 2.35 (s, 3H), 2.33 (s, 3H).

^{13}C-NMR (126 MHz, $CDCl_3$): δ_C (ppm) = 198.2, 140.0, 137.0, 134.1, 130.4, 130.0, 129.7, 128.7, 124.9, 80.3, 31.6, 21.4.

HRMS (APCI, +, Orbitrap): calc. for $C_{16}H_{17}O_3S$ $[M+H]^+$: 289.0893, found: 289.0890.

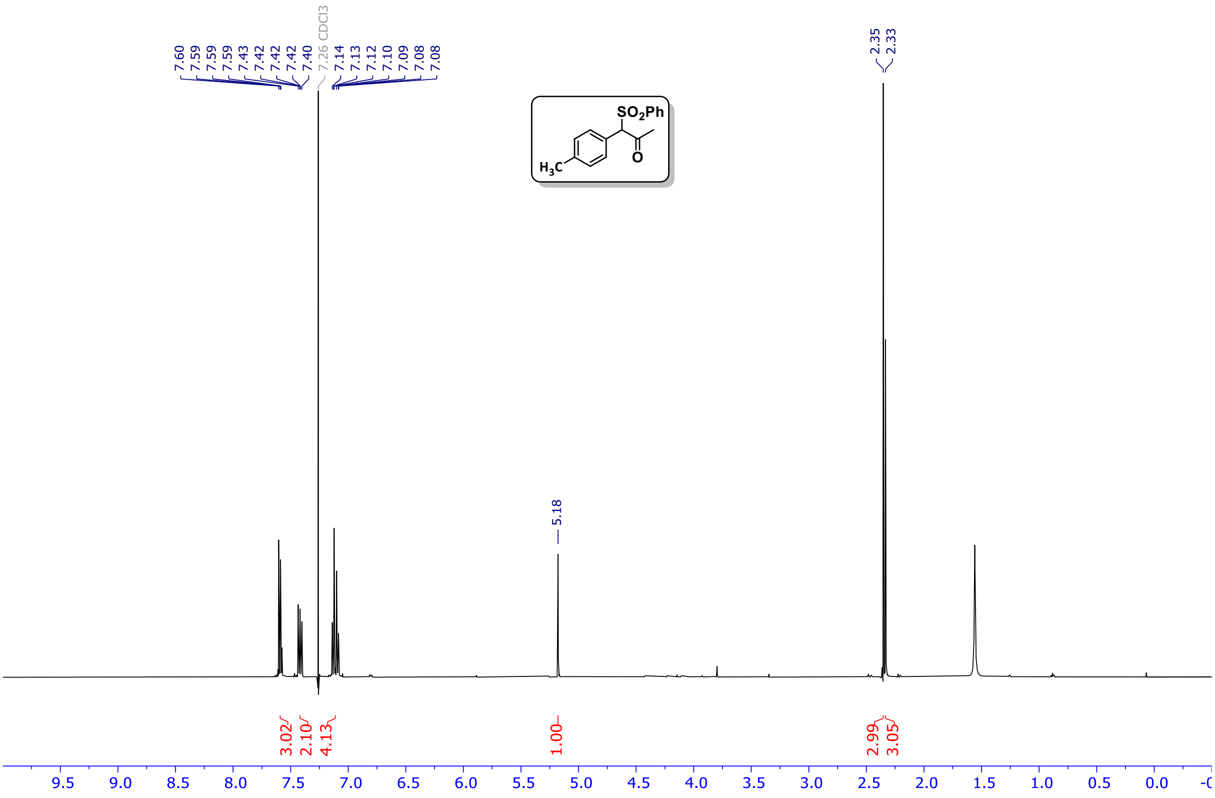

^{1}H-NMR spectrum (400 MHz, $CDCl_3$) of **SI-4e**.

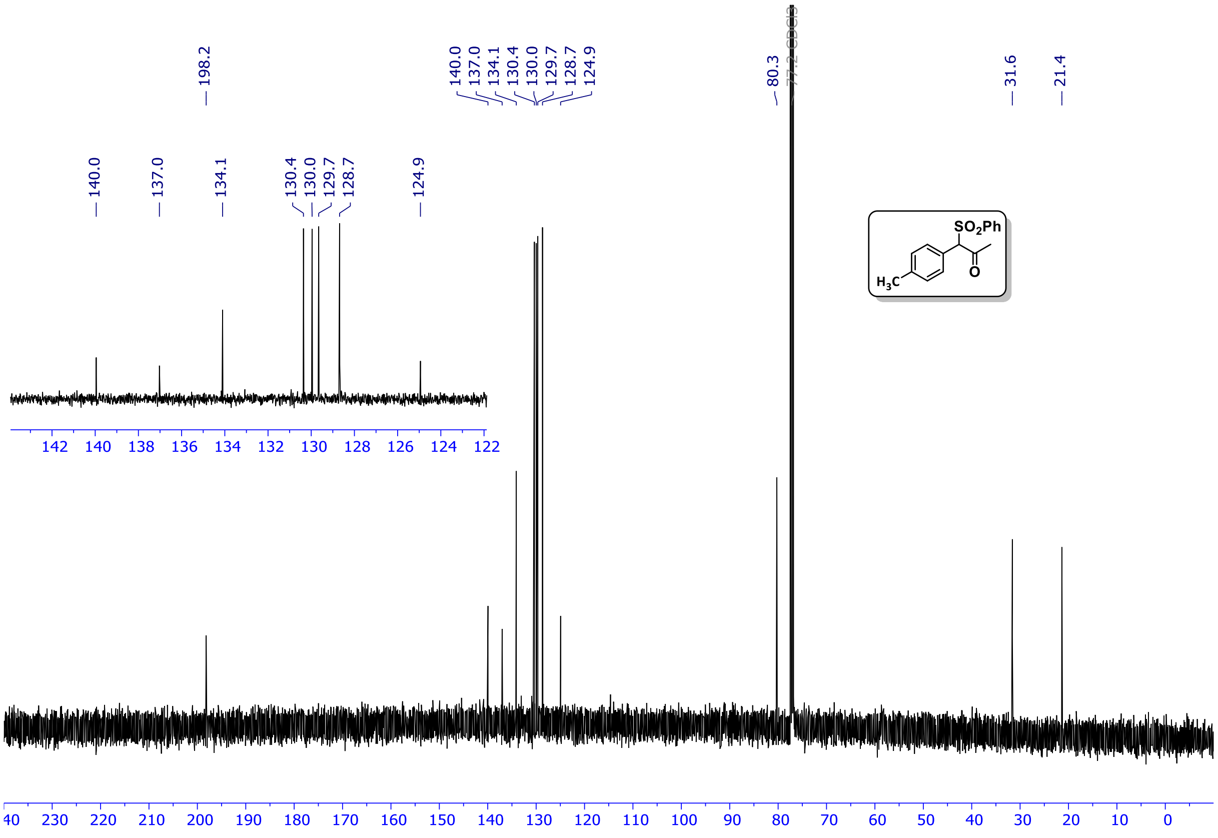

^{13}C-NMR spectrum (126 MHz, $CDCl_3$) of **SI-4e**.

Optimization of Reaction Conditions

Entry	Base (eq.)	PhCHO (eq.)	Solvent (conc.)	Temp (°C)	Duration	Additive (mol%)	NMR yield (%)	*dr*
1	LiTMP (2.0)	4.9	DME (0.1 M)	−40 °C to 80 °C	5 d	TBSOTf (20)	n.d.	–
2	*i*-PrMgCl (1.0)	4.9	THF (0.1 M)	−78 °C to r.t.	1.5 h	TBSOTf (35)	15	1.8
3	*i*-PrMgCl (1.3)	4.9	THF (0.1 M)	50 °C	2 h	TBSOTf (20)	n.d.	–
4	*i*-PrMgCl (1.3)	8.0	THF (0.1 M)	−78 °C	18 h	TBSOTf (30)	traces	–
5	*i*-Pr_2NEt (1.3)	1.7	THF (0.1 M)	r.t.		TBSOTf (30)	n.d.	–
6	*i*-Pr_2NMgBr (1.3)	2.6	THF (0.1 M)	r.t.		TBSOTf (30)	traces	–
7	*i*-PrMgCl (1.0)	5.9	THF (0.1 M)	−78 °C to r.t.	18 h	$BF_3 \cdot OEt_2$ (80)	traces	–
8	*i*-PrMgCl (1.3)	4.5	THF (0.1 M)	−78 °C to r.t.	17 h	$Sm(OTf)_3$ (30)	traces	–
9	*i*-PrMgCl (1.3)	5.8	THF (0.1 M)	−78 °C to r.t.	16 h	$Zn(OTf)_2$ (30)	traces	–
10	*i*-PrMgCl (1.3)	5.5	THF (0.1 M)	−78 °C to r.t.	24 h	TBSOTf (30)	traces	–
11	KHMDS (1.0)	5.4	THF (0.1 M)	−78 °C to r.t.	1.5 h	TBSOTf (30)	traces	–
12	LiHMDS (1.0)	5.5	THF (0.1 M)	−78 °C to r.t.	3 h	TBSOTf (30)	24	1.8
13	LDA (1.0)	5.0	THF (0.1 M)	−78 °C to r.t.	3 h	TBSOTf (30)	traces	1.3
14	LiHMDS (1.0)	4.8	THF (0.1 M)	−78 °C to r.t.	3 h	$Sn(OTf)_2$ (30)	23	1.9
15	LiHMDS (1.0)	5.4	THF (0.1 M)	−78 °C to r.t.	3 h	$Sc(OTf)_3$ (30)	n.d.	–
16	LiHMDS (1.0)	5.4	THF (0.1 M)	−78 °C to r.t.	2.5 h	$Sn(OTf)_2$ (100)	n.d.	–
17	NaHMDS (1.0)	5.5	THF (0.1 M)	−78 °C to r.t.	3 h	$Sn(OTf)_2$ (30)	n.d.	–
18	LiHMDS (1.0)	6.0	Toluene (0.1 M)	−78 °C to r.t.	18 h	$Sn(OTf)_2$ (30)	20	1.9
19	LiHMDS (1.0)	6.4	CH_2Cl_2 (0.1 M)	−78 °C to r.t.	18 h	$Sn(OTf)_2$ (30)	16	1.6
20	LiHMDS (1.0)	5.4	MeOH (0.1 M)	−78 °C to r.t.	18 h	$Sn(OTf)_2$ (30)	n.d.	–
21	LiHMDS (1.0)	5.2	THF (0.1 M)	r.t.	1 h	$Sn(OTf)_2$ (30)	19	2.0
22	LiHMDS (1.0)	5.1	THF (0.1 M)	−20 °C	24 h	$Sn(OTf)_2$ (30)	18	1.7
23	LiHMDS (1.0)	5.9	MeCN (0.1 M)	r.t.	16 h	$Sn(OTf)_2$ (30)	n.d.	–
24	LiHMDS (1.0)	5.3	DME (0.1 M)	r.t.	16 h	$Sn(OTf)_2$ (30)	14	1.6

Entry	Base (eq.)	PhCHO (eq.)	Solvent (conc.)	Temp (°C)	Duration	Additive (mol%)	NMR yield (%)	*dr*
25	LiHMDS (1.0)	6.5	THF (0.1 M)	0 °C to r.t.	24 h	$Sn(OTf)_2$ (10)	44	1.6
26	LiHMDS (1.0)	6.5	THF (0.1 M)	0 °C to r.t.	18.5 h	-	45	1.7
27	LiHMDS (1.0)	7.6	THF (0.1 M)	−20 °C	22.5 h	$Sn(OTf)_2$ (10)	44	1.5
28	LiHMDS (1.0)	7.7	THF (0.1 M)	50 °C	22 h	$Sn(OTf)_2$ (10)	42	1.6
29	LiHMDS (1.0)	7.5	$(ClCH_2)_2$ (0.1 M)	−20 °C	65 h	$Sn(OTf)_2$ (10)	25	1.6
30	LiHMDS (1.0)	6.6	PhCl (0.1 M)	−20 °C	65 h	$Sn(OTf)_2$ (10)	24	1.9
31	LiHMDS (1.0)	6.9	DMF (0.1 M)	−20 °C	18 h	$Sn(OTf)_2$ (10)	54	1.4
32	LiHMDS (1.0)	6.5	THF/DMF (1:1, 0.1 M)	−20 °C	19 h	Sn(OTf)2 (10)	58	1.4
33	LiHMDS (1.0)	7.7	THF/DMF (1:1, 0.1 M)	−20 °C	19 h	$SnCl_4$ (15)	63	1.3
34	LiHMDS (1.0)	8.3	THF/DMF (1:1, 0.1 M)	−20 °C	19 h	$Sn(OTf)_2$ (10), MgI_2 (10)	56	1.3
35	LiHMDS (1.0)	7.2	THF/DMF (1:1, 0.1 M)	−20 °C	19 h	$Sn(OTf)_2$ (10), $ZnBr_2$ (10)	44	1.3
36	LiHMDS (1.0)	4.9	THF/DMF (1:1, 0.1 M)	−20 °C	48 h	$SnCl_4$ (15)	28	1.4
37	LiHMDS (1.0)	7.4	NMP (0.1 M)	−20 °C	18 h	$SnCl_4$ (15)	53	1.3
38	LiHMDS (1.0)	7.8	THF/DMPU (1:1, 0.1 M)	−20 °C	19 h	$SnCl_4$ (15)	21	1.5
39	LiHMDS (1.0)	8.1	THF/DMF (1:1, 0.02 M)	−20 °C	25 h	$SnCl_4$ (15)	71	1.3
40	LiHMDS (1.0)	7.9	THF/DMF (1:1, 0.5 M)	−20 °C	19 h	$SnCl_4$ (15)	51	1.3
41	LiHMDS (1.0)		PhCHO/DMF (1:1, 0.1 M)	−20 °C	19 h	$SnCl_4$ (15)	29	1.3
42	LiHMDS (1.0)	7.9	THF/DMF (1:1, 0.02 M)	−78 °C	48 h	$SnCl_4$ (15)	73	1.3
43	LiHMDS (1.0)	8.2	THF/DMF (1:1, 0.02 M)	−78 °C	48 h	TBSOTf (15)	75	1.2
44	LiHMDS (1.0)	8.2	THF/DMF (1:1, 0.02 M)	−78 °C	19 h	TBSOTf (15)	80	1.2
45	LiHMDS (1.0)	8.0	THF/DMF (1:1, 0.02 M)	r.t.	19 h	TBSOTf (15)	33	1.4
46	LiHMDS (1.0)	8.0	THF/DMF (1:1, 0.02 M)	−20 °C	19.5 h	TBSOTf (15)	67	1.3
47	LiHMDS (1.0)	8.0	THF/DMF (1:1, 0.02 M)	−78 °C	6 h	TBSOTf (15)	76	1.2

References

(1) (a) Neese, F. The ORCA program system. *WIREs Comput Mol Sci* **2012**, *2*, 73–78. (b) Neese, F.; Wennmohs, F.; Becker, U.; Riplinger, C. The ORCA quantum chemistry program package. *J. Chem. Phys.* **2020**, *152*, 224108. (c) Neese, F. Software Update: The ORCA Program System—Version 6.0. *WIREs Comput. Mol. Sci.* **2025**, *15*.

(2) (a) Bannwarth, C.; Ehlert, S.; Grimme, S. GFN2-xTB-An Accurate and Broadly Parametrized Self-Consistent Tight-Binding Quantum Chemical Method with Multipole Electrostatics and Density-Dependent Dispersion Contributions. *J. Chem. Theory Comput.* **2019**, *15*, 1652–1671. (b) Bannwarth, C.; Caldeweyher, E.; Ehlert, S.; Hansen, A.; Pracht, P.; Seibert, J.; Spicher, S.; Grimme, S. Extended tight-binding quantum chemistry methods. *WIREs Comput. Mol. Sci.* **2021**, *11*.

(3) (a) Grimme, S.; Hansen, A.; Ehlert, S.; Mewes, J.-M. r2SCAN-3c: A "Swiss army knife" composite electronic-structure method. *J. Chem. Phys.* **2021**, *154*, 64103. (b) Gasevic, T.; Stückrath, J. B.; Grimme, S.; Bursch, M. Optimization of the r2SCAN-3c Composite Electronic-Structure Method for Use with Slater-Type Orbital Basis Sets. *J. Phys. Chem. A* **2022**, *126*, 3826–3838.

(4) Grimme, S. Supramolecular binding thermodynamics by dispersion-corrected density functional theory. *Chem. Eur. J.* **2012**, *18*, 9955–9964.

(5) (a) Zhao, Y.; Truhlar, D. G. Design of density functionals that are broadly accurate for thermochemistry, thermochemical kinetics, and nonbonded interactions. *J. Phys. Chem. A* **2005**, *109*, 5656–5667. (b) Hellweg, A.; Rappoport, D. Development of new auxiliary basis functions of the Karlsruhe segmented contracted basis sets including diffuse basis functions (def2-SVPD, def2-TZVPPD, and def2-QVPPD) for RI-MP2 and RI-CC calculations. *Phys. Chem. Chem. Phys.* **2015**, *17*, 1010–1017.

(6) (a) Caldeweyher, E.; Bannwarth, C.; Grimme, S. Extension of the D3 dispersion coefficient model. *J. Chem. Phys.* **2017**, *147*, 34112. (b) Caldeweyher, E.; Ehlert, S.; Hansen, A.; Neugebauer, H.; Spicher, S.; Bannwarth, C.; Grimme, S. A generally applicable atomic-charge dependent London dispersion correction. *J. Chem. Phys.* **2019**, *150*, 154122. (c) Caldeweyher, E.; Mewes, J.-M.; Ehlert, S.; Grimme, S. Extension and evaluation of the D4 London-dispersion model for periodic systems. *Phys. Chem. Chem. Phys.* **2020**, *22*, 8499–8512.

(7) (a) Garcia-Ratés, M.; Neese, F. Efficient implementation of the analytical second derivatives of hartree-fock and hybrid DFT energies within the framework of the conductor-like polarizable continuum model. *J. Comput. Chem.* **2019**, *40*, 1816–1828. (b) Garcia-Ratés, M.; Neese, F. Effect of the Solute Cavity on the Solvation Energy and its Derivatives within the Framework of the Gaussian Charge Scheme. *J. Comput. Chem.* **2020**, *41*, 922–939.

(8) Henkelman, G.; Jónsson, H. Improved tangent estimate in the nudged elastic band method for finding minimum energy paths and saddle points. *J. Chem. Phys.* **2000**, *113*, 9978–9985.

(9) Ishida, K.; Morokuma, K.; Komornicki, A. The intrinsic reaction coordinate. An ab initio calculation for HNC→HCN and H−+CH4→CH4+H−. *J. Chem. Phys.* **1977**, *66*, 2153–2156.

(10) Mills, G.; Jónsson, H.; Schenter, G. K. Reversible work transition state theory: application to dissociative adsorption of hydrogen. *Surface Science* **1995**, *324*, 305–337.

(11) Nakamura, E.; Oshino, H.; Kuwajima, I. Trichlorotitanium and alkoxytitanium homoenolates. Preparation, characterization, and utilization for organic synthesis. *J. Am. Chem. Soc.* **1986**, *108*, 3745–3755.

(12) Poteat, C. M.; Jang, Y.; Jung, M.; Johnson, J. D.; Williams, R. G.; Lindsay, V. N. G. Enantioselective Synthesis of Cyclopropanone Equivalents and Application to the Formation of Chiral β-Lactams. *Angew. Chem. Int. Ed.* **2020**, *59*, 18655–18661.

(13) Lange, M.; Werz, D. B. Ring-Enlargement of in Situ Generated Cyclopropanones by the Reaction with Sulfonium Ylides: One-Pot Synthesis of Cyclobutanones. *Org. Lett.* **2024**, *26*, 9871–9876.

(14) (a) Camp, R. L.; Greene, F. D. 1,3-Di-tert-butylallene oxide. Isolation and isomerization to trans-2,3-di-tert-butylcyclopropanone. *J. Am. Chem. Soc.* **1968**, *90*, 7349. (b) Turro, N. J. Cyclopropanones. *Acc. Chem. Res.* **1969**, *2*, 25–32. (c) Greene, F. D.; Sclove, D. B.; Pazos, J. F.; Camp, R. L. Thermal reactions of a cyclopropanone. Racemization and decarbonylation of trans-2,3-di-tert-butylcyclopropanone. *J. Am. Chem. Soc.* **1970**, *92*, 7488. (d) Liberles, A.; Greenberg, A.; Lesk, A. Ring opening of cyclopropanone. *J. Am. Chem. Soc.* **1972**, *94*, 8685–8688. (e) Liberles, A.; Kang, S.; Greenberg, A. Semiempirical calculations on the ring opening of substituted cyclopropanones. *J. Org. Chem.* **1973**, *38*, 1922–1924. (f) Cordes, M. H. J.; Berson, J. A. Thermal interconversion of a pair of diastereomeric cyclopropanones. An upper limit for a cyclopropanone-oxyallyl energy separation. *J. Am. Chem. Soc.* **1992**, *114*, 11010–11011. (g) Regnier, V.; Martin, D. The quest for observation and isolation of oxyallyl derivatives. *Org. Chem. Front.* **2015**, *2*, 1536–1545.
